# Global Evolutionary History and Dynamics of Dengue Viruses Inferred from Whole Genome Sequences

**DOI:** 10.3390/v14040703

**Published:** 2022-03-28

**Authors:** Caleb J. Stica, Roberto A. Barrero, Rachael Z. Murray, Gregor J. Devine, Matthew J. Phillips, Francesca D. Frentiu

**Affiliations:** 1 Centre for Immunology and Infection Control, School of Biomedical Sciences, Queensland University of Technology, 300 Herston Road, Herston, QLD 4006, Australia; calebjoshua.stica@hdr.qut.edu.au; 2 eResearch Office, Division of Research and Innovation, Queensland University of Technology, P Block, 2 George Street, Brisbane, QLD 4000, Australia; roberto.barrero@qut.edu.au; 3 School of Biomedical Sciences, Faculty of Health, Queensland University of Technology, KG-Q Block, 60 Musk Avenue, Kelvin Grove, Brisbane, QLD 4059, Australia; rachael.murray@qut.edu.au; 4 Mosquito Control Lab, QIMR Berghofer Medical Research Institute, 300 Herston Road, Herston, QLD 4006, Australia; greg.devine@qimrberghofer.edu.au; 5 School of Biology and Environmental Science, Queensland University of Technology, R Block, 2 George Street, Brisbane, QLD 4000, Australia; m9.phillips@qut.edu.au

**Keywords:** dengue, *Aedes*, mosquito, arbovirus, evolution, phylogeny

## Abstract

Dengue is an arboviral disease caused by dengue virus (DENV), leading to approximately 25,000 deaths/year and with over 40% of the world’s population at risk. Increased international travel and trade, poorly regulated urban expansion, and warming global temperatures have expanded the geographic range and incidence of the virus in recent decades. This study used phylogenetic and selection pressure analyses to investigate trends in DENV evolution, using whole genome coding sequences from publicly available databases alongside newly sequenced isolates collected between 1963–1997 from Southeast Asia and the Pacific. Results revealed very similar phylogenetic relationships when using the envelope gene and the whole genome coding sequences. Although DENV evolution is predominantly driven by negative selection, a number of amino acid sites undergoing positive selection were found across the genome, with the majority located in the envelope and NS5 genes. Some genotypes appear to be diversifying faster than others within each serotype. The results from this research improve our understanding of DENV evolution, with implications for disease control efforts such as *Wolbachia*-based biocontrol and vaccine design.

## 1. Introduction

Dengue is caused by a mosquito-borne virus of the same name, affecting human populations in tropical and sub-tropical regions of the world. Considered a disease of major public health importance by the World Health Organization (WHO), it causes the highest morbidity and mortality of all arthropod-borne viral (arboviral) diseases, with children being the most affected [1]. There are an estimated 390 million dengue virus (DENV) infections per year in 129 countries, with an estimated 25,000 deaths/year globally [2,3,4]. The virus is transmitted through the bite of infected female *Aedes* spp. mosquitoes, primarily *Aedes aegypti* and to a lesser extent *Aedes albopictus* [2]. Dengue infection results in a wide range of disease manifestations, from asymptomatic to a mild self-limiting flu-like illness, with approximately 1–3% of cases progressing to more severe haemorrhagic disease [2,5]. With no current treatment options other than supportive management and a vaccine that is only recommended in previously DENV-exposed populations, national disease control programs are predominantly focused on timely diagnosis, case management, and vector control [6]. However, an improved understanding of the viral diversification and evolutionary mechanisms of DENV could enhance disease control efforts such as *Wolbachia*-based biocontrol and vaccine development.

DENV is a positive-sense ssRNA flavivirus, with an approximately 11 kb-long genome that encodes for three structural proteins (capsid [C], pre-membrane [prM], and envelope [E]) and seven non-structural proteins (NS1, NS2A, NS2B, NS3, NS4A, NS4B, and NS5) [7]. The virus is maintained in four antigenically diverse serotypes (DENV-1, DENV-2, DENV-3, and DENV-4), which can co-exist in endemic areas of the world [8]. Currently, all four DENV serotypes have spread throughout the tropical and sub-tropical regions of the world and attained hyperendemicity (the circulation of multiple serotypes) worldwide [9,10]. Each DENV serotype is further composed of several genetic variants, called genotypes, frequently defined as strains with less than 6% divergence at the E/NS1 junction [11]. Previous work has suggested that DENV lineages go extinct every 7–10 years and are replaced by new genetic variants, typically resulting in epidemics [1,12,13].

Overall, a multitude of human and mosquito factors contribute to the evolutionary dynamics of DENV, including population immunity, viral and mosquito fitness, vectorial competence and capacity, seasonal variations, and random stochastic events [14,15,16,17,18,19,20,21]. DENV acquires, on average, one mutation per whole genome replication cycle within a vertebrate host, which is mainly attributed to its error-prone RNA-dependent RNA polymerase (RdRp) [22], resulting in genomic diversification within the infected host, otherwise known as variants or quasispecies [23]. While coinfection by multiple DENV serotypes is well documented, recombination events are rare in both intra- and inter-serotypic infections [21,24]. The majority of research into DENV evolution has focused largely on the evolutionary dynamics of single genes, typically the E gene. DENV evolution is driven by strong negative selection pressure, with a lesser role played by positive selection pressure [1,25,26,27]. Several studies have estimated the nucleotide substitution rate of the DENV E gene, and while this rate is variable amongst serotypes, all four evolve at approximately 7.6 × 10^−4^ substitutions/site/year, slower than other RNA viruses [28,29].

There is a wealth of literature on DENV evolution, but most studies have focused on identifying serotypes and genotypes involved in specific outbreaks or epidemics, occurring in particular geographic regions of the world [30,31,32,33,34,35,36]. While these studies are important, there has been a lack of understanding of DENV evolutionary dynamics on the global scale over the last few decades. The reduced costs and enhanced capacity to perform next generation sequencing (NGS), particularly in low resource settings, have increased the number of DENV whole genome sequences available for analysis and allows us to expand our understanding of evolutionary patterns in regions other than the E gene primarily. 

To date, the evolutionary dynamics of DENV across its complete coding sequence and at the global scale have remained relatively unexplored. We hypothesized that with increasing DENV incidence over the last decades, DENV strains have diversified, and the complete coding sequence has undergone positive selection pressures with beneficial mutations shared across genotypes and serotypes. We used publicly available whole genome sequence data alongside 22 older archived isolates from South-East Asia and the Pacific, that were previously lacking whole genome data, to better understand DENV diversity and evolutionary history on the global scale over the last few decades. Our results demonstrate that phylogenetic analysis of the whole genome and the E gene produce similar estimates, with evidence for several amino acids being under positive selection across the genome. Within serotypes, some genotypes appeared to be diversifying at faster rates than others. Further functional characterisation of sites under positive selection is needed to understand the drivers of DENV evolution. 

## 2. Materials and Methods

### 2.1. Sequence Dataset

Whole genome complete coding sequences for each DENV serotype were downloaded from GenBank (https://www.ncbi.nlm.nih.gov/genbank/, accessed on 19 November 2020) and ViPR (viprbrc.org, accessed on 19 November 2020) (accession numbers given in Supplementary Table S1). These two databases were cross referenced by accession number against each other to ensure inclusion of all available sequences in the final analysis dataset. To reduce redundancy and over-representation of some countries, complete coding sequences for each serotype dataset were uploaded to the CID-HIT-EST program [37,38] accessed through Galaxy Australia (usegalaxy.org.au, accessed on 26 November 2020), and clustered using a similarity threshold of 99%. Accessions unique for country of isolation and year were then randomly selected from each cluster. Additionally, the raw reads for a highly divergent DENV4 isolate DKE-121 were downloaded on 18 October 2021, assembled into a consensus sequence, and included in the dataset. 

Recombinant sequences were detected using RDP, GENECOV, BootScan, MaxChi, Chimaera, SiScan, and 3Seq in the Recombination Detection Program, RDP4 v4.101 package [39] and removed from further analysis (recombinant isolates listed by accession number are given in Supplementary Table S2). Resulting sequences were individually interrogated against the associated publication for correct country of origin, date of isolation, and specified genotype. Any accessions without a specified country of origin and date of isolation were excluded from further analysis.

Preliminary analysis was performed to optimize the datasets and reduce sequence redundancy for further analysis by removing sequences from alignments with indels or that caused misalignments and by removing sequences that originated from the same country and year that clustered together on the phylogenetic trees. Sequence alignments were performed using MUSCLE Alignment 3.8.425 [40] with a maximum of eight iterations. Preliminary phylogenetic trees were constructed using the Geneious Tree Builder (Geneious Prime 2019.2.1 software) with the Tamura-Nei genetic distance model, Neighbour-Joining method, and resampling by bootstrap for 1000 replicates, with an outgroup randomly selected from another serotype to anchor the tree. Final alignments using publicly available databases included 494, 492, 286, and 137 sequences from DENV1, DENV2, DENV3, and DENV4, respectively, representing viral isolates collected during 1944–2019 from 93 countries.

### 2.2. Virus Isolates, Viral RNA Extraction and Sequencing

When searching publicly available sequence databases, a gap was observed in the availability of DENV whole genome isolates older than the last two decades. The Queensland University of Technology (QUT) Arbovirus Collection, formerly a WHO Arbovirus Reference Centre established by Professor John Aaskov, contains DENV isolates from the Asia-Pacific region from 1963–2017. Thus, the QUT Arbovirus Collection was interrogated for defined provenance of country of origin and isolation year. Twenty-two of the oldest isolates available were selected for NGS, representing the following low-passage isolates: four isolates from DENV1, eight from DENV2, four from DENV3 and six from DENV4. Sequences are now available on GenBank (accession numbers given in Table 1). 

To obtain enough viral nucleic acid for NGS, DENV isolates were passaged once in C6/36 cell cultures over a period of 7–10 days, and supernatants harvested and concentrated using Amicon^®^ Ultra-15 Centrifugal Filter Units (Merck KGaA, Darmstadt, Germany). Viral RNA was extracted from concentrates using the Quick-DNA/RNA Pathogen Miniprep kit (Zymo Research, Irvine, CA, USA). RNA quality assays were performed using the Bioanalyser (Agilent Technologies, Santa Clara, CA, USA), and viral RNAs were sequenced using an Illumina NovaSeq at the Australian Genome Research Facility (AGRF, Melbourne, Australia). Consensus sequences were produced using BWA 0.7.17 [41], Samtools 1.7, Bcftools 1.9 (htslib.org, accessed on 2 June 2021), and Seqtk 1.3 (github.com/ih3/seqtk, accessed on 2 June 2021) and incorporated into their respective serotype alignments.

### 2.3. Bayesian Evolutionary Analyses

Maximum Clade Credibility (MCC) dated phylogenetic trees were constructed using the Bayesian Markov chain Monte Carlo (MCMC) approach in the BEAST2 package [42]. The complete data sets were evaluated at the level of the whole genome as well as E gene, and further divided into subgroups. These included for each serotype: (1) epidemic isolates, (2) epidemic and sylvatic isolates (for DENV1, DENV2, and DENV4), (3) sylvatic isolates (only for DENV2 given insufficient sample sizes for the other serotypes), and (4) complete datasets (epidemic, sylvatic, and highly divergent isolate [DENV1—Brun 2015, DENV2—QML-22, and DENV4—DKE-121]). The best-fit substitution models were determined using the PhyML platform as described in Section 2.4 below. Datasets were evaluated for phylogenetic relationships with a relaxed uncorrelated lognormal molecular clock and a Bayesian skyline coalescent prior [43]. Analysis was performed using the lowest number of generations that resulted in effective sample size (ESS) values >200 for all statistics, and trees and parameters were sampled every 1000 steps. Divergence time estimation was calibrated using the age of isolates as tip dates. The resulting tree and log files were combined using LogCombiner v1.10.4, set with a 10–25% burn-in. Effective sample sizes were verified in Tracer v1.7.1 [44], phylogenetic trees constructed, and node posterior probability values annotated with TreeAnnotator v1.10.4. Visualization and Time to Most Recent Common Ancestor (TMRCA) estimation were performed using FigTree v1.4.4. Bayesian Skyline analysis was used to estimate the demographic evolutionary history of each serotype’s whole genome coding sequence. Classification of genotypes used the most widely accepted classifications for DENV1, DENV2, DENV3, and DENV4 by Goncalvez et al. [45], Twiddy et al. [46], Lanciotti et al. [47], and Lanciotti et al. [48], respectively.

### 2.4. Maximum Likelihood Phylogenetic Analyses

Maximum likelihood (ML) phylogenetic analysis was performed on the ATGC bioinformatics platform (http://www.atgc-montpellier.fr/, accessed on 14 June 2020) using the Phylogenetic Maximum Likelihood (PhyML) utility [49]. Automatic model selection was performed by Smart Model Selection (SMS) based on the Akaike Information Criterion (AIC) and Bayesian Information Criterion (BIC) within PhyML [50]. ML phylogenetic trees were inferred with aLRT SH-like fast likelihood-based methods indicating branch support. The output from PhyML was visualized using FigTree v1.4.4. 

Resulting ML trees were also investigated for temporal structure by linear regression of root-to-tip genetic distance versus sample year for each sequence using TempEst v1.5.3 [51]. The rate of nucleotide substitution and TMRCA was estimated for both the E gene and whole genome, and further divided into subgroups, as mentioned above in Section 2.3.

### 2.5. Selection Pressure Analysis

Amino acid site-specific and branch-specific selection pressure across the DENV genome were evaluated using the HyPhy v2.5 package [52] on the Datamonkey 2.0 web application [53]. Analysis was performed on the following groups for each serotype: (1) epidemic isolates, (2) complete datasets (epidemic, sylvatic, and highly divergent sequences), (3) sylvatic isolates (only for DENV2), and (4) individual genotypes. Episodic selection was detected using the Mixed Effects Model of Evolution (MEME) algorithm and pervasive selection detected through the Single-Likelihood Ancestor Counting (SLAC); Fixed Effects Likelihood (FEL); and Fast, Unconstrained Bayesian AppRoximation (FUBAR) algorithms. Branch-specific selection pressure was detected using adaptive Branch-Site Random Effect Likelihood (aBSREL) [54,55,56,57]. Sites under selection were classified using a statistical significance of *p* < 0.1 or a posterior probability >0.9, or *p* ≤ 0.05 for aBSREL, with sites under pervasive selection that were identified by at least two methods deemed genuine findings.

## 3. Results

### 3.1. Bayesian Evolutionary Analysis and TMRCA

For each DENV serotype, Bayesian evolutionary analysis was performed using the complete coding sequences and the same sequence datasets trimmed to include just the E gene. The resulting Bayesian whole genome and E gene trees displayed similar groupings for the currently recognized genotypes but differed slightly in the estimated TMRCA, and also displayed varied clade groupings within genotypes. Maximum likelihood tree estimations for the E gene and complete coding sequences (Supplementary Figures S1–S8) produced very similar trees to Bayesian analyses, differing only slightly in some levels of branch support and clade definitions.

#### 3.1.1. DENV1

**Figure 1:**
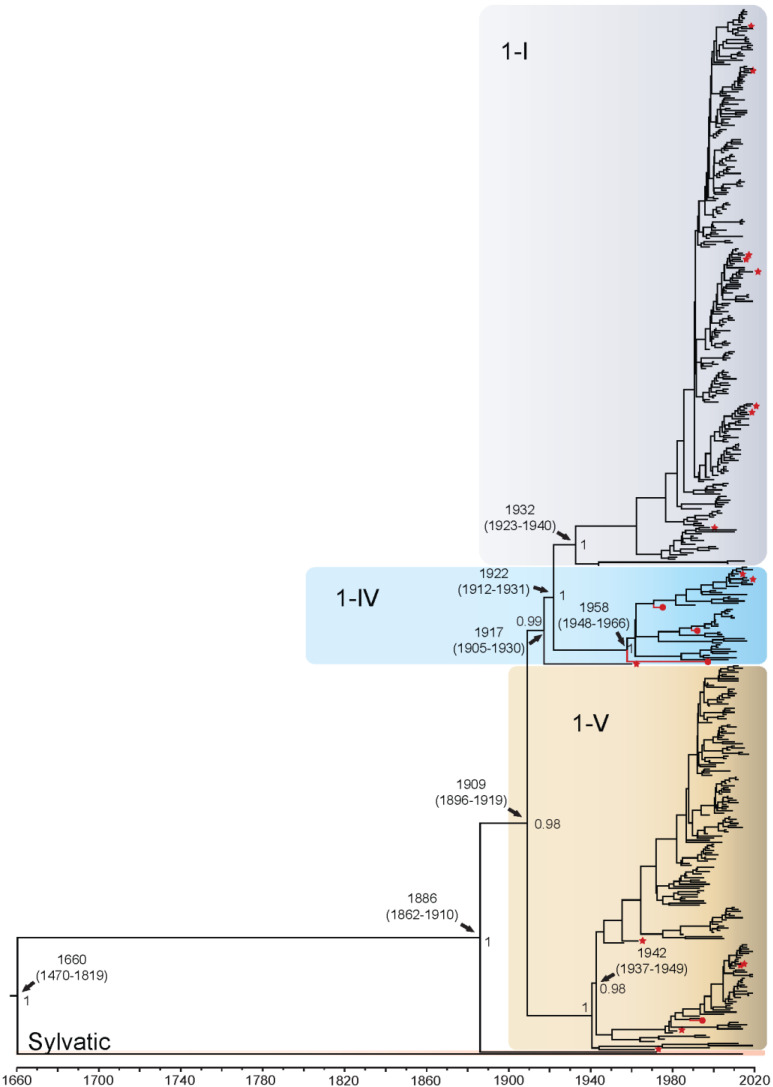
Bayesian phylogenetic tree from analysis of DENV1 whole genome complete coding sequences. Scale axis is representative of calendar years. Posterior node support is indicated for the major branches, with values <0.8 eliminated in most instances for clarity. Red circles indicate newly sequenced isolates from the QUT Arbovirus collection. Branches under positive selection (see Section 3.2.2) are indicated by red stars.

The DENV1 sequences grouped into four distinct genotypes: 1-I (*n* = 265), 1-IV (*n* = 43), 1-V (*n* = 184), and 1-III (sylvatic) (*n* = 2) (Figure 1). There were no available representative whole genome isolates for genotype 1-II. The QUT Arbovirus Collection sequences from this serotype clustered into genotypes 1-IV (*n* = 3) and 1-V (*n* = 1). The TMRCA for DENV1 whole genome sequences was estimated to be the year 1660 (95% Highest Posterior Density [HPD]: 1470–1819), when Brun2015 and the Malaysian sylvatic isolate were found to have diverged. The divergence time of the other DENV1 genotypes from the Malaysian sylvatic isolate is estimated to be 1886. Genotypes 1-I and 1-IV diverged from genotype 1-V around 1909, with genotype 1-V becoming established in two distinct groupings around 1942. The first grouping, clades 1-VA to 1-VF, diversified throughout the Americas, a process estimated to have started around the 1960s. Eleven isolates (denoted with arrows in Figure 1 and Supplementary Figures S1, S2, S9 and S10) classified in recent literature as genotype 1-III are interspersed within this 1-V cluster. The second grouping, clades 1-VG and 1-VH, diversified in the Asia-Pacific region starting in the early 1950s and more recently in the late 2010s into a few African countries. Our analysis estimated that genotype 1-I diverged from 1-IV around 1922 with each becoming established and diversifying further around the years 1932 and 1958, respectively. Genotype 1-I is composed primarily of isolates from Asia (97% of sequences). In the 1930s, genotype 1-I diverged into a clade represented by isolates from 1944, 2007, and 2015, then in the 1970s into Asia and East Africa, becoming established in Asia/Oceania in 1980–1990 forming clades 1-IA–1-ID. Genotype 1-IV diverged into several distinct clades in the 1980s, with clades 1-IVA and 1-IVD becoming established in Asian regions, and 1-IVB and 1-IVC in the Oceania region. Enlarged whole genome MCMC tree genotype groupings with associated accession number, country of isolation, and date of collection for each isolate can be found in Supplementary Figure S9. In the analyses comprising the E gene alone (Supplementary Figure S10), all divergence dates were estimated to be approximately 3–7 years earlier than found in the whole genome analysis, apart from the overall TMRCA, which was estimated to be 45 years later, at 1705 (95% HPD: 1540–1870). However, the 95% confidence intervals (CI) broadly overlap for the whole genome and E gene only analyses. All genotype groupings were well supported except the split where genotype 1-IV and 1-I diverged from genotype 1-V in 1902, which was not well supported with a posterior value of 0.14.

#### 3.1.2. DENV2

**Figure 2:**
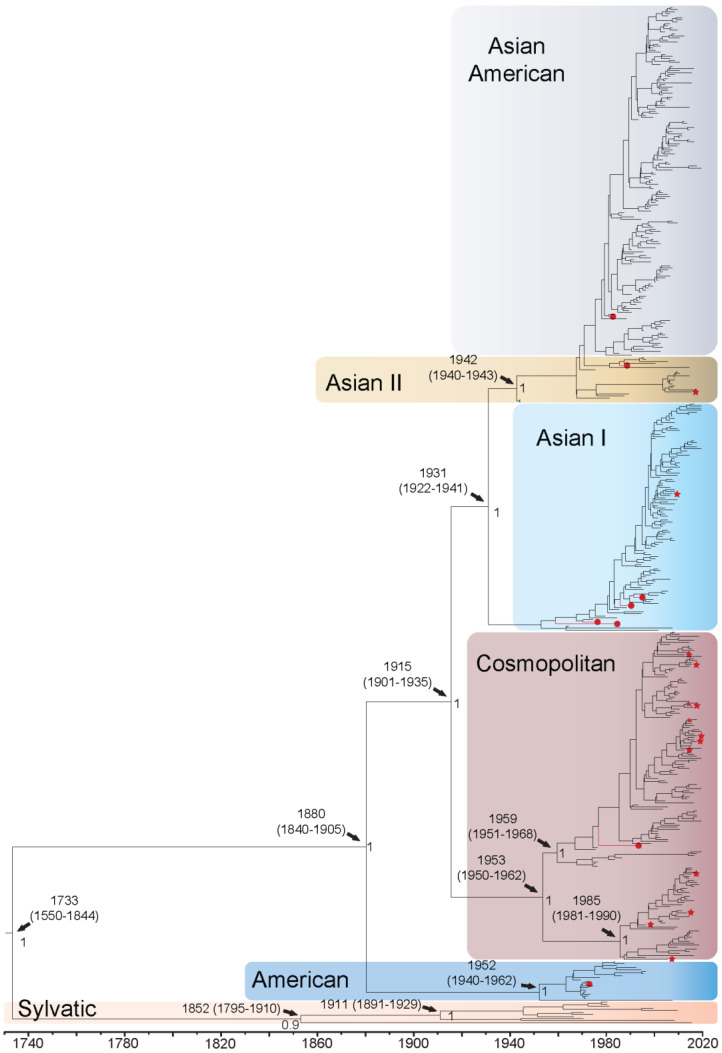
Bayesian phylogenetic tree from analysis of DENV2 whole genome complete coding sequences. Scale axis is representative of calendar years. Posterior node support is indicated for the major branches, with values <0.8 eliminated in most instances for clarity. Red circles indicate newly sequenced isolates from the QUT Arbovirus collection. Branches under positive selection (see Section 3.2.2) are indicated by red stars.

DENV2 whole genome sequences fell out into the following genotypes: Asian American (*n* = 171), Asian I (*n* = 108), Asian II (*n* = 22), Cosmopolitan (*n* = 161), American (*n* = 19), and Sylvatic (*n* = 11). The QUT Arbovirus Collection sequences fell into genotypes Asian American (*n* = 1), Asian I (*n* = 4), Asian II (*n* = 1), American (*n* = 1), and Cosmopolitan (*n* = 1). For the DENV2 whole genome sequences, the TMRCA was estimated to be 1733 (95% HPD: 1550–1844) when all epidemic genotypes diverged from the sylvatic isolates (Figure 2). The Malaysian and African sylvatic isolates are estimated to have separated from the lineage represented by the highly divergent sequence QML22 in approximately 1852, with the Malaysian and African isolates further diverging from each other around 1911. The American genotype is estimated to have diverged from the other genotypes in 1880 and later diversified into two distinct clades in the 1960s. The American A clade became established in the Americas and the American B clade in Oceania. It is estimated that the Cosmopolitan genotype diverged around 1915 and diversified into two distinct clusters, the larger spreading in Asia, Oceania, and West Africa from the 1960 onwards, and the smaller in Asia, Oceania, and East Africa in the late 1980s. The Asian I genotype diverged from Asian II and Asian American genotypes around 1931 and comprises three distinct clades, with the largest of the two becoming established in the late 1980s, primarily in Thailand, Viet Nam, and Cambodia. The Asian II and Asian American genotypes cluster together and do not form distinct genotype groupings. The isolates identified as Asian II diversified into two clades, Asian II B in the 1960s in Asia and further into the Americas, and Asian II A in the 1980s in Asia and Oceania. Further diversification into the Asian American genotype is estimated to have occurred in the mid-1970s, spreading throughout Asia and the Americas. Enlarged whole genome MCMC tree genotype groupings with associated accession number, country of isolation, and date of collection for each isolate can be found in Supplementary Figure S11. The TMRCA using the E gene sequences only was estimated to be 1568 (95% HPD: 1302–1764), 165 years earlier than estimates using whole genomes (Supplementary Figure S12). All other divergence dates were estimated to be 1–61 years earlier if using the E gene alone. However, most of these E gene-based estimates were not well supported, showing posterior values less than 0.8 for the genotype groupings between Asian I, Asian II, and Asian American, and the split between Cosmopolitan and American genotypes; however, they did form distinct groupings.

#### 3.1.3. DENV3

**Figure 3:**
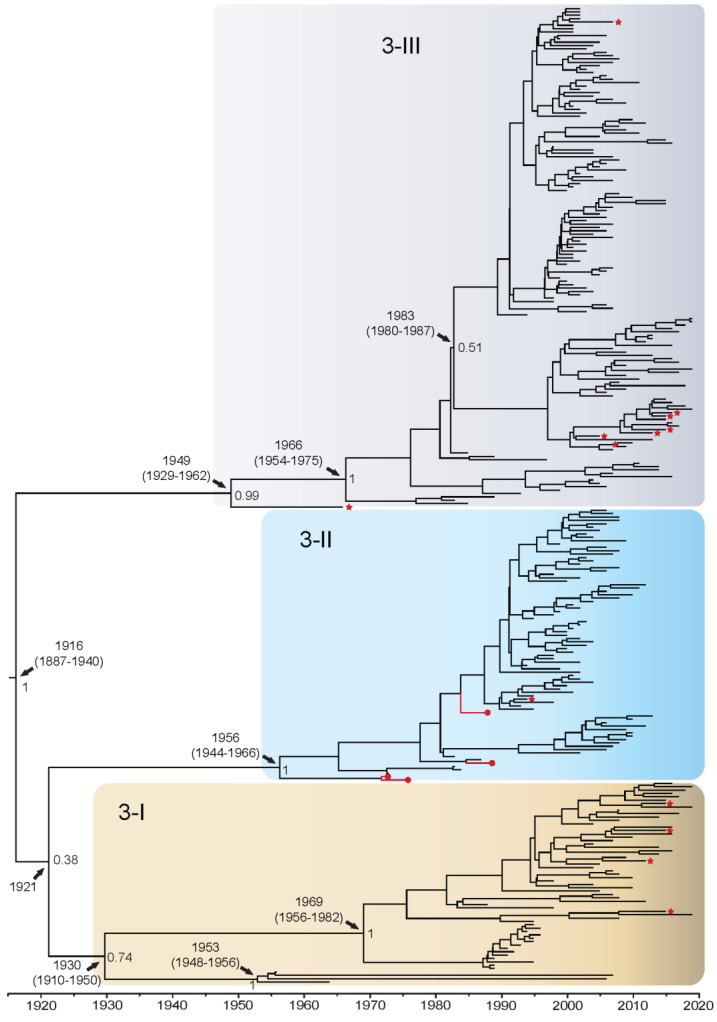
Bayesian phylogenetic tree from analysis of DENV3 whole genome complete coding sequences. Scale axis is representative of calendar years. Posterior node support is indicated for the major branches, with values <0.8 eliminated in most instances for clarity. Red circles indicate newly sequenced isolates from the QUT Arbovirus collection. Branches under positive selection (see Section 3.2.2) are indicated by red stars.

DENV3 whole genome sequences fell out into genotypes 3-I (*n* = 60), 3-II (*n* = 77), and 3-III (*n* = 149). There were no available representative whole genome isolates for genotype 3-IV, and a sylvatic DENV3 has yet to be isolated. Isolates from the QUT Arbovirus Collection (*n* = 4) fell into genotype 3-II. Using the complete coding sequence of the DENV3 genome, the TMRCA was estimated to be 1916 (95% HPD: 1887–1940) when genotype 3-III diverged from a grouping comprising genotypes 3-II and 3-I (Figure 3). These latter two genotypes then diverged from each other in 1921, however, this is poorly supported given the posterior value of 0.38. Genotype 3-I diverged into two clusters around 1930, a small cluster in the Southeast Asia and Brazil, and a larger cluster in Asia/Oceania, however, this split is weakly supported with a posterior probability of 0.74. The Asia/Oceania cluster can be further subdivided into four clades. Clade 3-ID became established in Oceania in the late 1980s, and clades 3-IA to 3-IC in Asian regions between 1980–1995. It is estimated that genotype 3-II became established in around 1956 and further diverged into two distinct clades with clade 3-IIA spreading into primarily Thailand and Cambodia beginning in the late 1980s and clade 3-IIB spreading into Southeast Asia, primarily Thailand, in approximately 1981. Genotype 3-III diverged in 1949 with isolates from India, and in 1966 further diversified in Sri Lanka and South-East Asian countries. Further diversification into two distinct clusters is observed in around 1983, however, this split is poorly supported with a posterior value of 0.51. The larger cluster forms clade 3-IIIA, becoming established in the early 1990s and further diversifying in the Americas. The smaller 3-III cluster diverged into clade 3-IIIB in Asia and Africa, and clade 3-IIIC in Asia, primarily Thailand, in approximately 1996. Enlarged whole genome MCMC tree genotype groupings with associated accession number, country of isolation, and date of collection for each isolate can be found in Supplementary Figure S13. In the analysis of the E gene alone (Supplementary Figure S14), the TMRCA was estimated to be approximately 7 years later, in 1923, but with confidence intervals that overlap (95% HPD: 1904–1941). All other genotype divergence dates were estimated to be approximately 3–7 years later when assessing the E gene alone. Additionally, the groupings for genotype 3-II and 3-III are poorly supported, yielding a posterior probability of 0.52.

#### 3.1.4. DENV4

**Figure 4:**
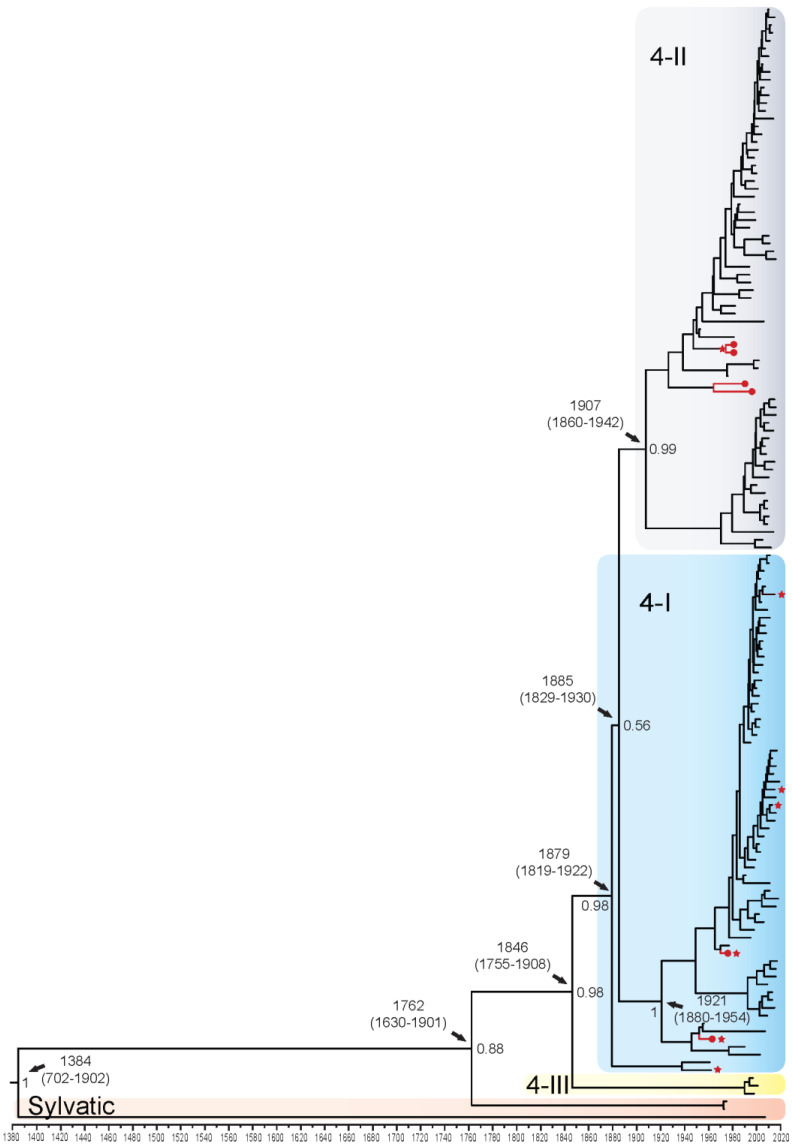
Bayesian phylogenetic tree from analysis of DENV4 whole genome complete coding sequences. Scale axis is representative of calendar years. Posterior node support is indicated for the major branches, with values <0.8 eliminated in most instances for clarity. Red circles indicate newly sequenced isolates from the QUT Arbovirus collection. Branches under positive selection (see Section 3.2.2) are indicated by red stars.

DENV4 displayed four distinct genotypes: 4-I (*n* = 65), 4-II (*n* = 66), 4-III (*n* = 3), and sylvatic (*n* = 3). The QUT Arbovirus Collection sequences fell into genotypes 4-I (*n* = 2) and 4-II (*n* = 4). Using the complete coding sequence of the DENV4 genome, the TMRCA was estimated to be 1384 (95% HPD: 702–1902), when the highly divergent DKE-121 diverged from the other Malaysian sylvatic isolates (Figure 4). It is estimated that all epidemic genotypes diverged from the Malaysian sylvatic isolates around the year 1762. Genotype 4-III diverged in approximately 1846 and is represented by three isolates from Thailand from the late 1990s and early 2000s. Genotypes 4-II and the majority of genotype 4-I diverged from each other around 1885, however, these groupings are poorly supported with a posterior probability of 0.56. Genotype 4-II is subdivided into two distinct clade groupings, 4-IIB diversifying in Asia/Oceania in 1970–2000 and 4-IIA in Asia/Oceania and Africa in the 1920s, becoming established in the Americas in the 1960s. When looking at genotype 4-I, three distinct clades emerge with 4-IA diverging around 1980 and spreading in Asia, primarily Thailand; 4-IB diverging in the early 1990s into India, China, Pakistan, and Sri Lanka; and 4-IC spreading in Asia and Puerto Rico in the 1940s. Enlarged whole genome MCMC tree genotype groupings with associated accession number, country of isolation, and date of collection for each isolate can be found in Supplementary Figure S15. The TMRCA for the E gene alone was estimated to be 1596 (95% HPD: 1138–1881), 212 years later than the whole genome estimation, with all other divergence estimates being 7–38 years later for the E gene alone (Supplementary Figure S16).

#### 3.1.5. Genotype and Clade Diversification

**Figure 5:**
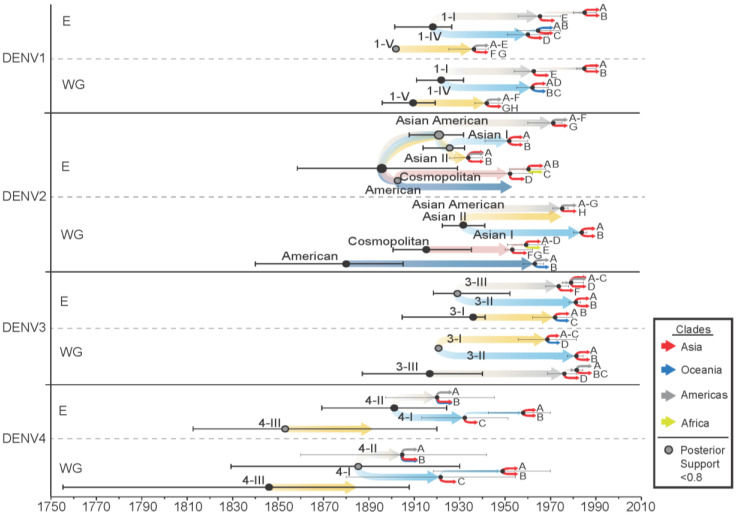
Genotype and major clade diversification date estimates and 95% HPD for the whole genome (WG) and E gene (E) for each serotype. Divergence nodes with posterior probability support < 0.8 are represented by a grey dot with a black outline. Major clades are denoted by clade letter as indicated on Supplementary Figures S9, S11, S13 and S15 and colour coded by region.

The divergence dates of genotypes within each serotype and clades within each genotype were very similar when comparing results from the whole genome and E gene (Figure 5). For all serotypes, most genotypes diverged in the late 1800s to mid-1900s, with the earliest estimated dates in the mid-to-late 1800s for DENV4 genotypes. The genotypes of DENV1, DENV2, and DENV3 all diverged in the early 1900s. For DENV1, clades began diverging in the 1940s for genotype 1-V and the 1960s and 1980s for genotypes 1-I and 1-IV. DENV2 clades diverged between 1930–1980, with analysis at the whole genome level estimating Asian I clades diverging in 1985 and 35 years earlier when looking at the E gene alone. The major clades for all genotypes of DENV3 diverged in the 1970s. For DENV4, major clades began diversifying for genotype 4-II in the early 1900s and for genotype 4-I between 1920 and 1960.

#### 3.1.6. Evolutionary Rates

**Figure 6:**
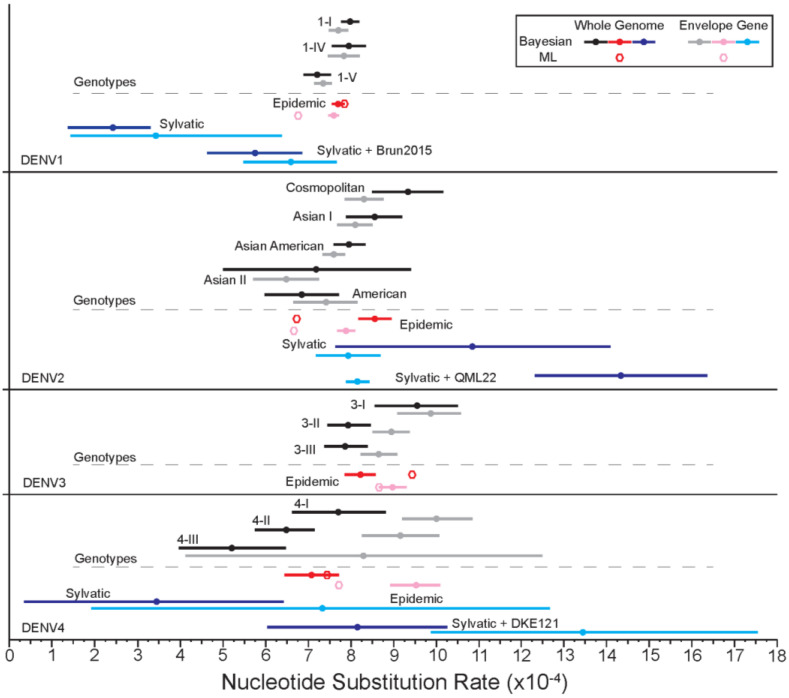
Bayesian estimates of nucleotide substitution rates (×10^−4^/site/year), increasing from left to right, along with corresponding 95% CI for the envelope (E) gene and whole genome for each serotype. ML point estimations for epidemic isolates are represented by hexagons. Estimates were calculated separately for genotype groupings (black/grey), epidemic isolates (red/pink), sylvatic isolates (dark blue/light blue), and sylvatic and highly divergent sylvatic isolates (dark blue/light blue), within each serotype.

The rate of nucleotide substitution was estimated using Bayesian methods (Figure 6). For DENV1, DENV2, and DENV3, the average nucleotide substitution rates (substitutions/site/year) of the epidemic strains for the E gene and across the whole genome were similar and fell between 7.58–8.98 × 10^−4^. The fastest average evolutionary rate for the epidemic strains was estimated for the DENV4 E gene at 9.52 × 10^−4^ (95% Confidence Interval [CI]: 8.91–10.12 × 10^−4^). Interestingly, the slowest average rate for the epidemic strains was also detected for DENV4 across the whole genome, with a rate of 7.08 × 10^−4^ (95% CI: 6.46–7.71 × 10^−4^). To investigate if there were differences in evolutionary rates of early isolates versus more recent ones, the datasets were divided into branches leading to pre-1990 (inclusive) and post-1990 viruses, for both whole genome and E gene trees. Most serotypes demonstrated very similar rates in their pre- and post-1990 groupings with broadly overlapping 95% CIs. However, for the DENV4 E gene, we found the pre-1990 isolates to be evolving at a rate of 7.52 × 10^−4^ (95% CI: 6.48–8.56 × 10^−4^) and a faster rate of 9.91 × 10^−4^ (95% CI: 9.23–10.59 × 10^−4^) for the post-1990 isolates (Supplementary Table S3).

Compared to the epidemic strains, sylvatic strains displayed varied average substitution rates across serotypes. DENV1 and DENV4 sylvatic isolates were estimated to be evolving slower than the epidemic strains, while DENV2 sylvatic strains are estimated to be evolving faster across the whole genome and at a similar rate for the E gene compared to the epidemic strains. The inclusion of the highly divergent sylvatic isolates into the sylvatic groups increased the average nucleotide substitution rate for DENV1, DENV2, and DENV4, resulting in a faster average evolutionary rate when compared to the epidemic strains for DENV2 and DENV4. However, as the sylvatic sample size is quite small, the confidence intervals for these estimations are quite large and overlapping. Maximum likelihood point estimations for epidemic strain substitution rates were relatively similar to those found by Bayesian means, with the whole genome having a slightly faster substitution rate than the E gene for all serotypes but DENV4.

At the genotype level, for DENV1, the average substitution rate was very similar for the E gene and whole genome with the fastest rate across the whole genome of genotype 1-I, 7.98 × 10^−4^ (95% CI: 7.76–8.19 × 10^−4^). For DENV2, again the E gene and whole genome for each genotype displayed relatively similar substitution rates, with the majority of the confidence intervals overlapping. The fastest average substitution rate was estimated for the Cosmopolitan genotype across the whole genome 9.4 × 10^−4^ (95% CI: 8.67–10.14 × 10^−4^) and slowest Asian II E gene, 6.49 × 10^−4^ (95% CI: 5.71–7.27 × 10^−4^). The average substitution rate for DENV3 genotypes ranged between 7.86 x 10–4 (95% CI: 7.36–8.37 × 10^−4^), for 3-III whole genome, and 9.87 × 10^−4^ (95% CI: 9.1–10.63 × 10^−4^), for the E gene of genotype 3-I. DENV4 had the greatest difference between the substitution rates of the E gene versus the whole genome, with the E gene evolving at a faster rate for all genotypes.

#### 3.1.7. Demographic Histories

**Figure 7:**
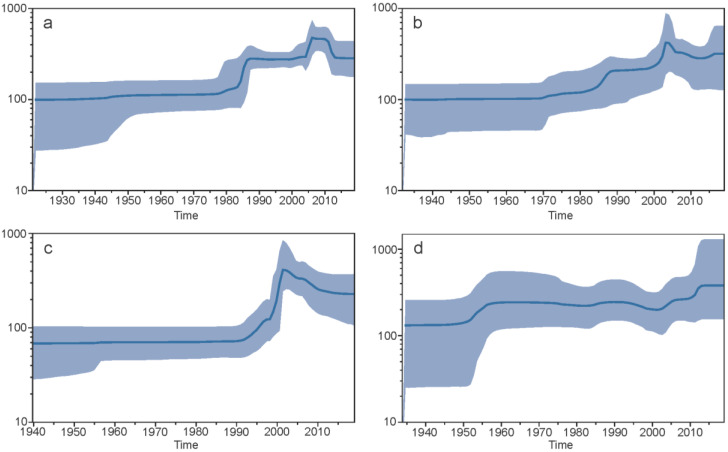
Demographic evolutionary history of whole genome epidemic isolate datasets for DENV1 (**a**), DENV2 (**b**), DENV3 (**c**), and DENV4 (**d**). The blue line represents the mean posterior value, and the shaded area represents the 95% HPD intervals. Time in years is found on the *x*-axis, and the *y*-axis is the log of the effective population size.

The demographic history of each serotype utilising epidemic datasets was constructed using a Bayesian skyline plot (Figure 7). The global population size for DENV1 remained constant until the early 1980s, increasing sharply until about 1987, followed by a constant period and a sharp increase from around 2004 to 2006, unchanged for approximately 4 years and then a decrease to the previous period of constancy. For DENV2, the global population size remained relatively stable until the 1970s, with a gradual increase and a sharp increase in the mid-1980s and early 2000s, followed by a decrease into the 2010s then a slight increase to the present. The plot of DENV3 depicts that the global population size remained constant until approximately 1992, sharply increased until 2002, followed by a slow decrease and leveling off until the present day. The DENV4 plot displayed a relatively constant population size, with an increase between 1952–1957, followed by a constant period, with a slight decline in the 2000s, followed by a gradual increase and a return to a higher but constant level at present. 

### 3.2. Selection Pressure Analysis

Site-specific (pervasive and episodic) and branch-specific selection pressure analyses were performed for the E gene and across the complete coding sequence for each serotype individually for (1) epidemic isolates, (2) complete datasets (epidemic, sylvatic, and highly divergent sequences), (3) sylvatic isolates (only for DENV2), and (4) individual genotypes. 

#### 3.2.1. Site Specific Selection

Consistent with previous reports [27], several negatively selected sites were found across the genome, but we also identified positively selected sites for each serotype and most subgroups within serotypes. When considering the whole genome sequences, pervasive selection (i.e., selection occurring across the whole phylogeny) was found for the epidemic strains (group 1) on the greatest number of sites for DENV1 (*n* = 7) and DENV2 (*n* = 7), and fewer sites on DENV3 (*n* = 1) and DENV4 (*n* = 2) (Table 2). Most positively selected sites were found on NS5 (the RdRp) for DENV1 and DENV2, on NS1 for DENV3, and on NS1 and NS5 for DENV4. For the complete datasets with the inclusion of the sylvatic isolates (group 2), fewer sites were identified for both DENV1 and DENV2, but the same sites were also identified in the epidemic group alone, with the exact same sites identified for DENV4. When the DENV2 sylvatic isolates were considered alone (group 3), no positively selected sites were identified. When considering individual genotype groups for each serotype (group 4), unique sites were identified across the genome for all serotypes. No sites under positive selection were found to be shared across serotypes or across genotypes within each serotype, with no sites found on the C and NS2B proteins for any serotype.

We found that episodic selection (i.e., heterogeneous selection on sites, across branches) was detected more frequently for all serotypes on all proteins. For the epidemic isolates (group 1), the largest number of positively selected sites across the whole genome were identified for DENV1 (*n* = 92), DENV2 (*n* = 112), and DENV3 (*n* = 95), with the least for DENV4 (*n* = 38) (Supplementary Table S4). For all serotypes, the majority of sites were identified on NS5, NS3, and the E gene. The addition of the sylvatic groups into the datasets (group 2) increased the number of identified sites on most genes by one or two compared to the epidemic group alone. For the DENV2 sylvatic group (group 3) and other individual genotypes for all serotypes (group 4), again unique sites were identified across the genome. Sites under episodic selection were identified that were shared across two genotypes within each serotype (indicated by * in Table 3) on NS5 for DENV1; prM, NS1, NS4B, and NS5 for DENV2; NS3 and NS5 for DENV3; and prM for DENV4. Sites were also found to be common across serotypes, with DENV1 sharing 16 sites, DENV2 19 sites, DENV3 21 sites, and DENV4 10 sites, and the majority being identified on the E and NS5 genes (Table 3). 

#### 3.2.2. Branch Specific Selection

Branch-specific positive selection (Supplementary Table S5) was found when evaluating the epidemic and sylvatic strains across the complete coding sequence for all serotypes (indicated by a red star on Figure 1, Figure 2, Figure 3 and Figure 4 and Supplementary Figures S1–S16). For DENV1, positive selection was identified on 16 of 927 branches. These included genotypes 1-I, 1-III, 1-IV, and 1-V, with most branches under positive selection found to belong to genotype 1-I (44%). These isolates from the 1-I genotype are from the Asian region between 2013–2019, and one isolate from Djibouti 1988. For DENV2, positive selection was found on 14 of 905 branches. These included Asian I, Asian II, and Cosmopolitan genotypes, with most branches under positive selection found to belong to the Cosmopolitan genotype (71%). These Cosmopolitan isolates are primarily from the Asian region from 1996–2017, with one isolate from New Guinea 2015. For DENV3, positive selection was found on 13 of 523 branches. These included genotypes 3-I, 3-II, and 3-III, with most branches under positive selection belonging to genotype 3-III (54%). These isolates from genotype 3-III are primarily from Asia 1966–2016 and one from Barbados 2007. Finally, for DENV4, branch-specific positive selection was found on 7 of 264 branches, all belonging to genotype 4-I. These isolates from genotype 4-I are primarily from Asia from 1962–2015 and one isolate from Puerto Rico in 1963.

## 4. Discussion

Dengue is the pre-eminent arboviral disease of humans in the world. We take advantage of the large accumulating set of whole-genome DENV sequences and provide new sequence data for several older isolates, to explore the evolutionary history and recent patterns of diversification of the virus. We constructed ML and Bayesian tree phylogenies for the complete coding sequences, as well as E genes only, for all serotypes. Isolates for all serotypes fell into currently recognized distinct genotypes, however, some genotype groupings were not well supported with low aLRT-sh like or posterior values, mainly for Bayesian estimations. The newly sequenced isolates from the QUT Arbovirus Collection clustered in known genotype groupings, enhancing our understanding of which genotypes were circulating in different regions of the Asia Pacific prior to the 1990s and during the early part of that decade. They also clustered basally within their respective clades, in the respective genotypes, consistent with their age. The incorporation of the older DENV isolates also allowed for an improved phylogenetic node calibration and a more robust estimation of evolutionary rates. For DENV1, one cluster consisted of isolates identified in the recent literature as genotype 1-III [58,59,60], but which here clustered with genotype 1-V and several isolates of unspecified genotype. However, according to the accepted genotype definitions for DENV1, genotype 1-III is reserved for sylvatic isolates [45]. Thus, we believe that this cluster is truly representative of genotype 1-V and those classified as genotype 1-III have been previously misclassified. This could be attributed to the fact that most DENV phylogenetic studies have been focused on specific regions of specific countries of the world, most often in response to an outbreak. While these are valuable analyses, they do not account for all available DENV isolates and genetic diversity, potentially leading to genotype misclassification. For DENV2, when considering Bayesian analyses using whole genomes, there was no distinct differentiation between the Asian American and Asian II genotypes, with Asian II isolates instead clustering basally to the isolates for the Asian American genotype. These two genotypes did form distinct well-supported groupings in the ML estimations, however, and distinct but unsupported groupings in the Bayesian estimation for the E gene. DENV3 demonstrated different but poorly supported genotype groupings, however, ML estimations produced very similar supported genotype groupings. In both the Bayesian and ML estimations for the DENV4 whole genome, genotype 4-I and 4-II did form distinct groupings, however, these were poorly supported by either aLRT-sh like means or posterior probabilities, and while these were supported when only considering the E gene, the other genotype groupings were not.

The estimated nucleotide substitution rate was dependent upon the method of phylogenetic analysis, ML or Bayesian. The current study found that the evolutionary rate for the epidemic strains across the E gene was similar to previous estimates [21]. The E gene might be expected to evolve faster than the rest of the genome due to its interaction with the immune system. Indeed, we found an increased rate in the E gene for DENV4 and DENV3, however, it was estimated to be slightly slower for DENV1 and DENV2, compared to the rate across the entire genome. In comparison, the ML root-to-tip estimations found the E gene to be evolving at a slower rate than the whole genome for all but DENV4 epidemic isolates. It is possible that the inclusion of the whole genome provides for a more accurate estimate or that it may include genes that evolve somewhat faster or slower than the E gene alone. The evolutionary rate for most serotypes across the E gene and whole genome appears to have remained relatively constant for DENV sequences isolated before and after 1990. Only the DENV4 E gene showed an increased evolutionary rate post-1990. The paucity of early isolates with available whole genome sequences means these findings should be verified with additional pre-1990s isolates.

The inclusion of the sylvatic strains, but excluding the highly divergent strains, into the datasets slightly increased the substitution rate for most serotypes. For DENV2, ML root-to-tip regression methods demonstrated similar results as previous research for whole genome sequences [61], with sylvatic strains evolving at a much slower rate than the epidemic strains. Further investigation into the DENV2 sylvatic sequences alone using Bayesian methods revealed that the epidemic and sylvatic strains had a very similar rate for the E gene and, counterintuitively, a faster substitution rate across the whole genome than epidemic strains. However, the small size of the DENV2 sylvatic datasets resulted in wide confidence intervals for whole genome estimates that encompassed the estimates for the E gene. Inclusion of the sylvatic strains also resulted in slower rates estimated for DENV1 and DENV4. Differences in evolutionary rates between sylvatic strains versus epidemic ones may reflect different replication dynamics, selection pressure, or a combination of the two [61]. As we highlight above, the current number of available whole genome sylvatic isolates is far too small to draw substantial conclusions. Further investigation into the sylvatic strains is necessary to discern the mechanisms driving their evolution.

We found that the E gene and the whole genome analyses led to similar estimates of TMRCA when only the epidemic strains were included. The TMRCA of the epidemic strains for the E gene was estimated to be the earliest for DENV2, followed by DENV4, DENV1, and DENV3. These findings are similar to the median found by previous studies [21], differing by 10–20 years by Bayesian means, except for DENV3. We estimated the date of emergence for the DENV3 epidemic strains to be 43 years later, in 1923 (95% HPD: 1904–1941), than the median from previously estimated studies [21]. For DENV2, the inclusion of sylvatic strains led to very different estimates based on the E gene versus whole genome. This finding again raises the hypothesis that sylvatic sequences may be under very different selective pressures than epidemic strains. However, including these sylvatic sequences in these analyses may be misleading as they may be evolving at different rates, at least for DENV2, and very few sylvatic samples are available in general for accurate reconstructions. As was anticipated, the inclusion of the highly divergent sequences into the datasets, DENV1—Brun2015, DENV2—QML-22, and DENV4—DKE-121, dramatically shifted the TMRCA for all serotypes. The inclusion of DKE-121 in the DENV4 dataset resulted in very broad 95% HPDs of about 700 years for the E gene and 1200 years for the whole genome. Further surveillance in regions such as Brunei and Malaysia is necessary to capture any potential for increasing viral diversification or zoonotic spillover events.

Similar divergence dates were estimated when comparing the whole genome and E gene for genotypes within serotypes and clades within genotypes. Most genotypes diverged in the early-to-mid 1900s with further major clade diversification estimated to have occurred between 1940–1985, concurrent with a post-war increase in global travel and commerce. The majority of clade diversification occurred in Asian and Oceanic countries, which coincides with the burden of disease in that region.

The evaluation of DENV demographic histories suggested a dramatic increase in the effective population size for DENV1, DENV2, and DENV3 in the late 1900s and early 2000s, with much less variation observed in DENV4. These changes are consistent with the spread of dengue around the world in the last few decades to new areas, particularly in the case of DENV1 and DENV2, with DENV3 and DENV4 more recently circulating [6,62]. However, it must be noted that there were very few early branches detected before the late 1900s, thus the constancy of the effective population size in earlier years may stem from a lack of statistical power to reject rate constancy. 

DENV faces selection pressures from the immune systems of both its human and mosquito hosts, resulting in novel virus variants [63]. It has also been proposed that with increasing hyperendemicity, selection pressures may be different as serotypes interact with one another [64]. Overall, we observed DENV evolution was driven primarily by negative selection. However, we did find several positively selected amino acid sites that warrant further investigation regarding their possible interaction with the host immune system. Both pervasive and episodic positive selection were identified on several proteins for all serotypes. Positive selection was greatest for the DENV1, DENV2, and DENV3 serotypes, with fewer sites detected for DENV4. These findings are consistent with the demonstrated demographic histories observed in our study, with the global population size of DENV4 displaying the least amount of change. The majority of positively selected sites was located on the E and NS5 proteins. As the E protein is involved in virus attachment and fusion with the host cell membrane, and thus more directly exposed to the host immune system, it may be undergoing positive selection pressure. The NS5 protein has methyltransferase activity and encodes the dengue RdRp. Although it is the largest and most highly conserved protein in the DENV genome [65], here we found evidence for positive selection at several sites across all serotypes. The RdRp is not present in vertebrate host cells and has thus been a recent topic of targeted drug design [66]. Our results suggest that diversification of some amino acid sites needs to be carefully evaluated for their potential impact on antiviral drugs. 

There were some positively selected sites shared across serotypes, with the majority found on the E and NS5 proteins. All but two positively selected sites were only shared between two serotypes, with positive selection at site 112 on NS4B and site 638 on NS5 shared by DENV2, DENV3, and DENV4. Branch-specific selection analyses identified that particular genotypes seem to be diversifying faster than others within serotypes, as seen in genotype 1 for DENV4 and Cosmopolitan for DENV2. Analyses identified primarily branch tips under positive selection, with very few internal nodes identified. These results could be genuine or represent mutations that have not yet had time to be purged by selection or may be the result of sequencing errors. Further study is necessary as capturing genetic diversity underpinned by selection processes is critical for vaccine design and development. 

Limitations in this study include the availability of whole genome sequences representing the global geographic distribution and representatives of endemic countries. For this study, 93% of sequences were collected after 1990, highlighting the lack of available historic whole genome DENV sequences in public databases. Archival virus collections, where available, should be interrogated for DENV isolates prior to and sequenced, allowing for more accurate phylogenetic reconstructions and time calibrations. We attempted to reduce as much sampling bias as possible by eliminating sequences that matched at ≥98% sequence identity, to obtain a random dataset from all available whole genome sequences. However, considering the large number of countries endemic for DENV, strains from Thailand predominated the available whole genome sequences in public databases. While there were representative strains from other South-East Asian countries, as well as Oceania, and the Americas, with the fewest representative isolates from Africa, this could bias the results. The more limited number of isolates for some genotypes may also have impacted the phylogenetic reconstructions. 

The results of this study contribute to the understanding of DENV evolution across the entire genome on the global scale. We found that while phylogenetic estimates for the E gene produced similar results to evaluation at the whole genome level, analysis at the single gene level does not reflect genetic diversity and processes across the rest of the genome. Several sites undergoing positive selection were also identified for all serotypes on most proteins across the whole genome, warranting further investigation in light of vaccine design, antiviral drug development, and complex patterns of antigenic evolution [64]. For example, specific sites that are rapidly diversifying may be involved in immune evasion and, potentially, future vaccine escape. It is also important to ascertain whether DENV diversity in circulating viruses is captured in the design of vaccines, to maximize their efficacy. Further global surveillance and sequencing programs, particularly in endemic regions, will provide an enhanced understanding of DENV genetic diversity and regional hotspots of diversification.

## Figures and Tables

**Table 1:** DENV isolates sourced for this study from the QUT Arbovirus Collection (formerly a WHO Arbovirus Collaborating Centre).

Accession Number	QUT Reference Number	Serotype	Country of Origin	Year Collected
OK469341	QUT 1070	DENV1	Singapore	1992
OK469342	QUT 1100	DENV1	Malaysia	1997
OK469343	QUT 1462	DENV1	Pakistan	1994
OK469344	QUT 1501	DENV1	Fiji	1975
OK469345	QUT 0605	DENV2	Myanmar	1985
OK469346	QUT 0792	DENV2	Jamaica	1981
OK469347	QUT 0868	DENV2	Palau	1988
OK469348	QUT 0870	DENV2	Myanmar	1976
OK469349	QUT 0871	DENV2	Samoa	1972
OK469350	QUT 1357	DENV2	Thailand	1990
OK469351	QUT 1453	DENV2	Myanmar	1995
OK469352	QUT 1537	DENV2	Singapore	1993
OK469353	QUT 0325	DENV3	Thailand	1988
OK469354	QUT 0327	DENV3	Thailand	1989
OK469355	QUT 0874	DENV3	Myanmar	1973
OK469356	QUT 0875	DENV3	Myanmar	1976
OK469357	QUT 0789	DENV4	Puerto Rico	1963
OK469358	QUT 0876	DENV4	Myanmar	1976
OK469359	QUT 0877	DENV4	Kiribati	1980
OK469360	QUT 0878	DENV4	Niue	1980
OK469361	QUT 1525	DENV4	Singapore	1990
OK469362	QUT 1541	DENV4	Singapore	1995

**Table 2:** Amino acid sites identified to be undergoing pervasive positive selection for DENV1, DENV2, DENV3, and DENV4 whole genome coding sequences: (1) epidemic isolates [Epi], (2) complete datasets (epidemic, sylvatic, and highly divergent) [Epi + S], (3) sylvatic isolates (DENV2) [Sylv], and (4) individual genotypes. AA = Asian American, AI = Asian I, AII = Asian II, Am = American, and C = Cosmopolitan. Number corresponds to site on specified protein. Grey is used to more clearly demarcate where the set of columns begin and end for each serotype.

	DENV1					DENV2								DENV3				DENV4			
	Epi + S	Epi	1-I	1-IV	1-V	Epi + S	Epi	AA	A I	A II	Am	C	Sylv	Epi	3-I	3-II	3-III	Epi + S	Epi	4-I	4-II
C	-	-	-	-	-	-	-	-	-	-	-	-	-	-	-	-	-	-	-	-	-
prM	-	-	-	-	-	-	-	-	-	28	-	-	-	-	-	-	-	-	-	-	-
E	-	-	-	-	-	-	226	-	-	124	-	-	-	-	-	81	132	-	-	-	-
NS1	324	324	-	-	-	-	-	164	-	-	-	-	-	93	-	-	-	290	290	-	-
NS2A	-	-	-	-	-	174	37	174	-	-	-	162		-	-	-	210	-	-	-	-
							174														
NS2B	-	-	-	-	-	-	-	-	-	-	-	-	-	-	-	-	-	-	-	-	-
NS3	-	185	-	-	-	14	14	14	-	-	-	-	-	-	-	-	-	-	-	-	-
NS4A	-	-	-	-	-	-	-	-	12	-	-	-	-	-	-	-	-	-	-	-	-
NS4B	-	-	-	207	-	-	-	-	-	-	-	148	-	-	-	-	-	-	-	-	-
NS5	285	23	626	629	-	271	271	-	-	-	-	271	-	-	-	200	-	51	51	-	-
	669	253	647			558	401														
		265	833				558														
		285																			
		669																			

**Table 3:** Shared amino acid sites identified to be undergoing episodic positive selection for DENV1, DENV2, DENV3, and DENV4 whole genome coding sequences: (1) epidemic isolates [Epi], (2) complete datasets (epidemic, sylvatic, and highly divergent) [Epi + S], (3) sylvatic isolates (DENV2) [Sylv], and (4) individual genotypes. AI = Asian I, AII = Asian II, and C = Cosmopolitan. Number corresponds to site on the specified protein. Sites shared across genotypes within each serotype are identified with *. Unlisted genotypes did not share any positively selected sites. Grey is used to more clearly demarcate where the set of columns begin and end for each serotype.

	DENV1				DENV2						DENV3				DENV4			
	Epi + S	Epi	1-I	1-V	Epi + S	Epi	A I	A II	C	Sylv	Epi	3-I	3-II	3-III	Epi + S	Epi	4-I	4-II
C	16	16		16	16	16			16									
	95				95	95			95									
prM					2	2		55 *	2		42	42			2	2	2 *	2 *
					42	42		57 *	42									
									55 *									
									57 *									
E	203	203	194		175	175	432		175		175	175		132	132			132
	432		203		216	192			194		192	192		216				203
			432		432	216					216							
NS1	14	14			264	264	232 *	174	232 *		14			14	264			
							264				174			174				
NS2A	43	43	43			37					43	43		37				
NS2B	60	60		60							127	127					60	
	127	127																
NS3	88	88	601								88	601 *	601 *					
	592	592									592							
	601	601									601							
NS4A	2	2	2												2	2		
NS4B	100	100	100		112	112		115 *	112	115 *	100	245		144	112	112		112
									144	245	112							
											144							
											245							
NS5	174	174	174	700	96	749	284 *	78 *	5		174		638	649 *	5	5	5	
	700	700	849 *	849 *	638				78 *		749		649 *	700	96	96		
					749				284 *							638		
									749									

## Data Availability

The data supporting this research is provided in the article and in the Supplementary Files.

## Supplementary Materials

The following supporting information can be downloaded at: https://www.mdpi.com/article/10.3390/v14040703/s1, Table S1: GenBank Accession Numbers; Table S2: Recombinants and Parents; Figure S1: DENV1 ML tree—Whole Genome; Figure S2: DENV1 ML tree—Envelope Gene; Figure S3: DENV2 ML tree—Whole Genome; Figure S4: DENV2 ML tree—Envelope Gene; Figure S5: DENV3 ML tree—Whole Genome; Figure S6: DENV3 ML tree—Envelope Gene; Figure S7: DENV4 ML tree—Whole Genome; Figure S8: DENV4 ML tree—Envelope Gene; Figure S9: DENV1 MCMC tree—Whole Genome Genotypes; Figure S10: DENV1 MCMC tree—Envelope Gene; Figure S11: DENV2 MCMC tree—Whole Genome Genotypes; Figure S12: DENV2 MCMC tree—Envelope Gene; Figure S13: DENV3 MCMC tree—Whole Genome Genotypes; Figure S14: DENV3 MCMC tree—Envelope Gene; Figure S15: DENV4 MCMC tree—Whole Genome Genotypes; Figure S16: DENV4 MCMC tree—Envelope Gene; Table S3: Evolutionary Rates pre- and post-1990; Table S4: Episodic Site-Specific Positive Selection; Table S5: Branch Specific Positive Selection.
