# Supplementary material for: Global Evolutionary History and Dynamics of Dengue Viruses Inferred from Whole Genome Sequences
Source: Viruses. 2022 Mar 28;14(4):703. doi: 10.3390/v14040703 (PMC9030598; doi:10.3390/v14040703)
Supplement: Supplementary file 1 [file viruses-14-00703-s001.zip › Figures_S1toS16_MCMCandMLtrees_v4.pdf]

Supplemental Figure S1: DENV1 Whole Genome ML Trees. (a) DENV1 Whole Genome ML tree, (b) Genotype 1-I, (c) Genotype 1-IV, (d) Genotype 1-V (isolates incorrectly classified as 1-III indicated by arrows) and sylvatic. aLRT SH-like branch support indicated for the major branches. Support values >0.8 have been eliminated in most instances for clarity. Red circles/font indicate newly sequenced isolates from the QUT Arbovirus collection. Branches under positive selection (see section 3.2.2) are indicated by a red star.

Figure S1a

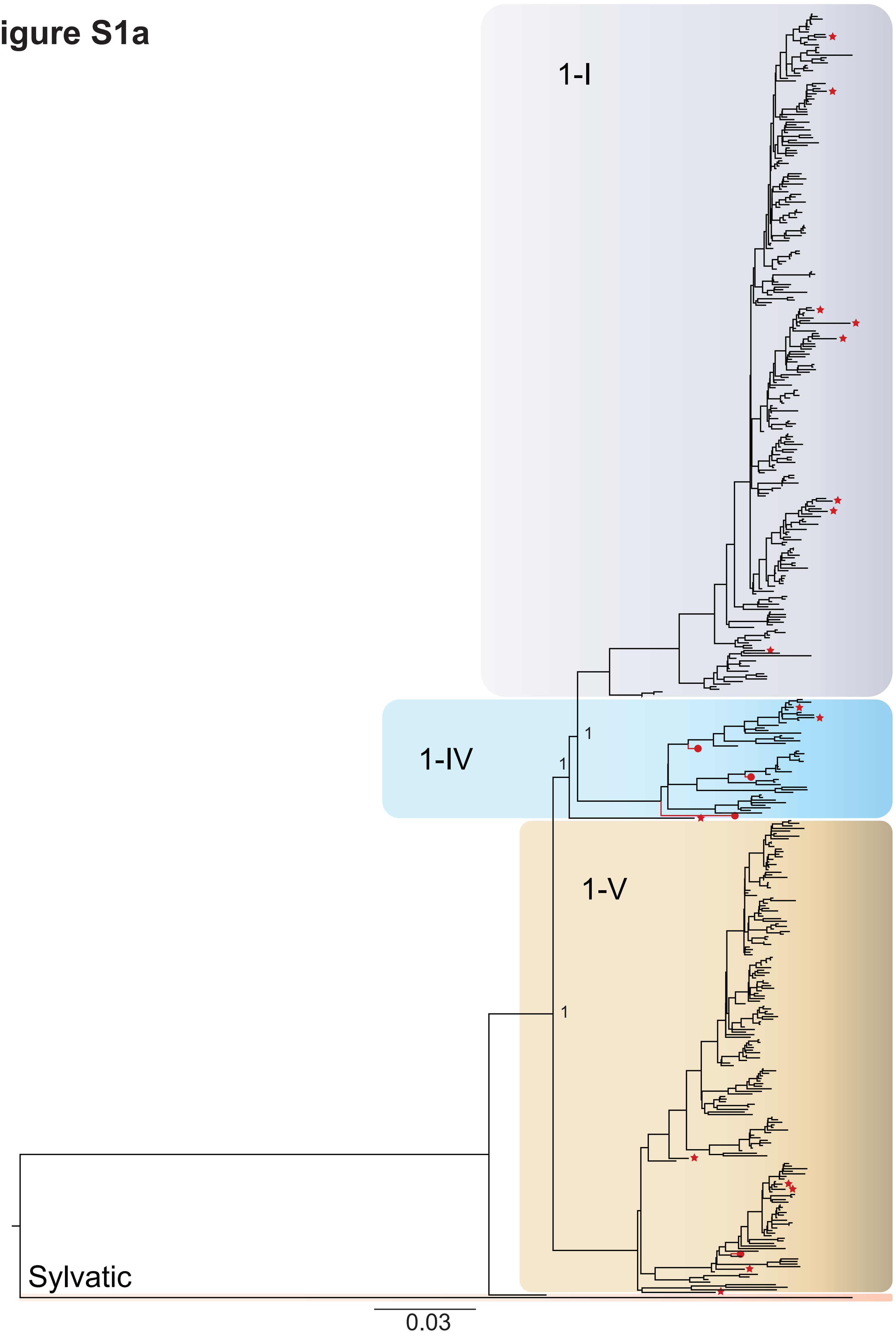

Figure S1b

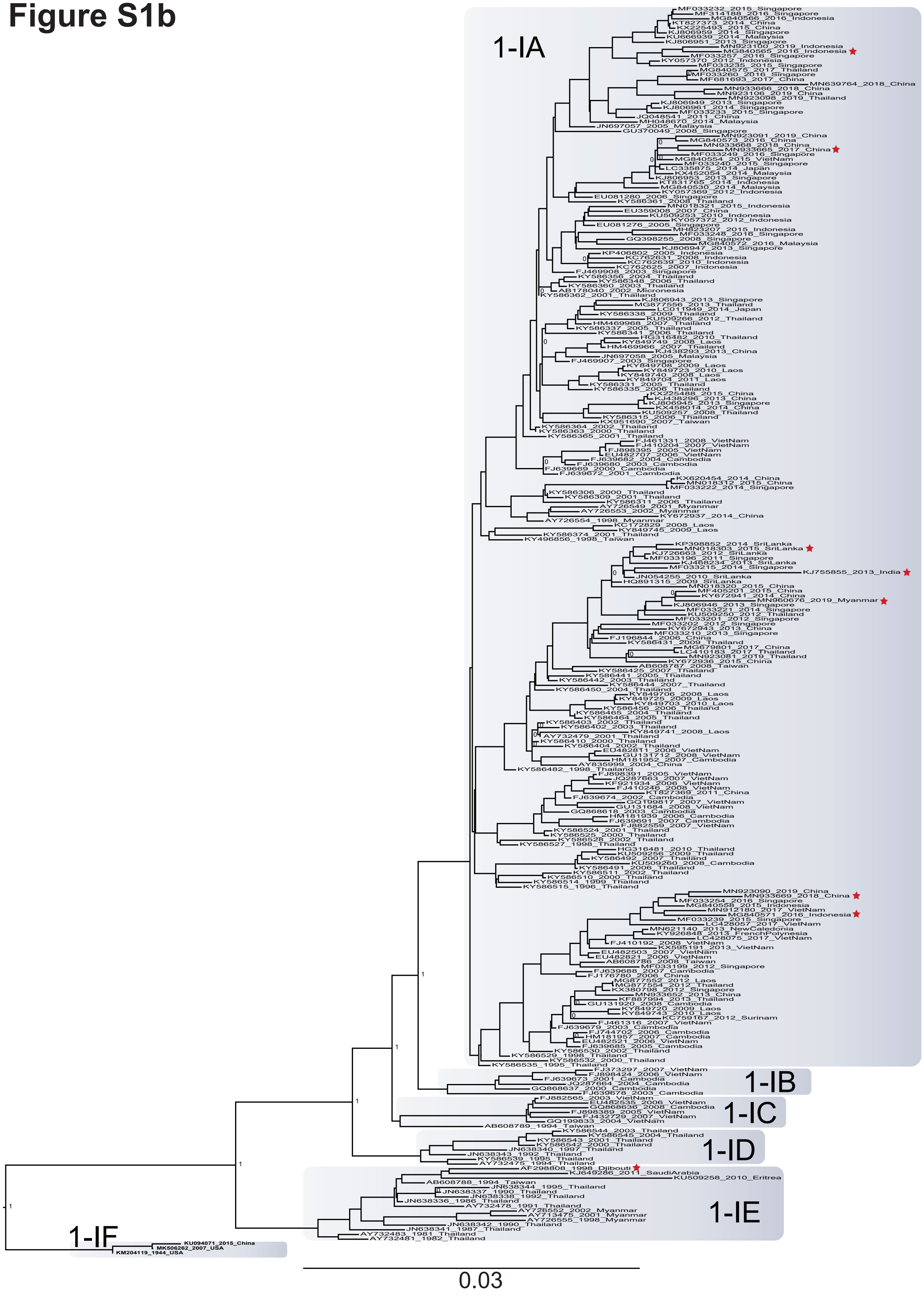

Figure S1c

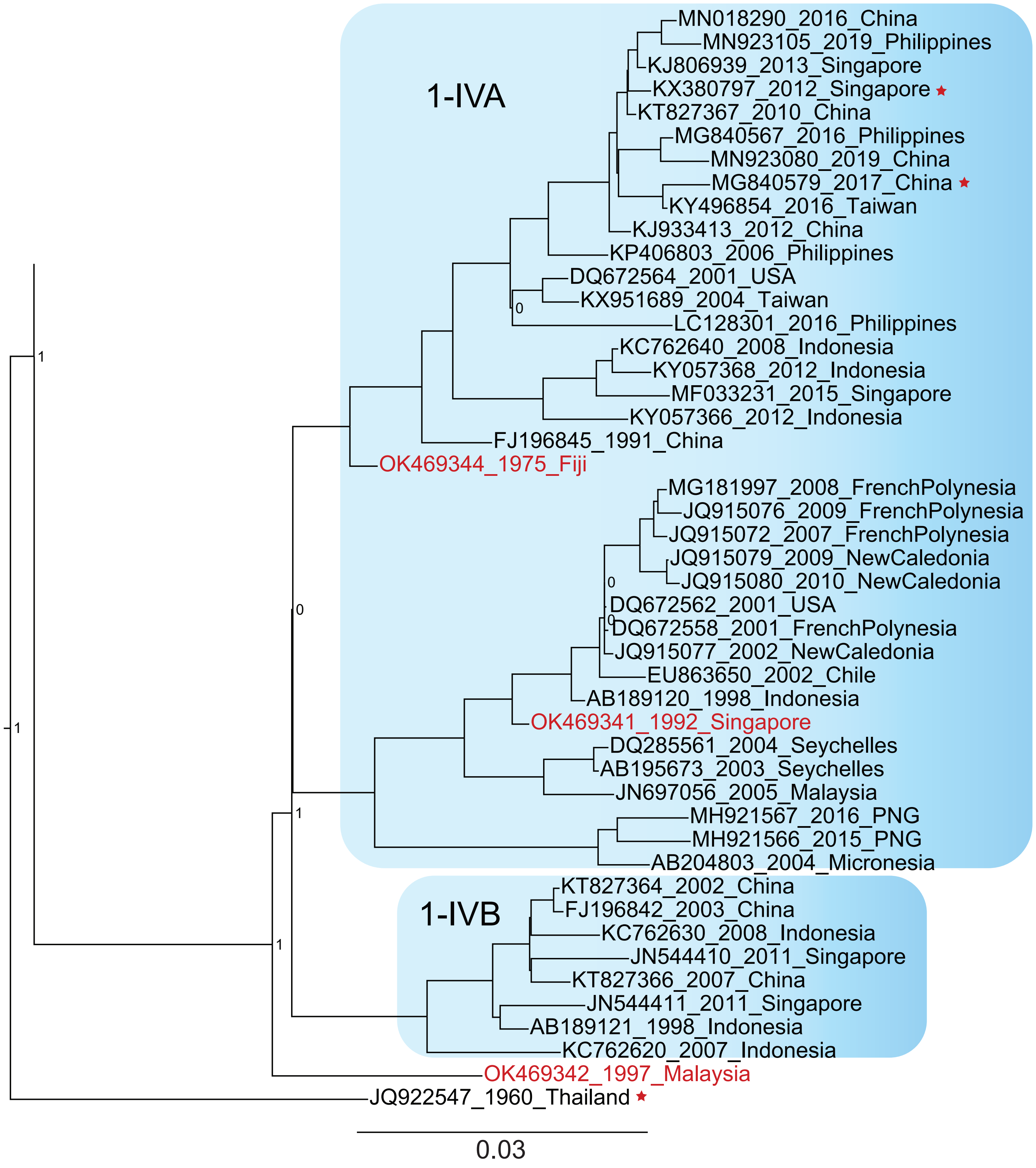
1-IVA
1-IVB
MN018290_2016_China
MN923105_2019_Philippines
KJ806939_2013_Singapore
KX380797_2012_Singapore ★
KT827367_2010_China
MG840567_2016_Philippines
MN923080_2019_China
MG840579_2017_China ★
KY496854_2016_Taiwan
KJ933413_2012_China
KP406803_2006_Philippines
DQ672564_2001_USA
KX951689_2004_Taiwan
LC128301_2016_Philippines
KC762640_2008_Indonesia
KY057368_2012_Indonesia
MF033231_2015_Singapore
KY057366_2012_Indonesia
FJ196845_1991_China
OK469344_1975_Fiji
MG181997_2008_FrenchPolynesia
JQ915076_2009_FrenchPolynesia
JQ915072_2007_FrenchPolynesia
JQ915079_2009_NewCaledonia
JQ915080_2010_NewCaledonia
DQ672562_2001_USA
DQ672558_2001_FrenchPolynesia
JQ915077_2002_NewCaledonia
EU863650_2002_Chile
AB189120_1998_Indonesia
OK469341_1992_Singapore
DQ285561_2004_Seychelles
AB195673_2003_Seychelles
JN697056_2005_Malaysia
MH921567_2016_PNG
MH921566_2015_PNG
AB204803_2004_Micronesia
KT827364_2002_China
FJ196842_2003_China
KC762630_2008_Indonesia
JN544410_2011_Singapore
KT827366_2007_China
JN544411_2011_Singapore
AB189121_1998_Indonesia
KC762620_2007_Indonesia
OK469342_1997_Malaysia
JQ922547_1960_Thailand ★
1
0
0
0
0
1
1
1
0.03

Figure S1d

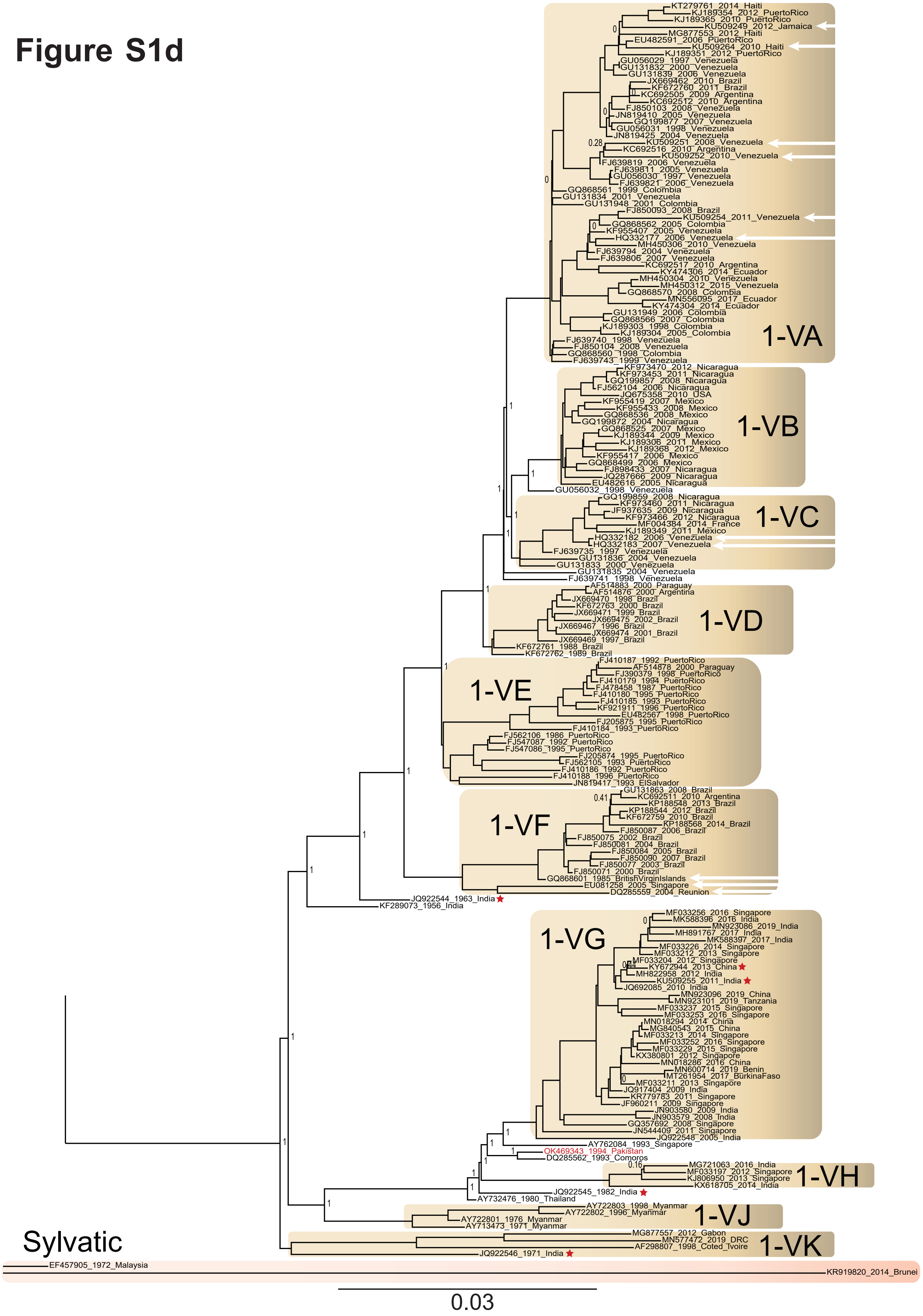
KT279761_2014_Haiti
KJ189354_2012_PuertoRico
KJ189365_2010_PuertoRico
KU509249_2012_Jamaica
MG877553_2012_Haiti
EU482591_2006_PuertoRico
KU509264_2010_Haiti
KJ189351_2012_PuertoRico
GU056029_1997_Venezuela
GU131832_2000_Venezuela
GU131839_2006_Venezuela
JX669462_2010_Brazil
KF672760_2011_Brazil
KC692505_2009_Argentina
KC692512_2010_Argentina
FJ850103_2008_Venezuela
JN819410_2005_Venezuela
GQ199877_2007_Venezuela
GU056031_1998_Venezuela
JN819425_2004_Venezuela
KU509251_2008_Venezuela
KC692516_2010_Argentina
KU509252_2010_Venezuela
FJ639819_2006_Venezuela
FJ639811_2005_Venezuela
GU056030_1997_Venezuela
FJ639821_2006_Venezuela
GQ868561_1999_Colombia
GU131834_2001_Venezuela
GU131948_2001_Colombia
FJ850093_2008_Brazil
KU509254_2011_Venezuela
GQ868562_2005_Colombia
KF955407_2005_Venezuela
HQ332177_2006_Venezuela
MH450306_2010_Venezuela
FJ639794_2004_Venezuela
FJ639806_2007_Venezuela
KC692517_2010_Argentina
KY474306_2014_Ecuador
MH450304_2010_Venezuela
MH450312_2015_Venezuela
GQ868570_2008_Colombia
MN556095_2017_Ecuador
KY474304_2014_Ecuador
GU131949_2006_Colombia
GQ868566_2007_Colombia
KJ189303_1998_Colombia
KJ189304_2005_Colombia
FJ639740_1998_Venezuela
FJ850104_2008_Venezuela
GQ868560_1998_Colombia
FJ639743_1999_Venezuela
1-VA
KF973470_2012_Nicaragua
KF973453_2011_Nicaragua
GQ199857_2008_Nicaragua
FJ562104_2006_Nicaragua
JQ675358_2010_USA
KF955419_2007_Mexico
KF955433_2008_Mexico
GQ868536_2008_Mexico
GQ199872_2004_Nicaragua
GQ868525_2007_Mexico
KJ189344_2009_Mexico
KJ189306_2011_Mexico
KJ189368_2012_Mexico
KF955417_2006_Mexico
GQ868499_2006_Mexico
FJ898433_2007_Nicaragua
JQ287666_2009_Nicaragua
EU482616_2005_Nicaragua
GU056032_1998_Venezuela
1-VB
GQ199859_2008_Nicaragua
KF973460_2011_Nicaragua
JF937635_2009_Nicaragua
KF973466_2012_Nicaragua
MF004384_2014_France
KJ189349_2011_Mexico
HQ332182_2006_Venezuela
HQ332183_2007_Venezuela
FJ639735_1997_Venezuela
GU131836_2004_Venezuela
GU131833_2000_Venezuela
GU131835_2004_Venezuela
FJ639741_1998_Venezuela
1-VC
AF514883_2000_Paraguay
AF514876_2000_Argentina
JX669470_1998_Brazil
KF672763_2000_Brazil
JX669471_1999_Brazil
JX669475_2002_Brazil
JX669467_1996_Brazil
JX669474_2001_Brazil
JX669469_1997_Brazil
KF672761_1988_Brazil
KF672762_1989_Brazil
1-VD
FJ410187_1992_PuertoRico
AF514878_2000_Paraguay
FJ390379_1998_PuertoRico
FJ410179_1994_PuertoRico
FJ478458_1987_PuertoRico
FJ410180_1995_PuertoRico
FJ410185_1993_PuertoRico
KF921911_1996_PuertoRico
EU482567_1998_PuertoRico
FJ205875_1995_PuertoRico
FJ410184_1993_PuertoRico
FJ562106_1986_PuertoRico
FJ547087_1992_PuertoRico
FJ547086_1995_PuertoRico
FJ205874_1995_PuertoRico
FJ562105_1993_PuertoRico
FJ410186_1992_PuertoRico
FJ410188_1996_PuertoRico
JN819417_1993_ElSalvador
1-VE
GU131863_2008_Brazil
KC692511_2010_Argentina
KP188548_2013_Brazil
KP188544_2012_Brazil
KF672759_2010_Brazil
KP188568_2014_Brazil
FJ850087_2006_Brazil
FJ850075_2002_Brazil
FJ850081_2004_Brazil
FJ850084_2005_Brazil
FJ850090_2007_Brazil
FJ850077_2003_Brazil
FJ850071_2000_Brazil
GQ868601_1985_BritishVirginIslands
EU081258_2005_Singapore
DQ285559_2004_Reunion
1-VF
JQ922544_1963_India
KF289073_1956_India
MF033256_2016_Singapore
MK588396_2016_India
MN923086_2019_India
MH891767_2017_India
MK588397_2017_India
MF033226_2014_Singapore
MF033212_2013_Singapore
MF033204_2012_Singapore
KY672944_2013_China
MH822958_2012_India
KU509255_2011_India
JQ692085_2010_India
MN923096_2019_China
MN923101_2019_Tanzania
MF033237_2015_Singapore
MF033253_2016_Singapore
MN018294_2014_China
MG840543_2015_China
MF033213_2014_Singapore
MF033252_2016_Singapore
MF033229_2015_Singapore
KX380801_2012_Singapore
MN018286_2016_China
MN600714_2019_Benin
MT261954_2017_BurkinaFaso
MF033211_2013_Singapore
JQ917404_2009_India
KR779783_2011_Singapore
JF960211_2009_Singapore
JN903580_2009_India
JN903579_2008_India
GQ357692_2008_Singapore
JN544409_2011_Singapore
JQ922548_2005_India
1-VG
AY762084_1993_Singapore
OK469343_1994_Pakistan
DQ285562_1993_Comoros
MG721063_2016_India
MF033197_2012_Singapore
KJ806950_2013_Singapore
KX618705_2014_India
1-VH
JQ922545_1982_India
AY732476_1980_Thailand
AY722803_1998_Myanmar
AY722802_1996_Myanmar
AY722801_1976_Myanmar
AY713473_1971_Myanmar
1-VJ
MG877557_2012_Gabon
MN577472_2019_DRC
AF298807_1998_Coted_Ivoire
JQ922546_1971_India
1-VK
Sylvatic
EF457905_1972_Malaysia
KR919820_2014_Brunei
0.03

Supplemental Figure S2: DENV1 E Gene ML Trees. (a) DENV1 E gene ML tree, (b) Genotype 1-I, (c) Genotype 1-IV, (d) Genotype 1-V (isolates incorrectly classified as 1-III indicated by arrows) and sylvatic. aLRT SH-like branch support indicated for the major branches. Support values >0.8 have been eliminated in most instances for clarity. Red circles/font indicate newly sequenced isolates from the QUT Arbovirus collection. Branches under positive selection (see section 3.2.2) are indicated by a red star.

Figure S2a

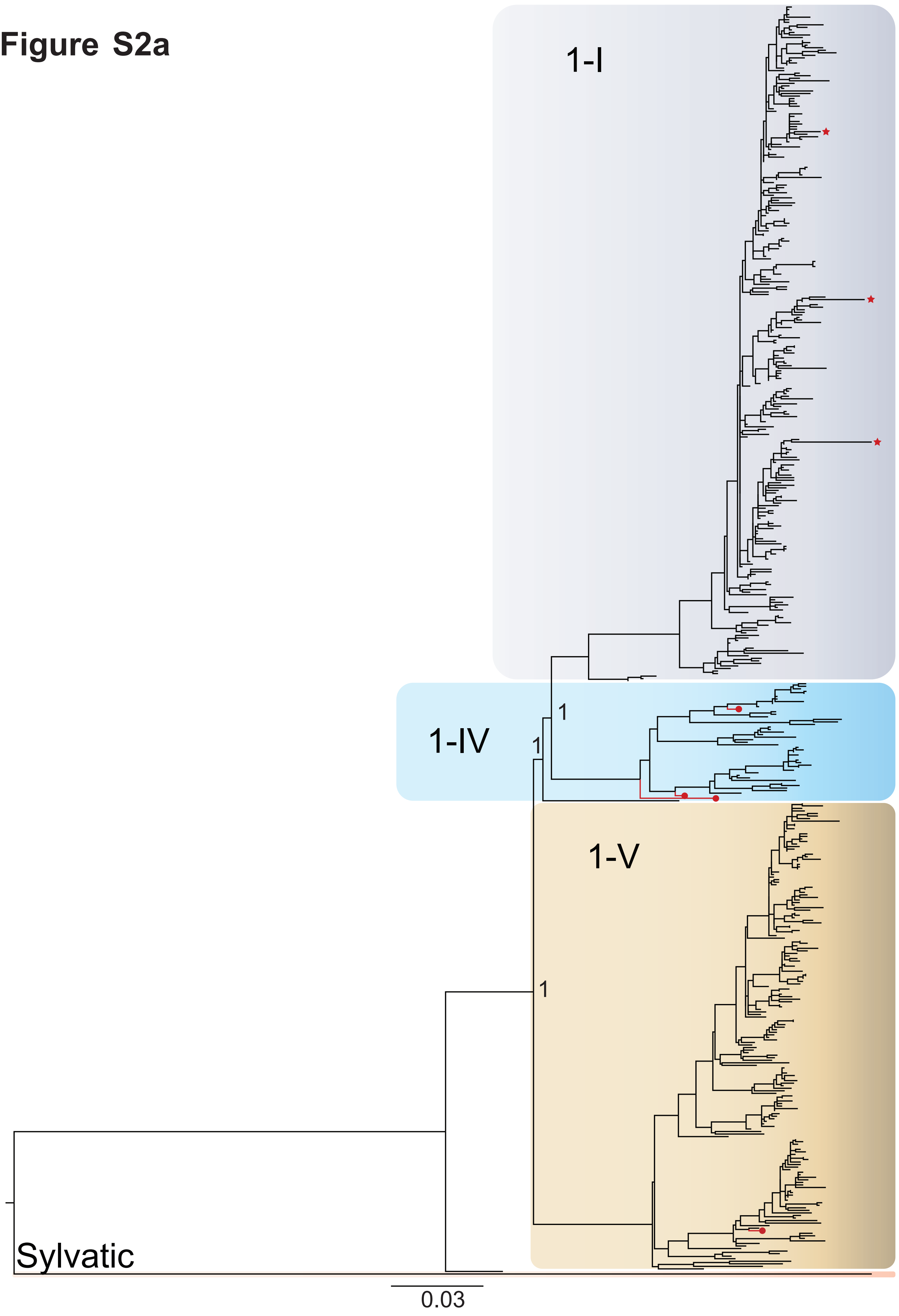

Figure S2b

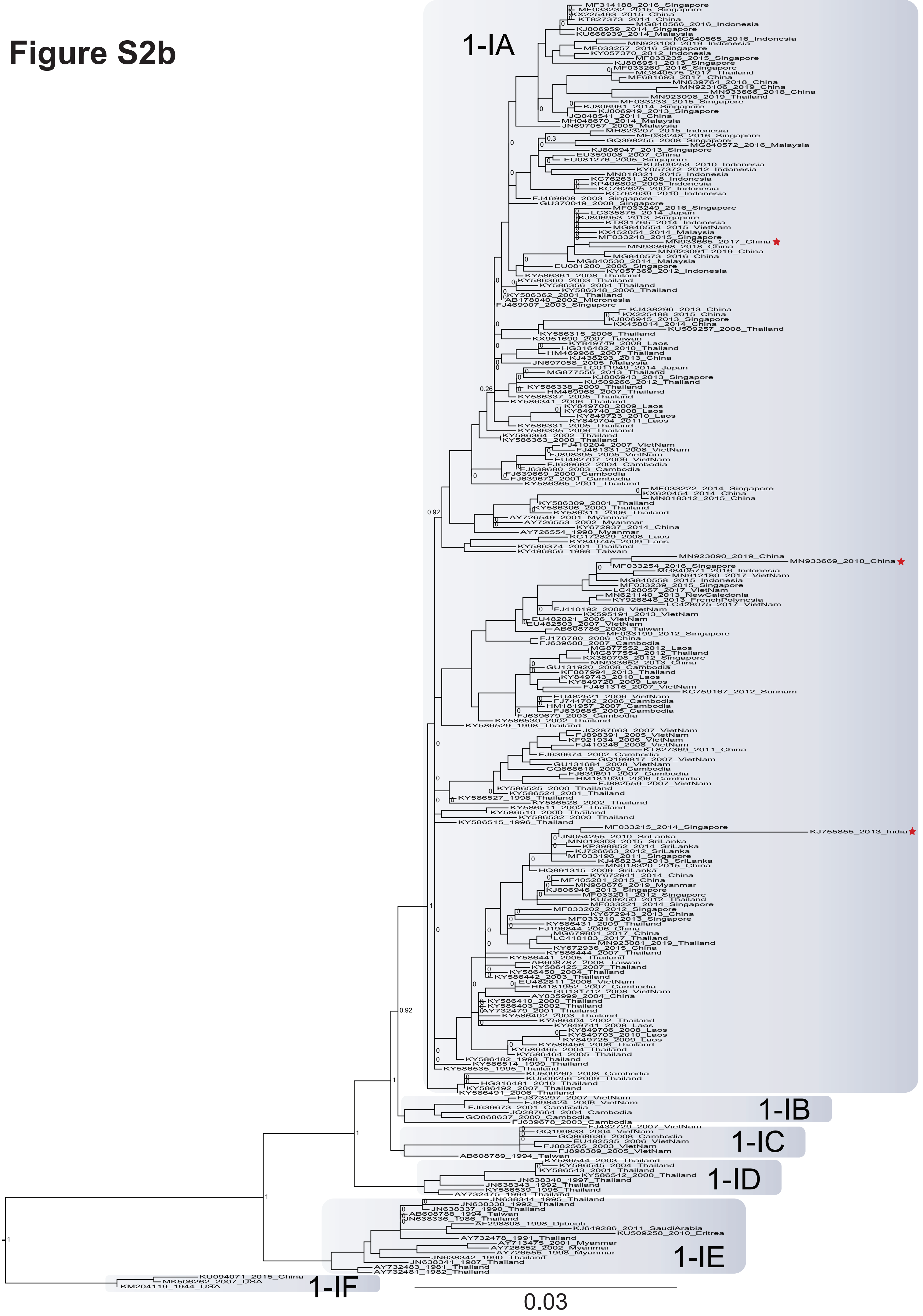

Figure S2c

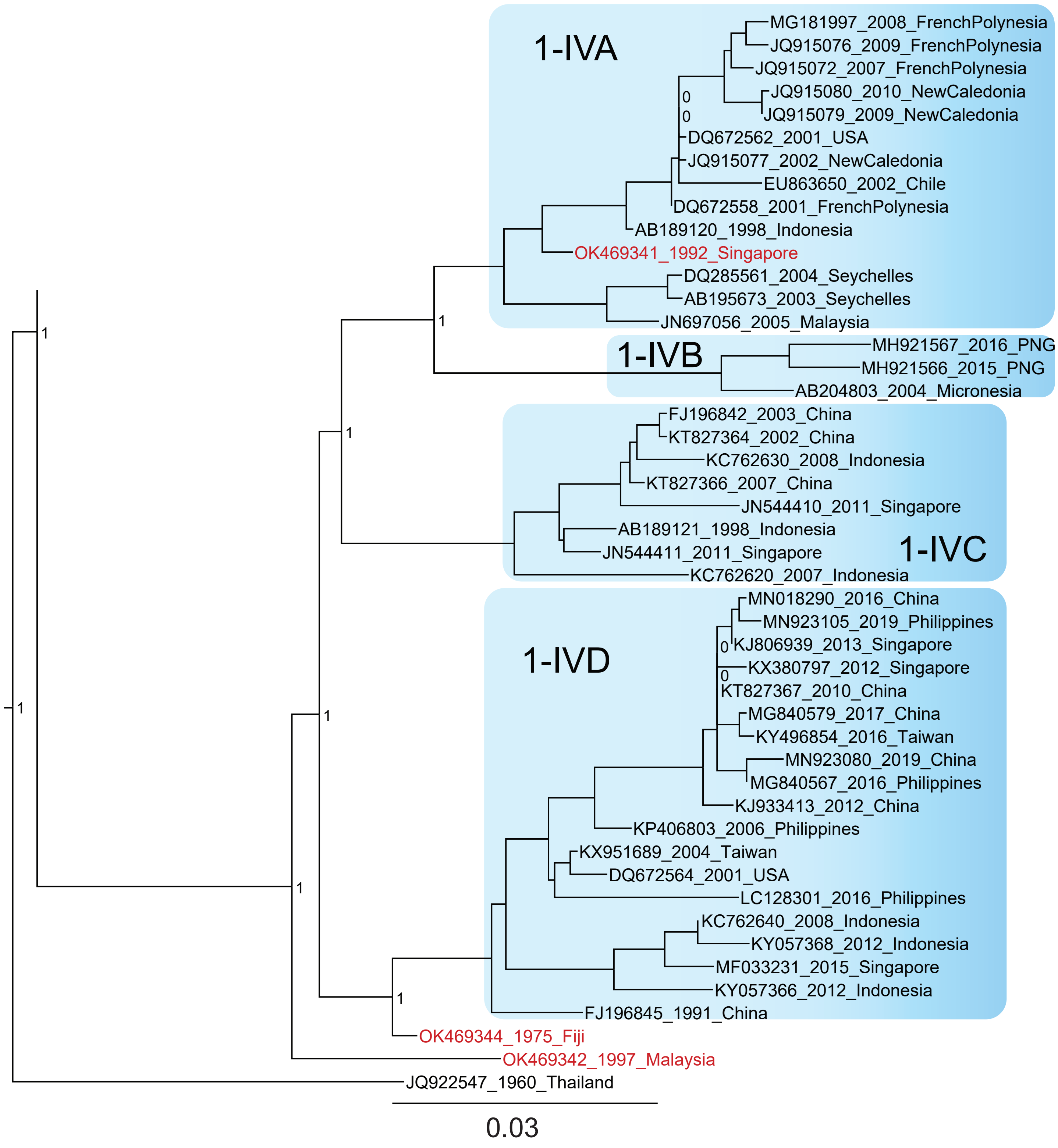
1-IVA
MG181997_2008_FrenchPolynesia
JQ915076_2009_FrenchPolynesia
JQ915072_2007_FrenchPolynesia
JQ915080_2010_NewCaledonia
JQ915079_2009_NewCaledonia
DQ672562_2001_USA
JQ915077_2002_NewCaledonia
EU863650_2002_Chile
DQ672558_2001_FrenchPolynesia
AB189120_1998_Indonesia
OK469341_1992_Singapore
DQ285561_2004_Seychelles
AB195673_2003_Seychelles
JN697056_2005_Malaysia
1-IVB
MH921567_2016_PNG
MH921566_2015_PNG
AB204803_2004_Micronesia
FJ196842_2003_China
KT827364_2002_China
KC762630_2008_Indonesia
KT827366_2007_China
JN544410_2011_Singapore
AB189121_1998_Indonesia
JN544411_2011_Singapore
1-IVC
KC762620_2007_Indonesia
MN018290_2016_China
MN923105_2019_Philippines
KJ806939_2013_Singapore
KX380797_2012_Singapore
KT827367_2010_China
1-IVD
MG840579_2017_China
KY496854_2016_Taiwan
MN923080_2019_China
MG840567_2016_Philippines
KJ933413_2012_China
KP406803_2006_Philippines
KX951689_2004_Taiwan
DQ672564_2001_USA
LC128301_2016_Philippines
KC762640_2008_Indonesia
KY057368_2012_Indonesia
MF033231_2015_Singapore
KY057366_2012_Indonesia
FJ196845_1991_China
OK469344_1975_Fiji
OK469342_1997_Malaysia
JQ922547_1960_Thailand
0.03

Figure S2d

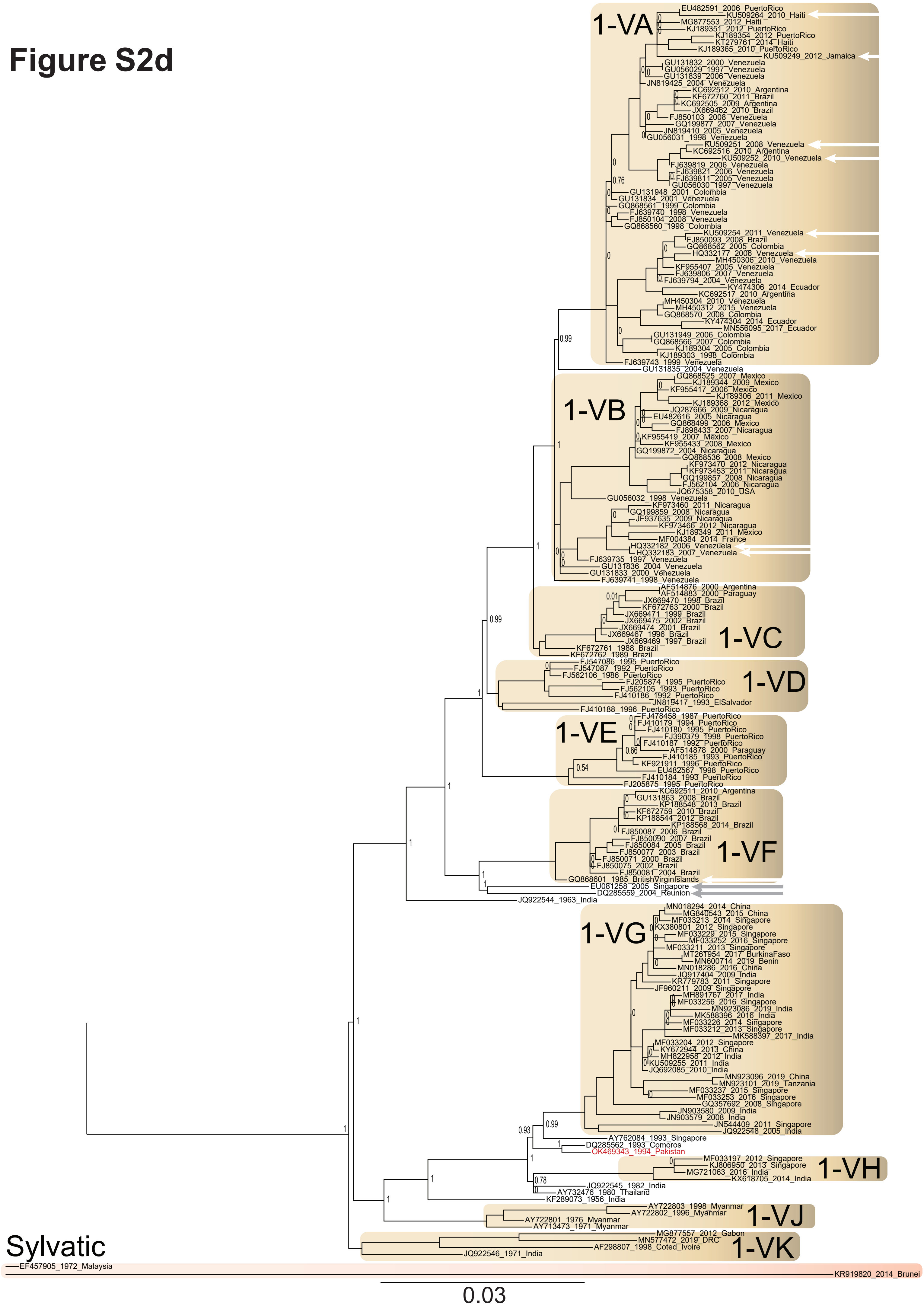

1-VA
EU482591_2006_PuertoRico
KU509264_2010_Haiti
MG877553_2012_Haiti
KJ189351_2012_PuertoRico
KJ189354_2012_PuertoRico
KT279761_2014_Haiti
KJ189365_2010_PuertoRico
KU509249_2012_Jamaica
GU131832_2000_Venezuela
GU056029_1997_Venezuela
GU131839_2006_Venezuela
JN819425_2004_Venezuela
KC692512_2010_Argentina
KF672760_2011_Brazil
KC692505_2009_Argentina
JX669462_2010_Brazil
FJ850103_2008_Venezuela
GQ199877_2007_Venezuela
JN819410_2005_Venezuela
GU056031_1998_Venezuela
KU509251_2008_Venezuela
KC692516_2010_Argentina
KU509252_2010_Venezuela
FJ639819_2006_Venezuela
FJ639821_2006_Venezuela
FJ639811_2005_Venezuela
GU056030_1997_Venezuela
GU131948_2001_Colombia
GU131834_2001_Venezuela
GQ868561_1999_Colombia
FJ639740_1998_Venezuela
FJ850104_2008_Venezuela
GQ868560_1998_Colombia
KU509254_2011_Venezuela
FJ850093_2008_Brazil
GQ868562_2005_Colombia
HQ332177_2006_Venezuela
MH450306_2010_Venezuela
KF955407_2005_Venezuela
FJ639806_2007_Venezuela
FJ639794_2004_Venezuela
KY474306_2014_Ecuador
KC692517_2010_Argentina
MH450304_2010_Venezuela
MH450312_2015_Venezuela
GQ868570_2008_Colombia
KY474304_2014_Ecuador
MN556095_2017_Ecuador
GU131949_2006_Colombia
GQ868566_2007_Colombia
KJ189304_2005_Colombia
KJ189303_1998_Colombia
FJ639743_1999_Venezuela
GU131835_2004_Venezuela
1-VB
GQ868525_2007_Mexico
KJ189344_2009_Mexico
KF955417_2006_Mexico
KJ189306_2011_Mexico
KJ189368_2012_Mexico
JQ287666_2009_Nicaragua
EU482616_2005_Nicaragua
GQ868499_2006_Mexico
FJ898433_2007_Nicaragua
KF955419_2007_Mexico
KF955433_2008_Mexico
GQ199872_2004_Nicaragua
GQ868536_2008_Mexico
KF973470_2012_Nicaragua
KF973453_2011_Nicaragua
GQ199857_2008_Nicaragua
FJ562104_2006_Nicaragua
JQ675358_2010_USA
GU056032_1998_Venezuela
KF973460_2011_Nicaragua
GQ199859_2008_Nicaragua
JF937635_2009_Nicaragua
KF973466_2012_Nicaragua
KJ189349_2011_Mexico
MF004384_2014_France
HQ332182_2006_Venezuela
HQ332183_2007_Venezuela
FJ639735_1997_Venezuela
GU131836_2004_Venezuela
GU131833_2000_Venezuela
FJ639741_1998_Venezuela
AF514876_2000_Argentina
AF514883_2000_Paraguay
JX669470_1998_Brazil
KF672763_2000_Brazil
JX669471_1999_Brazil
JX669475_2002_Brazil
JX669474_2001_Brazil
JX669467_1996_Brazil
JX669469_1997_Brazil
KF672761_1988_Brazil
KF672762_1989_Brazil
1-VC
FJ547086_1995_PuertoRico
FJ547087_1992_PuertoRico
FJ562106_1986_PuertoRico
FJ205874_1995_PuertoRico
FJ562105_1993_PuertoRico
FJ410186_1992_PuertoRico
JN819417_1993_ElSalvador
FJ410188_1996_PuertoRico
1-VD
FJ478458_1987_PuertoRico
FJ410179_1994_PuertoRico
FJ410180_1995_PuertoRico
FJ390379_1998_PuertoRico
FJ410187_1992_PuertoRico
AF514878_2000_Paraguay
FJ410185_1993_PuertoRico
KF921911_1996_PuertoRico
EU482567_1998_PuertoRico
FJ410184_1993_PuertoRico
FJ205875_1995_PuertoRico
1-VE
KC692511_2010_Argentina
GU131863_2008_Brazil
KP188548_2013_Brazil
KF672759_2010_Brazil
KP188544_2012_Brazil
KP188568_2014_Brazil
FJ850087_2006_Brazil
FJ850090_2007_Brazil
FJ850084_2005_Brazil
FJ850077_2003_Brazil
FJ850071_2000_Brazil
FJ850075_2002_Brazil
FJ850081_2004_Brazil
GQ868601_1985_BritishVirginIslands
1-VF
EU081258_2005_Singapore
DQ285559_2004_Reunion
JQ922544_1963_India
1-VG
MN018294_2014_China
MG840543_2015_China
MF033213_2014_Singapore
KX380801_2012_Singapore
MF033229_2015_Singapore
MF033252_2016_Singapore
MF033211_2013_Singapore
MT261954_2017_BurkinaFaso
MN600714_2019_Benin
MN018286_2016_China
JQ917404_2009_India
KR779783_2011_Singapore
JF960211_2009_Singapore
MH891767_2017_India
MF033256_2016_Singapore
MN923086_2019_India
MK588396_2016_India
MF033226_2014_Singapore
MF033212_2013_Singapore
MK588397_2017_India
MF033204_2012_Singapore
KY672944_2013_China
MH822958_2012_India
KU509255_2011_India
JQ692085_2010_India
MN923096_2019_China
MN923101_2019_Tanzania
MF033237_2015_Singapore
MF033253_2016_Singapore
GQ357692_2008_Singapore
JN903580_2009_India
JN903579_2008_India
JN544409_2011_Singapore
JQ922548_2005_India
AY762084_1993_Singapore
DQ285562_1993_Comoros
OK469343_1994_Pakistan
MF033197_2012_Singapore
KJ806950_2013_Singapore
MG721063_2016_India
KX618705_2014_India
1-VH
JQ922545_1982_India
AY732476_1980_Thailand
KF289073_1956_India
AY722803_1998_Myanmar
AY722802_1996_Myanmar
AY722801_1976_Myanmar
AY713473_1971_Myanmar
1-VJ
MG877557_2012_Gabon
MN577472_2019_DRC
AF298807_1998_Coted_Ivoire
JQ922546_1971_India
1-VK
Sylvatic
EF457905_1972_Malaysia
KR919820_2014_Brunei
0.99
0.76
0.01
0.66
0.54
0.93
0.78
0.03

Supplemental Figure S3: DENV2 Whole Genome ML Trees. (a) DENV2 Whole Genome ML tree, (b) Asian American genotype, (c) Asian I and Asian II genotypes, (d) Cosmopolitan genotype, (e) American and sylvatic genotypes. aLRT SH-like branch support indicated for the major branches. Support values >0.8 have been eliminated in most instances for clarity. Red circles/font indicate newly sequenced isolates from the QUT Arbovirus collection. Branches under positive selection (see section 3.2.2) are indicated by a red star.

Figure S3a

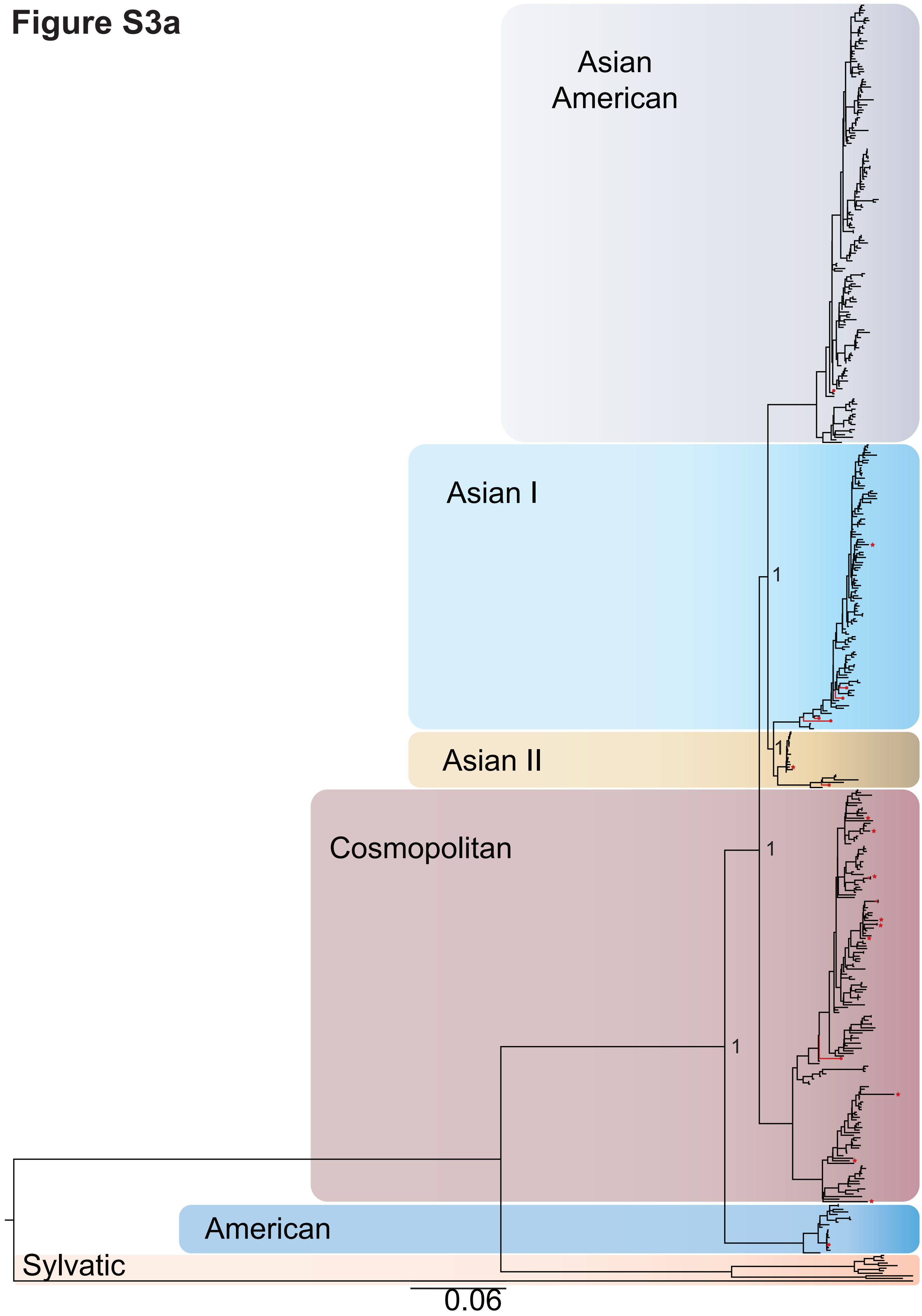

Asian American
Asian I
Asian II
Cosmopolitan
American
Sylvatic
1
1
1
1
0.06

Figure S3b

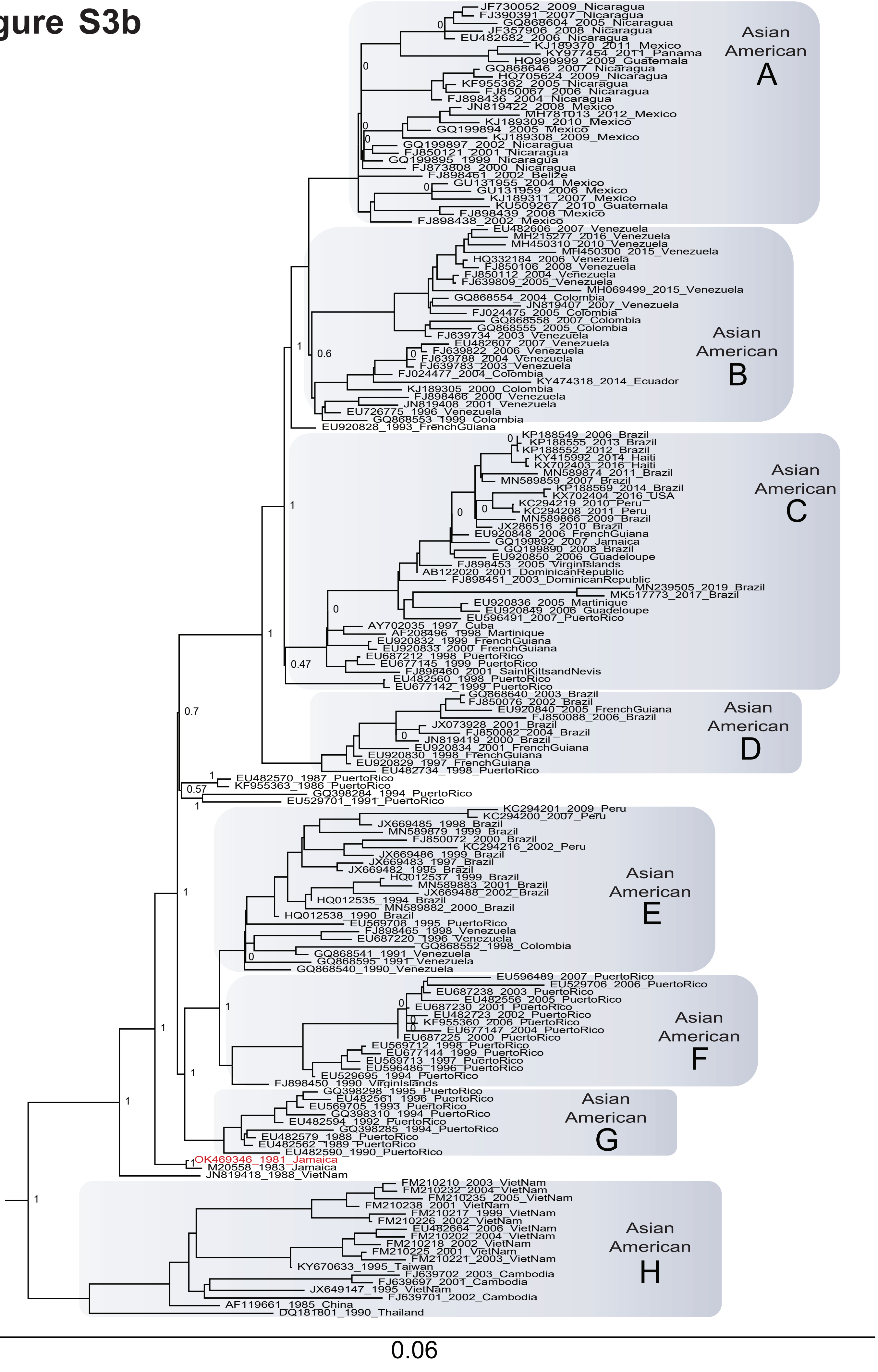
JF730052_2009_Nicaragua
FJ390391_2007_Nicaragua
GQ868604_2005_Nicaragua
JF357906_2008_Nicaragua
EU482682_2006_Nicaragua
KJ189370_2011_Mexico
KY977454_2011_Panama
HQ999999_2009_Guatemala
GQ868646_2007_Nicaragua
HQ705624_2009_Nicaragua
KF955362_2005_Nicaragua
FJ850067_2006_Nicaragua
FJ898436_2004_Nicaragua
JN819422_2008_Mexico
MH781013_2012_Mexico
KJ189309_2010_Mexico
GQ199894_2005_Mexico
KJ189308_2009_Mexico
GQ199897_2002_Nicaragua
FJ850121_2001_Nicaragua
GQ199895_1999_Nicaragua
FJ873808_2000_Nicaragua
FJ898461_2002_Belize
GU131955_2004_Mexico
GU131959_2006_Mexico
KJ189311_2007_Mexico
KU509267_2010_Guatemala
FJ898439_2008_Mexico
FJ898438_2002_Mexico
Asian American A
EU482606_2007_Venezuela
MH215277_2016_Venezuela
MH450310_2010_Venezuela
MH450300_2015_Venezuela
HQ332184_2006_Venezuela
FJ850106_2008_Venezuela
FJ850112_2004_Venezuela
FJ639809_2005_Venezuela
MH069499_2015_Venezuela
GQ868554_2004_Colombia
JN819407_2007_Venezuela
FJ024475_2005_Colombia
GQ868558_2007_Colombia
GQ868555_2005_Colombia
FJ639734_2003_Venezuela
EU482607_2007_Venezuela
FJ639822_2006_Venezuela
FJ639788_2004_Venezuela
FJ639783_2003_Venezuela
FJ024477_2004_Colombia
KY474318_2014_Ecuador
KJ189305_2000_Colombia
FJ898466_2000_Venezuela
JN819408_2001_Venezuela
EU726775_1996_Venezuela
GQ868553_1999_Colombia
EU920828_1993_FrenchGuiana
Asian American B
KP188549_2006_Brazil
KP188555_2013_Brazil
KP188552_2012_Brazil
KY415992_2014_Haiti
KX702403_2016_Haiti
MN589874_2011_Brazil
MN589859_2007_Brazil
KP188569_2014_Brazil
KX702404_2016_USA
KC294219_2010_Peru
KC294208_2011_Peru
MN589866_2009_Brazil
JX286516_2010_Brazil
EU920848_2006_FrenchGuiana
GQ199892_2007_Jamaica
GQ199890_2008_Brazil
EU920850_2006_Guadeloupe
FJ898453_2005_VirginIslands
AB122020_2001_DominicanRepublic
FJ898451_2003_DominicanRepublic
MN239505_2019_Brazil
MK517773_2017_Brazil
EU920836_2005_Martinique
EU920849_2006_Guadeloupe
EU596491_2007_PuertoRico
AY702035_1997_Cuba
AF208496_1998_Martinique
EU920832_1999_FrenchGuiana
EU920833_2000_FrenchGuiana
EU687212_1998_PuertoRico
EU677145_1999_PuertoRico
FJ898460_2001_SaintKittsandNevis
EU482560_1998_PuertoRico
EU677142_1999_PuertoRico
Asian American C
GQ868640_2003_Brazil
FJ850076_2002_Brazil
EU920840_2005_FrenchGuiana
FJ850088_2006_Brazil
JX073928_2001_Brazil
FJ850082_2004_Brazil
JN819419_2000_Brazil
EU920834_2001_FrenchGuiana
EU920830_1998_FrenchGuiana
EU920829_1997_FrenchGuiana
EU482734_1998_PuertoRico
Asian American D
EU482570_1987_PuertoRico
KF955363_1986_PuertoRico
GQ398284_1994_PuertoRico
EU529701_1991_PuertoRico
KC294201_2009_Peru
KC294200_2007_Peru
JX669485_1998_Brazil
MN589879_1999_Brazil
FJ850072_2000_Brazil
KC294216_2002_Peru
JX669486_1999_Brazil
JX669483_1997_Brazil
JX669482_1995_Brazil
HQ012537_1999_Brazil
MN589883_2001_Brazil
JX669488_2002_Brazil
HQ012535_1994_Brazil
MN589882_2000_Brazil
HQ012538_1990_Brazil
EU569708_1995_PuertoRico
FJ898465_1998_Venezuela
EU687220_1996_Venezuela
GQ868552_1998_Colombia
GQ868541_1991_Venezuela
GQ868595_1991_Venezuela
GQ868540_1990_Venezuela
Asian American E
EU596489_2007_PuertoRico
EU529706_2006_PuertoRico
EU687238_2003_PuertoRico
EU482556_2005_PuertoRico
EU687230_2001_PuertoRico
EU482723_2002_PuertoRico
KF955360_2006_PuertoRico
EU677147_2004_PuertoRico
EU687225_2000_PuertoRico
EU569712_1998_PuertoRico
EU677144_1999_PuertoRico
EU569713_1997_PuertoRico
EU596486_1996_PuertoRico
EU529695_1994_PuertoRico
FJ898450_1990_VirginIslands
Asian American F
GQ398298_1995_PuertoRico
EU482561_1996_PuertoRico
EU569705_1993_PuertoRico
GQ398310_1994_PuertoRico
EU482594_1992_PuertoRico
GQ398285_1994_PuertoRico
EU482579_1988_PuertoRico
EU482562_1989_PuertoRico
EU482590_1990_PuertoRico
Asian American G
OK469346_1981_Jamaica
M20558_1983_Jamaica
JN819418_1988_VietNam
FM210210_2003_VietNam
FM210232_2004_VietNam
FM210235_2005_VietNam
FM210238_2001_VietNam
FM210217_1999_VietNam
FM210226_2002_VietNam
EU482664_2006_VietNam
FM210202_2004_VietNam
FM210218_2002_VietNam
FM210225_2001_VietNam
FM210221_2003_VietNam
KY670633_1995_Taiwan
FJ639702_2003_Cambodia
FJ639697_2001_Cambodia
JX649147_1995_VietNam
FJ639701_2002_Cambodia
AF119661_1985_China
DQ181801_1990_Thailand
Asian American H
0.06

Figure S3c

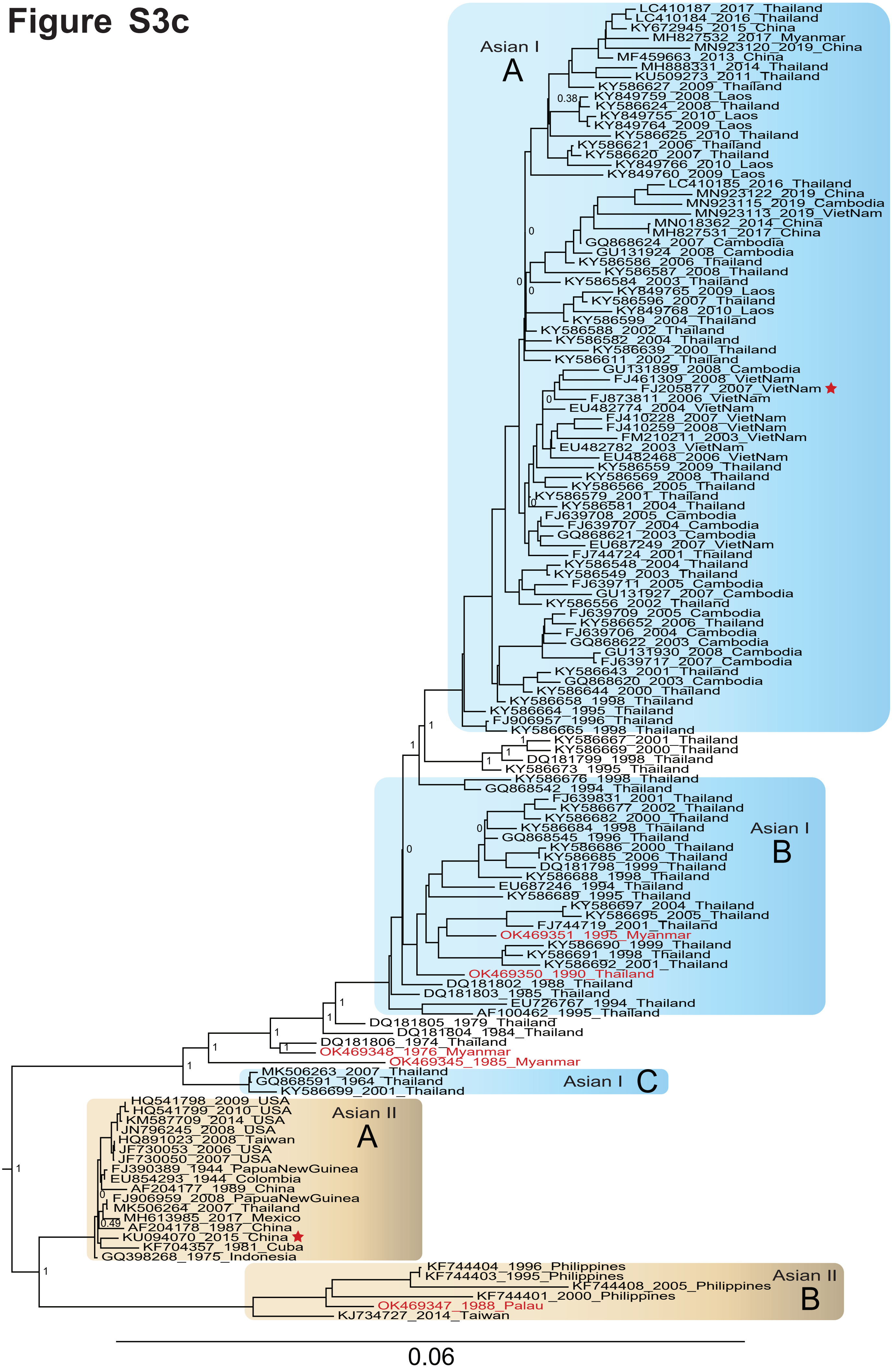
Asian I
A
LC410187_2017_Thailand
LC410184_2016_Thailand
KY672945_2015_China
MH827532_2017_Myanmar
MN923120_2019_China
MF459663_2013_China
MH888331_2014_Thailand
KU509273_2011_Thailand
KY586627_2009_Thailand
0.38
KY849759_2008_Laos
KY586624_2008_Thailand
KY849755_2010_Laos
KY849764_2009_Laos
KY586625_2010_Thailand
KY586621_2006_Thailand
KY586620_2007_Thailand
KY849766_2010_Laos
KY849760_2009_Laos
LC410185_2016_Thailand
MN923122_2019_China
MN923115_2019_Cambodia
MN923113_2019_VietNam
MN018362_2014_China
MH827531_2017_China
GQ868624_2007_Cambodia
GU131924_2008_Cambodia
KY586586_2006_Thailand
KY586587_2008_Thailand
KY586584_2003_Thailand
KY849765_2009_Laos
KY586596_2007_Thailand
KY849768_2010_Laos
KY586599_2004_Thailand
KY586588_2002_Thailand
KY586582_2004_Thailand
KY586639_2000_Thailand
KY586611_2002_Thailand
GU131899_2008_Cambodia
FJ461309_2008_VietNam
FJ205877_2007_VietNam
FJ873811_2006_VietNam
EU482774_2004_VietNam
FJ410228_2007_VietNam
FJ410259_2008_VietNam
FM210211_2003_VietNam
EU482782_2003_VietNam
EU482468_2006_VietNam
KY586559_2009_Thailand
KY586569_2008_Thailand
KY586566_2005_Thailand
KY586579_2001_Thailand
KY586581_2004_Thailand
FJ639708_2005_Cambodia
FJ639707_2004_Cambodia
GQ868621_2003_Cambodia
EU687249_2007_VietNam
FJ744724_2001_Thailand
KY586548_2004_Thailand
KY586549_2003_Thailand
FJ639711_2005_Cambodia
GU131927_2007_Cambodia
KY586556_2002_Thailand
FJ639709_2005_Cambodia
KY586652_2006_Thailand
FJ639706_2004_Cambodia
GQ868622_2003_Cambodia
GU131930_2008_Cambodia
FJ639717_2007_Cambodia
KY586643_2001_Thailand
GQ868620_2003_Cambodia
KY586644_2000_Thailand
KY586658_1998_Thailand
KY586664_1995_Thailand
FJ906957_1996_Thailand
KY586665_1998_Thailand
KY586667_2001_Thailand
KY586669_2000_Thailand
DQ181799_1998_Thailand
KY586673_1995_Thailand
KY586676_1998_Thailand
GQ868542_1994_Thailand
Asian I
B
FJ639831_2001_Thailand
KY586677_2002_Thailand
KY586682_2000_Thailand
KY586684_1998_Thailand
GQ868545_1996_Thailand
KY586686_2000_Thailand
KY586685_2006_Thailand
DQ181798_1999_Thailand
KY586688_1998_Thailand
EU687246_1994_Thailand
KY586689_1995_Thailand
KY586697_2004_Thailand
KY586695_2005_Thailand
FJ744719_2001_Thailand
OK469351_1995_Myanmar
KY586690_1999_Thailand
KY586691_1998_Thailand
KY586692_2001_Thailand
OK469350_1990_Thailand
DQ181802_1988_Thailand
DQ181803_1985_Thailand
EU726767_1994_Thailand
AF100462_1995_Thailand
DQ181805_1979_Thailand
DQ181804_1984_Thailand
DQ181806_1974_Thailand
OK469348_1976_Myanmar
OK469345_1985_Myanmar
MK506263_2007_Thailand
GQ868591_1964_Thailand
KY586699_2001_Thailand
Asian I C
Asian II
A
HQ541798_2009_USA
HQ541799_2010_USA
KM587709_2014_USA
JN796245_2008_USA
HQ891023_2008_Taiwan
JF730053_2006_USA
JF730050_2007_USA
FJ390389_1944_PapuaNewGuinea
EU854293_1944_Colombia
AF204177_1989_China
FJ906959_2008_PapuaNewGuinea
MK506264_2007_Thailand
MH613985_2017_Mexico
AF204178_1987_China
KU094070_2015_China
KF704357_1981_Cuba
GQ398268_1975_Indonesia
Asian II
B
KF744404_1996_Philippines
KF744403_1995_Philippines
KF744408_2005_Philippines
KF744401_2000_Philippines
OK469347_1988_Palau
KJ734727_2014_Taiwan
0.06

Figure S3d

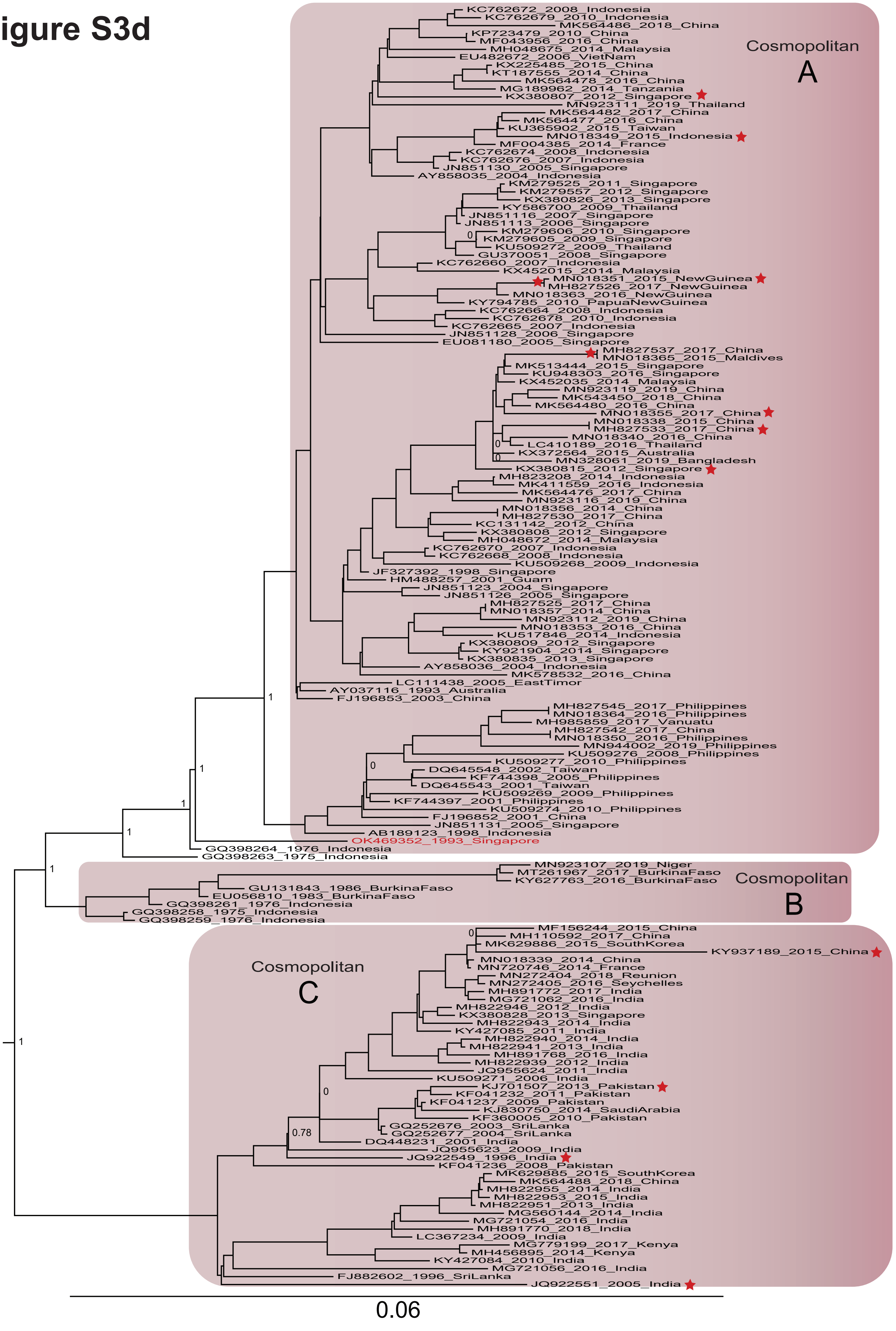

Cosmopolitan A
KC762672_2008_Indonesia
KC762679_2010_Indonesia
MK564486_2018_China
KP723479_2010_China
MF043956_2016_China
MH048675_2014_Malaysia
EU482672_2006_VietNam
KX225485_2015_China
KT187555_2014_China
MK564478_2016_China
MG189962_2014_Tanzania
KX380807_2012_Singapore
MN923111_2019_Thailand
MK564482_2017_China
MK564477_2016_China
KU365902_2015_Taiwan
MN018349_2015_Indonesia
MF004385_2014_France
KC762674_2008_Indonesia
KC762676_2007_Indonesia
JN851130_2005_Singapore
AY858035_2004_Indonesia
KM279525_2011_Singapore
KM279557_2012_Singapore
KX380826_2013_Singapore
KY586700_2009_Thailand
JN851116_2007_Singapore
JN851113_2006_Singapore
KM279606_2010_Singapore
0
KM279605_2009_Singapore
KU509272_2009_Thailand
GU370051_2008_Singapore
KC762660_2007_Indonesia
KX452015_2014_Malaysia
MN018351_2015_NewGuinea
MH827526_2017_NewGuinea
MN018363_2016_NewGuinea
KY794785_2010_PapuaNewGuinea
KC762664_2008_Indonesia
KC762678_2010_Indonesia
KC762665_2007_Indonesia
JN851128_2006_Singapore
EU081180_2005_Singapore
MH827537_2017_China
MN018365_2015_Maldives
MK513444_2015_Singapore
KU948303_2016_Singapore
KX452035_2014_Malaysia
MN923119_2019_China
MK543450_2018_China
MK564480_2016_China
MN018355_2017_China
MN018338_2015_China
MH827533_2017_China
MN018340_2016_China
0
LC410189_2016_Thailand
KX372564_2015_Australia
0
MN328061_2019_Bangladesh
KX380815_2012_Singapore
MH823208_2014_Indonesia
MK411559_2016_Indonesia
MK564476_2017_China
MN923116_2019_China
MN018356_2014_China
MH827530_2017_China
KC131142_2012_China
KX380808_2012_Singapore
MH048672_2014_Malaysia
KC762670_2007_Indonesia
KC762668_2008_Indonesia
KU509268_2009_Indonesia
JF327392_1998_Singapore
HM488257_2001_Guam
JN851123_2004_Singapore
JN851126_2005_Singapore
MH827525_2017_China
MN018357_2014_China
MN923112_2019_China
MN018353_2016_China
KU517846_2014_Indonesia
KX380809_2012_Singapore
KY921904_2014_Singapore
KX380835_2013_Singapore
AY858036_2004_Indonesia
MK578532_2016_China
LC111438_2005_EastTimor
AY037116_1993_Australia
FJ196853_2003_China
1
MH827545_2017_Philippines
MN018364_2016_Philippines
MH985859_2017_Vanuatu
MH827542_2017_China
MN018350_2016_Philippines
MN944002_2019_Philippines
KU509276_2008_Philippines
KU509277_2010_Philippines
0
DQ645548_2002_Taiwan
KF744398_2005_Philippines
DQ645543_2001_Taiwan
KU509269_2009_Philippines
KF744397_2001_Philippines
KU509274_2010_Philippines
FJ196852_2001_China
JN851131_2005_Singapore
AB189123_1998_Indonesia
1
1
OK469352_1993_Singapore
GQ398264_1976_Indonesia
1
GQ398263_1975_Indonesia
1
MN923107_2019_Niger
MT261967_2017_BurkinaFaso
KY627763_2016_BurkinaFaso
Cosmopolitan B
GU131843_1986_BurkinaFaso
EU056810_1983_BurkinaFaso
GQ398261_1976_Indonesia
GQ398258_1975_Indonesia
GQ398259_1976_Indonesia
1
Cosmopolitan C
MF156244_2015_China
0
MH110592_2017_China
MK629886_2015_SouthKorea
KY937189_2015_China
MN018339_2014_China
MN720746_2014_France
MN272404_2018_Reunion
MN272405_2016_Seychelles
MH891772_2017_India
MG721062_2016_India
MH822946_2012_India
KX380828_2013_Singapore
MH822943_2014_India
KY427085_2011_India
MH822940_2014_India
MH822941_2013_India
MH891768_2016_India
MH822939_2012_India
JQ955624_2011_India
KU509271_2006_India
0
KJ701507_2013_Pakistan
KF041232_2011_Pakistan
KF041237_2009_Pakistan
KJ830750_2014_SaudiArabia
KF360005_2010_Pakistan
GQ252676_2003_SriLanka
0.78
GQ252677_2004_SriLanka
DQ448231_2001_India
JQ955623_2009_India
JQ922549_1996_India
KF041236_2008_Pakistan
MK629885_2015_SouthKorea
MK564488_2018_China
MH822955_2014_India
MH822953_2015_India
MH822951_2013_India
MG560144_2014_India
MG721054_2016_India
MH891770_2018_India
LC367234_2009_India
MG779199_2017_Kenya
MH456895_2014_Kenya
KY427084_2010_India
MG721056_2016_India
FJ882602_1996_SriLanka
JQ922551_2005_India
0.06

Figure S3e

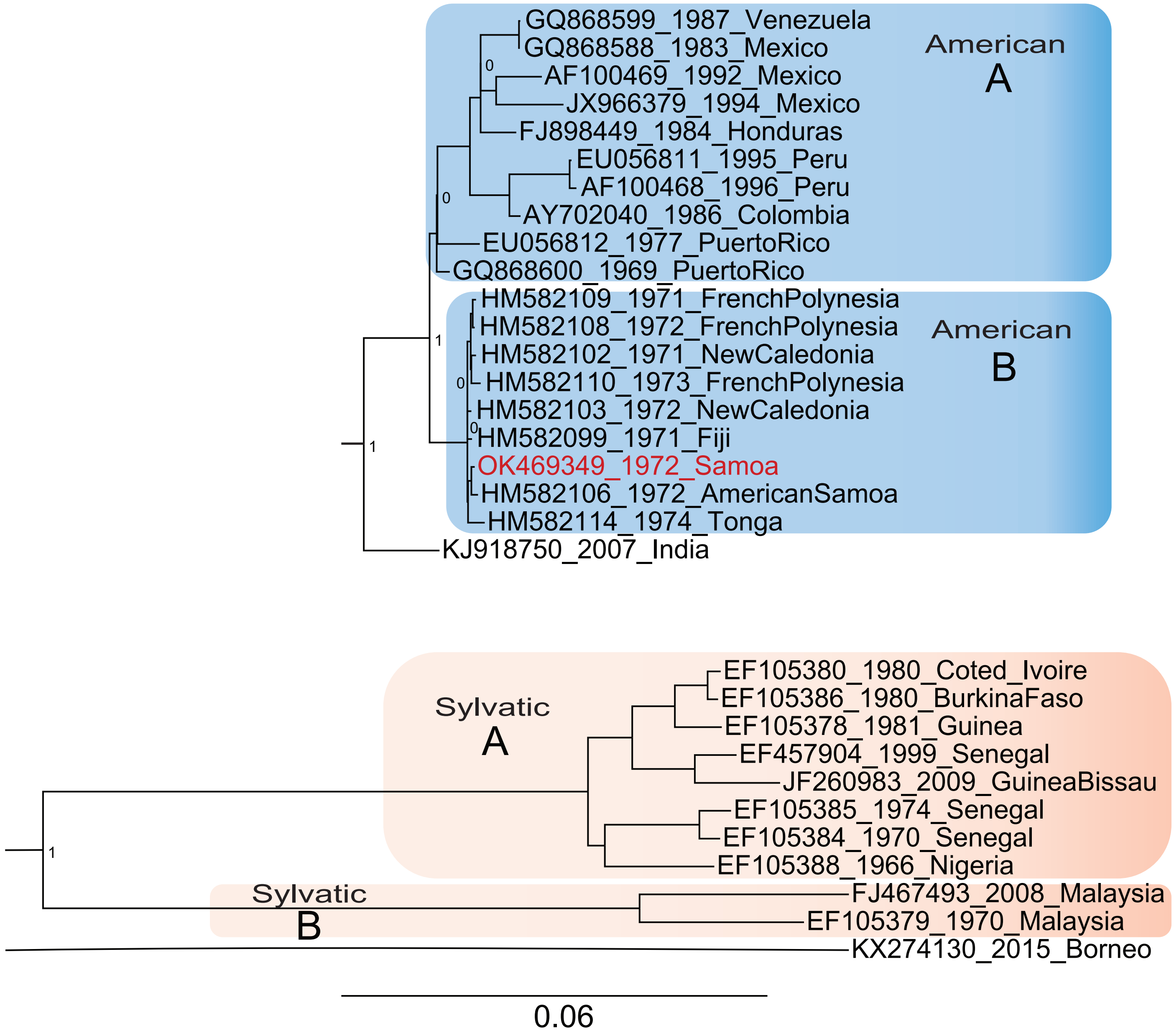

GQ868599_1987_Venezuela
GQ868588_1983_Mexico
AF100469_1992_Mexico
JX966379_1994_Mexico
FJ898449_1984_Honduras
EU056811_1995_Peru
AF100468_1996_Peru
AY702040_1986_Colombia
EU056812_1977_PuertoRico
GQ868600_1969_PuertoRico
American
A
HM582109_1971_FrenchPolynesia
HM582108_1972_FrenchPolynesia
HM582102_1971_NewCaledonia
HM582110_1973_FrenchPolynesia
HM582103_1972_NewCaledonia
HM582099_1971_Fiji
OK469349_1972_Samoa
HM582106_1972_AmericanSamoa
HM582114_1974_Tonga
American
B
KJ918750_2007_India
0
0
1
0
0
1
Sylvatic
A
EF105380_1980_Coted_Ivoire
EF105386_1980_BurkinaFaso
EF105378_1981_Guinea
EF457904_1999_Senegal
JF260983_2009_GuineaBissau
EF105385_1974_Senegal
EF105384_1970_Senegal
EF105388_1966_Nigeria
Sylvatic
B
FJ467493_2008_Malaysia
EF105379_1970_Malaysia
KX274130_2015_Borneo
1
0.06

Supplemental Figure S4: DENV2 E Gene ML Trees. (a) DENV2 E gene ML tree, (b) Cosmopolitan genotype, (c) American and sylvatic genotypes, (d) Asian I and Asian II genotypes, (e) Asian American genotype. aLRT SH-like branch support indicated for the major branches. Support values >0.8 have been eliminated in most instances for clarity. Red circles/font indicate newly sequenced isolates from the QUT Arbovirus collection. Branches under positive selection (see section 3.2.2) are indicated by a red star.

Figure S4a

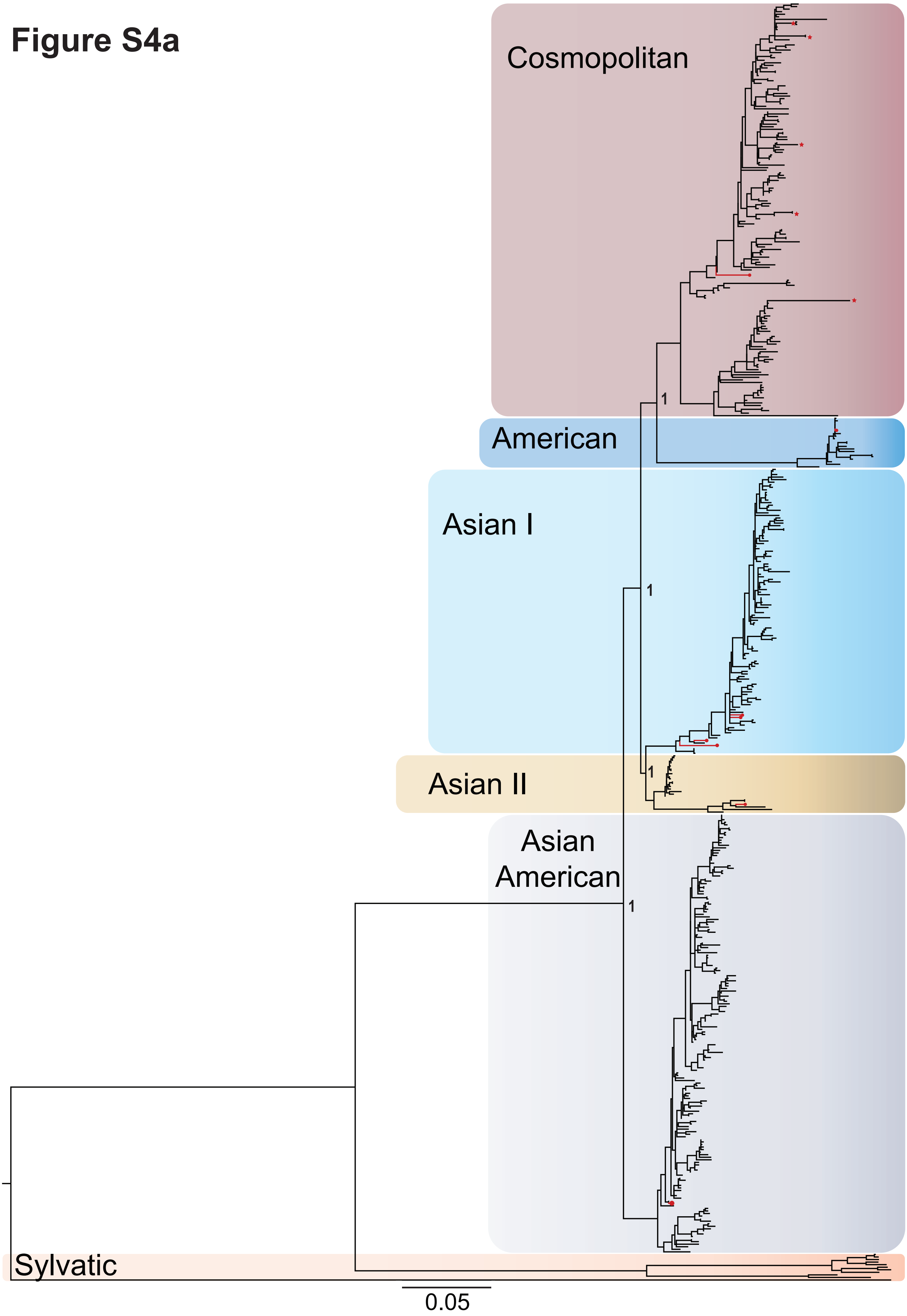
Cosmopolitan
1
American
Asian I
1
Asian II
1
Asian
American
1
Sylvatic
0.05

Figure S4b

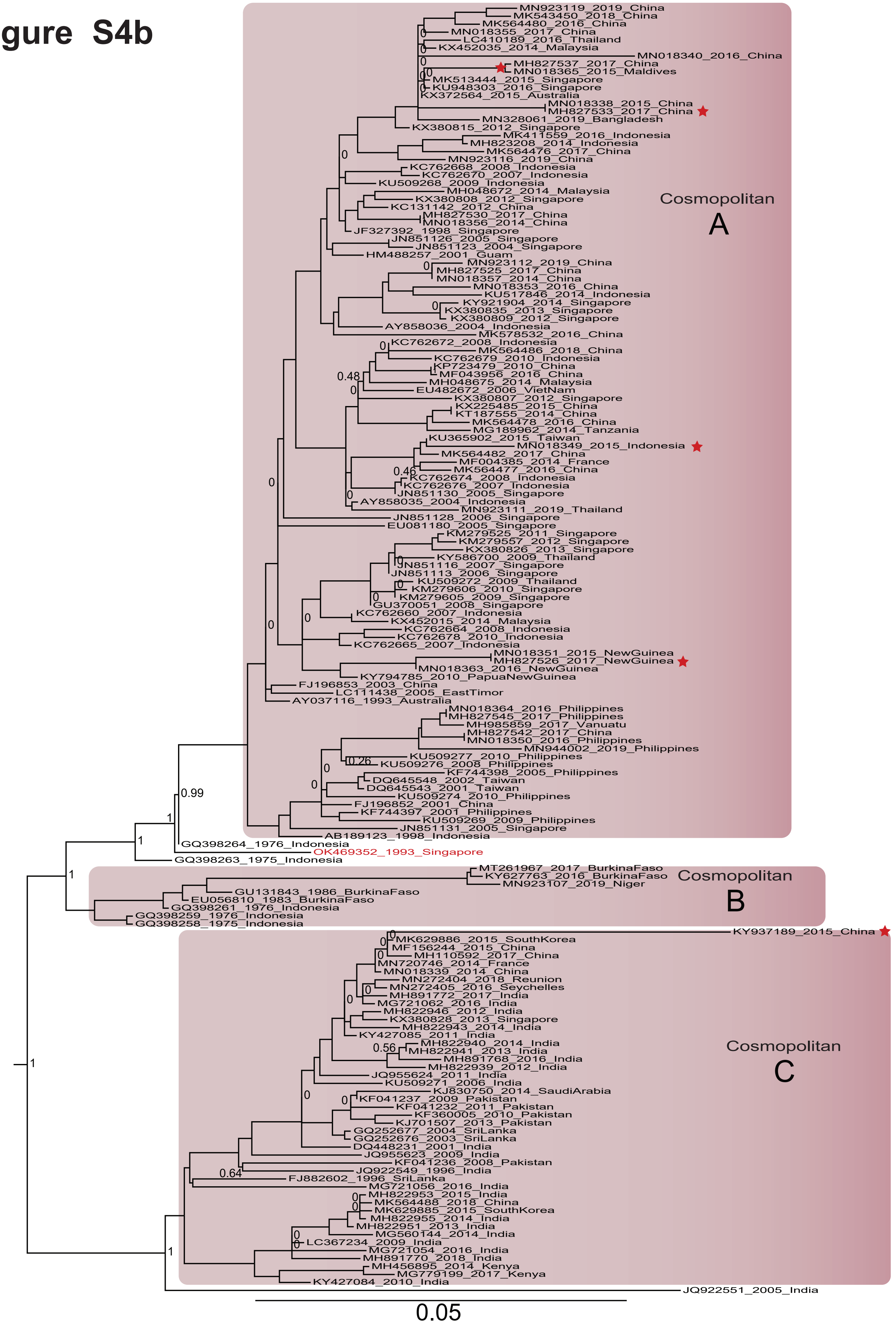

MN923119_2019_China
MK543450_2018_China
MK564480_2016_China
MN018355_2017_China
LC410189_2016_Thailand
KX452035_2014_Malaysia
MN018340_2016_China
MH827537_2017_China
MN018365_2015_Maldives
MK513444_2015_Singapore
KU948303_2016_Singapore
KX372564_2015_Australia
MN018338_2015_China
MH827533_2017_China
MN328061_2019_Bangladesh
KX380815_2012_Singapore
MK411559_2016_Indonesia
MH823208_2014_Indonesia
MK564476_2017_China
MN923116_2019_China
KC762668_2008_Indonesia
KC762670_2007_Indonesia
KU509268_2009_Indonesia
MH048672_2014_Malaysia
KX380808_2012_Singapore
KC131142_2012_China
MH827530_2017_China
MN018356_2014_China
JF327392_1998_Singapore
JN851126_2005_Singapore
JN851123_2004_Singapore
HM488257_2001_Guam
MN923112_2019_China
MH827525_2017_China
MN018357_2014_China
MN018353_2016_China
KU517846_2014_Indonesia
KY921904_2014_Singapore
KX380835_2013_Singapore
KX380809_2012_Singapore
AY858036_2004_Indonesia
MK578532_2016_China
KC762672_2008_Indonesia
MK564486_2018_China
KC762679_2010_Indonesia
KP723479_2010_China
MF043956_2016_China
MH048675_2014_Malaysia
EU482672_2006_VietNam
KX380807_2012_Singapore
KX225485_2015_China
KT187555_2014_China
MK564478_2016_China
MG189962_2014_Tanzania
KU365902_2015_Taiwan
MN018349_2015_Indonesia
MK564482_2017_China
MF004385_2014_France
MK564477_2016_China
KC762674_2008_Indonesia
KC762676_2007_Indonesia
JN851130_2005_Singapore
AY858035_2004_Indonesia
MN923111_2019_Thailand
JN851128_2006_Singapore
EU081180_2005_Singapore
KM279525_2011_Singapore
KM279557_2012_Singapore
KX380826_2013_Singapore
KY586700_2009_Thailand
JN851116_2007_Singapore
JN851113_2006_Singapore
KU509272_2009_Thailand
KM279606_2010_Singapore
KM279605_2009_Singapore
GU370051_2008_Singapore
KC762660_2007_Indonesia
KX452015_2014_Malaysia
KC762664_2008_Indonesia
KC762678_2010_Indonesia
KC762665_2007_Indonesia
MN018351_2015_NewGuinea
MH827526_2017_NewGuinea
MN018363_2016_NewGuinea
KY794785_2010_PapuaNewGuinea
FJ196853_2003_China
LC111438_2005_EastTimor
AY037116_1993_Australia
MN018364_2016_Philippines
MH827545_2017_Philippines
MH985859_2017_Vanuatu
MH827542_2017_China
MN018350_2016_Philippines
MN944002_2019_Philippines
KU509277_2010_Philippines
KU509276_2008_Philippines
KF744398_2005_Philippines
DQ645548_2002_Taiwan
DQ645543_2001_Taiwan
KU509274_2010_Philippines
FJ196852_2001_China
KF744397_2001_Philippines
KU509269_2009_Philippines
JN851131_2005_Singapore
AB189123_1998_Indonesia
GQ398264_1976_Indonesia
OK469352_1993_Singapore
GQ398263_1975_Indonesia
Cosmopolitan
A
MT261967_2017_BurkinaFaso
KY627763_2016_BurkinaFaso
MN923107_2019_Niger
GU131843_1986_BurkinaFaso
EU056810_1983_BurkinaFaso
GQ398261_1976_Indonesia
GQ398259_1976_Indonesia
GQ398258_1975_Indonesia
Cosmopolitan
B
KY937189_2015_China
MK629886_2015_SouthKorea
MF156244_2015_China
MH110592_2017_China
MN720746_2014_France
MN018339_2014_China
MN272404_2018_Reunion
MN272405_2016_Seychelles
MH891772_2017_India
MG721062_2016_India
MH822946_2012_India
KX380828_2013_Singapore
MH822943_2014_India
KY427085_2011_India
MH822940_2014_India
MH822941_2013_India
MH891768_2016_India
MH822939_2012_India
JQ955624_2011_India
KU509271_2006_India
KJ830750_2014_SaudiArabia
KF041237_2009_Pakistan
KF041232_2011_Pakistan
KF360005_2010_Pakistan
KJ701507_2013_Pakistan
GQ252677_2004_SriLanka
GQ252676_2003_SriLanka
DQ448231_2001_India
JQ955623_2009_India
KF041236_2008_Pakistan
JQ922549_1996_India
FJ882602_1996_SriLanka
MG721056_2016_India
MH822953_2015_India
MK564488_2018_China
MK629885_2015_SouthKorea
MH822955_2014_India
MH822951_2013_India
MG560144_2014_India
LC367234_2009_India
MG721054_2016_India
MH891770_2018_India
MH456895_2014_Kenya
MG779199_2017_Kenya
KY427084_2010_India
JQ922551_2005_India
Cosmopolitan
C
0.05

Figure S4c

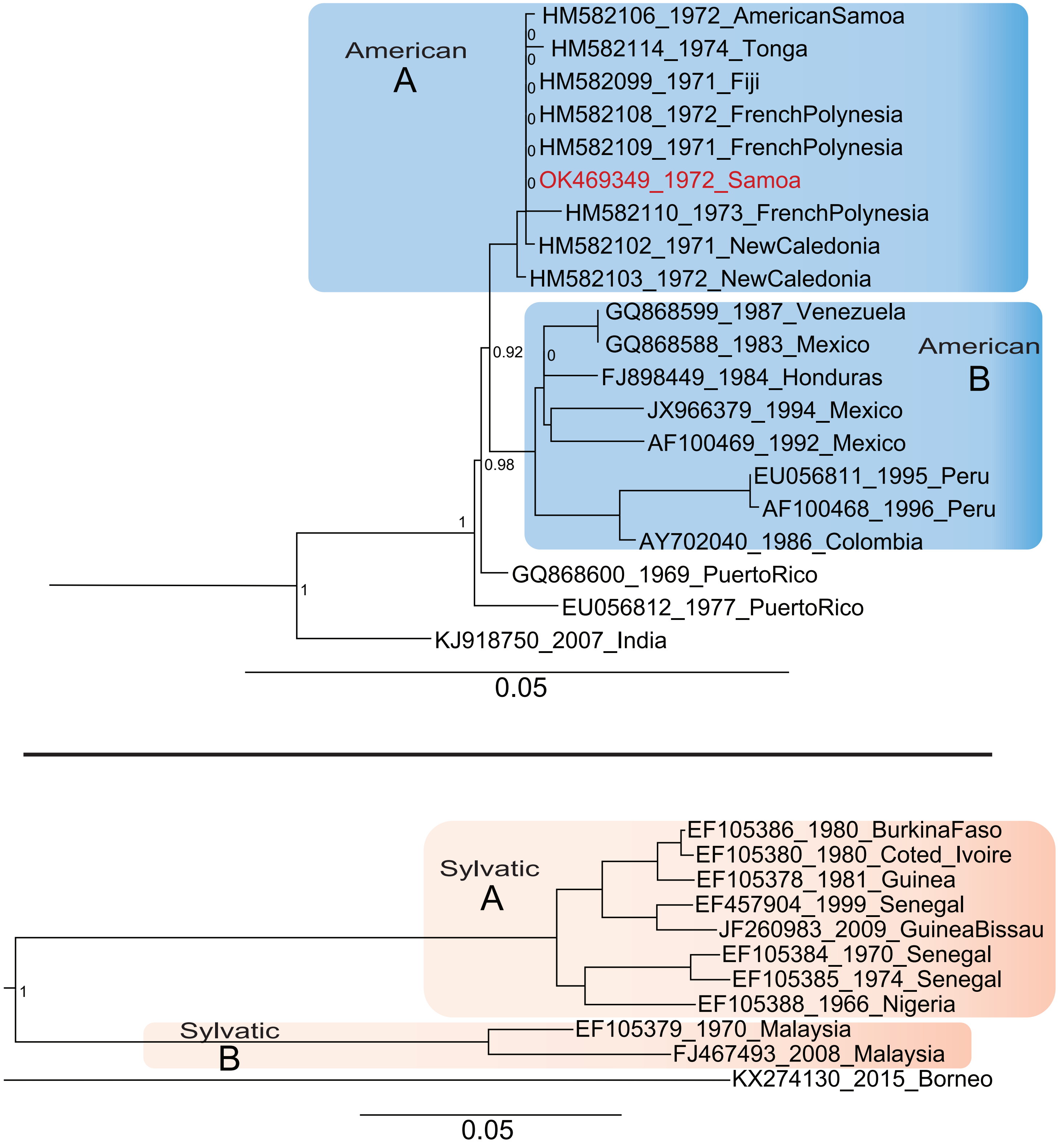

American
A
HM582106_1972_AmericanSamoa
HM582114_1974_Tonga
HM582099_1971_Fiji
HM582108_1972_FrenchPolynesia
HM582109_1971_FrenchPolynesia
OK469349_1972_Samoa
HM582110_1973_FrenchPolynesia
HM582102_1971_NewCaledonia
HM582103_1972_NewCaledonia
GQ868599_1987_Venezuela
GQ868588_1983_Mexico
American
B
FJ898449_1984_Honduras
JX966379_1994_Mexico
AF100469_1992_Mexico
EU056811_1995_Peru
AF100468_1996_Peru
AY702040_1986_Colombia
GQ868600_1969_PuertoRico
EU056812_1977_PuertoRico
KJ918750_2007_India
0.92
0.98
1
1
0
0.05
Sylvatic
A
EF105386_1980_BurkinaFaso
EF105380_1980_Coted_Ivoire
EF105378_1981_Guinea
EF457904_1999_Senegal
JF260983_2009_GuineaBissau
EF105384_1970_Senegal
EF105385_1974_Senegal
EF105388_1966_Nigeria
Sylvatic
B
EF105379_1970_Malaysia
FJ467493_2008_Malaysia
KX274130_2015_Borneo
1
0.05

Figure S4d

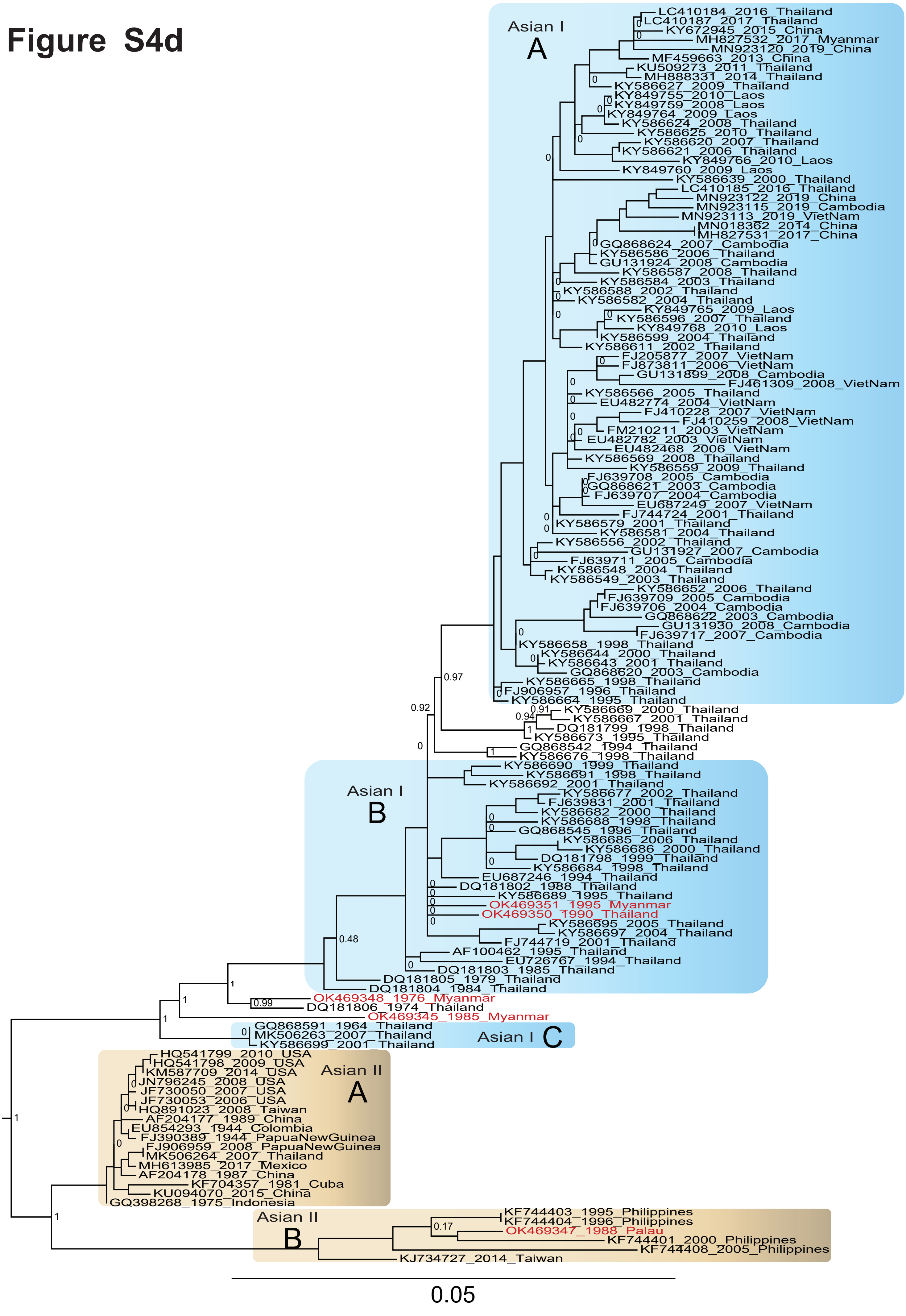

Asian I
A
LC410184_2016_Thailand
LC410187_2017_Thailand
KY672945_2015_China
MH827532_2017_Myanmar
MN923120_2019_China
MF459663_2013_China
KU509273_2011_Thailand
MH888331_2014_Thailand
KY586627_2009_Thailand
KY849755_2010_Laos
KY849759_2008_Laos
KY849764_2009_Laos
KY586624_2008_Thailand
KY586625_2010_Thailand
KY586620_2007_Thailand
KY586621_2006_Thailand
KY849766_2010_Laos
KY849760_2009_Laos
KY586639_2000_Thailand
LC410185_2016_Thailand
MN923122_2019_China
MN923115_2019_Cambodia
MN923113_2019_VietNam
MN018362_2014_China
MH827531_2017_China
GQ868624_2007_Cambodia
KY586586_2006_Thailand
GU131924_2008_Cambodia
KY586587_2008_Thailand
KY586584_2003_Thailand
KY586588_2002_Thailand
KY586582_2004_Thailand
KY849765_2009_Laos
KY586596_2007_Thailand
KY849768_2010_Laos
KY586599_2004_Thailand
KY586611_2002_Thailand
FJ205877_2007_VietNam
FJ873811_2006_VietNam
GU131899_2008_Cambodia
FJ461309_2008_VietNam
KY586566_2005_Thailand
EU482774_2004_VietNam
FJ410228_2007_VietNam
FJ410259_2008_VietNam
FM210211_2003_VietNam
EU482782_2003_VietNam
EU482468_2006_VietNam
KY586569_2008_Thailand
KY586559_2009_Thailand
FJ639708_2005_Cambodia
GQ868621_2003_Cambodia
FJ639707_2004_Cambodia
EU687249_2007_VietNam
FJ744724_2001_Thailand
KY586579_2001_Thailand
KY586581_2004_Thailand
KY586556_2002_Thailand
GU131927_2007_Cambodia
FJ639711_2005_Cambodia
KY586548_2004_Thailand
KY586549_2003_Thailand
KY586652_2006_Thailand
FJ639709_2005_Cambodia
FJ639706_2004_Cambodia
GQ868622_2003_Cambodia
GU131930_2008_Cambodia
FJ639717_2007_Cambodia
KY586658_1998_Thailand
KY586644_2000_Thailand
KY586643_2001_Thailand
GQ868620_2003_Cambodia
KY586665_1998_Thailand
FJ906957_1996_Thailand
KY586664_1995_Thailand
KY586669_2000_Thailand
KY586667_2001_Thailand
DQ181799_1998_Thailand
KY586673_1995_Thailand
GQ868542_1994_Thailand
KY586676_1998_Thailand
Asian I
B
KY586690_1999_Thailand
KY586691_1998_Thailand
KY586692_2001_Thailand
KY586677_2002_Thailand
FJ639831_2001_Thailand
KY586682_2000_Thailand
KY586688_1998_Thailand
GQ868545_1996_Thailand
KY586685_2006_Thailand
KY586686_2000_Thailand
DQ181798_1999_Thailand
KY586684_1998_Thailand
EU687246_1994_Thailand
DQ181802_1988_Thailand
KY586689_1995_Thailand
OK469351_1995_Myanmar
OK469350_1990_Thailand
KY586695_2005_Thailand
KY586697_2004_Thailand
FJ744719_2001_Thailand
AF100462_1995_Thailand
EU726767_1994_Thailand
DQ181803_1985_Thailand
DQ181805_1979_Thailand
DQ181804_1984_Thailand
OK469348_1976_Myanmar
DQ181806_1974_Thailand
OK469345_1985_Myanmar
GQ868591_1964_Thailand
MK506263_2007_Thailand
KY586699_2001_Thailand
Asian I C
Asian II
A
HQ541799_2010_USA
HQ541798_2009_USA
KM587709_2014_USA
JN796245_2008_USA
JF730050_2007_USA
JF730053_2006_USA
HQ891023_2008_Taiwan
AF204177_1989_China
EU854293_1944_Colombia
FJ390389_1944_PapuaNewGuinea
FJ906959_2008_PapuaNewGuinea
MK506264_2007_Thailand
MH613985_2017_Mexico
AF204178_1987_China
KF704357_1981_Cuba
KU094070_2015_China
GQ398268_1975_Indonesia
Asian II
B
KF744403_1995_Philippines
KF744404_1996_Philippines
OK469347_1988_Palau
KF744401_2000_Philippines
KF744408_2005_Philippines
KJ734727_2014_Taiwan
0.97
0.92
0.91
0.94
0.48
0.99
0.17
0.05

Figure S4e

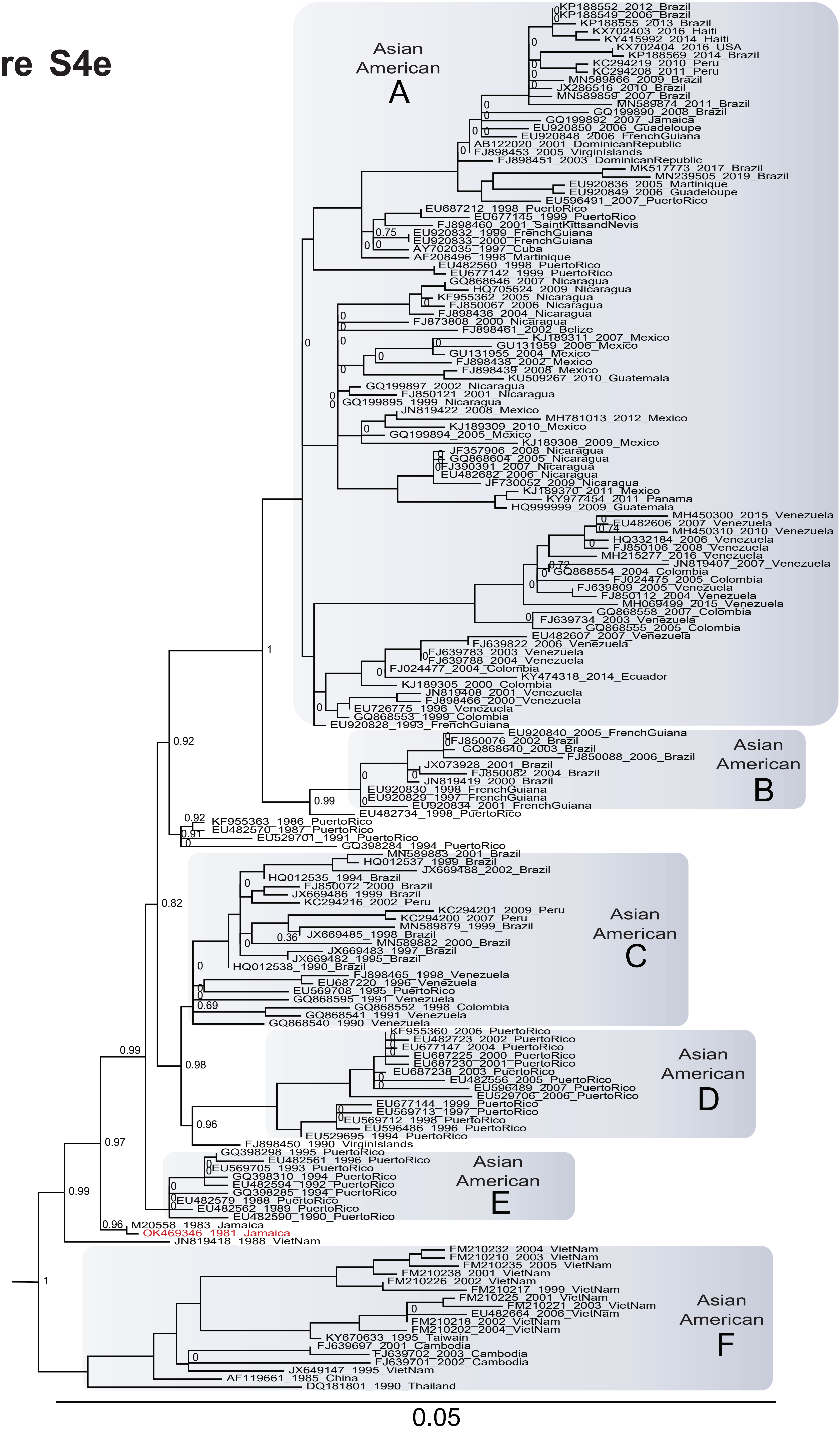

Asian American A
KP188552_2012_Brazil
KP188549_2006_Brazil
KP188555_2013_Brazil
KX702403_2016_Haiti
KY415992_2014_Haiti
KX702404_2016_USA
KP188569_2014_Brazil
KC294219_2010_Peru
KC294208_2011_Peru
MN589866_2009_Brazil
JX286516_2010_Brazil
MN589859_2007_Brazil
MN589874_2011_Brazil
GQ199890_2008_Brazil
GQ199892_2007_Jamaica
EU920850_2006_Guadeloupe
EU920848_2006_FrenchGuiana
AB122020_2001_DominicanRepublic
FJ898453_2005_VirginIslands
FJ898451_2003_DominicanRepublic
MK517773_2017_Brazil
MN239505_2019_Brazil
EU920836_2005_Martinique
EU920849_2006_Guadeloupe
EU596491_2007_PuertoRico
EU687212_1998_PuertoRico
EU677145_1999_PuertoRico
FJ898460_2001_SaintKittsandNevis
EU920832_1999_FrenchGuiana
EU920833_2000_FrenchGuiana
AY702035_1997_Cuba
AF208496_1998_Martinique
EU482560_1998_PuertoRico
EU677142_1999_PuertoRico
GQ868646_2007_Nicaragua
HQ705624_2009_Nicaragua
KF955362_2005_Nicaragua
FJ850067_2006_Nicaragua
FJ898436_2004_Nicaragua
FJ873808_2000_Nicaragua
FJ898461_2002_Belize
KJ189311_2007_Mexico
GU131959_2006_Mexico
GU131955_2004_Mexico
FJ898438_2002_Mexico
FJ898439_2008_Mexico
KU509267_2010_Guatemala
GQ199897_2002_Nicaragua
FJ850121_2001_Nicaragua
GQ199895_1999_Nicaragua
JN819422_2008_Mexico
MH781013_2012_Mexico
KJ189309_2010_Mexico
GQ199894_2005_Mexico
KJ189308_2009_Mexico
JF357906_2008_Nicaragua
GQ868604_2005_Nicaragua
FJ390391_2007_Nicaragua
EU482682_2006_Nicaragua
JF730052_2009_Nicaragua
KJ189370_2011_Mexico
KY977454_2011_Panama
HQ999999_2009_Guatemala
MH450300_2015_Venezuela
EU482606_2007_Venezuela
MH450310_2010_Venezuela
HQ332184_2006_Venezuela
FJ850106_2008_Venezuela
MH215277_2016_Venezuela
JN819407_2007_Venezuela
GQ868554_2004_Colombia
FJ024475_2005_Colombia
FJ639809_2005_Venezuela
FJ850112_2004_Venezuela
MH069499_2015_Venezuela
GQ868558_2007_Colombia
FJ639734_2003_Venezuela
GQ868555_2005_Colombia
EU482607_2007_Venezuela
FJ639822_2006_Venezuela
FJ639783_2003_Venezuela
FJ639788_2004_Venezuela
FJ024477_2004_Colombia
KY474318_2014_Ecuador
KJ189305_2000_Colombia
JN819408_2001_Venezuela
FJ898466_2000_Venezuela
EU726775_1996_Venezuela
GQ868553_1999_Colombia
EU920828_1993_FrenchGuiana
Asian American B
EU920840_2005_FrenchGuiana
FJ850076_2002_Brazil
GQ868640_2003_Brazil
FJ850088_2006_Brazil
JX073928_2001_Brazil
FJ850082_2004_Brazil
JN819419_2000_Brazil
EU920830_1998_FrenchGuiana
EU920829_1997_FrenchGuiana
EU920834_2001_FrenchGuiana
EU482734_1998_PuertoRico
KF955363_1986_PuertoRico
EU482570_1987_PuertoRico
EU529701_1991_PuertoRico
GQ398284_1994_PuertoRico
Asian American C
MN589883_2001_Brazil
HQ012537_1999_Brazil
JX669488_2002_Brazil
HQ012535_1994_Brazil
FJ850072_2000_Brazil
JX669486_1999_Brazil
KC294216_2002_Peru
KC294201_2009_Peru
KC294200_2007_Peru
MN589879_1999_Brazil
JX669485_1998_Brazil
MN589882_2000_Brazil
JX669483_1997_Brazil
JX669482_1995_Brazil
HQ012538_1990_Brazil
FJ898465_1998_Venezuela
EU687220_1996_Venezuela
EU569708_1995_PuertoRico
GQ868595_1991_Venezuela
GQ868552_1998_Colombia
GQ868541_1991_Venezuela
GQ868540_1990_Venezuela
Asian American D
KF955360_2006_PuertoRico
EU482723_2002_PuertoRico
EU677147_2004_PuertoRico
EU687225_2000_PuertoRico
EU687230_2001_PuertoRico
EU687238_2003_PuertoRico
EU482556_2005_PuertoRico
EU596489_2007_PuertoRico
EU529706_2006_PuertoRico
EU677144_1999_PuertoRico
EU569713_1997_PuertoRico
EU569712_1998_PuertoRico
EU596486_1996_PuertoRico
EU529695_1994_PuertoRico
FJ898450_1990_VirginIslands
Asian American E
GQ398298_1995_PuertoRico
EU482561_1996_PuertoRico
EU569705_1993_PuertoRico
GQ398310_1994_PuertoRico
EU482594_1992_PuertoRico
GQ398285_1994_PuertoRico
EU482579_1988_PuertoRico
EU482562_1989_PuertoRico
EU482590_1990_PuertoRico
M20558_1983_Jamaica
OK469346_T981_Jamaica
JN819418_1988_VietNam
Asian American F
FM210232_2004_VietNam
FM210210_2003_VietNam
FM210235_2005_VietNam
FM210238_2001_VietNam
FM210226_2002_VietNam
FM210217_1999_VietNam
FM210225_2001_VietNam
FM210221_2003_VietNam
EU482664_2006_VietNam
FM210218_2002_VietNam
FM210202_2004_VietNam
KY670633_1995_Taiwan
FJ639697_2001_Cambodia
FJ639702_2003_Cambodia
FJ639701_2002_Cambodia
JX649147_1995_VietNam
AF119661_1985_China
DQ181801_1990_Thailand
0.05

Supplemental Figure S5: DENV3 Whole Genome ML Trees. (a) DENV3 Whole Genome ML tree, (b) Genotype 3-III, (c) Genotype 3-II, (d) Genotype 3-I. aLRT SH-like branch support indicated for the major branches. Support values >0.8 have been eliminated in most instances for clarity. Red circles/font indicate newly sequenced isolates from the QUT Arbovirus collection. Branches under positive selection (see section 3.2.2) are indicated by a red star.

Figure S5a

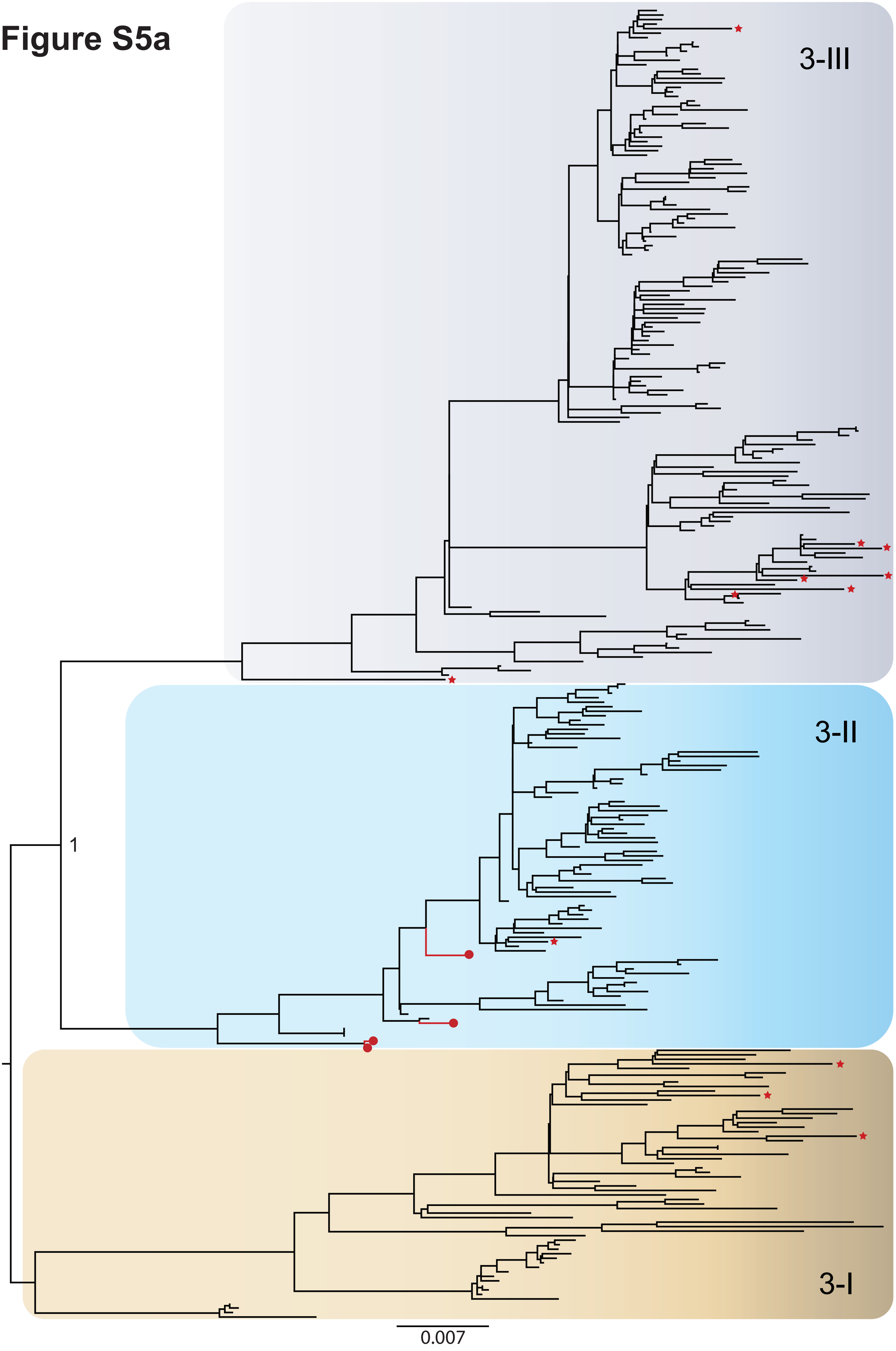

Figure S5b

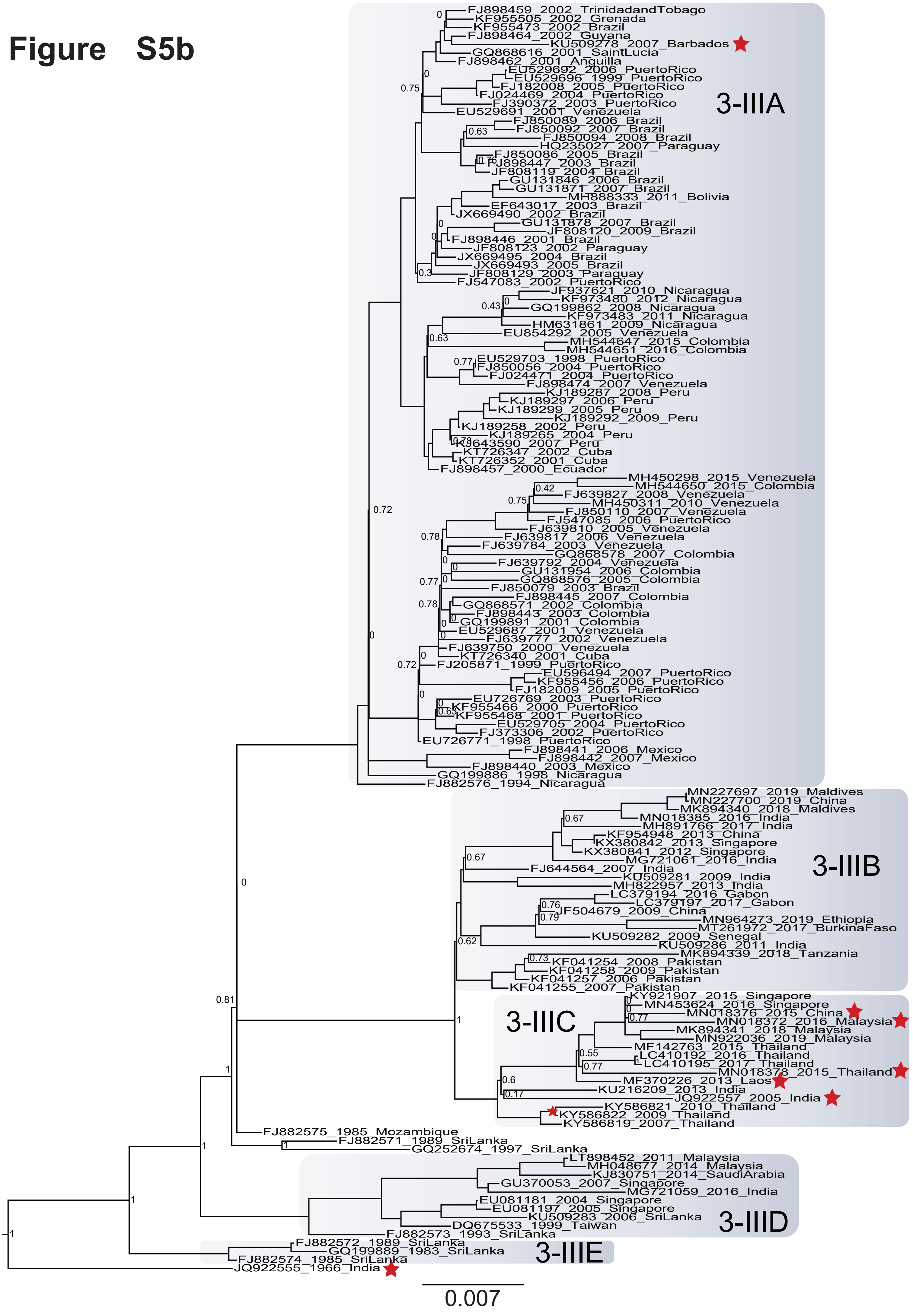

3-IIIA
FJ898459_2002_TrinidadandTobago
KF955505_2002_Grenada
KF955473_2002_Brazil
FJ898464_2002_Guyana
KU509278_2007_Barbados
GQ868616_2001_SaintLucia
FJ898462_2001_Anguilla
EU529692_2006_PuertoRico
EU529696_1999_PuertoRico
FJ182008_2005_PuertoRico
FJ024469_2004_PuertoRico
FJ390372_2003_PuertoRico
EU529691_2001_Venezuela
FJ850089_2006_Brazil
FJ850092_2007_Brazil
FJ850094_2008_Brazil
HQ235027_2007_Paraguay
FJ850086_2005_Brazil
FJ898447_2003_Brazil
JF808119_2004_Brazil
GU131846_2006_Brazil
GU131871_2007_Brazil
MH888333_2011_Bolivia
EF643017_2003_Brazil
JX669490_2002_Brazil
GU131878_2007_Brazil
JF808120_2009_Brazil
FJ898446_2001_Brazil
JF808123_2002_Paraguay
JX669495_2004_Brazil
JX669493_2005_Brazil
JF808129_2003_Paraguay
FJ547083_2002_PuertoRico
JF937621_2010_Nicaragua
KF973480_2012_Nicaragua
GQ199862_2008_Nicaragua
KF973483_2011_Nicaragua
HM631861_2009_Nicaragua
EU854292_2005_Venezuela
MH544647_2015_Colombia
MH544651_2016_Colombia
EU529703_1998_PuertoRico
FJ850056_2004_PuertoRico
FJ024471_2004_PuertoRico
FJ898474_2007_Venezuela
KJ189287_2008_Peru
KJ189297_2006_Peru
KJ189299_2005_Peru
KJ189292_2009_Peru
KJ189258_2002_Peru
KJ189265_2004_Peru
KJ643590_2007_Peru
KT726347_2002_Cuba
KT726352_2001_Cuba
FJ898457_2000_Ecuador
MH450298_2015_Venezuela
MH544650_2015_Colombia
FJ639827_2008_Venezuela
MH450311_2010_Venezuela
FJ850110_2007_Venezuela
FJ547085_2006_PuertoRico
FJ639810_2005_Venezuela
FJ639817_2006_Venezuela
FJ639784_2003_Venezuela
GQ868578_2007_Colombia
FJ639792_2004_Venezuela
GU131954_2006_Colombia
GQ868576_2005_Colombia
FJ850079_2003_Brazil
FJ898445_2007_Colombia
GQ868571_2002_Colombia
FJ898443_2003_Colombia
GQ199891_2001_Colombia
EU529687_2001_Venezuela
FJ639777_2002_Venezuela
FJ639750_2000_Venezuela
KT726340_2001_Cuba
FJ205871_1999_PuertoRico
EU596494_2007_PuertoRico
KF955456_2006_PuertoRico
FJ182009_2005_PuertoRico
EU726769_2003_PuertoRico
KF955466_2000_PuertoRico
KF955468_2001_PuertoRico
EU529705_2004_PuertoRico
FJ373306_2002_PuertoRico
EU726771_1998_PuertoRico
FJ898441_2006_Mexico
FJ898442_2007_Mexico
FJ898440_2003_Mexico
GQ199886_1998_Nicaragua
FJ882576_1994_Nicaragua
3-IIIB
MN227697_2019_Maldives
MN227700_2019_China
MK894340_2018_Maldives
MN018385_2016_India
MH891766_2017_India
KF954948_2013_China
KX380842_2013_Singapore
KX380841_2012_Singapore
MG721061_2016_India
FJ644564_2007_India
KU509281_2009_India
MH822957_2013_India
LC379194_2016_Gabon
LC379197_2017_Gabon
JF504679_2009_China
MN964273_2019_Ethiopia
MT261972_2017_BurkinaFaso
KU509282_2009_Senegal
KU509286_2011_India
MK894339_2018_Tanzania
KF041254_2008_Pakistan
KF041258_2009_Pakistan
KF041257_2006_Pakistan
KF041255_2007_Pakistan
3-IIIC
KY921907_2015_Singapore
MN453624_2016_Singapore
MN018376_2015_China
MN018372_2016_Malaysia
MK894341_2018_Malaysia
MN922036_2019_Malaysia
MF142763_2015_Thailand
LC410192_2016_Thailand
LC410195_2017_Thailand
MN018378_2015_Thailand
MF370226_2013_Laos
KU216209_2013_India
JQ922557_2005_India
KY586821_2010_Thailand
KY586822_2009_Thailand
KY586819_2007_Thailand
FJ882575_1985_Mozambique
FJ882571_1989_SriLanka
GQ252674_1997_SriLanka
3-IIID
LT898452_2011_Malaysia
MH048677_2014_Malaysia
KJ830751_2014_SaudiArabia
GU370053_2007_Singapore
MG721059_2016_India
EU081181_2004_Singapore
EU081197_2005_Singapore
KU509283_2006_SriLanka
DQ675533_1999_Taiwan
FJ882573_1993_SriLanka
3-IIIE
FJ882572_1989_SriLanka
GQ199889_1983_SriLanka
FJ882574_1985_SriLanka
JQ922555_1966_India
0.007

Figure S5c

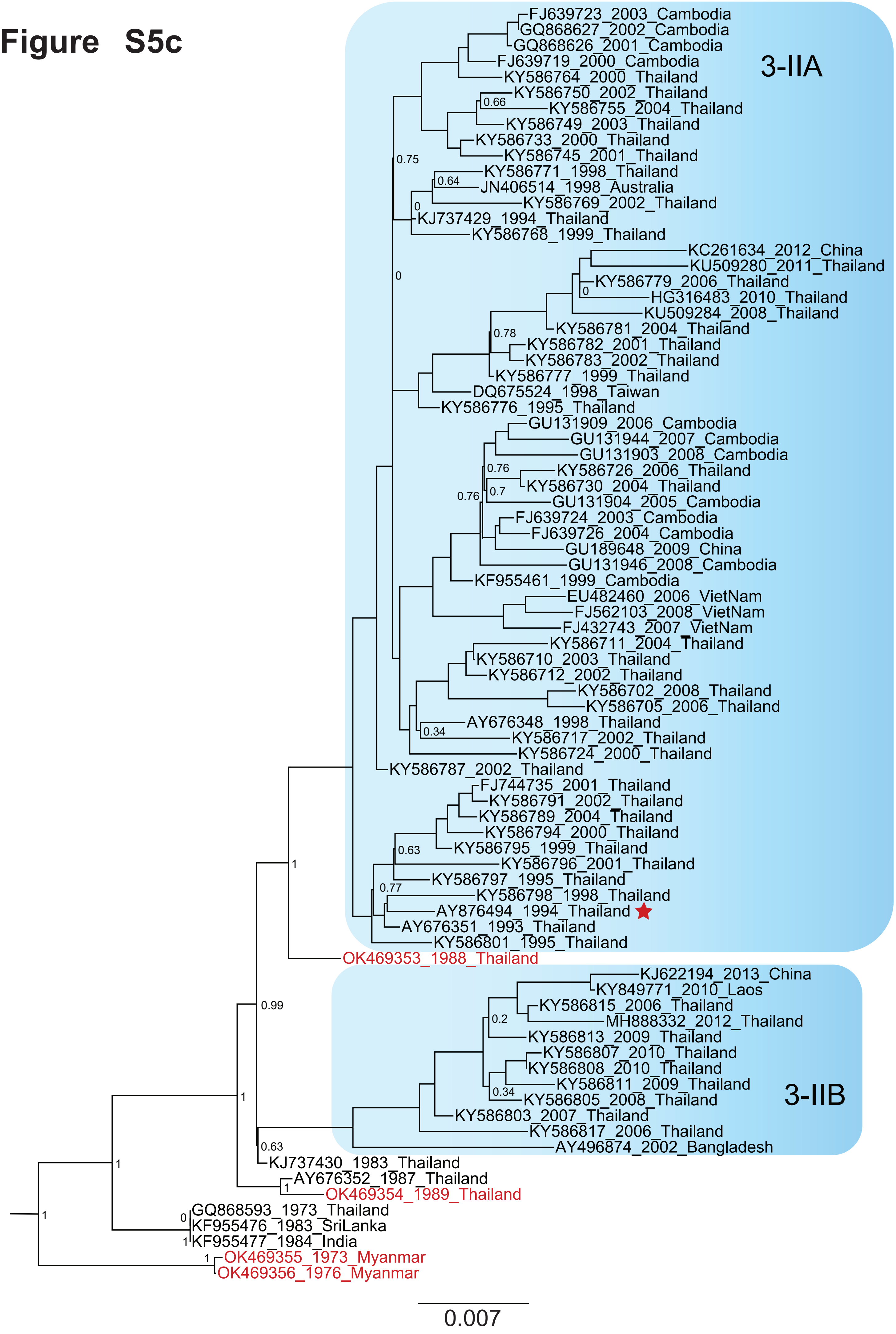
3-IIA
FJ639723_2003_Cambodia
GQ868627_2002_Cambodia
GQ868626_2001_Cambodia
FJ639719_2000_Cambodia
KY586764_2000_Thailand
KY586750_2002_Thailand
0.66
KY586755_2004_Thailand
KY586749_2003_Thailand
KY586733_2000_Thailand
KY586745_2001_Thailand
0.75
KY586771_1998_Thailand
0.64
JN406514_1998_Australia
KY586769_2002_Thailand
0
KJ737429_1994_Thailand
KY586768_1999_Thailand
KC261634_2012_China
KU509280_2011_Thailand
0
KY586779_2006_Thailand
0
HG316483_2010_Thailand
KU509284_2008_Thailand
KY586781_2004_Thailand
0.78
KY586782_2001_Thailand
KY586783_2002_Thailand
KY586777_1999_Thailand
DQ675524_1998_Taiwan
KY586776_1995_Thailand
GU131909_2006_Cambodia
GU131944_2007_Cambodia
GU131903_2008_Cambodia
0.76
KY586726_2006_Thailand
KY586730_2004_Thailand
0.7
0.76
GU131904_2005_Cambodia
FJ639724_2003_Cambodia
FJ639726_2004_Cambodia
GU189648_2009_China
GU131946_2008_Cambodia
KF955461_1999_Cambodia
EU482460_2006_VietNam
FJ562103_2008_VietNam
FJ432743_2007_VietNam
KY586711_2004_Thailand
KY586710_2003_Thailand
KY586712_2002_Thailand
KY586702_2008_Thailand
KY586705_2006_Thailand
AY676348_1998_Thailand
0.34
KY586717_2002_Thailand
KY586724_2000_Thailand
KY586787_2002_Thailand
FJ744735_2001_Thailand
KY586791_2002_Thailand
KY586789_2004_Thailand
KY586794_2000_Thailand
KY586795_1999_Thailand
0.63
KY586796_2001_Thailand
1
KY586797_1995_Thailand
0.77
KY586798_1998_Thailand
AY876494_1994_Thailand
AY676351_1993_Thailand
KY586801_1995_Thailand
OK469353_1988_Thailand
KJ622194_2013_China
1
KY849771_2010_Laos
0.99
KY586815_2006_Thailand
0.2
MH888332_2012_Thailand
KY586813_2009_Thailand
KY586807_2010_Thailand
KY586808_2010_Thailand
KY586811_2009_Thailand
1
0.34
KY586805_2008_Thailand
3-IIB
KY586803_2007_Thailand
KY586817_2006_Thailand
AY496874_2002_Bangladesh
0.63
1
KJ737430_1983_Thailand
AY676352_1987_Thailand
1
OK469354_1989_Thailand
GQ868593_1973_Thailand
0
KF955476_1983_SriLanka
1
1
KF955477_1984_India
1
OK469355_1973_Myanmar
OK469356_1976_Myanmar
0.007

Figure S5d

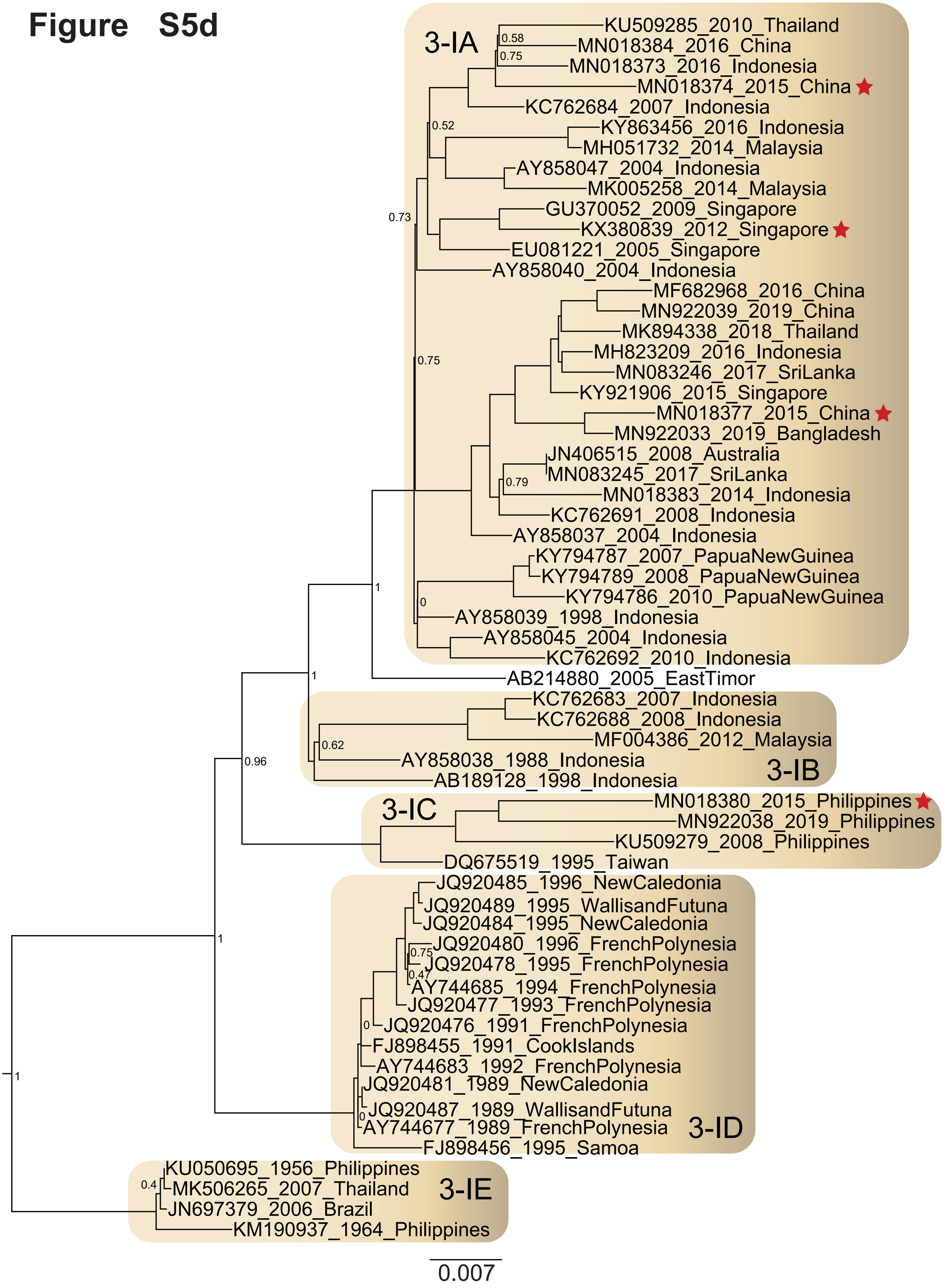

3-IA
KU509285_2010_Thailand
MN018384_2016_China
MN018373_2016_Indonesia
MN018374_2015_China
KC762684_2007_Indonesia
KY863456_2016_Indonesia
MH051732_2014_Malaysia
AY858047_2004_Indonesia
MK005258_2014_Malaysia
GU370052_2009_Singapore
KX380839_2012_Singapore
EU081221_2005_Singapore
AY858040_2004_Indonesia
MF682968_2016_China
MN922039_2019_China
MK894338_2018_Thailand
MH823209_2016_Indonesia
MN083246_2017_SriLanka
KY921906_2015_Singapore
MN018377_2015_China
MN922033_2019_Bangladesh
JN406515_2008_Australia
MN083245_2017_SriLanka
MN018383_2014_Indonesia
KC762691_2008_Indonesia
AY858037_2004_Indonesia
KY794787_2007_PapuaNewGuinea
KY794789_2008_PapuaNewGuinea
KY794786_2010_PapuaNewGuinea
AY858039_1998_Indonesia
AY858045_2004_Indonesia
KC762692_2010_Indonesia
AB214880_2005_EastTimor
KC762683_2007_Indonesia
KC762688_2008_Indonesia
MF004386_2012_Malaysia
AY858038_1988_Indonesia
AB189128_1998_Indonesia
3-IB
3-IC
MN018380_2015_Philippines
MN922038_2019_Philippines
KU509279_2008_Philippines
DQ675519_1995_Taiwan
JQ920485_1996_NewCaledonia
JQ920489_1995_WallisandFutuna
JQ920484_1995_NewCaledonia
JQ920480_1996_FrenchPolynesia
JQ920478_1995_FrenchPolynesia
AY744685_1994_FrenchPolynesia
JQ920477_1993_FrenchPolynesia
JQ920476_1991_FrenchPolynesia
FJ898455_1991_CookIslands
AY744683_1992_FrenchPolynesia
JQ920481_1989_NewCaledonia
JQ920487_1989_WallisandFutuna
AY744677_1989_FrenchPolynesia
FJ898456_1995_Samoa
3-ID
KU050695_1956_Philippines
MK506265_2007_Thailand
JN697379_2006_Brazil
KM190937_1964_Philippines
3-IE
0.58
0.75
0.52
0.73
0.75
0.79
0
1
1
0.62
0.96
0.75
0.47
0
0
1
1
0.4
0.007

Supplemental Figure S6: DENV3 E Gene ML Trees. (a) DENV3 E gene ML tree, (b) Genotype 3-III, (c) Genotype 3-II, (d) Genotype 3-I. aLRT SH-like branch support indicated for the major branches. Support values >0.8 have been eliminated in most instances for clarity. Red circles/font indicate newly sequenced isolates from the QUT Arbovirus collection. Branches under positive selection (see section 3.2.2) are indicated by a red star.

Figure S6a

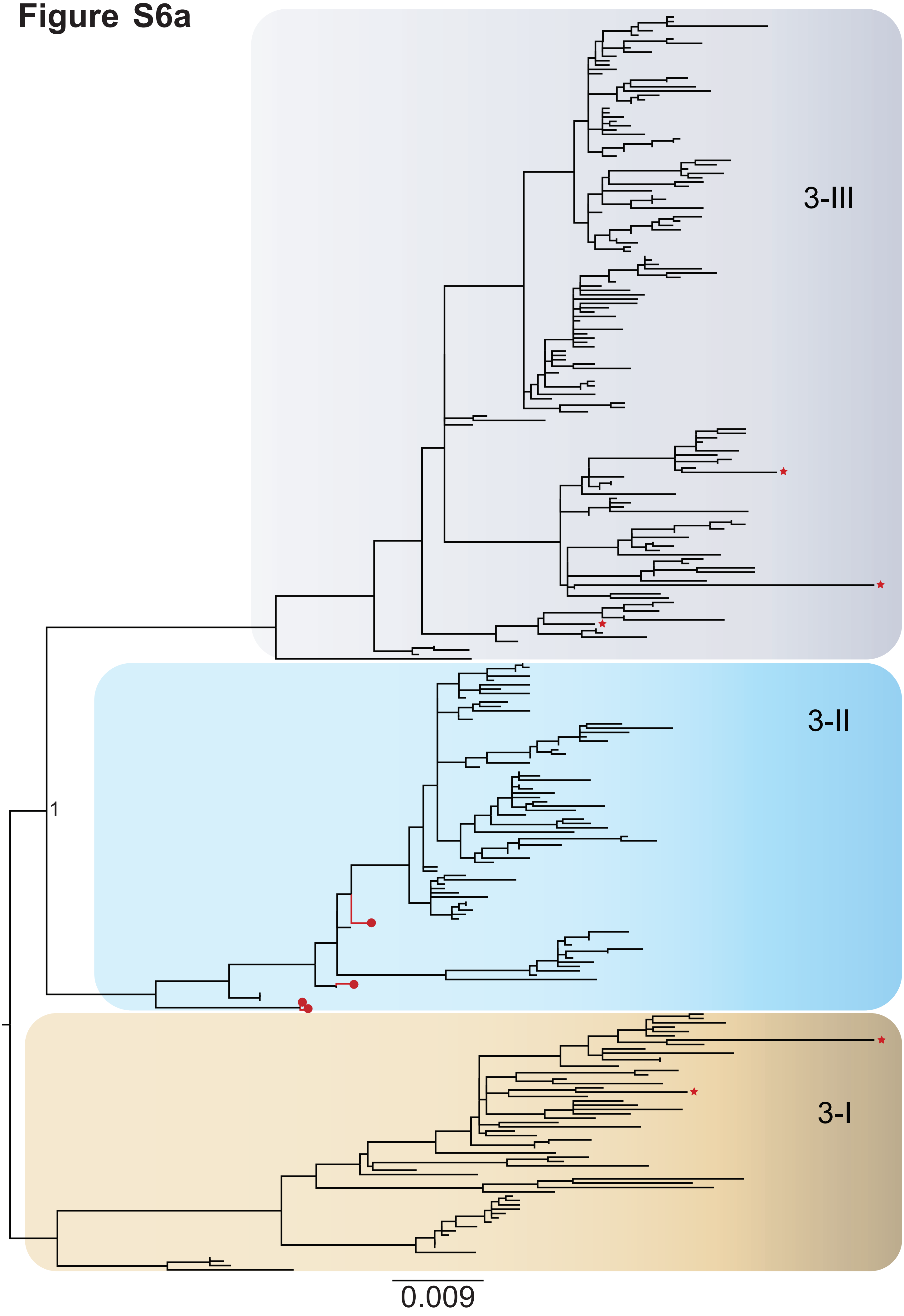

Figure S6b

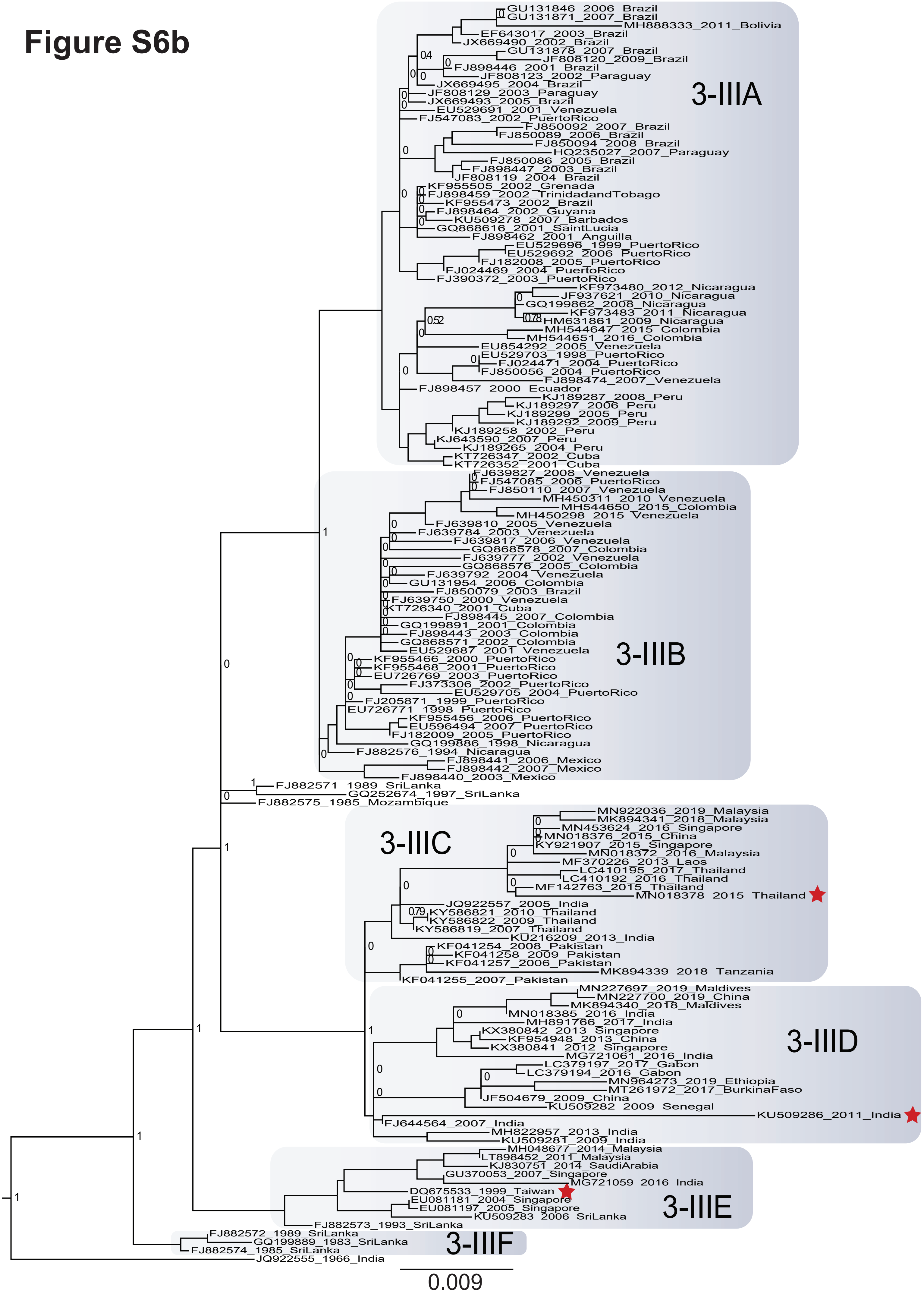

3-IIIA
GU131846_2006_Brazil
GU131871_2007_Brazil
MH888333_2011_Bolivia
EF643017_2003_Brazil
JX669490_2002_Brazil
GU131878_2007_Brazil
JF808120_2009_Brazil
FJ898446_2001_Brazil
JF808123_2002_Paraguay
JX669495_2004_Brazil
JF808129_2003_Paraguay
JX669493_2005_Brazil
EU529691_2001_Venezuela
FJ547083_2002_PuertoRico
FJ850092_2007_Brazil
FJ850089_2006_Brazil
FJ850094_2008_Brazil
HQ235027_2007_Paraguay
FJ850086_2005_Brazil
FJ898447_2003_Brazil
JF808119_2004_Brazil
KF955505_2002_Grenada
FJ898459_2002_TrinidadandTobago
KF955473_2002_Brazil
FJ898464_2002_Guyana
KU509278_2007_Barbados
GQ868616_2001_SaintLucia
FJ898462_2001_Anguilla
EU529696_1999_PuertoRico
EU529692_2006_PuertoRico
FJ182008_2005_PuertoRico
FJ024469_2004_PuertoRico
FJ390372_2003_PuertoRico
KF973480_2012_Nicaragua
JF937621_2010_Nicaragua
GQ199862_2008_Nicaragua
KF973483_2011_Nicaragua
HM631861_2009_Nicaragua
MH544647_2015_Colombia
MH544651_2016_Colombia
EU854292_2005_Venezuela
EU529703_1998_PuertoRico
FJ024471_2004_PuertoRico
FJ850056_2004_PuertoRico
FJ898474_2007_Venezuela
FJ898457_2000_Ecuador
KJ189287_2008_Peru
KJ189297_2006_Peru
KJ189299_2005_Peru
KJ189292_2009_Peru
KJ189258_2002_Peru
KJ643590_2007_Peru
KJ189265_2004_Peru
KT726347_2002_Cuba
KT726352_2001_Cuba
3-IIIB
FJ639827_2008_Venezuela
FJ547085_2006_PuertoRico
FJ850110_2007_Venezuela
MH450311_2010_Venezuela
MH544650_2015_Colombia
MH450298_2015_Venezuela
FJ639810_2005_Venezuela
FJ639784_2003_Venezuela
FJ639817_2006_Venezuela
GQ868578_2007_Colombia
FJ639777_2002_Venezuela
GQ868576_2005_Colombia
FJ639792_2004_Venezuela
GU131954_2006_Colombia
FJ850079_2003_Brazil
FJ639750_2000_Venezuela
KT726340_2001_Cuba
FJ898445_2007_Colombia
GQ199891_2001_Colombia
FJ898443_2003_Colombia
GQ868571_2002_Colombia
EU529687_2001_Venezuela
KF955466_2000_PuertoRico
KF955468_2001_PuertoRico
EU726769_2003_PuertoRico
FJ373306_2002_PuertoRico
EU529705_2004_PuertoRico
FJ205871_1999_PuertoRico
EU726771_1998_PuertoRico
KF955456_2006_PuertoRico
EU596494_2007_PuertoRico
FJ182009_2005_PuertoRico
GQ199886_1998_Nicaragua
FJ882576_1994_Nicaragua
FJ898441_2006_Mexico
FJ898442_2007_Mexico
FJ898440_2003_Mexico
FJ882571_1989_SriLanka
GQ252674_1997_SriLanka
FJ882575_1985_Mozambique
3-IIIC
MN922036_2019_Malaysia
MK894341_2018_Malaysia
MN453624_2016_Singapore
MN018376_2015_China
KY921907_2015_Singapore
MN018372_2016_Malaysia
MF370226_2013_Laos
LC410195_2017_Thailand
LC410192_2016_Thailand
MF142763_2015_Thailand
MN018378_2015_Thailand
JQ922557_2005_India
KY586821_2010_Thailand
KY586822_2009_Thailand
KY586819_2007_Thailand
KU216209_2013_India
KF041254_2008_Pakistan
KF041258_2009_Pakistan
KF041257_2006_Pakistan
MK894339_2018_Tanzania
KF041255_2007_Pakistan
3-IIID
MN227697_2019_Maldives
MN227700_2019_China
MK894340_2018_Maldives
MN018385_2016_India
MH891766_2017_India
KX380842_2013_Singapore
KF954948_2013_China
KX380841_2012_Singapore
MG721061_2016_India
LC379197_2017_Gabon
LC379194_2016_Gabon
MN964273_2019_Ethiopia
MT261972_2017_BurkinaFaso
JF504679_2009_China
KU509282_2009_Senegal
KU509286_2011_India
FJ644564_2007_India
MH822957_2013_India
KU509281_2009_India
3-IIIE
MH048677_2014_Malaysia
LT898452_2011_Malaysia
KJ830751_2014_SaudiArabia
GU370053_2007_Singapore
MG721059_2016_India
DQ675533_1999_Taiwan
EU081181_2004_Singapore
EU081197_2005_Singapore
KU509283_2006_SriLanka
FJ882573_1993_SriLanka
3-IIIF
FJ882572_1989_SriLanka
GQ199889_1983_SriLanka
FJ882574_1985_SriLanka
JQ922555_1966_India
0.009

Figure S6c

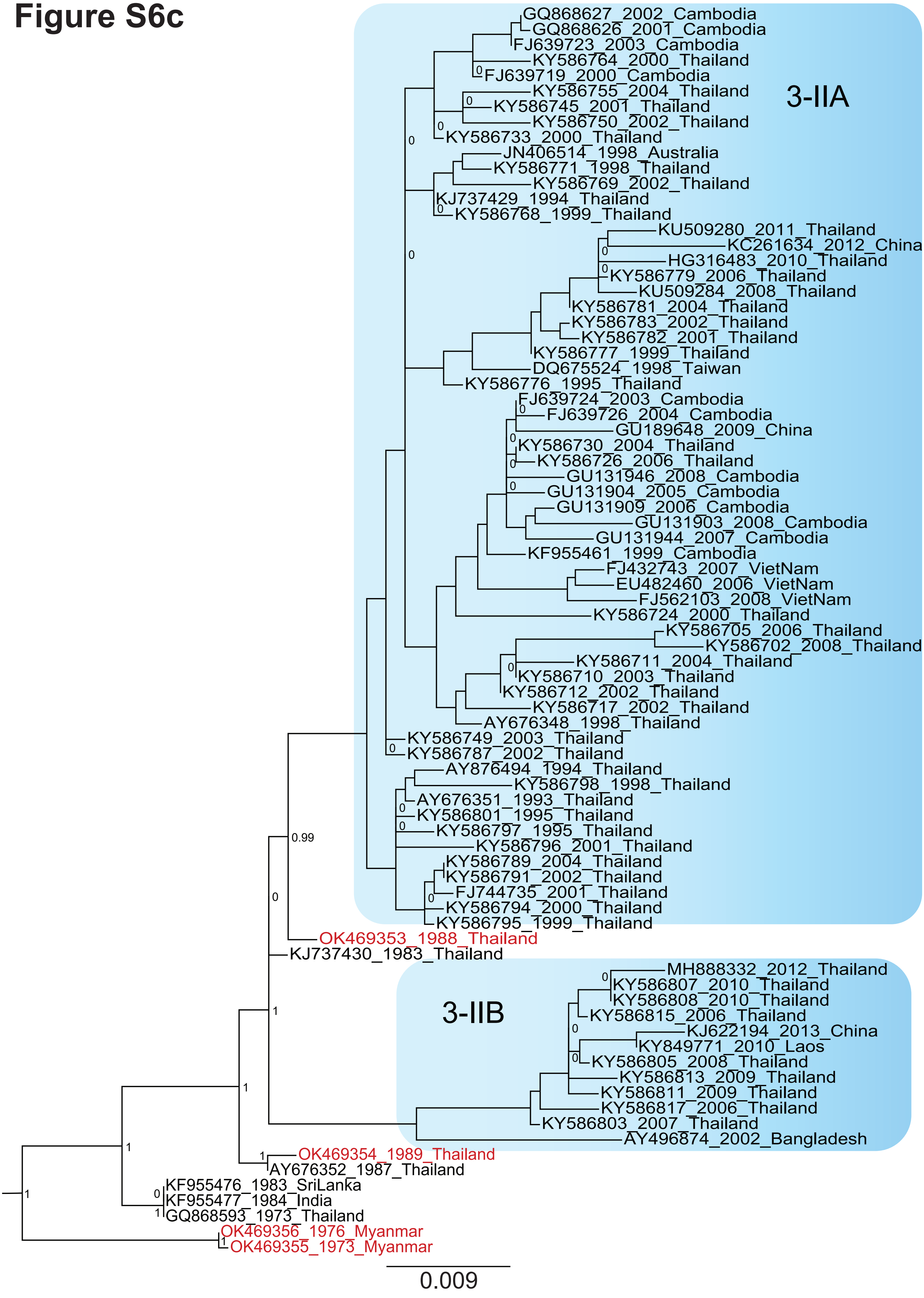

GQ868627_2002_Cambodia
GQ868626_2001_Cambodia
FJ639723_2003_Cambodia
KY586764_2000_Thailand
FJ639719_2000_Cambodia
KY586755_2004_Thailand
KY586745_2001_Thailand
KY586750_2002_Thailand
KY586733_2000_Thailand
JN406514_1998_Australia
KY586771_1998_Thailand
KY586769_2002_Thailand
KJ737429_1994_Thailand
KY586768_1999_Thailand
KU509280_2011_Thailand
KC261634_2012_China
HG316483_2010_Thailand
KY586779_2006_Thailand
KU509284_2008_Thailand
KY586781_2004_Thailand
KY586783_2002_Thailand
KY586782_2001_Thailand
KY586777_1999_Thailand
DQ675524_1998_Taiwan
KY586776_1995_Thailand
FJ639724_2003_Cambodia
FJ639726_2004_Cambodia
GU189648_2009_China
KY586730_2004_Thailand
KY586726_2006_Thailand
GU131946_2008_Cambodia
GU131904_2005_Cambodia
GU131909_2006_Cambodia
GU131903_2008_Cambodia
GU131944_2007_Cambodia
KF955461_1999_Cambodia
FJ432743_2007_VietNam
EU482460_2006_VietNam
FJ562103_2008_VietNam
KY586724_2000_Thailand
KY586705_2006_Thailand
KY586702_2008_Thailand
KY586711_2004_Thailand
KY586710_2003_Thailand
KY586712_2002_Thailand
KY586717_2002_Thailand
AY676348_1998_Thailand
KY586749_2003_Thailand
KY586787_2002_Thailand
AY876494_1994_Thailand
KY586798_1998_Thailand
AY676351_1993_Thailand
KY586801_1995_Thailand
KY586797_1995_Thailand
KY586796_2001_Thailand
KY586789_2004_Thailand
KY586791_2002_Thailand
FJ744735_2001_Thailand
KY586794_2000_Thailand
KY586795_1999_Thailand
3-IIA
0.99
OK469353_1988_Thailand
KJ737430_1983_Thailand
MH888332_2012_Thailand
KY586807_2010_Thailand
KY586808_2010_Thailand
KY586815_2006_Thailand
KJ622194_2013_China
KY849771_2010_Laos
KY586805_2008_Thailand
KY586813_2009_Thailand
KY586811_2009_Thailand
KY586817_2006_Thailand
KY586803_2007_Thailand
AY496874_2002_Bangladesh
3-IIB
OK469354_1989_Thailand
AY676352_1987_Thailand
KF955476_1983_SriLanka
KF955477_1984_India
GQ868593_1973_Thailand
OK469356_1976_Myanmar
OK469355_1973_Myanmar
0.009

Figure S6d

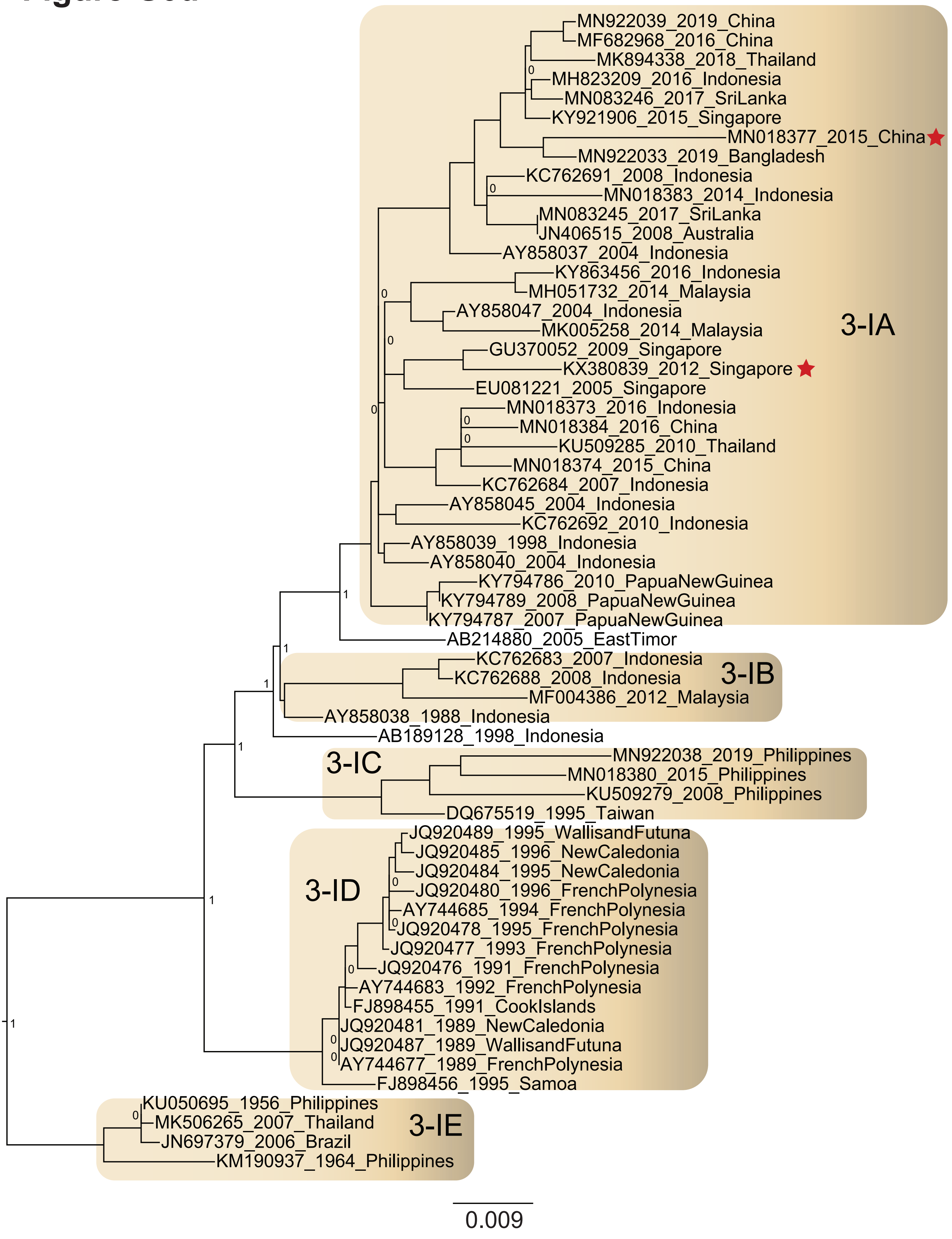

MN922039_2019_China
MF682968_2016_China
MK894338_2018_Thailand
MH823209_2016_Indonesia
MN083246_2017_SriLanka
KY921906_2015_Singapore
MN018377_2015_China
MN922033_2019_Bangladesh
KC762691_2008_Indonesia
MN018383_2014_Indonesia
MN083245_2017_SriLanka
JN406515_2008_Australia
AY858037_2004_Indonesia
KY863456_2016_Indonesia
MH051732_2014_Malaysia
AY858047_2004_Indonesia
MK005258_2014_Malaysia
3-IA
GU370052_2009_Singapore
KX380839_2012_Singapore
EU081221_2005_Singapore
MN018373_2016_Indonesia
MN018384_2016_China
KU509285_2010_Thailand
MN018374_2015_China
KC762684_2007_Indonesia
AY858045_2004_Indonesia
KC762692_2010_Indonesia
AY858039_1998_Indonesia
AY858040_2004_Indonesia
KY794786_2010_PapuaNewGuinea
KY794789_2008_PapuaNewGuinea
KY794787_2007_PapuaNewGuinea
AB214880_2005_EastTimor
KC762683_2007_Indonesia
KC762688_2008_Indonesia
3-IB
MF004386_2012_Malaysia
AY858038_1988_Indonesia
AB189128_1998_Indonesia
3-IC
MN922038_2019_Philippines
MN018380_2015_Philippines
KU509279_2008_Philippines
DQ675519_1995_Taiwan
JQ920489_1995_WallisandFutuna
JQ920485_1996_NewCaledonia
JQ920484_1995_NewCaledonia
3-ID
JQ920480_1996_FrenchPolynesia
AY744685_1994_FrenchPolynesia
JQ920478_1995_FrenchPolynesia
JQ920477_1993_FrenchPolynesia
JQ920476_1991_FrenchPolynesia
AY744683_1992_FrenchPolynesia
FJ898455_1991_CookIslands
JQ920481_1989_NewCaledonia
JQ920487_1989_WallisandFutuna
AY744677_1989_FrenchPolynesia
FJ898456_1995_Samoa
KU050695_1956_Philippines
MK506265_2007_Thailand
3-IE
JN697379_2006_Brazil
KM190937_1964_Philippines
0.009

Supplemental Figure S7: DENV4 Whole Genome ML Trees. (a) DENV4 Whole Genome ML tree, (b) Genotype 4-II, (c) Genotypes 4-I, 4-III, and sylvatic. aLRT SH-like branch support indicated for the major branches. Support values >0.8 have been eliminated in most instances for clarity. Red circles/font indicate newly sequenced isolates from the QUT Arbovirus collection. Branches under positive selection (see section 3.2.2) are indicated by a red star.

Figure S7a

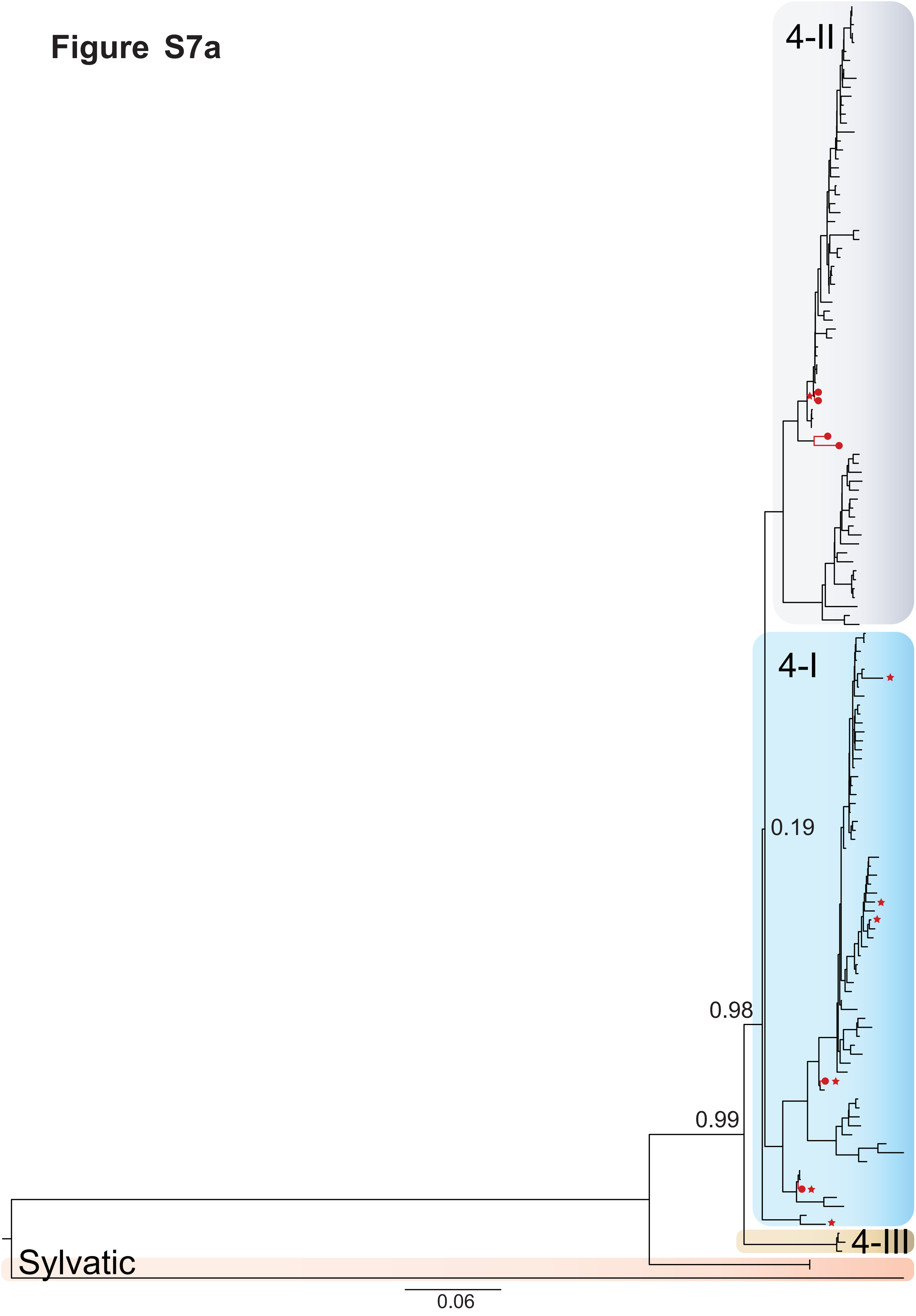

Figure S7b

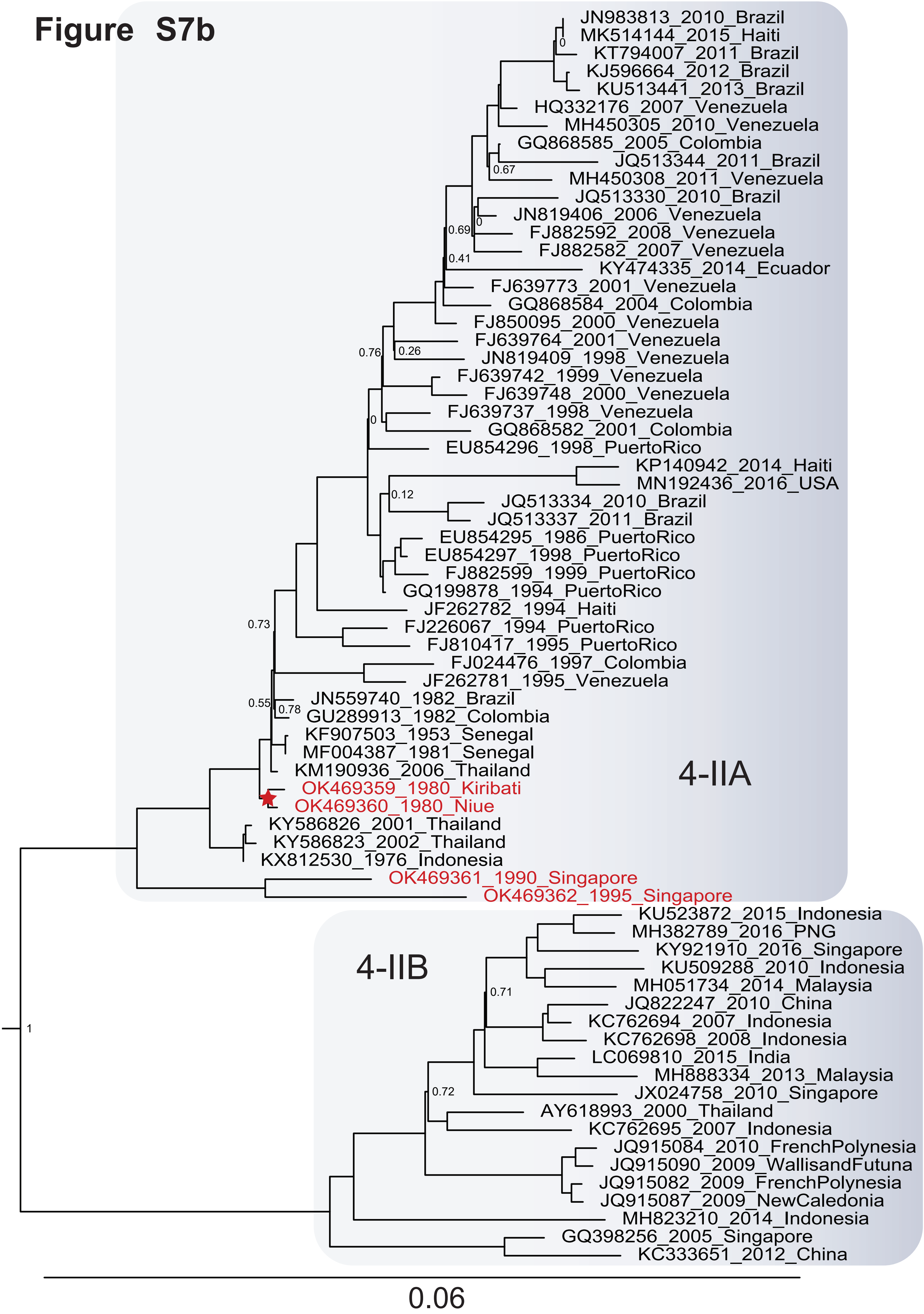
JN983813_2010_Brazil
MK514144_2015_Haiti
KT794007_2011_Brazil
KJ596664_2012_Brazil
KU513441_2013_Brazil
HQ332176_2007_Venezuela
MH450305_2010_Venezuela
GQ868585_2005_Colombia
JQ513344_2011_Brazil
MH450308_2011_Venezuela
JQ513330_2010_Brazil
JN819406_2006_Venezuela
FJ882592_2008_Venezuela
FJ882582_2007_Venezuela
KY474335_2014_Ecuador
FJ639773_2001_Venezuela
GQ868584_2004_Colombia
FJ850095_2000_Venezuela
FJ639764_2001_Venezuela
JN819409_1998_Venezuela
FJ639742_1999_Venezuela
FJ639748_2000_Venezuela
FJ639737_1998_Venezuela
GQ868582_2001_Colombia
EU854296_1998_PuertoRico
KP140942_2014_Haiti
MN192436_2016_USA
JQ513334_2010_Brazil
JQ513337_2011_Brazil
EU854295_1986_PuertoRico
EU854297_1998_PuertoRico
FJ882599_1999_PuertoRico
GQ199878_1994_PuertoRico
JF262782_1994_Haiti
FJ226067_1994_PuertoRico
FJ810417_1995_PuertoRico
FJ024476_1997_Colombia
JF262781_1995_Venezuela
JN559740_1982_Brazil
GU289913_1982_Colombia
KF907503_1953_Senegal
MF004387_1981_Senegal
KM190936_2006_Thailand
OK469359_1980_Kiribati
OK469360_1980_Niue
KY586826_2001_Thailand
KY586823_2002_Thailand
KX812530_1976_Indonesia
OK469361_1990_Singapore
OK469362_1995_Singapore
4-IIA
KU523872_2015_Indonesia
MH382789_2016_PNG
KY921910_2016_Singapore
KU509288_2010_Indonesia
MH051734_2014_Malaysia
JQ822247_2010_China
KC762694_2007_Indonesia
KC762698_2008_Indonesia
LC069810_2015_India
MH888334_2013_Malaysia
JX024758_2010_Singapore
AY618993_2000_Thailand
KC762695_2007_Indonesia
JQ915084_2010_FrenchPolynesia
JQ915090_2009_WallisandFutuna
JQ915082_2009_FrenchPolynesia
JQ915087_2009_NewCaledonia
MH823210_2014_Indonesia
GQ398256_2005_Singapore
KC333651_2012_China
4-IIB
0.67
0.69
0.41
0.76
0.26
0.12
0.73
0.55
0.78
0.71
0.72
1
0.06

Figure S7c

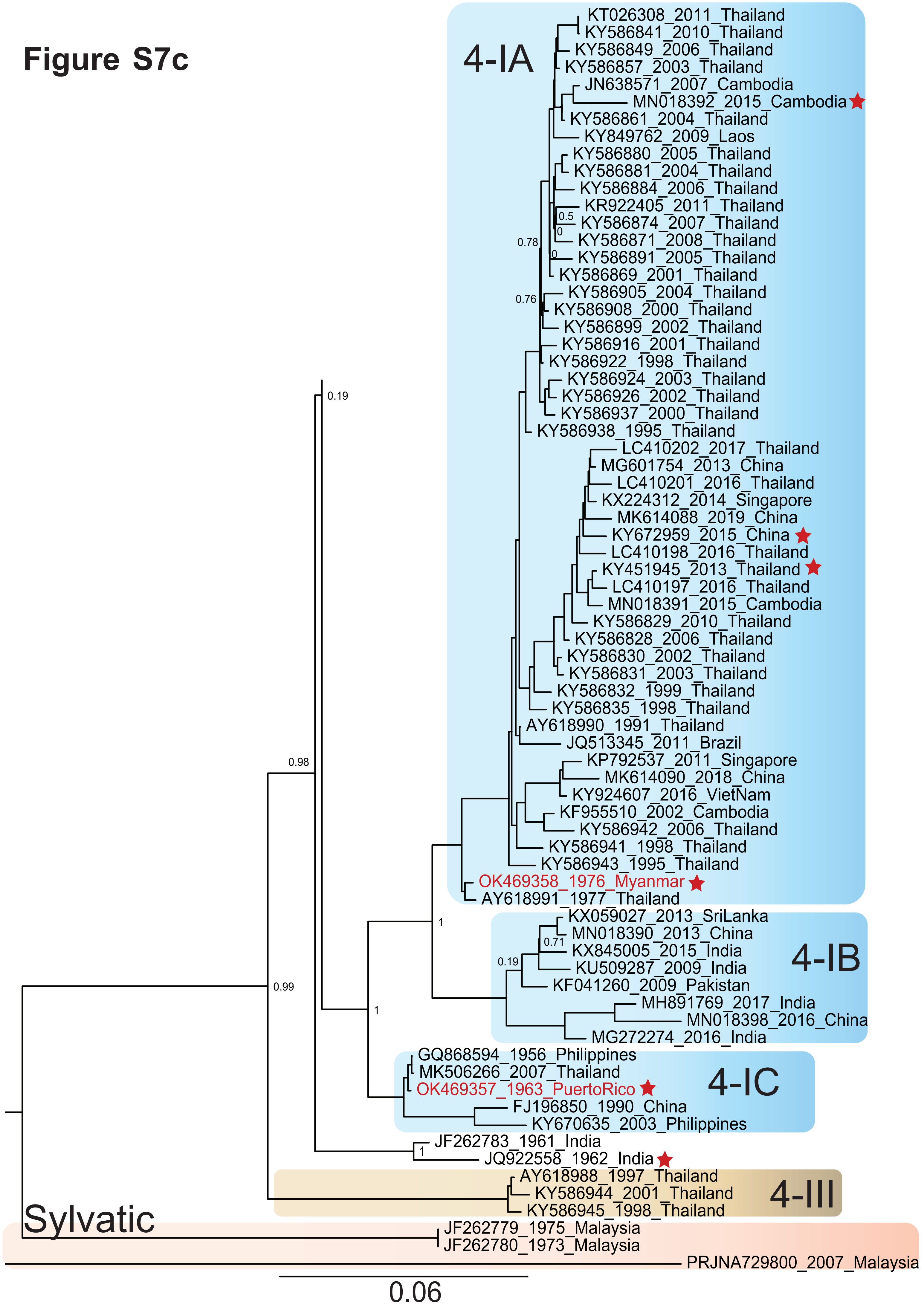
4-IA
KT026308_2011_Thailand
KY586841_2010_Thailand
KY586849_2006_Thailand
KY586857_2003_Thailand
JN638571_2007_Cambodia
MN018392_2015_Cambodia
KY586861_2004_Thailand
KY849762_2009_Laos
KY586880_2005_Thailand
KY586881_2004_Thailand
KY586884_2006_Thailand
KR922405_2011_Thailand
0.5
KY586874_2007_Thailand
0
KY586871_2008_Thailand
0.78
0
KY586891_2005_Thailand
KY586869_2001_Thailand
KY586905_2004_Thailand
0.76
KY586908_2000_Thailand
KY586899_2002_Thailand
KY586916_2001_Thailand
KY586922_1998_Thailand
KY586924_2003_Thailand
KY586926_2002_Thailand
KY586937_2000_Thailand
KY586938_1995_Thailand
0.19
LC410202_2017_Thailand
MG601754_2013_China
LC410201_2016_Thailand
KX224312_2014_Singapore
MK614088_2019_China
KY672959_2015_China
LC410198_2016_Thailand
KY451945_2013_Thailand
LC410197_2016_Thailand
MN018391_2015_Cambodia
KY586829_2010_Thailand
KY586828_2006_Thailand
KY586830_2002_Thailand
KY586831_2003_Thailand
KY586832_1999_Thailand
KY586835_1998_Thailand
AY618990_1991_Thailand
JQ513345_2011_Brazil
0.98
KP792537_2011_Singapore
MK614090_2018_China
KY924607_2016_VietNam
KF955510_2002_Cambodia
KY586942_2006_Thailand
KY586941_1998_Thailand
KY586943_1995_Thailand
OK469358_1976_Myanmar
AY618991_1977_Thailand
KX059027_2013_SriLanka
1
MN018390_2013_China
0.71
4-IB
KX845005_2015_India
0.19
KU509287_2009_India
0.99
KF041260_2009_Pakistan
1
MH891769_2017_India
MN018398_2016_China
MG272274_2016_India
GQ868594_1956_Philippines
MK506266_2007_Thailand
4-IC
OK469357_1963_PuertoRico
FJ196850_1990_China
KY670635_2003_Philippines
JF262783_1961_India
1
JQ922558_1962_India
AY618988_1997_Thailand
KY586944_2001_Thailand
4-III
KY586945_1998_Thailand
Sylvatic
JF262779_1975_Malaysia
JF262780_1973_Malaysia
PRJNA729800_2007_Malaysia
0.06

Supplemental Figure S8: DENV4 E Gene ML Trees. (a) DENV4 E gene ML tree, (b) Genotype 4-II, (c) Genotypes 4-I, 4-III, and sylvatic. aLRT SH-like branch support indicated for the major branches. Support values >0.8 have been eliminated in most instances for clarity. Red circles/font indicate newly sequenced isolates from the QUT Arbovirus collection. Branches under positive selection (see section 3.2.2) are indicated by a red star.

Figure S8a

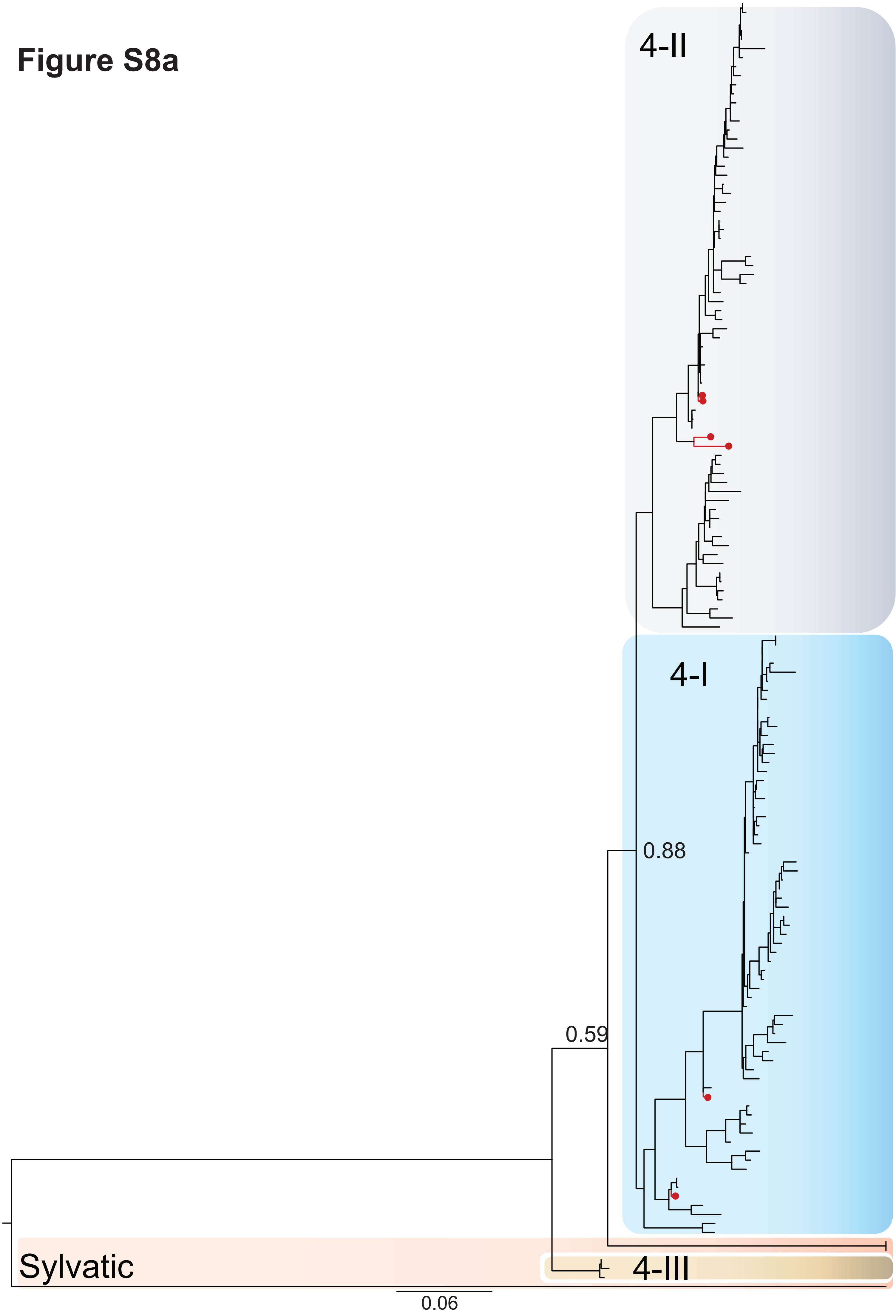

Figure S8b

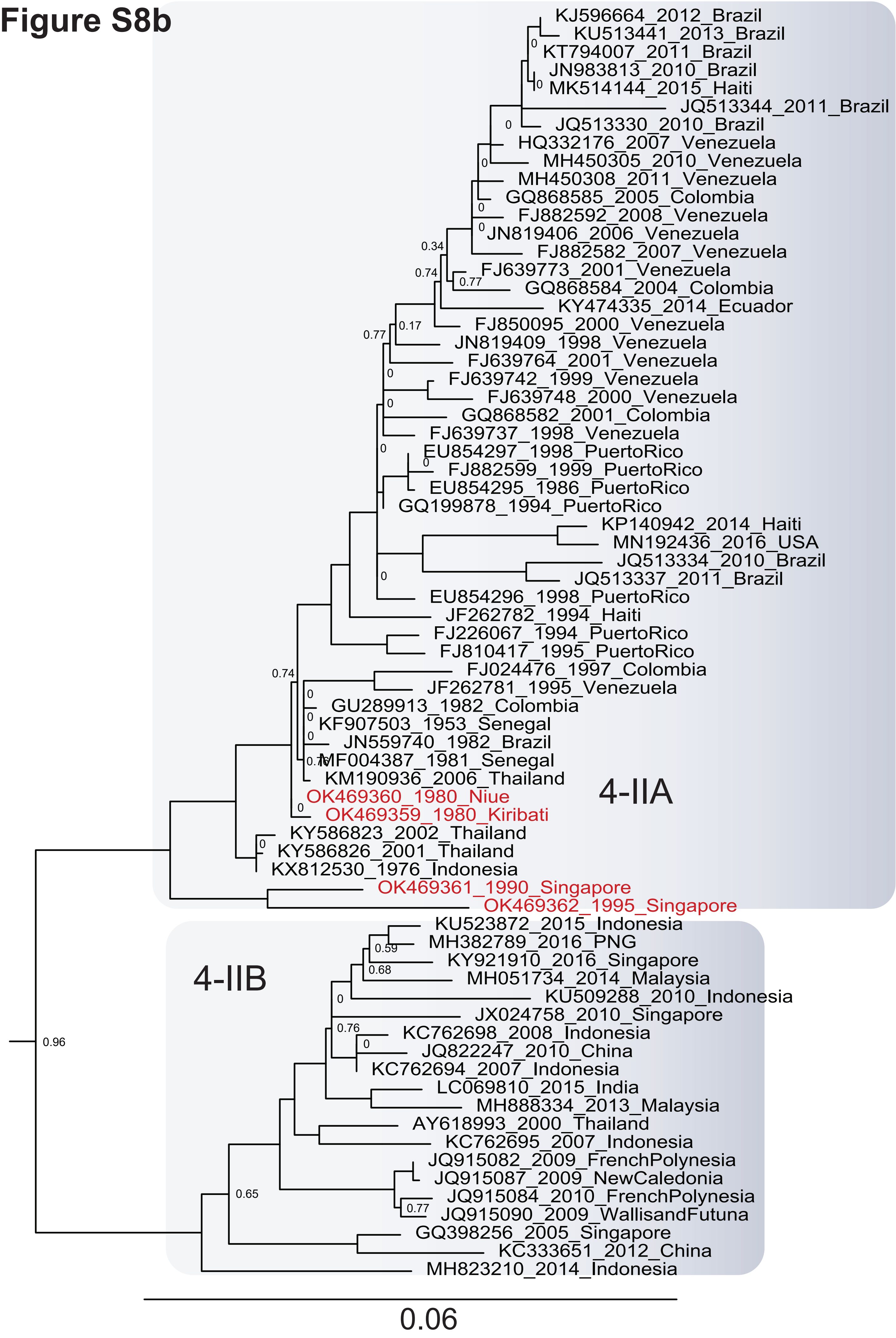
KJ596664_2012_Brazil
KU513441_2013_Brazil
KT794007_2011_Brazil
JN983813_2010_Brazil
MK514144_2015_Haiti
JQ513344_2011_Brazil
JQ513330_2010_Brazil
HQ332176_2007_Venezuela
MH450305_2010_Venezuela
MH450308_2011_Venezuela
GQ868585_2005_Colombia
FJ882592_2008_Venezuela
JN819406_2006_Venezuela
FJ882582_2007_Venezuela
FJ639773_2001_Venezuela
GQ868584_2004_Colombia
KY474335_2014_Ecuador
FJ850095_2000_Venezuela
JN819409_1998_Venezuela
FJ639764_2001_Venezuela
FJ639742_1999_Venezuela
FJ639748_2000_Venezuela
GQ868582_2001_Colombia
FJ639737_1998_Venezuela
EU854297_1998_PuertoRico
FJ882599_1999_PuertoRico
EU854295_1986_PuertoRico
GQ199878_1994_PuertoRico
KP140942_2014_Haiti
MN192436_2016_USA
JQ513334_2010_Brazil
JQ513337_2011_Brazil
EU854296_1998_PuertoRico
JF262782_1994_Haiti
FJ226067_1994_PuertoRico
FJ810417_1995_PuertoRico
FJ024476_1997_Colombia
JF262781_1995_Venezuela
GU289913_1982_Colombia
KF907503_1953_Senegal
JN559740_1982_Brazil
MF004387_1981_Senegal
KM190936_2006_Thailand
OK469360_1980_Niue
OK469359_1980_Kiribati
KY586823_2002_Thailand
KY586826_2001_Thailand
KX812530_1976_Indonesia
OK469361_1990_Singapore
OK469362_1995_Singapore
4-IIA
4-IIB
KU523872_2015_Indonesia
MH382789_2016_PNG
KY921910_2016_Singapore
MH051734_2014_Malaysia
KU509288_2010_Indonesia
JX024758_2010_Singapore
KC762698_2008_Indonesia
JQ822247_2010_China
KC762694_2007_Indonesia
LC069810_2015_India
MH888334_2013_Malaysia
AY618993_2000_Thailand
KC762695_2007_Indonesia
JQ915082_2009_FrenchPolynesia
JQ915087_2009_NewCaledonia
JQ915084_2010_FrenchPolynesia
JQ915090_2009_WallisandFutuna
GQ398256_2005_Singapore
KC333651_2012_China
MH823210_2014_Indonesia
0.96
0.65
0.06

Figure S8c

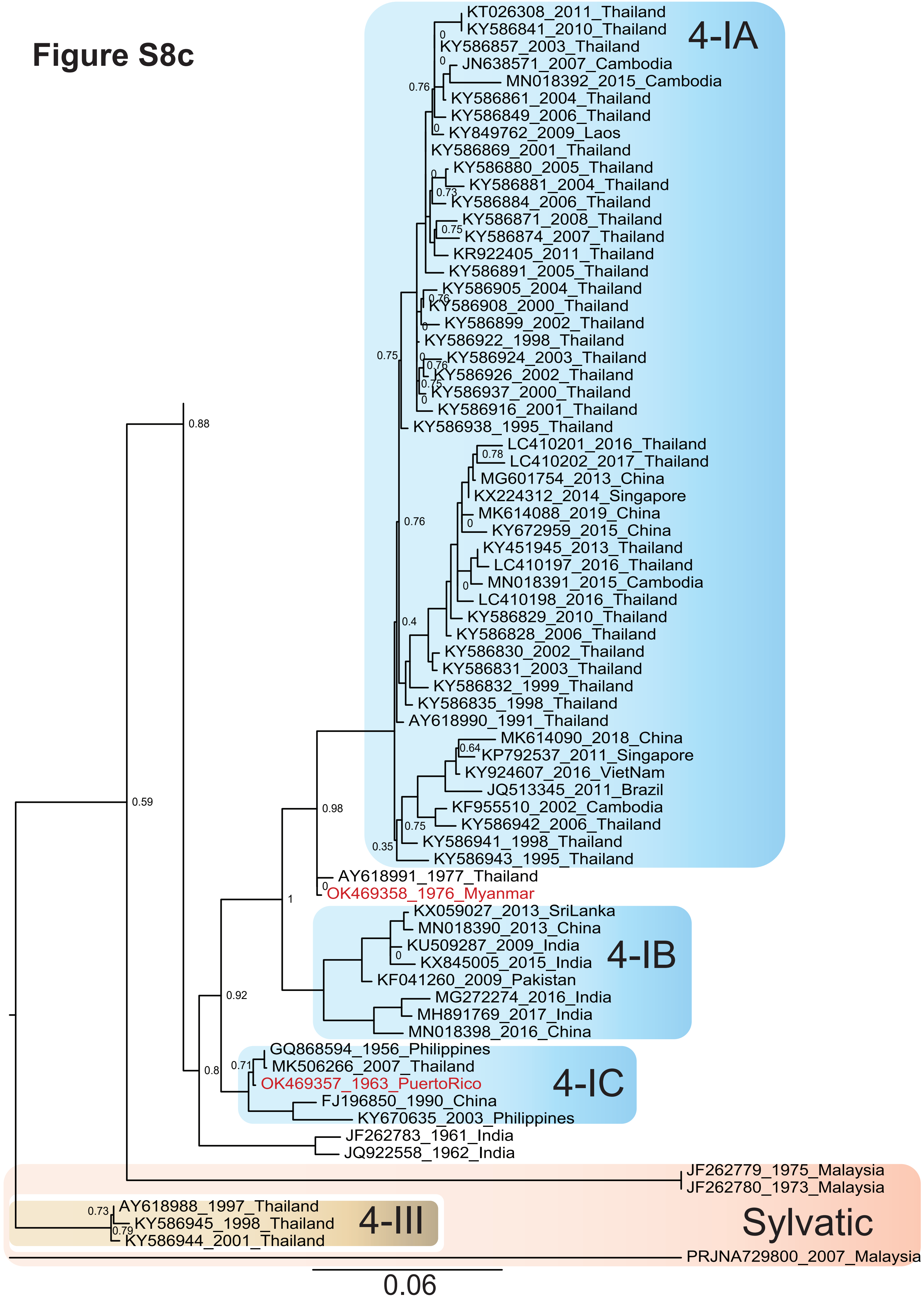
4-IA
KT026308_2011_Thailand
KY586841_2010_Thailand
KY586857_2003_Thailand
JN638571_2007_Cambodia
MN018392_2015_Cambodia
KY586861_2004_Thailand
KY586849_2006_Thailand
KY849762_2009_Laos
KY586869_2001_Thailand
KY586880_2005_Thailand
KY586881_2004_Thailand
KY586884_2006_Thailand
KY586871_2008_Thailand
KY586874_2007_Thailand
KR922405_2011_Thailand
KY586891_2005_Thailand
KY586905_2004_Thailand
KY586908_2000_Thailand
KY586899_2002_Thailand
KY586922_1998_Thailand
KY586924_2003_Thailand
KY586926_2002_Thailand
KY586937_2000_Thailand
KY586916_2001_Thailand
KY586938_1995_Thailand
LC410201_2016_Thailand
LC410202_2017_Thailand
MG601754_2013_China
KX224312_2014_Singapore
MK614088_2019_China
KY672959_2015_China
KY451945_2013_Thailand
LC410197_2016_Thailand
MN018391_2015_Cambodia
LC410198_2016_Thailand
KY586829_2010_Thailand
KY586828_2006_Thailand
KY586830_2002_Thailand
KY586831_2003_Thailand
KY586832_1999_Thailand
KY586835_1998_Thailand
AY618990_1991_Thailand
MK614090_2018_China
KP792537_2011_Singapore
KY924607_2016_VietNam
JQ513345_2011_Brazil
KF955510_2002_Cambodia
KY586942_2006_Thailand
KY586941_1998_Thailand
KY586943_1995_Thailand
AY618991_1977_Thailand
OK469358_1976_Myanmar
4-IB
KX059027_2013_SriLanka
MN018390_2013_China
KU509287_2009_India
KX845005_2015_India
KF041260_2009_Pakistan
MG272274_2016_India
MH891769_2017_India
MN018398_2016_China
4-IC
GQ868594_1956_Philippines
MK506266_2007_Thailand
OK469357_1963_PuertoRico
FJ196850_1990_China
KY670635_2003_Philippines
JF262783_1961_India
JQ922558_1962_India
Sylvatic
JF262779_1975_Malaysia
JF262780_1973_Malaysia
4-III
AY618988_1997_Thailand
KY586945_1998_Thailand
KY586944_2001_Thailand
PRJNA729800_2007_Malaysia
0.06

Supplemental Figure S9: DENV1 Whole Genome Genotypes MCMC Trees. (a) Genotype 1-I, (b) Genotype 1-IV, (c) Genotypes 1-V (isolates incorrectly classified as 1-III indicated by arrows) and sylvatic. Posterior node support indicated for the major branches. Support values >0.8 have been eliminated in most instances for clarity. Red font indicates newly sequenced isolates from the QUT Arbovirus collection. Branches under positive selection (see section 3.2.2) are indicated by a red star. Scale axis is representative of calendar years.

Figure S9a

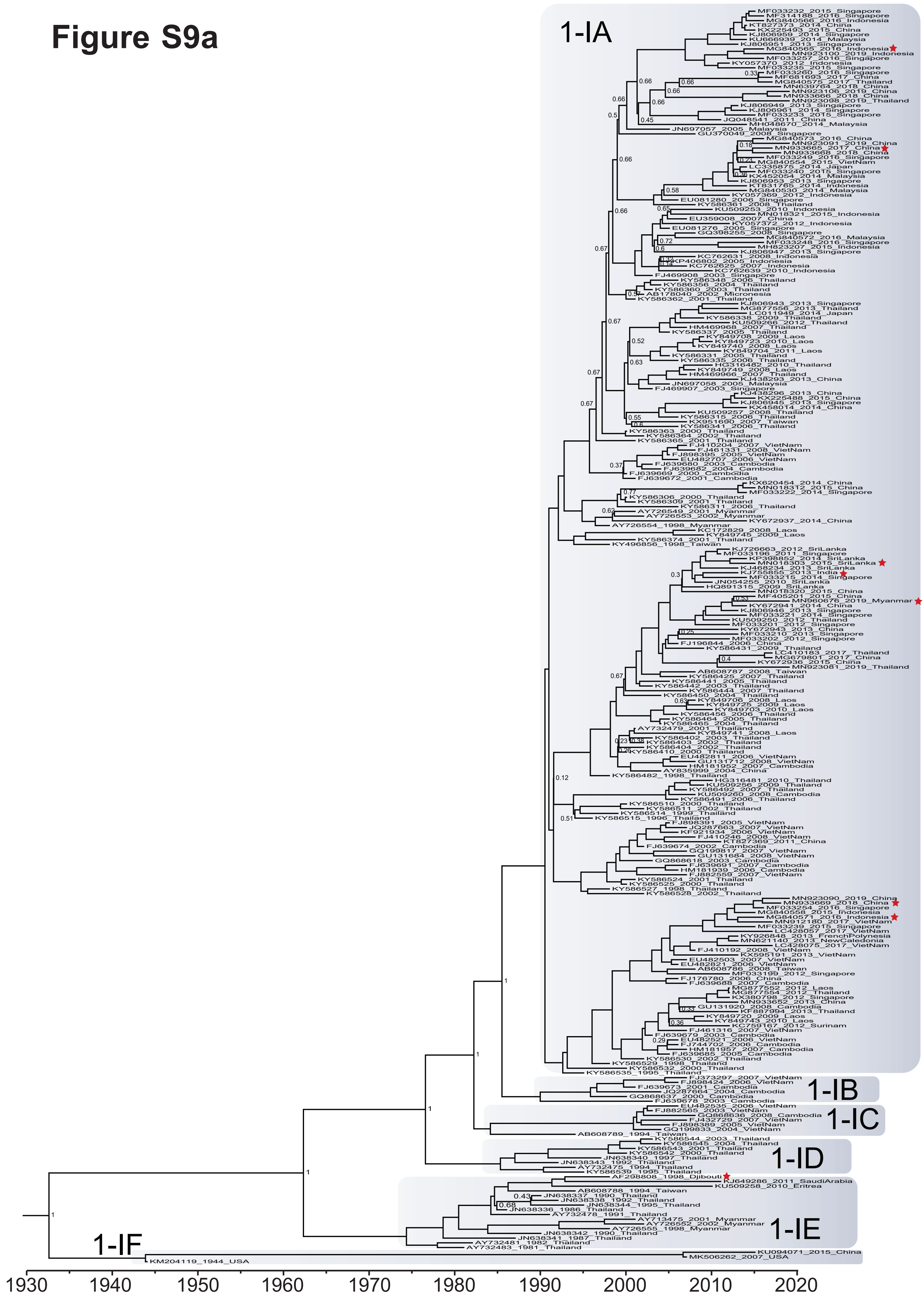

1-IA
1-IB
1-IC
1-ID
1-IE
1-IF
1930
1940
1950
1960
1970
1980
1990
2000
2010
2020

Figure S9b

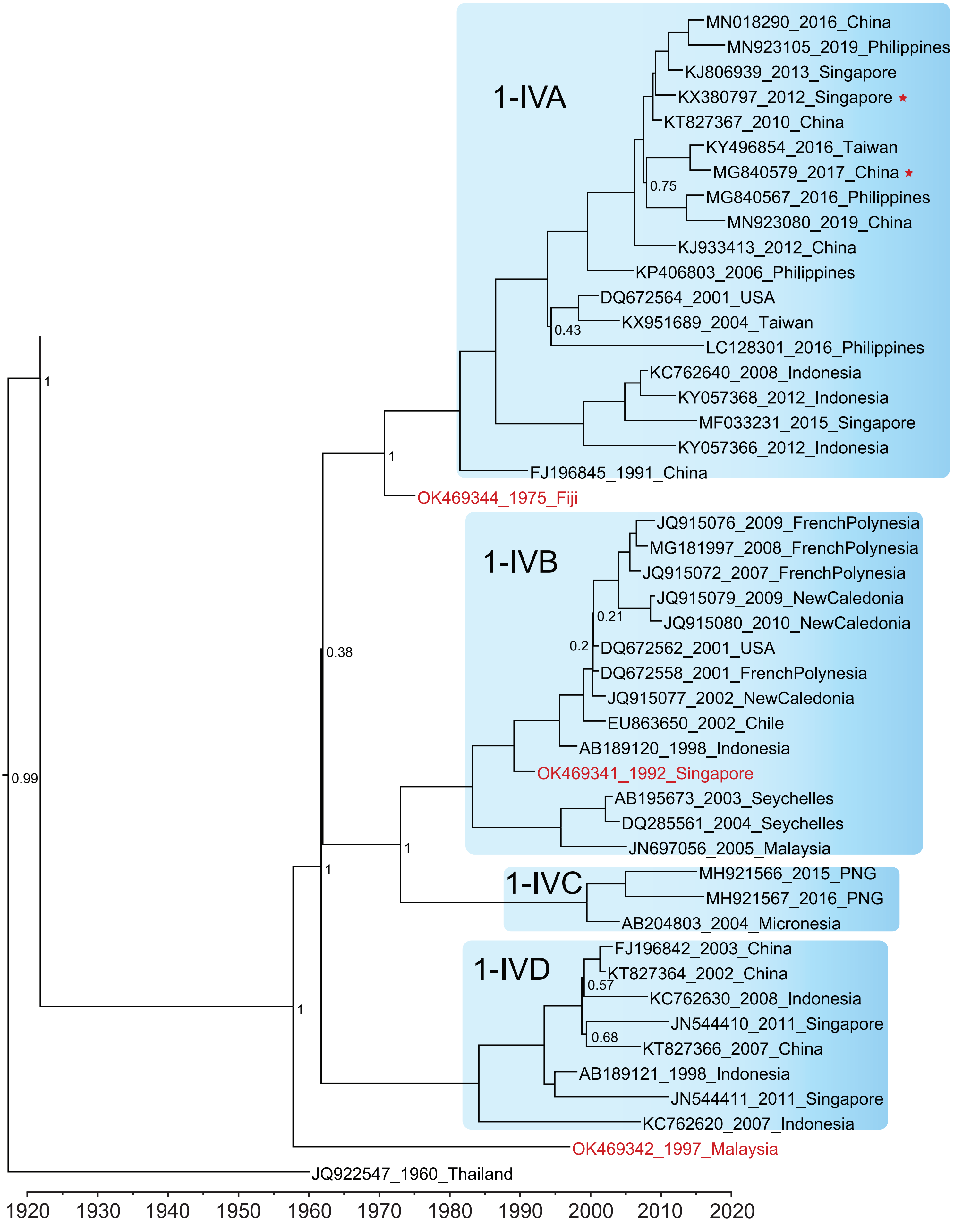
1-IVA
MN018290_2016_China
MN923105_2019_Philippines
KJ806939_2013_Singapore
KX380797_2012_Singapore ★
KT827367_2010_China
KY496854_2016_Taiwan
MG840579_2017_China ★
0.75
MG840567_2016_Philippines
MN923080_2019_China
KJ933413_2012_China
KP406803_2006_Philippines
DQ672564_2001_USA
KX951689_2004_Taiwan
0.43
LC128301_2016_Philippines
KC762640_2008_Indonesia
KY057368_2012_Indonesia
MF033231_2015_Singapore
KY057366_2012_Indonesia
FJ196845_1991_China
1
OK469344_1975_Fiji
1-IVB
JQ915076_2009_FrenchPolynesia
MG181997_2008_FrenchPolynesia
JQ915072_2007_FrenchPolynesia
JQ915079_2009_NewCaledonia
0.21
JQ915080_2010_NewCaledonia
0.2
DQ672562_2001_USA
0.38
DQ672558_2001_FrenchPolynesia
JQ915077_2002_NewCaledonia
EU863650_2002_Chile
AB189120_1998_Indonesia
OK469341_1992_Singapore
AB195673_2003_Seychelles
DQ285561_2004_Seychelles
1
JN697056_2005_Malaysia
1-IVC
MH921566_2015_PNG
1
MH921567_2016_PNG
AB204803_2004_Micronesia
1-IVD
FJ196842_2003_China
KT827364_2002_China
0.57
KC762630_2008_Indonesia
1
JN544410_2011_Singapore
0.68
KT827366_2007_China
AB189121_1998_Indonesia
JN544411_2011_Singapore
KC762620_2007_Indonesia
OK469342_1997_Malaysia
JQ922547_1960_Thailand
0.99
1920
1930
1940
1950
1960
1970
1980
1990
2000
2010
2020

Figure S9c

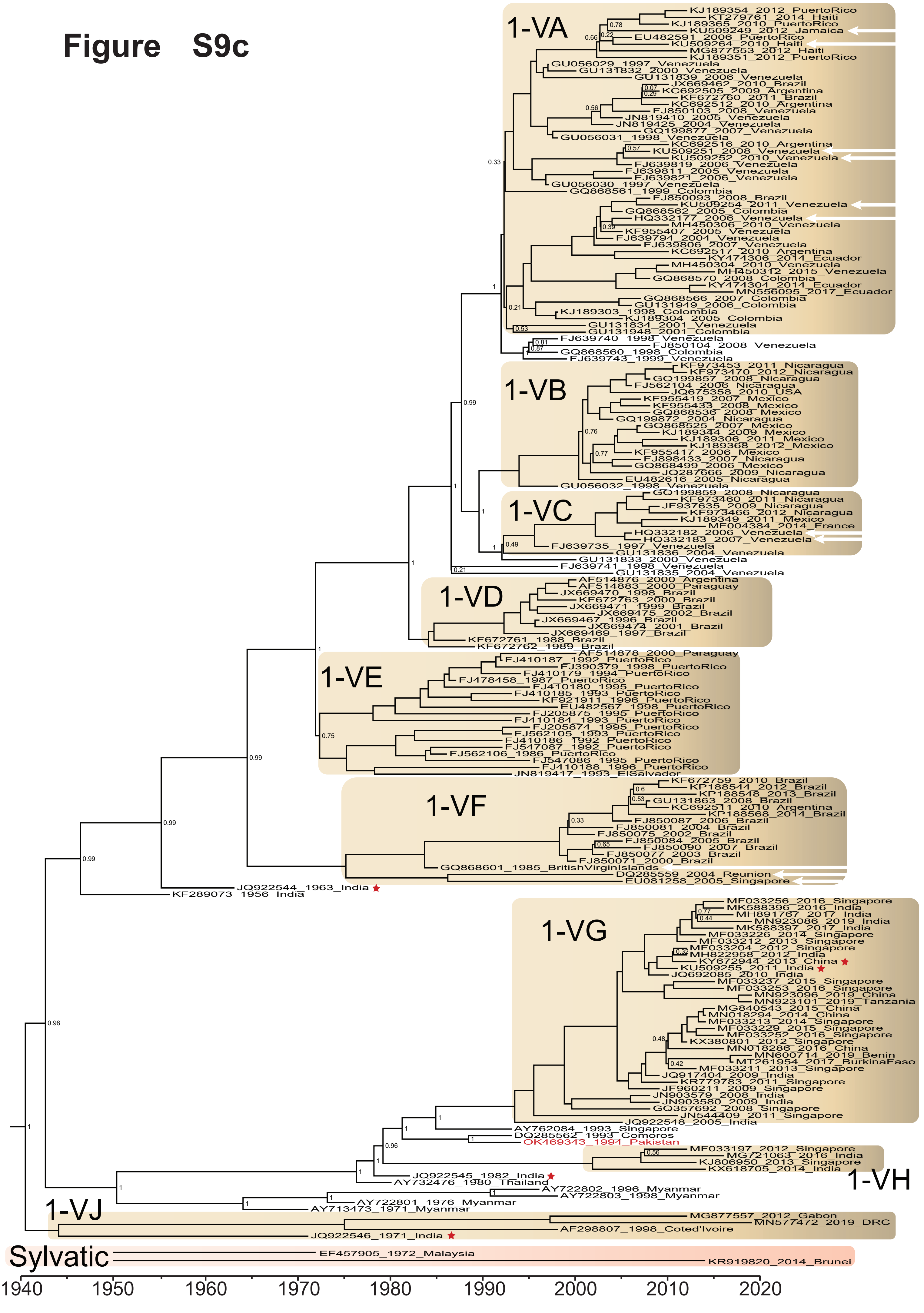

1-VA
KJ189354_2012_PuertoRico
KT279761_2014_Haiti
KJ189365_2010_PuertoRico
KU509249_2012_Jamaica
EU482591_2006_PuertoRico
KU509264_2010_Haiti
MG877553_2012_Haiti
KJ189351_2012_PuertoRico
GU056029_1997_Venezuela
GU131832_2000_Venezuela
GU131839_2006_Venezuela
JX669462_2010_Brazil
KC692505_2009_Argentina
KF672760_2011_Brazil
KC692512_2010_Argentina
FJ850103_2008_Venezuela
JN819410_2005_Venezuela
JN819425_2004_Venezuela
GQ199877_2007_Venezuela
GU056031_1998_Venezuela
KC692516_2010_Argentina
KU509251_2008_Venezuela
KU509252_2010_Venezuela
FJ639819_2006_Venezuela
FJ639811_2005_Venezuela
FJ639821_2006_Venezuela
GU056030_1997_Venezuela
GQ868561_1999_Colombia
FJ850093_2008_Brazil
KU509254_2011_Venezuela
GQ868562_2005_Colombia
HQ332177_2006_Venezuela
MH450306_2010_Venezuela
KF955407_2005_Venezuela
FJ639794_2004_Venezuela
FJ639806_2007_Venezuela
KC692517_2010_Argentina
KY474306_2014_Ecuador
MH450304_2010_Venezuela
MH450312_2015_Venezuela
GQ868570_2008_Colombia
KY474304_2014_Ecuador
MN556095_2017_Ecuador
GQ868566_2007_Colombia
GU131949_2006_Colombia
KJ189303_1998_Colombia
KJ189304_2005_Colombia
GU131834_2001_Venezuela
GU131948_2001_Colombia
FJ639740_1998_Venezuela
FJ850104_2008_Venezuela
GQ868560_1998_Colombia
FJ639743_1999_Venezuela
1-VB
KF973453_2011_Nicaragua
KF973470_2012_Nicaragua
GQ199857_2008_Nicaragua
FJ562104_2006_Nicaragua
JQ675358_2010_USA
KF955419_2007_Mexico
KF955433_2008_Mexico
GQ868536_2008_Mexico
GQ199872_2004_Nicaragua
GQ868525_2007_Mexico
KJ189344_2009_Mexico
KJ189306_2011_Mexico
KJ189368_2012_Mexico
KF955417_2006_Mexico
FJ898433_2007_Nicaragua
GQ868499_2006_Mexico
JQ287666_2009_Nicaragua
EU482616_2005_Nicaragua
GU056032_1998_Venezuela
1-VC
GQ199859_2008_Nicaragua
KF973460_2011_Nicaragua
JF937635_2009_Nicaragua
KF973466_2012_Nicaragua
KJ189349_2011_Mexico
MF004384_2014_France
HQ332182_2006_Venezuela
HQ332183_2007_Venezuela
FJ639735_1997_Venezuela
GU131836_2004_Venezuela
GU131833_2000_Venezuela
FJ639741_1998_Venezuela
GU131835_2004_Venezuela
1-VD
AF514876_2000_Argentina
AF514883_2000_Paraguay
JX669470_1998_Brazil
KF672763_2000_Brazil
JX669471_1999_Brazil
JX669475_2002_Brazil
JX669467_1996_Brazil
JX669474_2001_Brazil
JX669469_1997_Brazil
KF672761_1988_Brazil
KF672762_1989_Brazil
1-VE
AF514878_2000_Paraguay
FJ410187_1992_PuertoRico
FJ390379_1998_PuertoRico
FJ410179_1994_PuertoRico
FJ478458_1987_PuertoRico
FJ410180_1995_PuertoRico
FJ410185_1993_PuertoRico
KF921911_1996_PuertoRico
EU482567_1998_PuertoRico
FJ205875_1995_PuertoRico
FJ410184_1993_PuertoRico
FJ205874_1995_PuertoRico
FJ562105_1993_PuertoRico
FJ410186_1992_PuertoRico
FJ547087_1992_PuertoRico
FJ562106_1986_PuertoRico
FJ547086_1995_PuertoRico
FJ410188_1996_PuertoRico
JN819417_1993_ElSalvador
1-VF
KF672759_2010_Brazil
KP188544_2012_Brazil
KP188548_2013_Brazil
GU131863_2008_Brazil
KC692511_2010_Argentina
KP188568_2014_Brazil
FJ850087_2006_Brazil
FJ850081_2004_Brazil
FJ850075_2002_Brazil
FJ850084_2005_Brazil
FJ850090_2007_Brazil
FJ850077_2003_Brazil
FJ850071_2000_Brazil
GQ868601_1985_BritishVirginIslands
DQ285559_2004_Reunion
EU081258_2005_Singapore
JQ922544_1963_India
KF289073_1956_India
1-VG
MF033256_2016_Singapore
MK588396_2016_India
MH891767_2017_India
MN923086_2019_India
MK588397_2017_India
MF033226_2014_Singapore
MF033212_2013_Singapore
MF033204_2012_Singapore
MH822958_2012_India
KY672944_2013_China
KU509255_2011_India
JQ692085_2010_India
MF033237_2015_Singapore
MF033253_2016_Singapore
MN923096_2019_China
MN923101_2019_Tanzania
MG840543_2015_China
MN018294_2014_China
MF033213_2014_Singapore
MF033229_2015_Singapore
MF033252_2016_Singapore
KX380801_2012_Singapore
MN018286_2016_China
MN600714_2019_Benin
MT261954_2017_BurkinaFaso
MF033211_2013_Singapore
JQ917404_2009_India
KR779783_2011_Singapore
JF960211_2009_Singapore
JN903579_2008_India
JN903580_2009_India
GQ357692_2008_Singapore
JN544409_2011_Singapore
JQ922548_2005_India
AY762084_1993_Singapore
DQ285562_1993_Comoros
OK469343_1994_Pakistan
MF033197_2012_Singapore
MG721063_2016_India
KJ806950_2013_Singapore
KX618705_2014_India
1-VH
JQ922545_1982_India
AY732476_1980_Thailand
AY722802_1996_Myanmar
AY722803_1998_Myanmar
AY722801_1976_Myanmar
AY713473_1971_Myanmar
1-VJ
MG877557_2012_Gabon
MN577472_2019_DRC
AF298807_1998_Coted'Ivoire
JQ922546_1971_India
Sylvatic
EF457905_1972_Malaysia
KR919820_2014_Brunei
1940
1950
1960
1970
1980
1990
2000
2010
2020

Supplemental Figure S10: DENV1 E Gene MCMC Trees. (a) DENV1 E gene MCMC tree, (b) Genotype 1-I, (c) Genotype 1-IV, (d) Genotypes 1-V (isolates incorrectly classified as 1-III indicated by arrows) and sylvatic. Posterior node support indicated for the major branches. Support values >0.8 have been eliminated in most instances for clarity. Red circles/font indicates newly sequenced isolates from the QUT Arbovirus collection. Branches under positive selection (see section 3.2.2) are indicated by a red star. Scale axis is representative of calendar years.

Figure S10a

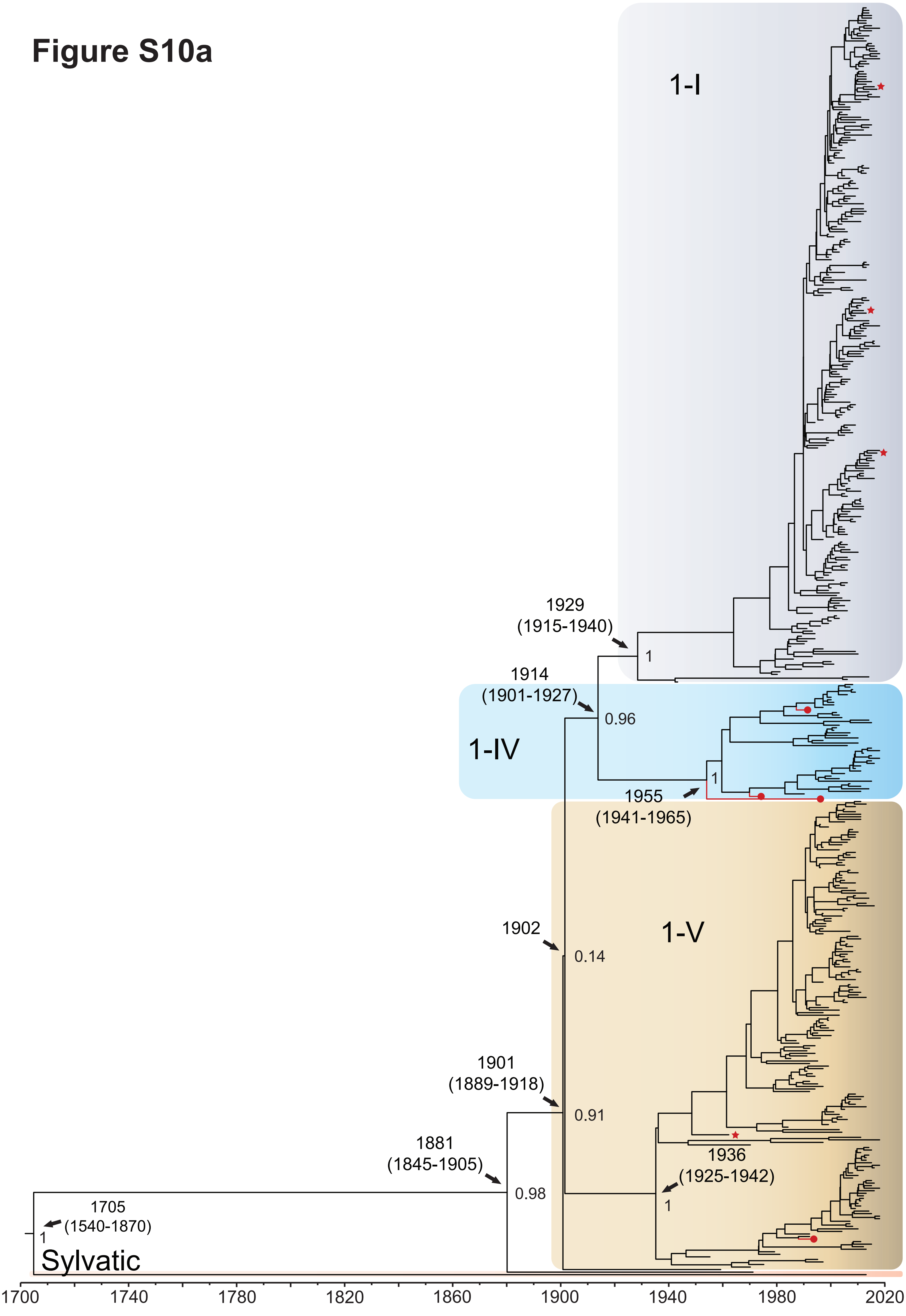

1-I
1929
(1915-1940)
1
1914
(1901-1927)
0.96
1-IV
1
1955
(1941-1965)
1-V
1902
0.14
1901
(1889-1918)
0.91
1936
(1925-1942)
1
1881
(1845-1905)
0.98
1705
(1540-1870)
1
Sylvatic
1700
1740
1780
1820
1860
1900
1940
1980
2020

Figure S10b

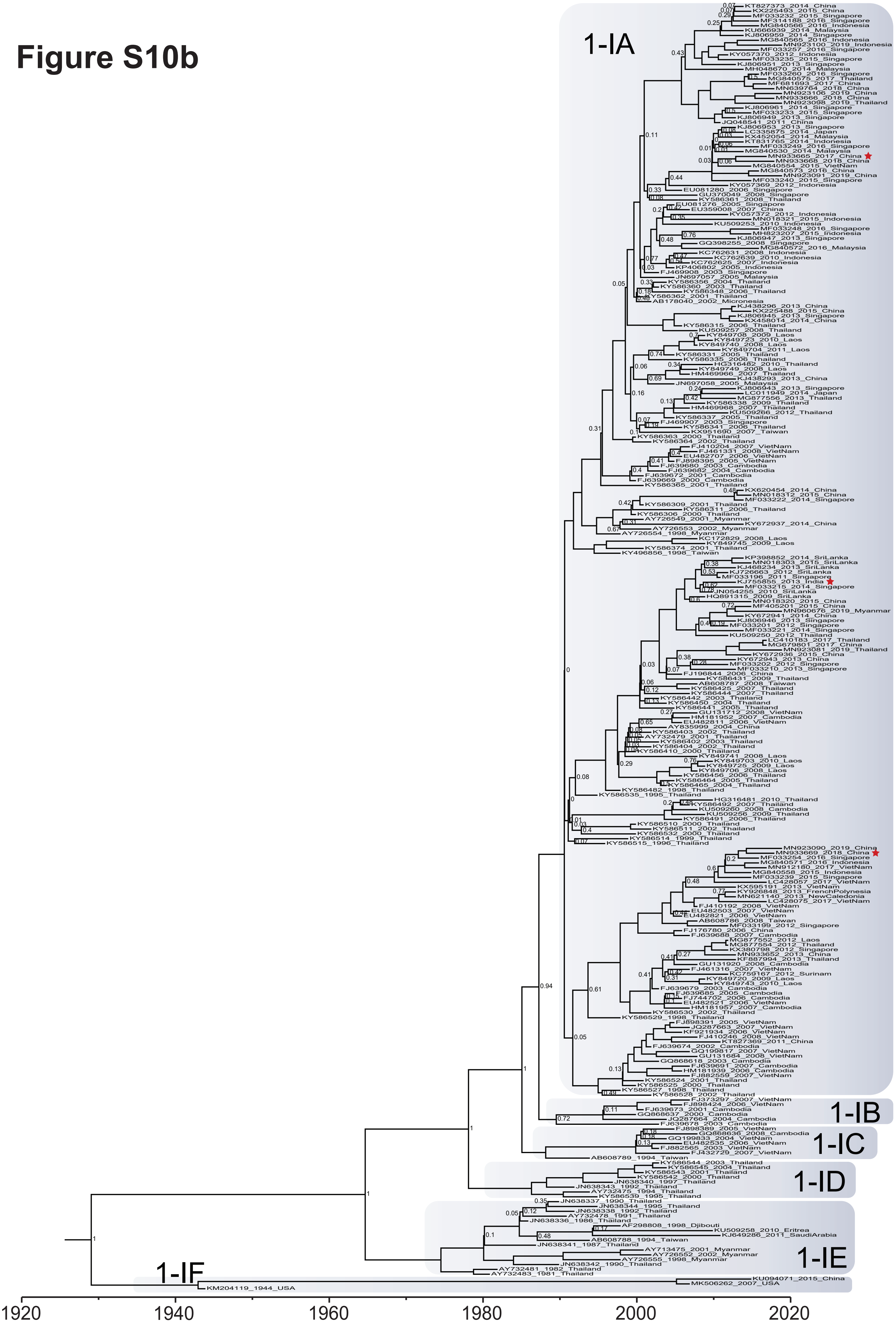

Figure S10c

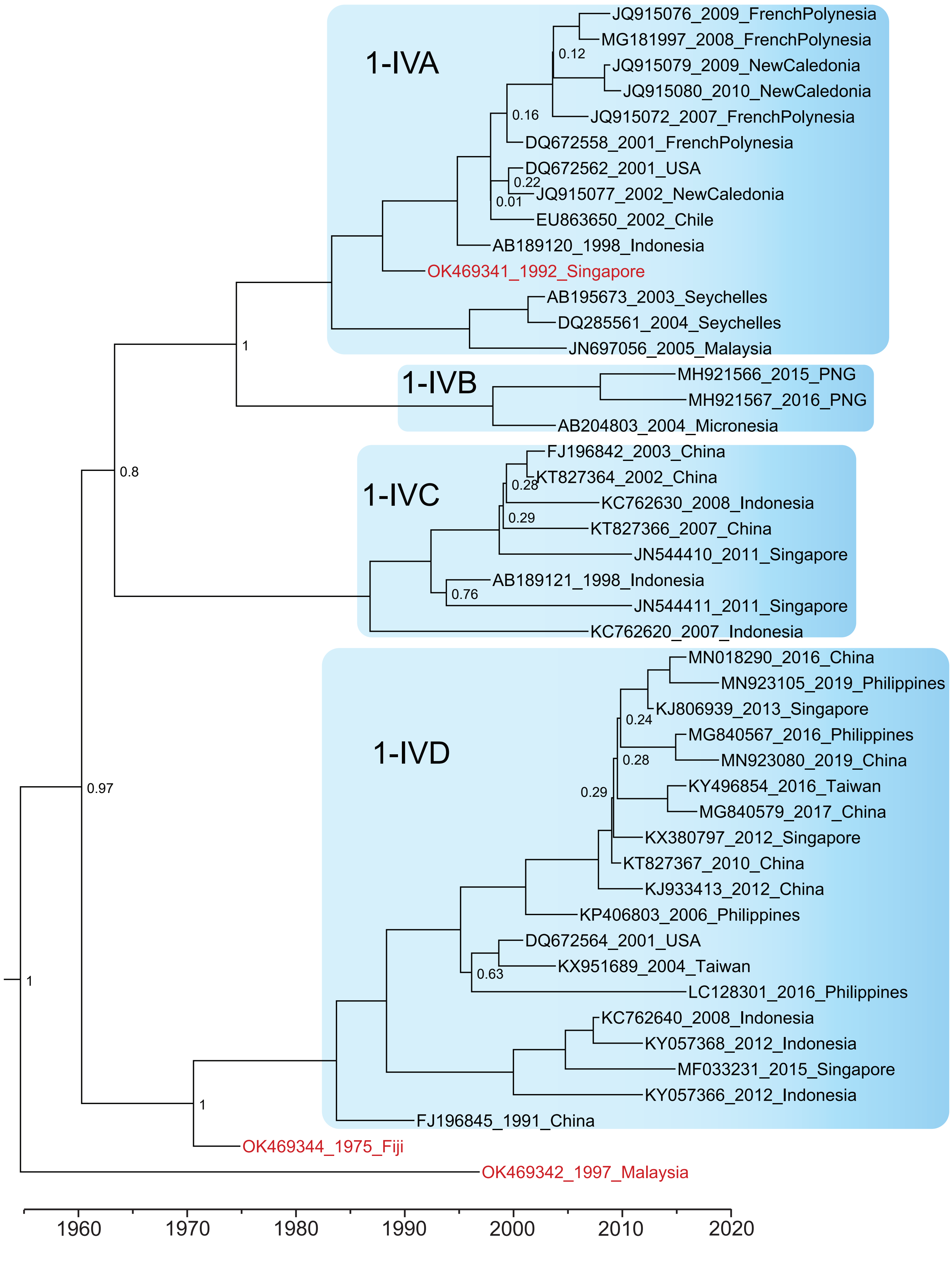

1-IVA
JQ915076_2009_FrenchPolynesia
MG181997_2008_FrenchPolynesia
0.12
JQ915079_2009_NewCaledonia
JQ915080_2010_NewCaledonia
0.16
JQ915072_2007_FrenchPolynesia
DQ672558_2001_FrenchPolynesia
DQ672562_2001_USA
0.22
JQ915077_2002_NewCaledonia
0.01
EU863650_2002_Chile
AB189120_1998_Indonesia
OK469341_1992_Singapore
AB195673_2003_Seychelles
DQ285561_2004_Seychelles
JN697056_2005_Malaysia
1
1-IVB
MH921566_2015_PNG
MH921567_2016_PNG
AB204803_2004_Micronesia
0.8
1-IVC
FJ196842_2003_China
KT827364_2002_China
0.28
KC762630_2008_Indonesia
0.29
KT827366_2007_China
JN544410_2011_Singapore
AB189121_1998_Indonesia
0.76
JN544411_2011_Singapore
KC762620_2007_Indonesia
1-IVD
MN018290_2016_China
MN923105_2019_Philippines
KJ806939_2013_Singapore
0.24
MG840567_2016_Philippines
0.28
MN923080_2019_China
0.29
KY496854_2016_Taiwan
MG840579_2017_China
KX380797_2012_Singapore
KT827367_2010_China
KJ933413_2012_China
KP406803_2006_Philippines
DQ672564_2001_USA
KX951689_2004_Taiwan
0.63
LC128301_2016_Philippines
KC762640_2008_Indonesia
KY057368_2012_Indonesia
MF033231_2015_Singapore
KY057366_2012_Indonesia
FJ196845_1991_China
0.97
1
1
OK469344_1975_Fiji
OK469342_1997_Malaysia
1960
1970
1980
1990
2000
2010
2020

Figure S10d

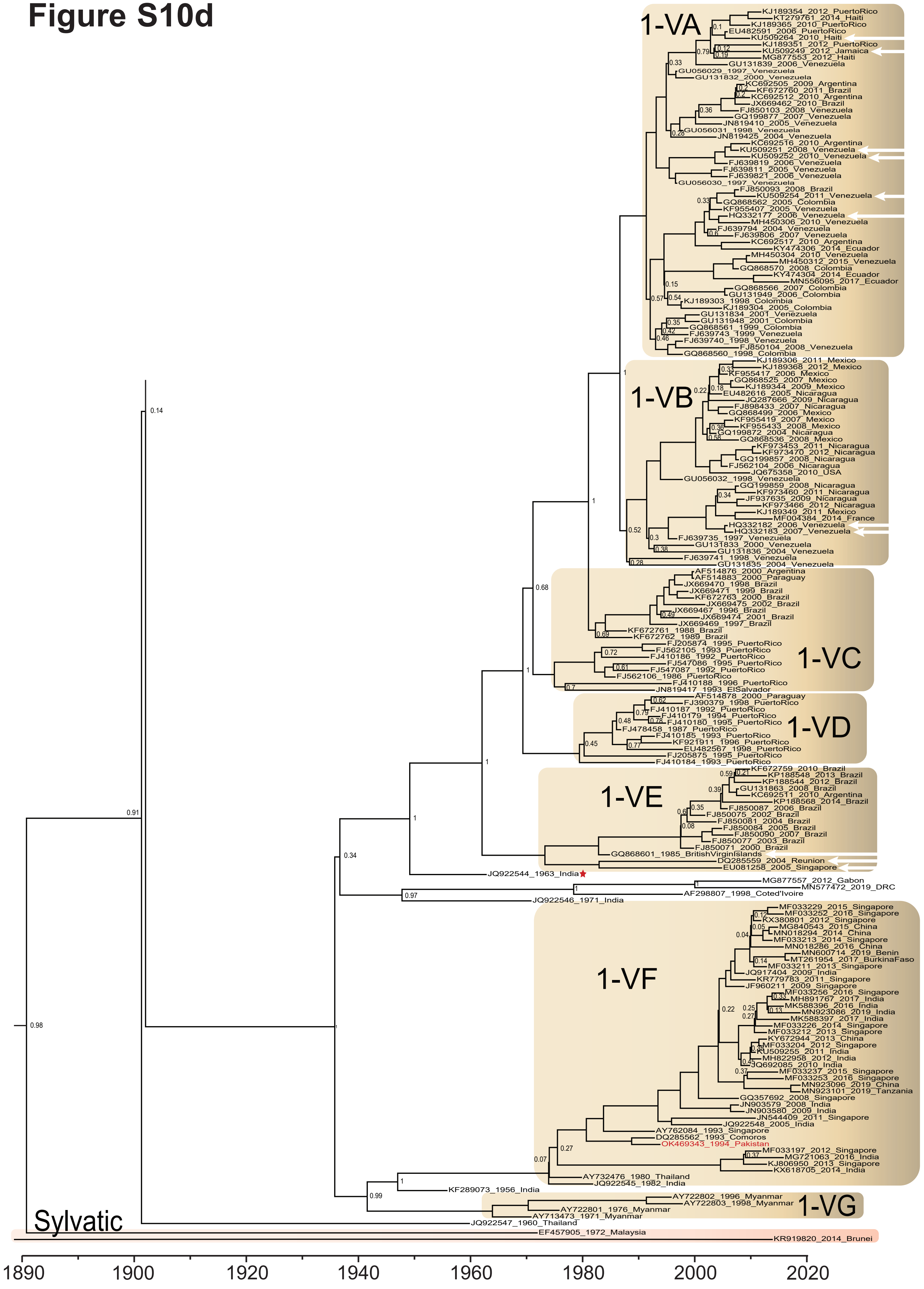

1-VA
KJ189354_2012_PuertoRico
KT279761_2014_Haiti
KJ189365_2010_PuertoRico
EU482591_2006_PuertoRico
KU509264_2010_Haiti
KJ189351_2012_PuertoRico
KU509249_2012_Jamaica
MG877553_2012_Haiti
GU131839_2006_Venezuela
GU056029_1997_Venezuela
GU131832_2000_Venezuela
KC692505_2009_Argentina
KF672760_2011_Brazil
KC692512_2010_Argentina
JX669462_2010_Brazil
FJ850103_2008_Venezuela
GQ199877_2007_Venezuela
JN819410_2005_Venezuela
GU056031_1998_Venezuela
JN819425_2004_Venezuela
KC692516_2010_Argentina
KU509251_2008_Venezuela
KU509252_2010_Venezuela
FJ639819_2006_Venezuela
FJ639811_2005_Venezuela
FJ639821_2006_Venezuela
GU056030_1997_Venezuela
FJ850093_2008_Brazil
KU509254_2011_Venezuela
GQ868562_2005_Colombia
KF955407_2005_Venezuela
HQ332177_2006_Venezuela
MH450306_2010_Venezuela
FJ639794_2004_Venezuela
FJ639806_2007_Venezuela
KC692517_2010_Argentina
KY474306_2014_Ecuador
MH450304_2010_Venezuela
MH450312_2015_Venezuela
GQ868570_2008_Colombia
KY474304_2014_Ecuador
MN556095_2017_Ecuador
GQ868566_2007_Colombia
GU131949_2006_Colombia
KJ189303_1998_Colombia
KJ189304_2005_Colombia
GU131834_2001_Venezuela
GU131948_2001_Colombia
GQ868561_1999_Colombia
FJ639743_1999_Venezuela
FJ639740_1998_Venezuela
FJ850104_2008_Venezuela
GQ868560_1998_Colombia
1-VB
KJ189306_2011_Mexico
KJ189368_2012_Mexico
KF955417_2006_Mexico
GQ868525_2007_Mexico
KJ189344_2009_Mexico
EU482616_2005_Nicaragua
JQ287666_2009_Nicaragua
FJ898433_2007_Nicaragua
GQ868499_2006_Mexico
KF955419_2007_Mexico
KF955433_2008_Mexico
GQ199872_2004_Nicaragua
GQ868536_2008_Mexico
KF973453_2011_Nicaragua
KF973470_2012_Nicaragua
GQ199857_2008_Nicaragua
FJ562104_2006_Nicaragua
JQ675358_2010_USA
GU056032_1998_Venezuela
GQ199859_2008_Nicaragua
KF973460_2011_Nicaragua
JF937635_2009_Nicaragua
KF973466_2012_Nicaragua
KJ189349_2011_Mexico
MF004384_2014_France
HQ332182_2006_Venezuela
HQ332183_2007_Venezuela
FJ639735_1997_Venezuela
GU131833_2000_Venezuela
GU131836_2004_Venezuela
FJ639741_1998_Venezuela
GU131835_2004_Venezuela
AF514876_2000_Argentina
AF514883_2000_Paraguay
JX669470_1998_Brazil
JX669471_1999_Brazil
KF672763_2000_Brazil
JX669475_2002_Brazil
JX669467_1996_Brazil
JX669474_2001_Brazil
JX669469_1997_Brazil
KF672761_1988_Brazil
KF672762_1989_Brazil
FJ205874_1995_PuertoRico
FJ562105_1993_PuertoRico
FJ410186_1992_PuertoRico
FJ547086_1995_PuertoRico
FJ547087_1992_PuertoRico
FJ562106_1986_PuertoRico
FJ410188_1996_PuertoRico
JN819417_1993_ElSalvador
1-VC
AF514878_2000_Paraguay
FJ390379_1998_PuertoRico
FJ410187_1992_PuertoRico
FJ410179_1994_PuertoRico
FJ410180_1995_PuertoRico
FJ478458_1987_PuertoRico
FJ410185_1993_PuertoRico
KF921911_1996_PuertoRico
EU482567_1998_PuertoRico
FJ205875_1995_PuertoRico
FJ410184_1993_PuertoRico
1-VD
1-VE
KF672759_2010_Brazil
KP188548_2013_Brazil
KP188544_2012_Brazil
GU131863_2008_Brazil
KC692511_2010_Argentina
KP188568_2014_Brazil
FJ850087_2006_Brazil
FJ850075_2002_Brazil
FJ850081_2004_Brazil
FJ850084_2005_Brazil
FJ850090_2007_Brazil
FJ850077_2003_Brazil
FJ850071_2000_Brazil
GQ868601_1985_BritishVirginIslands
DQ285559_2004_Reunion
EU081258_2005_Singapore
JQ922544_1963_India
MG877557_2012_Gabon
MN577472_2019_DRC
AF298807_1998_Coted'Ivoire
JQ922546_1971_India
1-VF
MF033229_2015_Singapore
MF033252_2016_Singapore
KX380801_2012_Singapore
MG840543_2015_China
MN018294_2014_China
MF033213_2014_Singapore
MN018286_2016_China
MN600714_2019_Benin
MT261954_2017_BurkinaFaso
MF033211_2013_Singapore
JQ917404_2009_India
KR779783_2011_Singapore
JF960211_2009_Singapore
MF033256_2016_Singapore
MH891767_2017_India
MK588396_2016_India
MN923086_2019_India
MK588397_2017_India
MF033226_2014_Singapore
MF033212_2013_Singapore
KY672944_2013_China
MF033204_2012_Singapore
KU509255_2011_India
MH822958_2012_India
JQ692085_2010_India
MF033237_2015_Singapore
MF033253_2016_Singapore
MN923096_2019_China
MN923101_2019_Tanzania
GQ357692_2008_Singapore
JN903579_2008_India
JN903580_2009_India
JN544409_2011_Singapore
JQ922548_2005_India
AY762084_1993_Singapore
DQ285562_1993_Comoros
OK469343_1994_Pakistan
MF033197_2012_Singapore
MG721063_2016_India
KJ806950_2013_Singapore
KX618705_2014_India
AY732476_1980_Thailand
JQ922545_1982_India
KF289073_1956_India
AY722802_1996_Myanmar
AY722803_1998_Myanmar
AY722801_1976_Myanmar
AY713473_1971_Myanmar
1-VG
JQ922547_1960_Thailand
Sylvatic
EF457905_1972_Malaysia
KR919820_2014_Brunei
1890
1900
1920
1940
1960
1980
2000
2020

Supplemental Figure S11: DENV2 Whole Genome Genotypes MCMC Trees. (a) Asian American and Asian II genotypes, (b) Asian I genotype, (c) Cosmopolitan genotype, (d) American and sylvatic genotypes. Posterior node support indicated for the major branches. Support values >0.8 have been eliminated in most instances for clarity. Red font indicates newly sequenced isolates from the QUT Arbovirus collection. Branches under positive selection (see section 3.2.2) are indicated by a red star. Scale axis is representative of calendar years.

Figure S11a

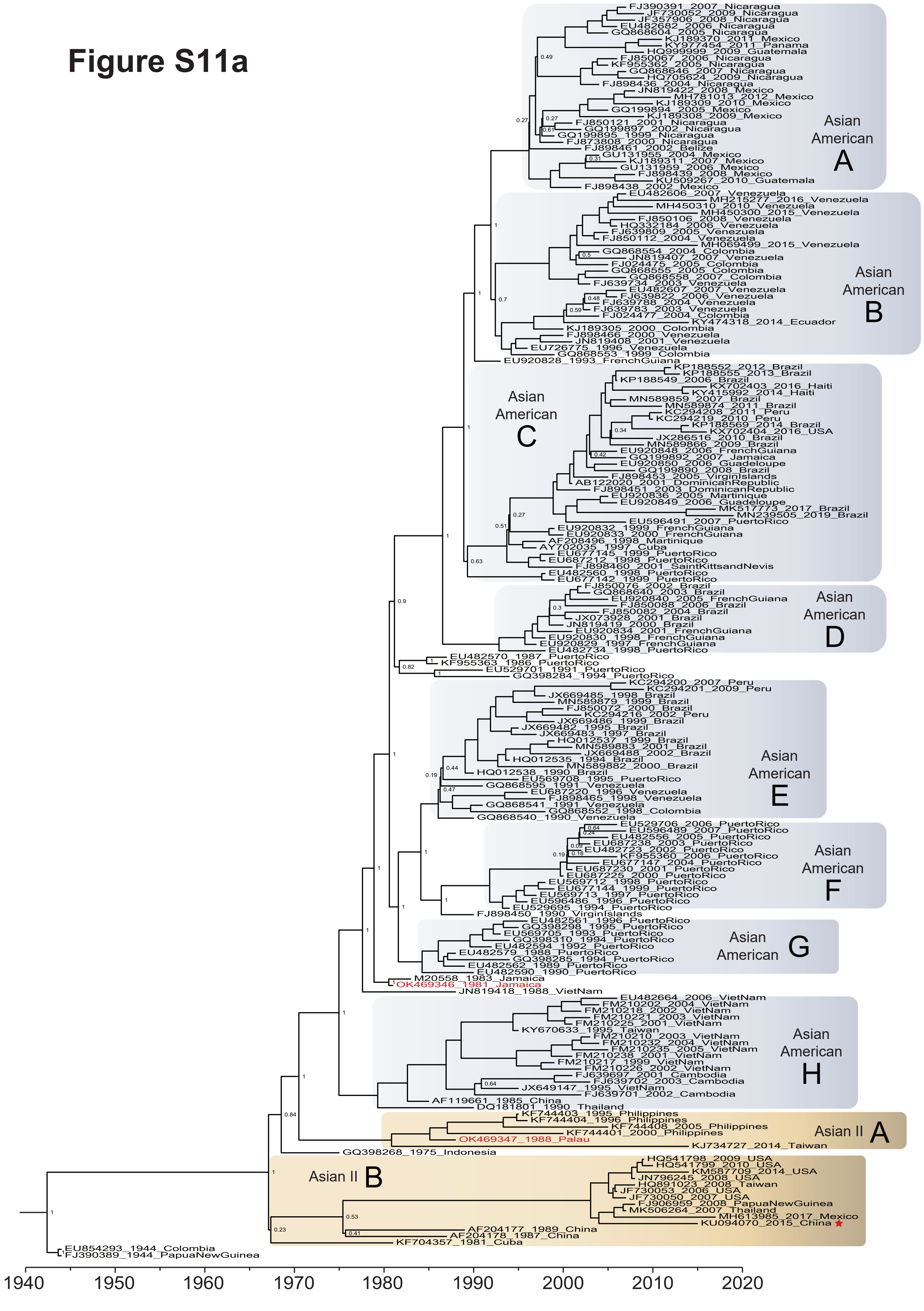
Asian American A
Asian American B
Asian American C
Asian American D
Asian American E
Asian American F
Asian American G
Asian American H
Asian II A
Asian II B
OK469346_1981_Jamaica
OK469347_1988_Palau
KU094070_2015_China
1940
1950
1960
1970
1980
1990
2000
2010
2020

Figure S11b

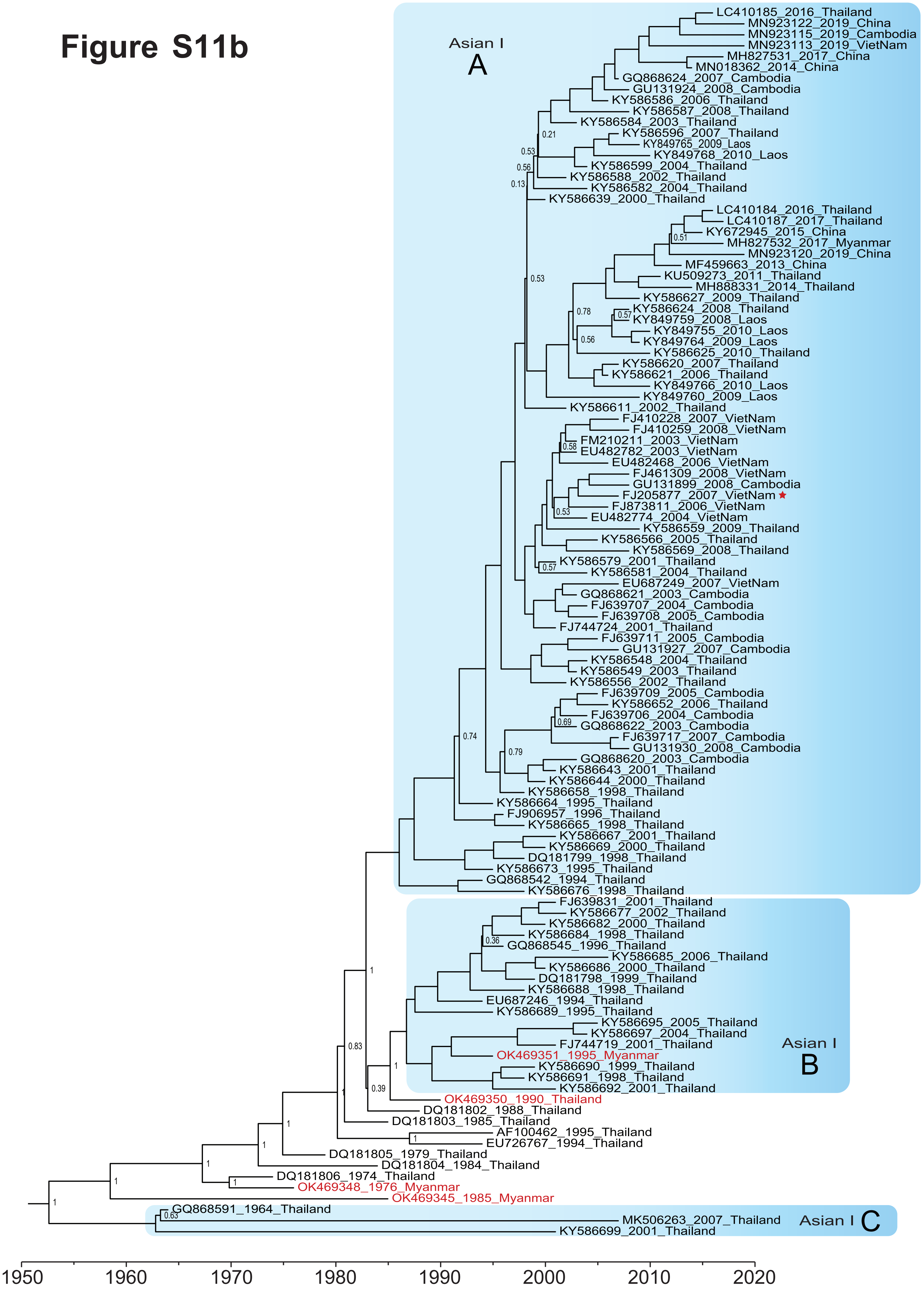

Asian I
A
LC410185_2016_Thailand
MN923122_2019_China
MN923115_2019_Cambodia
MN923113_2019_VietNam
MH827531_2017_China
MN018362_2014_China
GQ868624_2007_Cambodia
GU131924_2008_Cambodia
KY586586_2006_Thailand
KY586587_2008_Thailand
KY586584_2003_Thailand
KY586596_2007_Thailand
KY849765_2009_Laos
KY849768_2010_Laos
KY586599_2004_Thailand
KY586588_2002_Thailand
KY586582_2004_Thailand
KY586639_2000_Thailand
LC410184_2016_Thailand
LC410187_2017_Thailand
KY672945_2015_China
MH827532_2017_Myanmar
MN923120_2019_China
MF459663_2013_China
KU509273_2011_Thailand
MH888331_2014_Thailand
KY586627_2009_Thailand
KY586624_2008_Thailand
KY849759_2008_Laos
KY849755_2010_Laos
KY849764_2009_Laos
KY586625_2010_Thailand
KY586620_2007_Thailand
KY586621_2006_Thailand
KY849766_2010_Laos
KY849760_2009_Laos
KY586611_2002_Thailand
FJ410228_2007_VietNam
FJ410259_2008_VietNam
FM210211_2003_VietNam
EU482782_2003_VietNam
EU482468_2006_VietNam
FJ461309_2008_VietNam
GU131899_2008_Cambodia
FJ205877_2007_VietNam ★
FJ873811_2006_VietNam
EU482774_2004_VietNam
KY586559_2009_Thailand
KY586566_2005_Thailand
KY586569_2008_Thailand
KY586579_2001_Thailand
KY586581_2004_Thailand
EU687249_2007_VietNam
GQ868621_2003_Cambodia
FJ639707_2004_Cambodia
FJ639708_2005_Cambodia
FJ744724_2001_Thailand
FJ639711_2005_Cambodia
GU131927_2007_Cambodia
KY586548_2004_Thailand
KY586549_2003_Thailand
KY586556_2002_Thailand
FJ639709_2005_Cambodia
KY586652_2006_Thailand
FJ639706_2004_Cambodia
GQ868622_2003_Cambodia
FJ639717_2007_Cambodia
GU131930_2008_Cambodia
GQ868620_2003_Cambodia
KY586643_2001_Thailand
KY586644_2000_Thailand
KY586658_1998_Thailand
KY586664_1995_Thailand
FJ906957_1996_Thailand
KY586665_1998_Thailand
KY586667_2001_Thailand
KY586669_2000_Thailand
DQ181799_1998_Thailand
KY586673_1995_Thailand
GQ868542_1994_Thailand
KY586676_1998_Thailand
FJ639831_2001_Thailand
KY586677_2002_Thailand
KY586682_2000_Thailand
KY586684_1998_Thailand
GQ868545_1996_Thailand
KY586685_2006_Thailand
KY586686_2000_Thailand
DQ181798_1999_Thailand
KY586688_1998_Thailand
EU687246_1994_Thailand
KY586689_1995_Thailand
KY586695_2005_Thailand
KY586697_2004_Thailand
FJ744719_2001_Thailand
OK469351_1995_Myanmar
KY586690_1999_Thailand
KY586691_1998_Thailand
KY586692_2001_Thailand
Asian I
B
OK469350_1990_Thailand
DQ181802_1988_Thailand
DQ181803_1985_Thailand
AF100462_1995_Thailand
EU726767_1994_Thailand
DQ181805_1979_Thailand
DQ181804_1984_Thailand
DQ181806_1974_Thailand
OK469348_1976_Myanmar
OK469345_1985_Myanmar
GQ868591_1964_Thailand
MK506263_2007_Thailand
KY586699_2001_Thailand
Asian I C
0.21
0.53
0.56
0.13
0.53
0.51
0.78
0.57
0.56
0.58
0.53
0.57
0.69
0.74
0.79
0.36
1
0.83
1
0.39
1
1
1
1
1
1
0.63
1
1950
1960
1970
1980
1990
2000
2010
2020

Figure S11c

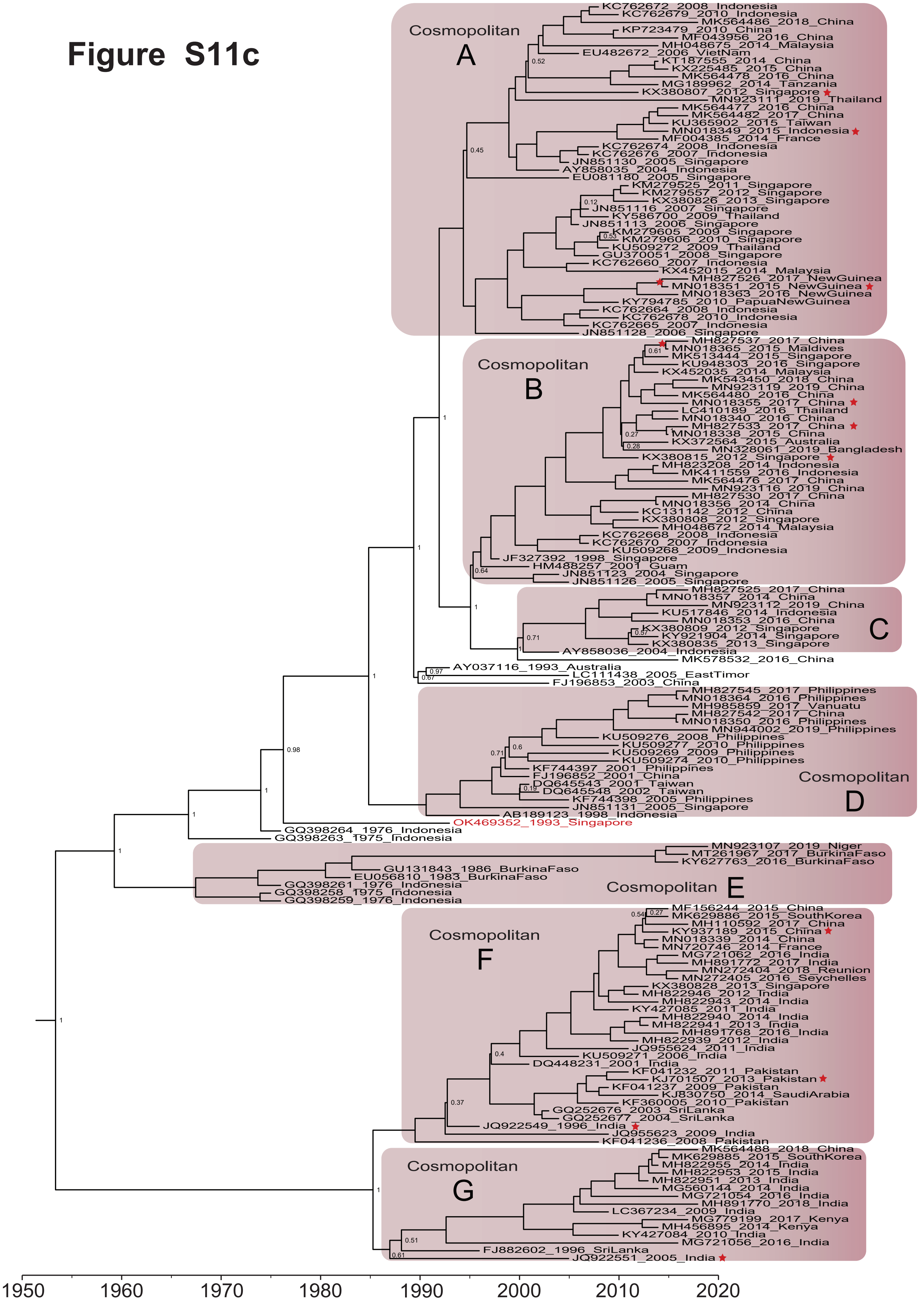

Cosmopolitan A
KC762672_2008_Indonesia
KC762679_2010_Indonesia
MK564486_2018_China
KP723479_2010_China
MF043956_2016_China
MH048675_2014_Malaysia
EU482672_2006_VietNam
0.52
KT187555_2014_China
KX225485_2015_China
MK564478_2016_China
MG189962_2014_Tanzania
KX380807_2012_Singapore
MN923111_2019_Thailand
MK564477_2016_China
MK564482_2017_China
KU365902_2015_Taiwan
MN018349_2015_Indonesia
MF004385_2014_France
0.45
KC762674_2008_Indonesia
KC762676_2007_Indonesia
JN851130_2005_Singapore
AY858035_2004_Indonesia
EU081180_2005_Singapore
KM279525_2011_Singapore
KM279557_2012_Singapore
KX380826_2013_Singapore
0.12
JN851116_2007_Singapore
KY586700_2009_Thailand
JN851113_2006_Singapore
KM279605_2009_Singapore
0.53
KM279606_2010_Singapore
KU509272_2009_Thailand
GU370051_2008_Singapore
KC762660_2007_Indonesia
KX452015_2014_Malaysia
MH827526_2017_NewGuinea
MN018351_2015_NewGuinea
MN018363_2016_NewGuinea
KY794785_2010_PapuaNewGuinea
KC762664_2008_Indonesia
KC762678_2010_Indonesia
KC762665_2007_Indonesia
JN851128_2006_Singapore
Cosmopolitan B
MH827537_2017_China
MN018365_2015_Maldives
0.61
MK513444_2015_Singapore
KU948303_2016_Singapore
KX452035_2014_Malaysia
MK543450_2018_China
MN923119_2019_China
MK564480_2016_China
MN018355_2017_China
LC410189_2016_Thailand
MN018340_2016_China
MH827533_2017_China
0.27
MN018338_2015_China
KX372564_2015_Australia
0.28
MN328061_2019_Bangladesh
KX380815_2012_Singapore
MH823208_2014_Indonesia
MK411559_2016_Indonesia
MK564476_2017_China
MN923116_2019_China
MH827530_2017_China
MN018356_2014_China
KC131142_2012_China
KX380808_2012_Singapore
MH048672_2014_Malaysia
KC762668_2008_Indonesia
KC762670_2007_Indonesia
KU509268_2009_Indonesia
JF327392_1998_Singapore
HM488257_2001_Guam
0.64
JN851123_2004_Singapore
JN851126_2005_Singapore
1
MH827525_2017_China
MN018357_2014_China
MN923112_2019_China
KU517846_2014_Indonesia
MN018353_2016_China
KX380809_2012_Singapore
0.57
KY921904_2014_Singapore
KX380835_2013_Singapore
0.71
AY858036_2004_Indonesia
MK578532_2016_China
C
0.97
AY037116_1993_Australia
0.67
LC111438_2005_EastTimor
FJ196853_2003_China
MH827545_2017_Philippines
MN018364_2016_Philippines
MH985859_2017_Vanuatu
MH827542_2017_China
MN018350_2016_Philippines
MN944002_2019_Philippines
KU509276_2008_Philippines
0.6
KU509277_2010_Philippines
0.71
KU509269_2009_Philippines
KU509274_2010_Philippines
KF744397_2001_Philippines
FJ196852_2001_China
DQ645543_2001_Taiwan
0.19
DQ645548_2002_Taiwan
KF744398_2005_Philippines
JN851131_2005_Singapore
AB189123_1998_Indonesia
Cosmopolitan D
0.98
OK469352_1993_Singapore
GQ398264_1976_Indonesia
GQ398263_1975_Indonesia
MN923107_2019_Niger
MT261967_2017_BurkinaFaso
KY627763_2016_BurkinaFaso
GU131843_1986_BurkinaFaso
EU056810_1983_BurkinaFaso
GQ398261_1976_Indonesia
GQ398258_1975_Indonesia
GQ398259_1976_Indonesia
Cosmopolitan E
Cosmopolitan F
MF156244_2015_China
0.54
0.27
MK629886_2015_SouthKorea
MH110592_2017_China
KY937189_2015_China
MN018339_2014_China
MN720746_2014_France
MG721062_2016_India
MH891772_2017_India
MN272404_2018_Reunion
MN272405_2016_Seychelles
KX380828_2013_Singapore
MH822946_2012_India
MH822943_2014_India
KY427085_2011_India
MH822940_2014_India
MH822941_2013_India
MH891768_2016_India
MH822939_2012_India
JQ955624_2011_India
KU509271_2006_India
0.4
DQ448231_2001_India
KF041232_2011_Pakistan
KJ701507_2013_Pakistan
KF041237_2009_Pakistan
KJ830750_2014_SaudiArabia
KF360005_2010_Pakistan
0.37
GQ252676_2003_SriLanka
GQ252677_2004_SriLanka
JQ922549_1996_India
JQ955623_2009_India
KF041236_2008_Pakistan
Cosmopolitan G
MK564488_2018_China
MK629885_2015_SouthKorea
MH822955_2014_India
MH822953_2015_India
MH822951_2013_India
MG560144_2014_India
MG721054_2016_India
MH891770_2018_India
LC367234_2009_India
MG779199_2017_Kenya
MH456895_2014_Kenya
KY427084_2010_India
0.51
MG721056_2016_India
FJ882602_1996_SriLanka
0.61
JQ922551_2005_India
1950
1960
1970
1980
1990
2000
2010
2020

Figure S11d

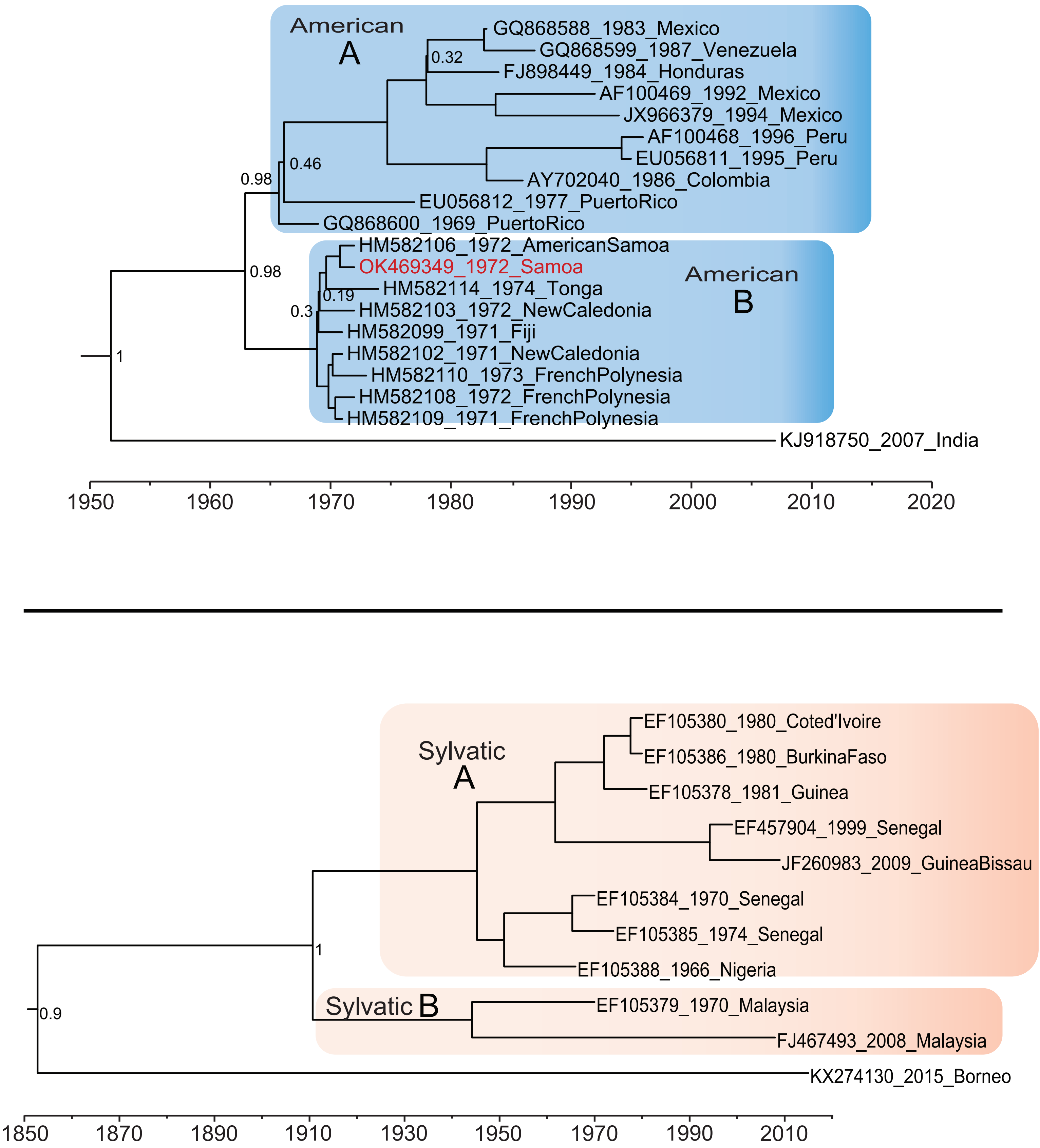
American A
GQ868588_1983_Mexico
GQ868599_1987_Venezuela
FJ898449_1984_Honduras
AF100469_1992_Mexico
JX966379_1994_Mexico
AF100468_1996_Peru
EU056811_1995_Peru
AY702040_1986_Colombia
EU056812_1977_PuertoRico
GQ868600_1969_PuertoRico
0.32
0.46
0.98
0.98
1
0.19
0.3
HM582106_1972_AmericanSamoa
OK469349_1972_Samoa
HM582114_1974_Tonga
HM582103_1972_NewCaledonia
HM582099_1971_Fiji
HM582102_1971_NewCaledonia
HM582110_1973_FrenchPolynesia
HM582108_1972_FrenchPolynesia
HM582109_1971_FrenchPolynesia
American B
KJ918750_2007_India
1950
1960
1970
1980
1990
2000
2010
2020
Sylvatic A
EF105380_1980_Coted'Ivoire
EF105386_1980_BurkinaFaso
EF105378_1981_Guinea
EF457904_1999_Senegal
JF260983_2009_GuineaBissau
EF105384_1970_Senegal
EF105385_1974_Senegal
EF105388_1966_Nigeria
1
0.9
Sylvatic B
EF105379_1970_Malaysia
FJ467493_2008_Malaysia
KX274130_2015_Borneo
1850
1870
1890
1910
1930
1950
1970
1990
2010

Supplemental Figure S12: DENV2 E Gene MCMC Trees. (a) DENV2 E gene MCMC tree, (b) Asian American genotype, (c) Asian I and Asian II genotypes, (d) Cosmopolitan genotype, (e) American and sylvatic genotypes. Posterior node support indicated for the major branches. Support values >0.8 have been eliminated in most instances for clarity. Red circles/font indicates newly sequenced isolates from the QUT Arbovirus collection. Branches under positive selection (see section 3.2.2) are indicated by a red star. Scale axis is representative of calendar years.

Figure S12a

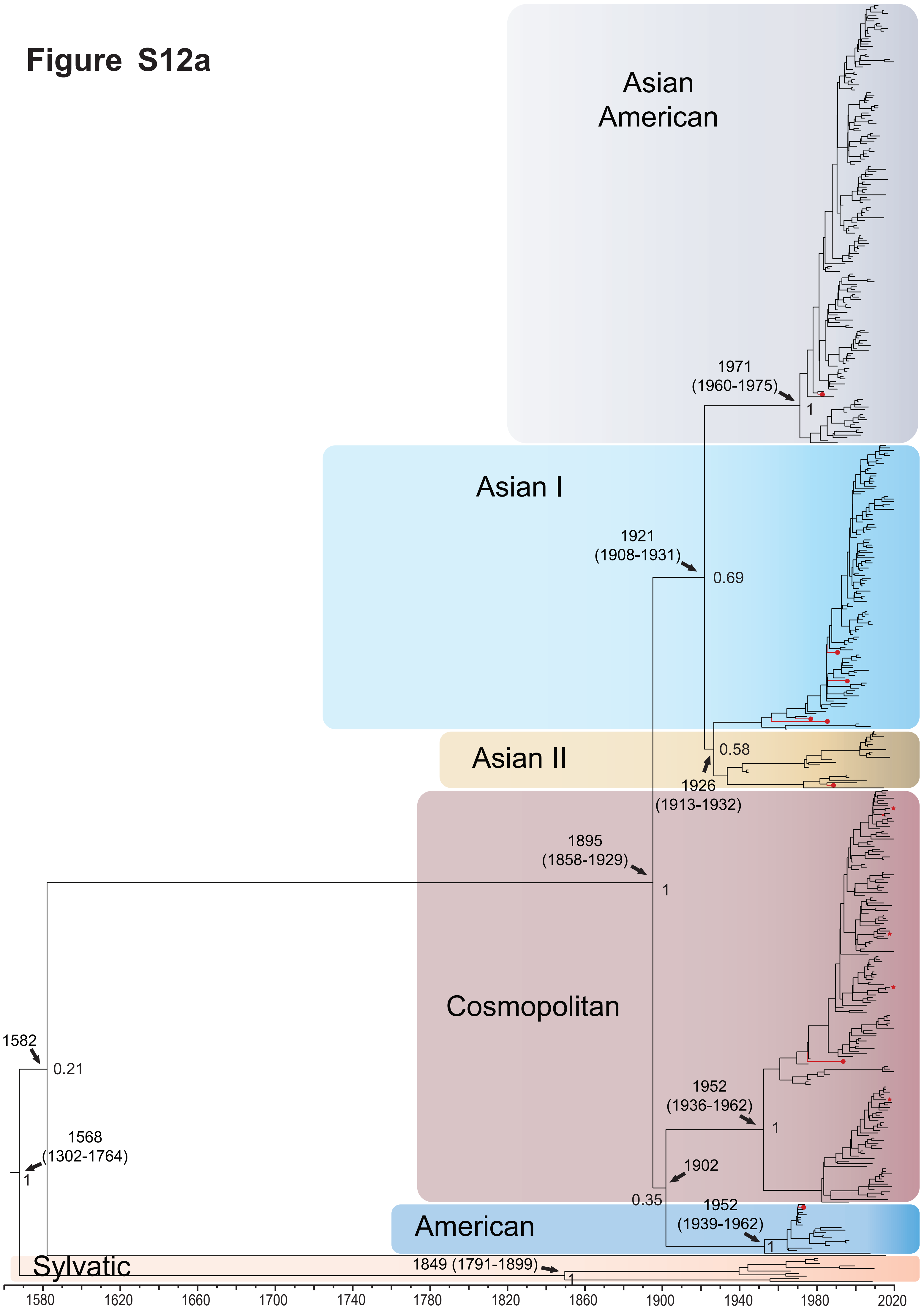

Asian American
1971 (1960-1975)
1
Asian I
1921 (1908-1931)
0.69
Asian II
0.58
1926 (1913-1932)
1895 (1858-1929)
1
Cosmopolitan
1582
0.21
1568 (1302-1764)
1
1952 (1936-1962)
1
1902
0.35
1952 (1939-1962)
American
1
Sylvatic
1849 (1791-1899)
1
1580
1620
1660
1700
1740
1780
1820
1860
1900
1940
1980
2020

Figure S12b

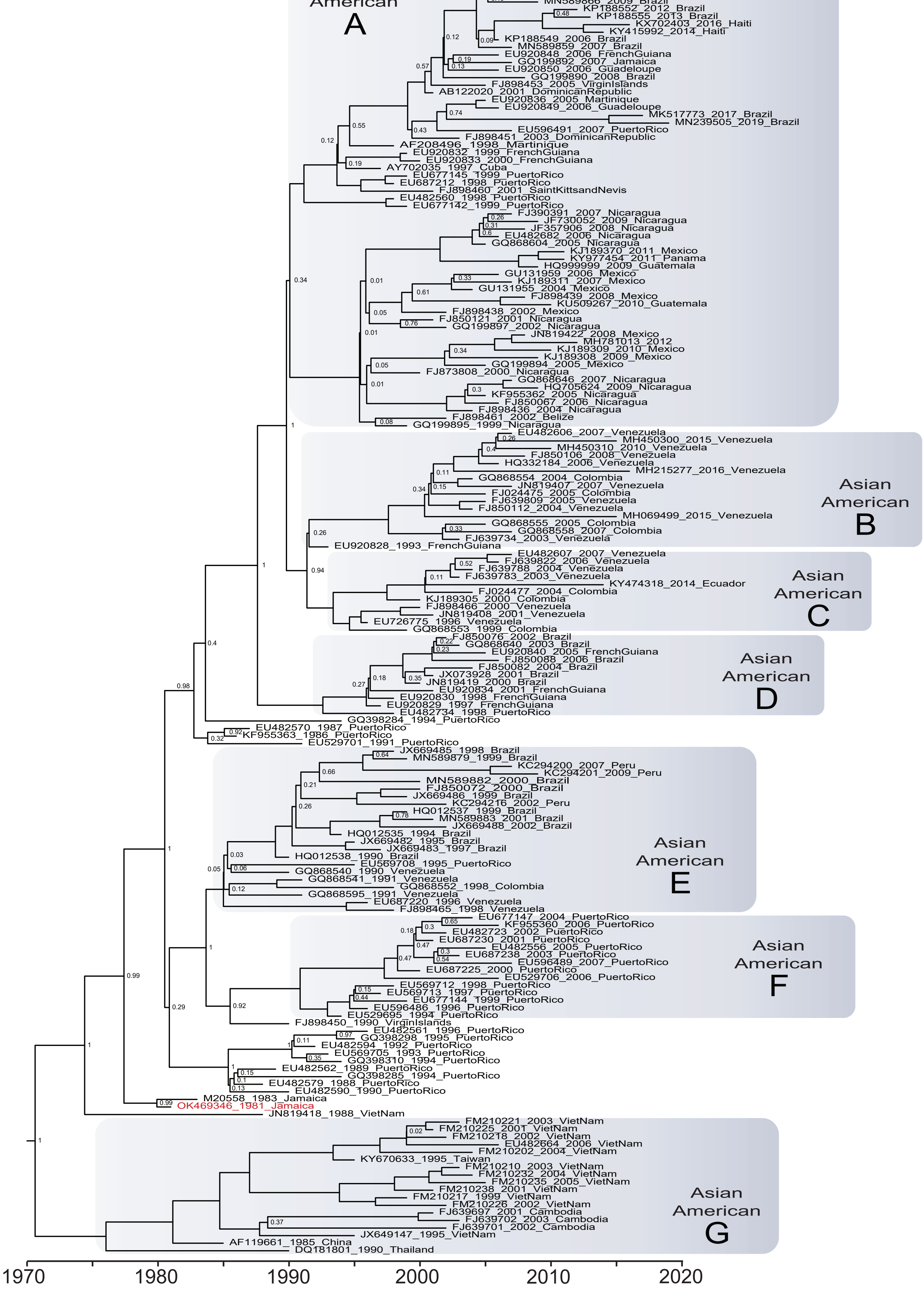
Asian American A
KC294208_2011_Peru
KC294219_2010_Peru
KP188569_2014_Brazil
KX702404_2016_USA
JX286516_2010_Brazil
MN589874_2011_Brazil
MN589866_2009_Brazil
KP188552_2012_Brazil
KP188555_2013_Brazil
KX702403_2016_Haiti
KY415992_2014_Haiti
KP188549_2006_Brazil
MN589859_2007_Brazil
EU920848_2006_FrenchGuiana
GQ199892_2007_Jamaica
EU920850_2006_Guadeloupe
GQ199890_2008_Brazil
FJ898453_2005_VirginIslands
AB122020_2001_DominicanRepublic
EU920836_2005_Martinique
EU920849_2006_Guadeloupe
MK517773_2017_Brazil
MN239505_2019_Brazil
EU596491_2007_PuertoRico
FJ898451_2003_DominicanRepublic
AF208496_1998_Martinique
EU920832_1999_FrenchGuiana
EU920833_2000_FrenchGuiana
AY702035_1997_Cuba
EU677145_1999_PuertoRico
EU687212_1998_PuertoRico
FJ898460_2001_SaintKittsandNevis
EU482560_1998_PuertoRico
EU677142_1999_PuertoRico
FJ390391_2007_Nicaragua
JF730052_2009_Nicaragua
JF357906_2008_Nicaragua
EU482682_2006_Nicaragua
GQ868604_2005_Nicaragua
KJ189370_2011_Mexico
KY977454_2011_Panama
HQ999999_2009_Guatemala
GU131959_2006_Mexico
KJ189311_2007_Mexico
GU131955_2004_Mexico
FJ898439_2008_Mexico
KU509267_2010_Guatemala
FJ898438_2002_Mexico
FJ850121_2001_Nicaragua
GQ199897_2002_Nicaragua
JN819422_2008_Mexico
MH781013_2012
KJ189309_2010_Mexico
KJ189308_2009_Mexico
GQ199894_2005_Mexico
FJ873808_2000_Nicaragua
GQ868646_2007_Nicaragua
HQ705624_2009_Nicaragua
KF955362_2005_Nicaragua
FJ850067_2006_Nicaragua
FJ898436_2004_Nicaragua
FJ898461_2002_Belize
GQ199895_1999_Nicaragua
Asian American B
EU482606_2007_Venezuela
MH450300_2015_Venezuela
MH450310_2010_Venezuela
FJ850106_2008_Venezuela
HQ332184_2006_Venezuela
MH215277_2016_Venezuela
GQ868554_2004_Colombia
JN819407_2007_Venezuela
FJ024475_2005_Colombia
FJ639809_2005_Venezuela
FJ850112_2004_Venezuela
MH069499_2015_Venezuela
GQ868555_2005_Colombia
GQ868558_2007_Colombia
FJ639734_2003_Venezuela
EU920828_1993_FrenchGuiana
Asian American C
EU482607_2007_Venezuela
FJ639822_2006_Venezuela
FJ639788_2004_Venezuela
FJ639783_2003_Venezuela
KY474318_2014_Ecuador
FJ024477_2004_Colombia
KJ189305_2000_Colombia
FJ898466_2000_Venezuela
JN819408_2001_Venezuela
EU726775_1996_Venezuela
GQ868553_1999_Colombia
Asian American D
FJ850076_2002_Brazil
GQ868640_2003_Brazil
EU920840_2005_FrenchGuiana
FJ850088_2006_Brazil
FJ850082_2004_Brazil
JX073928_2001_Brazil
JN819419_2000_Brazil
EU920834_2001_FrenchGuiana
EU920830_1998_FrenchGuiana
EU920829_1997_FrenchGuiana
EU482734_1998_PuertoRico
GQ398284_1994_PuertoRico
EU482570_1987_PuertoRico
KF955363_1986_PuertoRico
EU529701_1991_PuertoRico
Asian American E
JX669485_1998_Brazil
MN589879_1999_Brazil
KC294200_2007_Peru
KC294201_2009_Peru
MN589882_2000_Brazil
FJ850072_2000_Brazil
JX669486_1999_Brazil
KC294216_2002_Peru
HQ012537_1999_Brazil
MN589883_2001_Brazil
JX669488_2002_Brazil
HQ012535_1994_Brazil
JX669482_1995_Brazil
JX669483_1997_Brazil
HQ012538_1990_Brazil
EU569708_1995_PuertoRico
GQ868540_1990_Venezuela
GQ868541_1991_Venezuela
GQ868552_1998_Colombia
GQ868595_1991_Venezuela
EU687220_1996_Venezuela
FJ898465_1998_Venezuela
Asian American F
EU677147_2004_PuertoRico
KF955360_2006_PuertoRico
EU482723_2002_PuertoRico
EU687230_2001_PuertoRico
EU482556_2005_PuertoRico
EU687238_2003_PuertoRico
EU596489_2007_PuertoRico
EU687225_2000_PuertoRico
EU529706_2006_PuertoRico
EU569712_1998_PuertoRico
EU569713_1997_PuertoRico
EU677144_1999_PuertoRico
EU596486_1996_PuertoRico
EU529695_1994_PuertoRico
FJ898450_1990_VirginIslands
EU482561_1996_PuertoRico
GQ398298_1995_PuertoRico
EU482594_1992_PuertoRico
EU569705_1993_PuertoRico
GQ398310_1994_PuertoRico
EU482562_1989_PuertoRico
GQ398285_1994_PuertoRico
EU482579_1988_PuertoRico
EU482590_1990_PuertoRico
M20558_1983_Jamaica
OK469346_1981_Jamaica
JN819418_1988_VietNam
Asian American G
FM210221_2003_VietNam
FM210225_2001_VietNam
FM210218_2002_VietNam
EU482664_2006_VietNam
FM210202_2004_VietNam
KY670633_1995_Taiwan
FM210210_2003_VietNam
FM210232_2004_VietNam
FM210235_2005_VietNam
FM210238_2001_VietNam
FM210217_1999_VietNam
FM210226_2002_VietNam
FJ639697_2001_Cambodia
FJ639702_2003_Cambodia
FJ639701_2002_Cambodia
JX649147_1995_VietNam
AF119661_1985_China
DQ181801_1990_Thailand
1970
1980
1990
2000
2010
2020

Figure S12c

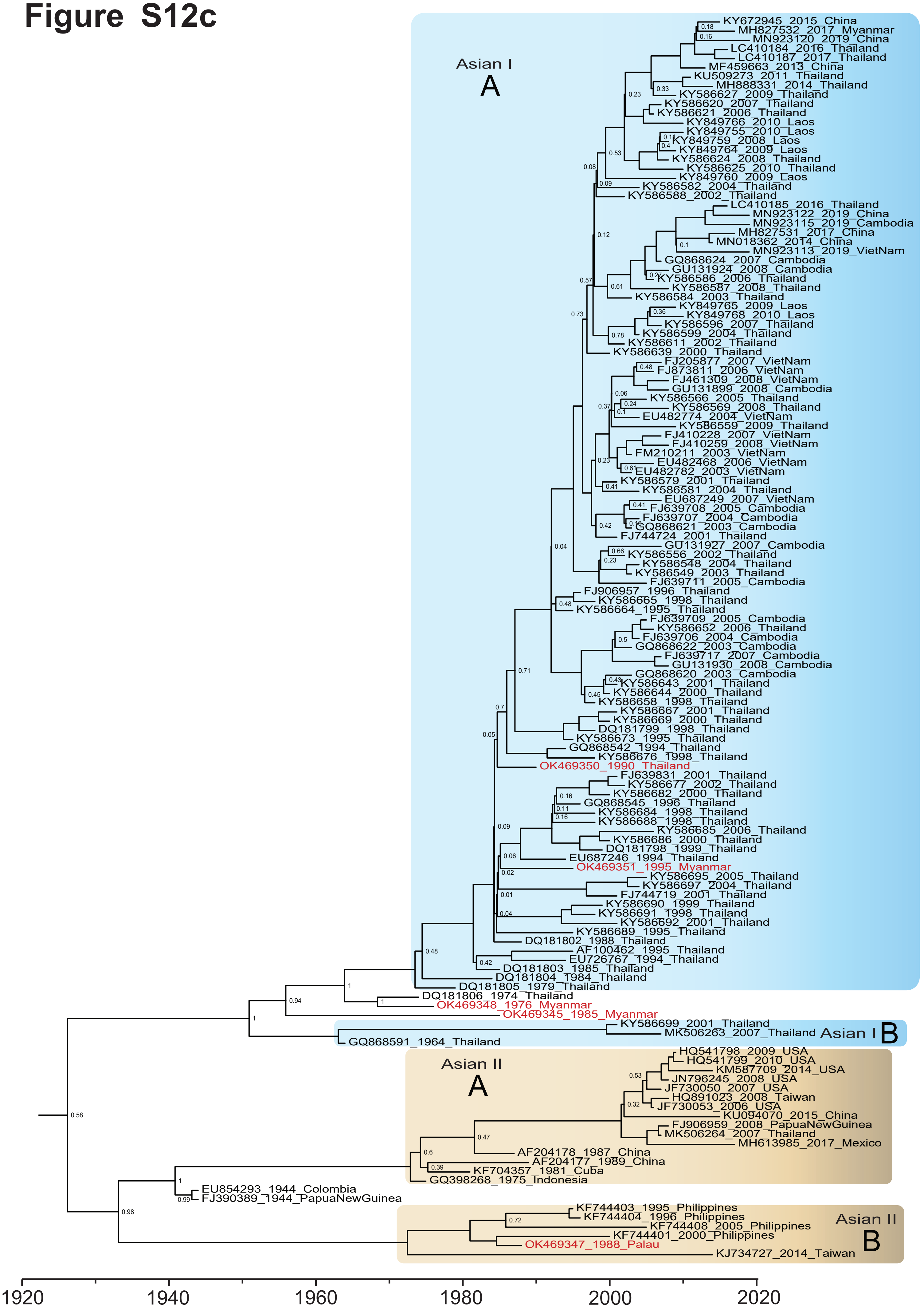

Asian I
A
KY672945_2015_China
MH827532_2017_Myanmar
MN923120_2019_China
LC410184_2016_Thailand
LC410187_2017_Thailand
MF459663_2013_China
KU509273_2011_Thailand
MH888331_2014_Thailand
KY586627_2009_Thailand
KY586620_2007_Thailand
KY586621_2006_Thailand
KY849766_2010_Laos
KY849755_2010_Laos
KY849759_2008_Laos
KY849764_2009_Laos
KY586624_2008_Thailand
KY586625_2010_Thailand
KY849760_2009_Laos
KY586582_2004_Thailand
KY586588_2002_Thailand
LC410185_2016_Thailand
MN923122_2019_China
MN923115_2019_Cambodia
MH827531_2017_China
MN018362_2014_China
MN923113_2019_VietNam
GQ868624_2007_Cambodia
GU131924_2008_Cambodia
KY586586_2006_Thailand
KY586587_2008_Thailand
KY586584_2003_Thailand
KY849765_2009_Laos
KY849768_2010_Laos
KY586596_2007_Thailand
KY586599_2004_Thailand
KY586611_2002_Thailand
KY586639_2000_Thailand
FJ205877_2007_VietNam
FJ873811_2006_VietNam
FJ461309_2008_VietNam
GU131899_2008_Cambodia
KY586566_2005_Thailand
KY586569_2008_Thailand
EU482774_2004_VietNam
KY586559_2009_Thailand
FJ410228_2007_VietNam
FJ410259_2008_VietNam
FM210211_2003_VietNam
EU482468_2006_VietNam
EU482782_2003_VietNam
KY586579_2001_Thailand
KY586581_2004_Thailand
EU687249_2007_VietNam
FJ639708_2005_Cambodia
FJ639707_2004_Cambodia
GQ868621_2003_Cambodia
FJ744724_2001_Thailand
GU131927_2007_Cambodia
KY586556_2002_Thailand
KY586548_2004_Thailand
KY586549_2003_Thailand
FJ639711_2005_Cambodia
FJ906957_1996_Thailand
KY586665_1998_Thailand
KY586664_1995_Thailand
FJ639709_2005_Cambodia
KY586652_2006_Thailand
FJ639706_2004_Cambodia
GQ868622_2003_Cambodia
FJ639717_2007_Cambodia
GU131930_2008_Cambodia
GQ868620_2003_Cambodia
KY586643_2001_Thailand
KY586644_2000_Thailand
KY586658_1998_Thailand
KY586667_2001_Thailand
KY586669_2000_Thailand
DQ181799_1998_Thailand
KY586673_1995_Thailand
GQ868542_1994_Thailand
KY586676_1998_Thailand
OK469350_1990_Thailand
FJ639831_2001_Thailand
KY586677_2002_Thailand
KY586682_2000_Thailand
GQ868545_1996_Thailand
KY586684_1998_Thailand
KY586688_1998_Thailand
KY586685_2006_Thailand
KY586686_2000_Thailand
DQ181798_1999_Thailand
EU687246_1994_Thailand
OK469351_1995_Myanmar
KY586695_2005_Thailand
KY586697_2004_Thailand
FJ744719_2001_Thailand
KY586690_1999_Thailand
KY586691_1998_Thailand
KY586692_2001_Thailand
KY586689_1995_Thailand
DQ181802_1988_Thailand
AF100462_1995_Thailand
EU726767_1994_Thailand
DQ181803_1985_Thailand
DQ181804_1984_Thailand
DQ181805_1979_Thailand
DQ181806_1974_Thailand
OK469348_1976_Myanmar
OK469345_1985_Myanmar
KY586699_2001_Thailand
MK506263_2007_Thailand
Asian I B
GQ868591_1964_Thailand
Asian II
A
HQ541798_2009_USA
HQ541799_2010_USA
KM587709_2014_USA
JN796245_2008_USA
JF730050_2007_USA
HQ891023_2008_Taiwan
JF730053_2006_USA
KU094070_2015_China
FJ906959_2008_PapuaNewGuinea
MK506264_2007_Thailand
MH613985_2017_Mexico
AF204178_1987_China
AF204177_1989_China
KF704357_1981_Cuba
GQ398268_1975_Indonesia
EU854293_1944_Colombia
FJ390389_1944_PapuaNewGuinea
KF744403_1995_Philippines
KF744404_1996_Philippines
KF744408_2005_Philippines
KF744401_2000_Philippines
OK469347_1988_Palau
KJ734727_2014_Taiwan
Asian II
B
1920
1940
1960
1980
2000
2020

Figure S12d

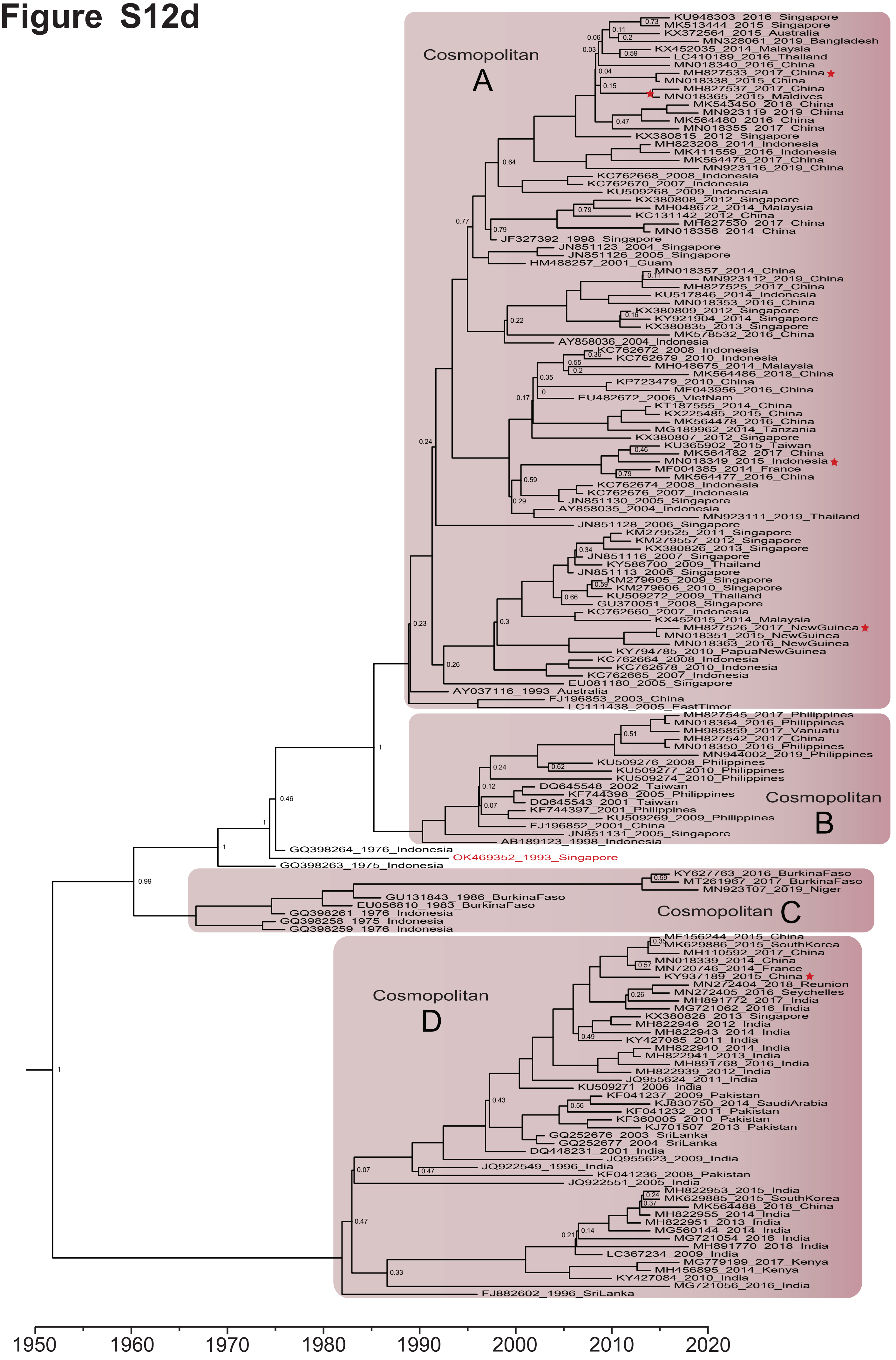

Cosmopolitan A
KU948303_2016_Singapore
MK513444_2015_Singapore
KX372564_2015_Australia
MN328061_2019_Bangladesh
KX452035_2014_Malaysia
LC410189_2016_Thailand
MN018340_2016_China
MH827533_2017_China
MN018338_2015_China
MH827537_2017_China
MN018365_2015_Maldives
MK543450_2018_China
MN923119_2019_China
MK564480_2016_China
MN018355_2017_China
KX380815_2012_Singapore
MH823208_2014_Indonesia
MK411559_2016_Indonesia
MK564476_2017_China
MN923116_2019_China
KC762668_2008_Indonesia
KC762670_2007_Indonesia
KU509268_2009_Indonesia
KX380808_2012_Singapore
MH048672_2014_Malaysia
KC131142_2012_China
MH827530_2017_China
MN018356_2014_China
JF327392_1998_Singapore
JN851123_2004_Singapore
JN851126_2005_Singapore
HM488257_2001_Guam
MN018357_2014_China
MN923112_2019_China
MH827525_2017_China
KU517846_2014_Indonesia
MN018353_2016_China
KX380809_2012_Singapore
KY921904_2014_Singapore
KX380835_2013_Singapore
MK578532_2016_China
AY858036_2004_Indonesia
KC762672_2008_Indonesia
KC762679_2010_Indonesia
MH048675_2014_Malaysia
MK564486_2018_China
KP723479_2010_China
MF043956_2016_China
EU482672_2006_VietNam
KT187555_2014_China
KX225485_2015_China
MK564478_2016_China
MG189962_2014_Tanzania
KX380807_2012_Singapore
KU365902_2015_Taiwan
MK564482_2017_China
MN018349_2015_Indonesia
MF004385_2014_France
MK564477_2016_China
KC762674_2008_Indonesia
KC762676_2007_Indonesia
JN851130_2005_Singapore
AY858035_2004_Indonesia
MN923111_2019_Thailand
JN851128_2006_Singapore
KM279525_2011_Singapore
KM279557_2012_Singapore
KX380826_2013_Singapore
JN851116_2007_Singapore
KY586700_2009_Thailand
JN851113_2006_Singapore
KM279605_2009_Singapore
KM279606_2010_Singapore
KU509272_2009_Thailand
GU370051_2008_Singapore
KC762660_2007_Indonesia
KX452015_2014_Malaysia
MH827526_2017_NewGuinea
MN018351_2015_NewGuinea
MN018363_2016_NewGuinea
KY794785_2010_PapuaNewGuinea
KC762664_2008_Indonesia
KC762678_2010_Indonesia
KC762665_2007_Indonesia
EU081180_2005_Singapore
AY037116_1993_Australia
FJ196853_2003_China
LC111438_2005_EastTimor
MH827545_2017_Philippines
MN018364_2016_Philippines
MH985859_2017_Vanuatu
MH827542_2017_China
MN018350_2016_Philippines
MN944002_2019_Philippines
KU509276_2008_Philippines
KU509277_2010_Philippines
KU509274_2010_Philippines
DQ645548_2002_Taiwan
KF744398_2005_Philippines
DQ645543_2001_Taiwan
KF744397_2001_Philippines
KU509269_2009_Philippines
FJ196852_2001_China
JN851131_2005_Singapore
AB189123_1998_Indonesia
Cosmopolitan B
GQ398264_1976_Indonesia
OK469352_1993_Singapore
GQ398263_1975_Indonesia
KY627763_2016_BurkinaFaso
MT261967_2017_BurkinaFaso
MN923107_2019_Niger
GU131843_1986_BurkinaFaso
EU056810_1983_BurkinaFaso
GQ398261_1976_Indonesia
GQ398258_1975_Indonesia
GQ398259_1976_Indonesia
Cosmopolitan C
MF156244_2015_China
MK629886_2015_SouthKorea
MH110592_2017_China
MN018339_2014_China
MN720746_2014_France
KY937189_2015_China
MN272404_2018_Reunion
MN272405_2016_Seychelles
MH891772_2017_India
MG721062_2016_India
KX380828_2013_Singapore
MH822946_2012_India
MH822943_2014_India
KY427085_2011_India
MH822940_2014_India
MH822941_2013_India
MH891768_2016_India
MH822939_2012_India
JQ955624_2011_India
KU509271_2006_India
KF041237_2009_Pakistan
KJ830750_2014_SaudiArabia
KF041232_2011_Pakistan
KF360005_2010_Pakistan
KJ701507_2013_Pakistan
GQ252676_2003_SriLanka
GQ252677_2004_SriLanka
DQ448231_2001_India
JQ955623_2009_India
JQ922549_1996_India
KF041236_2008_Pakistan
JQ922551_2005_India
MH822953_2015_India
MK629885_2015_SouthKorea
MK564488_2018_China
MH822955_2014_India
MH822951_2013_India
MG560144_2014_India
MG721054_2016_India
MH891770_2018_India
LC367234_2009_India
MG779199_2017_Kenya
MH456895_2014_Kenya
KY427084_2010_India
MG721056_2016_India
FJ882602_1996_SriLanka
Cosmopolitan D
1950
1960
1970
1980
1990
2000
2010
2020

Figure S12e

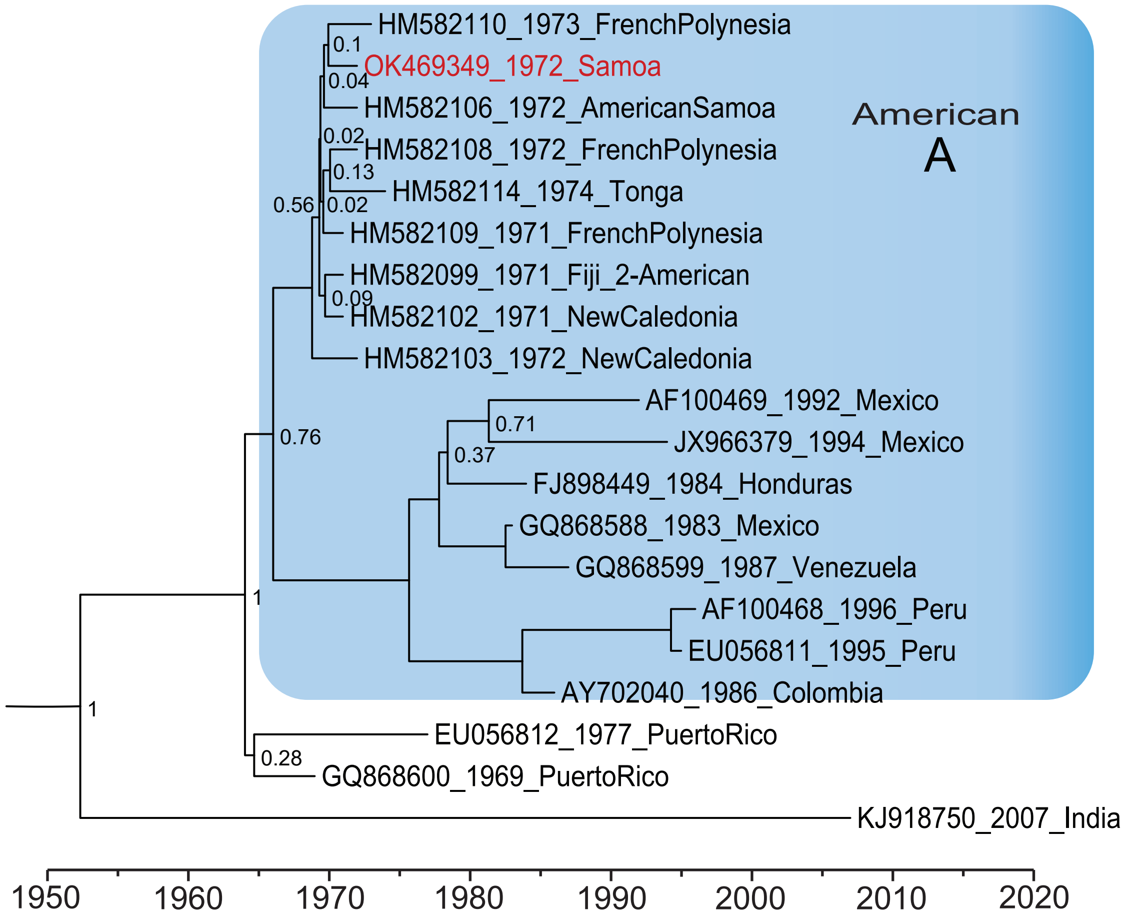
HM582110_1973_FrenchPolynesia
0.1
OK469349_1972_Samoa
0.04
HM582106_1972_AmericanSamoa
0.02
HM582108_1972_FrenchPolynesia
0.13
HM582114_1974_Tonga
0.56
0.02
HM582109_1971_FrenchPolynesia
HM582099_1971_Fiji_2-American
0.09
HM582102_1971_NewCaledonia
HM582103_1972_NewCaledonia
AF100469_1992_Mexico
0.71
JX966379_1994_Mexico
0.76
0.37
FJ898449_1984_Honduras
GQ868588_1983_Mexico
GQ868599_1987_Venezuela
1
AF100468_1996_Peru
EU056811_1995_Peru
AY702040_1986_Colombia
1
EU056812_1977_PuertoRico
0.28
GQ868600_1969_PuertoRico
KJ918750_2007_India
American
A
1950
1960
1970
1980
1990
2000
2010
2020

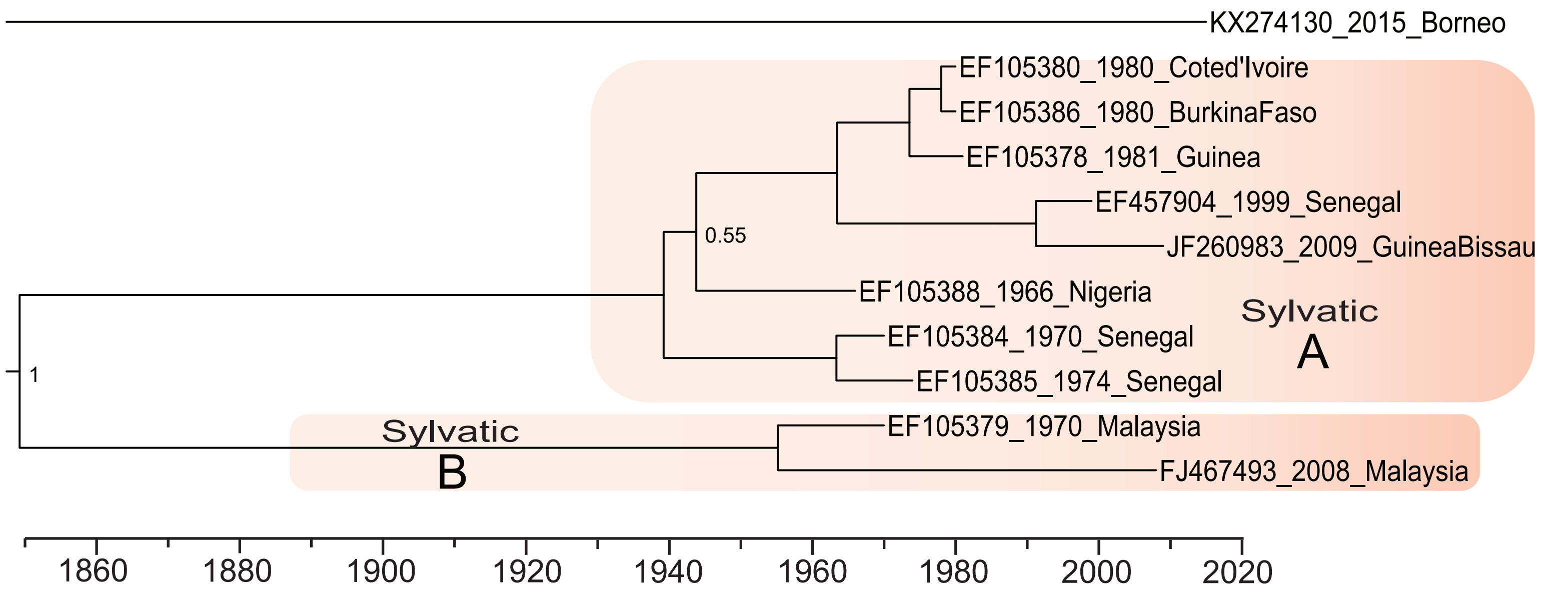
KX274130_2015_Borneo
EF105380_1980_Coted'Ivoire
EF105386_1980_BurkinaFaso
EF105378_1981_Guinea
EF457904_1999_Senegal
JF260983_2009_GuineaBissau
0.55
EF105388_1966_Nigeria
EF105384_1970_Senegal
EF105385_1974_Senegal
1
Sylvatic
A
EF105379_1970_Malaysia
FJ467493_2008_Malaysia
Sylvatic
B
1860
1880
1900
1920
1940
1960
1980
2000
2020

Supplemental Figure S13: DENV3 Whole Genome Genotypes MCMC Trees. (a) Genotype 3-III, (b) Genotype 3-II and 3-I. Posterior node support indicated for the major branches. Support values >0.8 have been eliminated in most instances for clarity. Red font indicates newly sequenced isolates from the QUT Arbovirus collection. Branches under positive selection (see section 3.2.2) are indicated by a red star. Scale axis is representative of calendar years.

Figure S13a

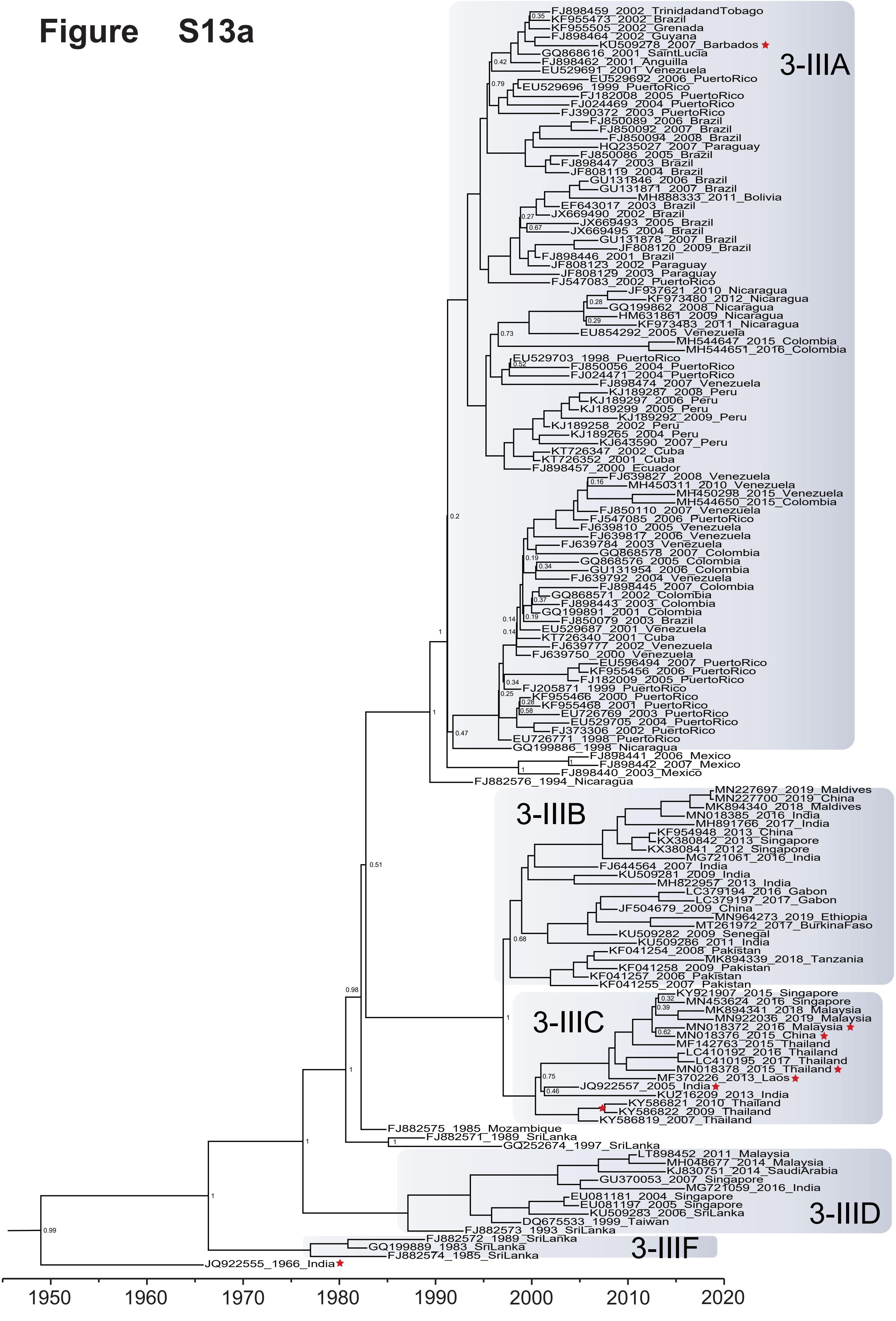

FJ898459_2002_TrinidadandTobago
KF955473_2002_Brazil
KF955505_2002_Grenada
FJ898464_2002_Guyana
KU509278_2007_Barbados
GQ868616_2001_SaintLucia
FJ898462_2001_Anguilla
EU529691_2001_Venezuela
EU529692_2006_PuertoRico
EU529696_1999_PuertoRico
FJ182008_2005_PuertoRico
FJ024469_2004_PuertoRico
FJ390372_2003_PuertoRico
FJ850089_2006_Brazil
FJ850092_2007_Brazil
FJ850094_2008_Brazil
HQ235027_2007_Paraguay
FJ850086_2005_Brazil
FJ898447_2003_Brazil
JF808119_2004_Brazil
GU131846_2006_Brazil
GU131871_2007_Brazil
MH888333_2011_Bolivia
EF643017_2003_Brazil
JX669490_2002_Brazil
JX669493_2005_Brazil
JX669495_2004_Brazil
GU131878_2007_Brazil
JF808120_2009_Brazil
FJ898446_2001_Brazil
JF808123_2002_Paraguay
JF808129_2003_Paraguay
FJ547083_2002_PuertoRico
JF937621_2010_Nicaragua
KF973480_2012_Nicaragua
GQ199862_2008_Nicaragua
HM631861_2009_Nicaragua
KF973483_2011_Nicaragua
EU854292_2005_Venezuela
MH544647_2015_Colombia
MH544651_2016_Colombia
EU529703_1998_PuertoRico
FJ850056_2004_PuertoRico
FJ024471_2004_PuertoRico
FJ898474_2007_Venezuela
KJ189287_2008_Peru
KJ189297_2006_Peru
KJ189299_2005_Peru
KJ189292_2009_Peru
KJ189258_2002_Peru
KJ189265_2004_Peru
KJ643590_2007_Peru
KT726347_2002_Cuba
KT726352_2001_Cuba
FJ898457_2000_Ecuador
FJ639827_2008_Venezuela
MH450311_2010_Venezuela
MH450298_2015_Venezuela
MH544650_2015_Colombia
FJ850110_2007_Venezuela
FJ547085_2006_PuertoRico
FJ639810_2005_Venezuela
FJ639817_2006_Venezuela
FJ639784_2003_Venezuela
GQ868578_2007_Colombia
GQ868576_2005_Colombia
GU131954_2006_Colombia
FJ639792_2004_Venezuela
FJ898445_2007_Colombia
GQ868571_2002_Colombia
FJ898443_2003_Colombia
GQ199891_2001_Colombia
FJ850079_2003_Brazil
EU529687_2001_Venezuela
KT726340_2001_Cuba
FJ639777_2002_Venezuela
FJ639750_2000_Venezuela
EU596494_2007_PuertoRico
KF955456_2006_PuertoRico
FJ182009_2005_PuertoRico
FJ205871_1999_PuertoRico
KF955466_2000_PuertoRico
KF955468_2001_PuertoRico
EU726769_2003_PuertoRico
EU529705_2004_PuertoRico
FJ373306_2002_PuertoRico
EU726771_1998_PuertoRico
GQ199886_1998_Nicaragua
FJ898441_2006_Mexico
FJ898442_2007_Mexico
FJ898440_2003_Mexico
FJ882576_1994_Nicaragua
3-IIIA
MN227697_2019_Maldives
MN227700_2019_China
MK894340_2018_Maldives
MN018385_2016_India
MH891766_2017_India
KF954948_2013_China
KX380842_2013_Singapore
KX380841_2012_Singapore
MG721061_2016_India
FJ644564_2007_India
KU509281_2009_India
MH822957_2013_India
LC379194_2016_Gabon
LC379197_2017_Gabon
JF504679_2009_China
MN964273_2019_Ethiopia
MT261972_2017_BurkinaFaso
KU509282_2009_Senegal
KU509286_2011_India
KF041254_2008_Pakistan
MK894339_2018_Tanzania
KF041258_2009_Pakistan
KF041257_2006_Pakistan
KF041255_2007_Pakistan
3-IIIB
KY921907_2015_Singapore
MN453624_2016_Singapore
MK894341_2018_Malaysia
MN922036_2019_Malaysia
MN018372_2016_Malaysia
MN018376_2015_China
MF142763_2015_Thailand
LC410192_2016_Thailand
LC410195_2017_Thailand
MN018378_2015_Thailand
MF370226_2013_Laos
JQ922557_2005_India
KU216209_2013_India
KY586821_2010_Thailand
KY586822_2009_Thailand
KY586819_2007_Thailand
3-IIIC
FJ882575_1985_Mozambique
FJ882571_1989_SriLanka
GQ252674_1997_SriLanka
LT898452_2011_Malaysia
MH048677_2014_Malaysia
KJ830751_2014_SaudiArabia
GU370053_2007_Singapore
MG721059_2016_India
EU081181_2004_Singapore
EU081197_2005_Singapore
KU509283_2006_SriLanka
DQ675533_1999_Taiwan
FJ882573_1993_SriLanka
3-IIID
FJ882572_1989_SriLanka
GQ199889_1983_SriLanka
FJ882574_1985_SriLanka
3-IIIF
JQ922555_1966_India
1950
1960
1970
1980
1990
2000
2010
2020

Figure S13b

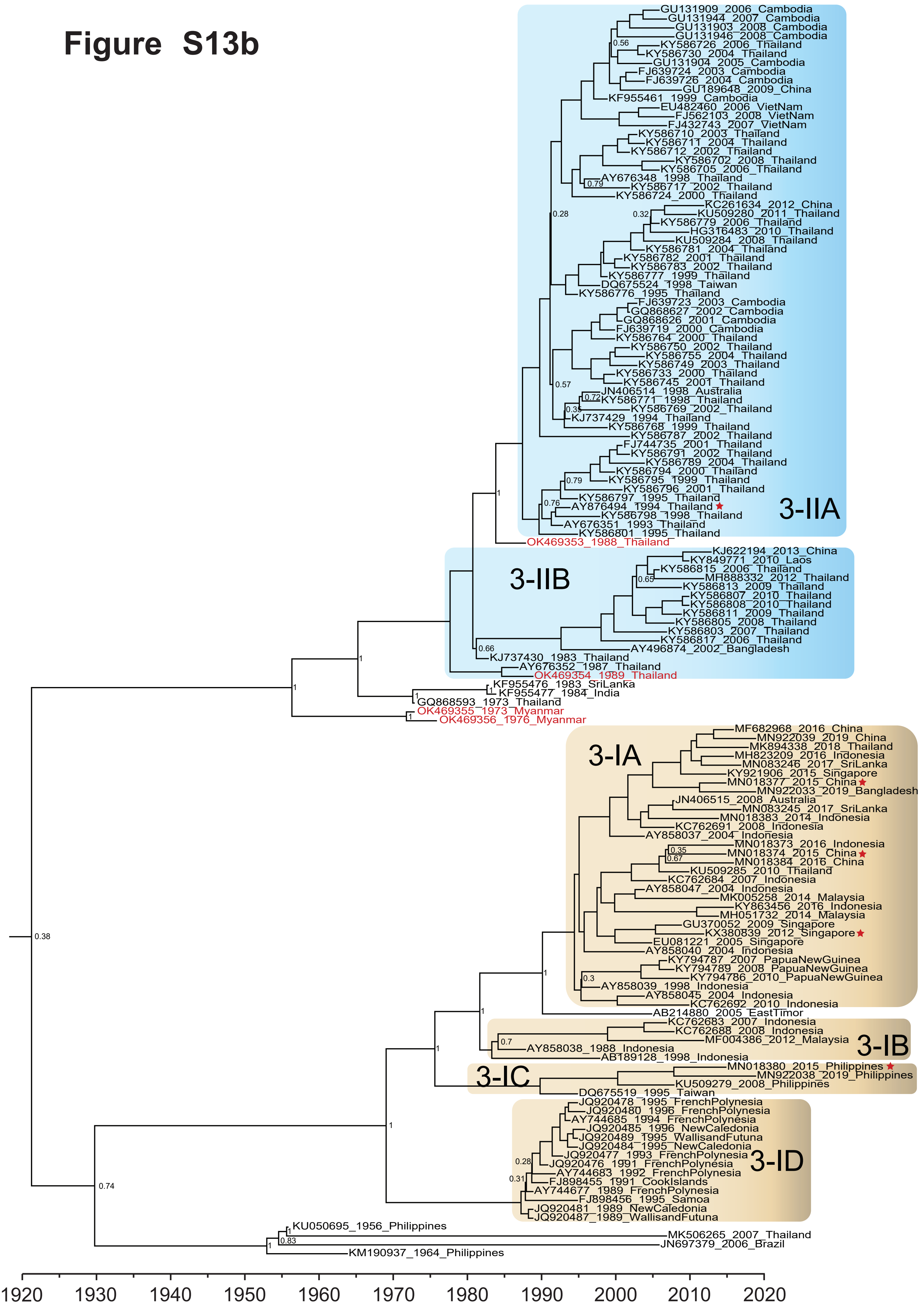

3-IIA
3-IIB
3-IA
3-IB
3-IC
3-ID
OK469353_1988_Thailand
OK469354_1989_Thailand
OK469355_1973_Myanmar
OK469356_1976_Myanmar
KF955476_1983_SriLanka
KF955477_1984_India
GQ868593_1973_Thailand
KU050695_1956_Philippines
MK506265_2007_Thailand
JN697379_2006_Brazil
KM190937_1964_Philippines
1920
1930
1940
1950
1960
1970
1980
1990
2000
2010
2020

Supplemental Figure S14: DENV3 E Gene MCMC Trees. (a) DENV3 E gene MCMC tree, (b) Genotype 3-I, (c) Genotypes 3-III and 3-II. Posterior node support indicated for the major branches. Support values >0.8 have been eliminated in most instances for clarity. Red circles/font indicates newly sequenced isolates from the QUT Arbovirus collection. Branches under positive selection (see section 3.2.2) are indicated by a red star. Scale axis is representative of calendar years.

Figure S14a

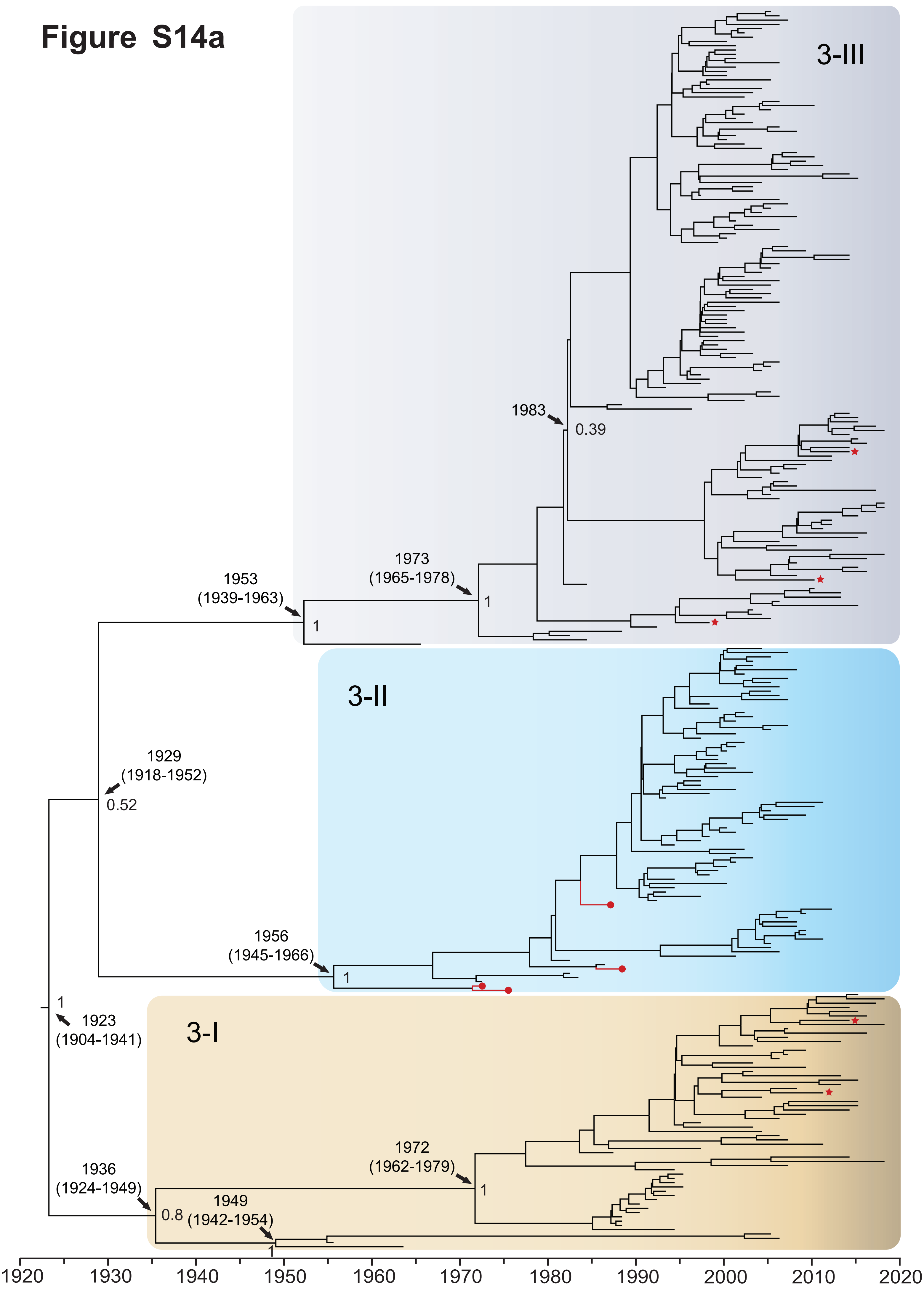

3-III
3-II
3-I
1983
0.39
1973
(1965-1978)
1
1953
(1939-1963)
1
1929
(1918-1952)
0.52
1956
(1945-1966)
1
1
1923
(1904-1941)
1972
(1962-1979)
1
1936
(1924-1949)
0.8
1949
(1942-1954)
1
1920
1930
1940
1950
1960
1970
1980
1990
2000
2010
2020

Figure S14b

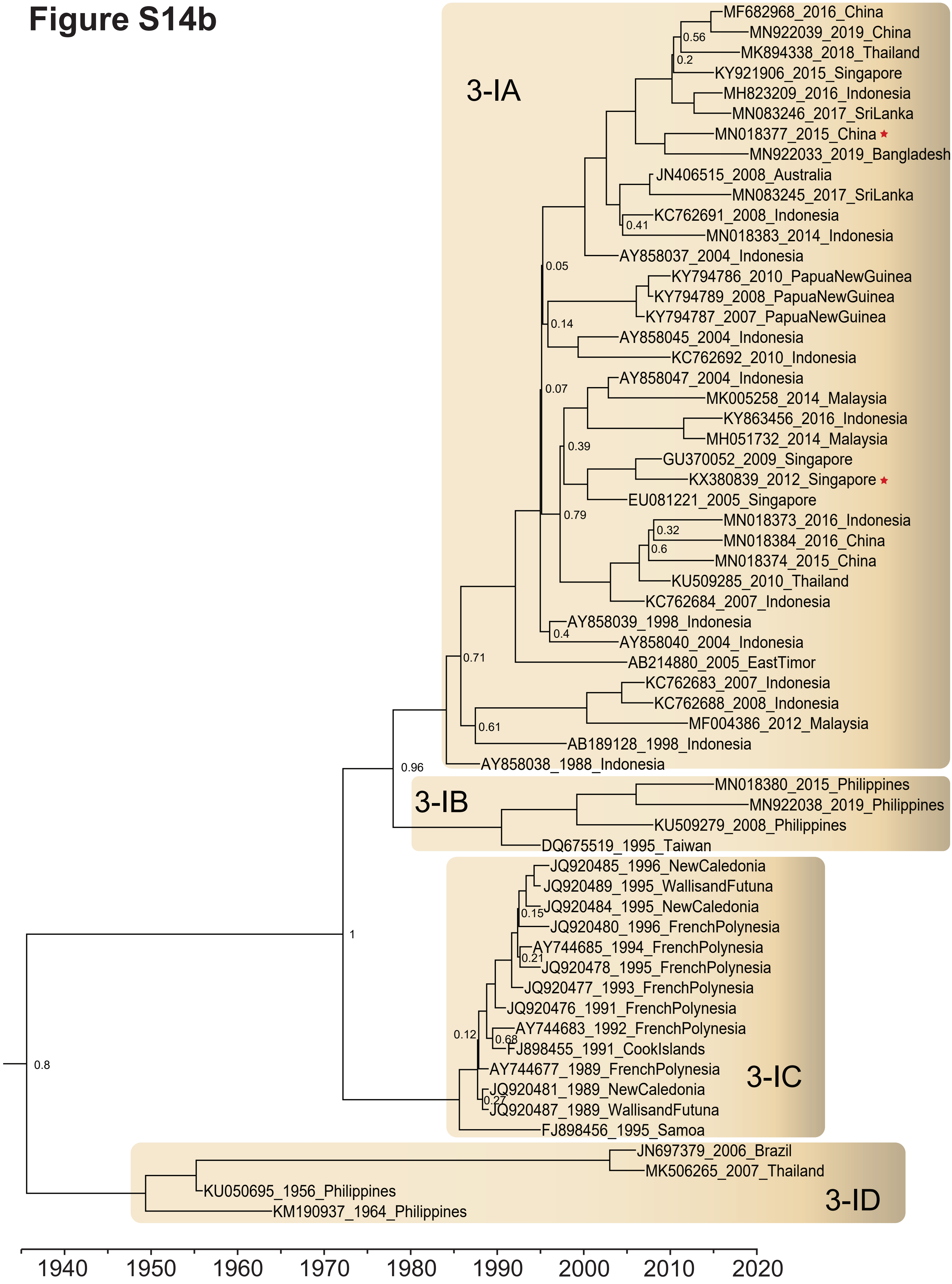

3-IA
MF682968_2016_China
MN922039_2019_China
MK894338_2018_Thailand
KY921906_2015_Singapore
MH823209_2016_Indonesia
MN083246_2017_SriLanka
MN018377_2015_China
MN922033_2019_Bangladesh
JN406515_2008_Australia
MN083245_2017_SriLanka
KC762691_2008_Indonesia
MN018383_2014_Indonesia
AY858037_2004_Indonesia
KY794786_2010_PapuaNewGuinea
KY794789_2008_PapuaNewGuinea
KY794787_2007_PapuaNewGuinea
AY858045_2004_Indonesia
KC762692_2010_Indonesia
AY858047_2004_Indonesia
MK005258_2014_Malaysia
KY863456_2016_Indonesia
MH051732_2014_Malaysia
GU370052_2009_Singapore
KX380839_2012_Singapore
EU081221_2005_Singapore
MN018373_2016_Indonesia
MN018384_2016_China
MN018374_2015_China
KU509285_2010_Thailand
KC762684_2007_Indonesia
AY858039_1998_Indonesia
AY858040_2004_Indonesia
AB214880_2005_EastTimor
KC762683_2007_Indonesia
KC762688_2008_Indonesia
MF004386_2012_Malaysia
AB189128_1998_Indonesia
AY858038_1988_Indonesia
3-IB
MN018380_2015_Philippines
MN922038_2019_Philippines
KU509279_2008_Philippines
DQ675519_1995_Taiwan
JQ920485_1996_NewCaledonia
JQ920489_1995_WallisandFutuna
JQ920484_1995_NewCaledonia
JQ920480_1996_FrenchPolynesia
AY744685_1994_FrenchPolynesia
JQ920478_1995_FrenchPolynesia
JQ920477_1993_FrenchPolynesia
JQ920476_1991_FrenchPolynesia
AY744683_1992_FrenchPolynesia
FJ898455_1991_CookIslands
AY744677_1989_FrenchPolynesia
JQ920481_1989_NewCaledonia
JQ920487_1989_WallisandFutuna
FJ898456_1995_Samoa
3-IC
JN697379_2006_Brazil
MK506265_2007_Thailand
KU050695_1956_Philippines
KM190937_1964_Philippines
3-ID
0.56
0.2
0.41
0.05
0.14
0.07
0.39
0.79
0.32
0.6
0.4
0.71
0.61
0.96
1
0.15
0.21
0.12
0.68
0.27
0.8
1940
1950
1960
1970
1980
1990
2000
2010
2020

Figure S14c

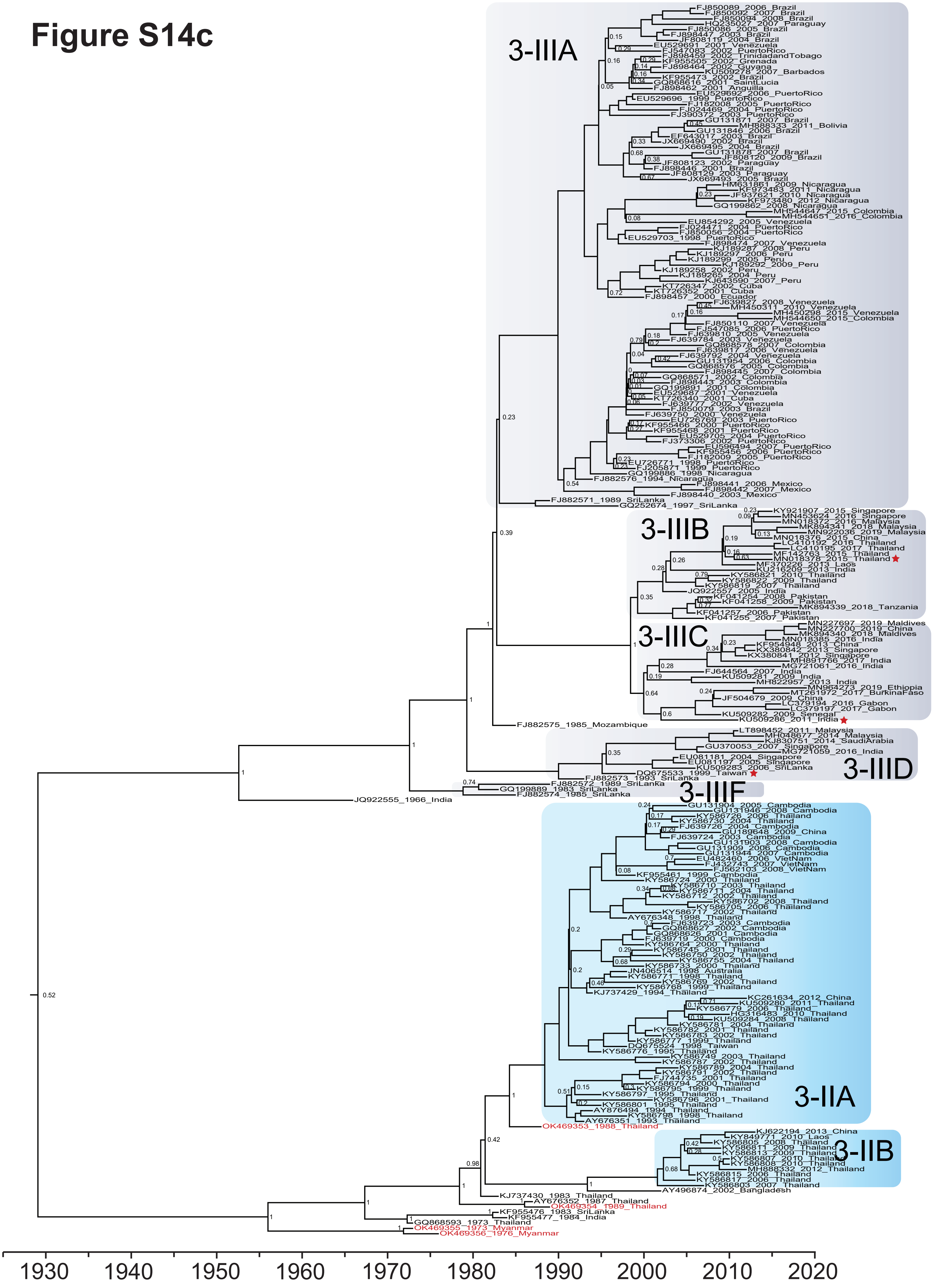

3-IIIA
3-IIIB
3-IIIC
3-IIID
3-IIIF
3-IIA
3-IIB
1930
1940
1950
1960
1970
1980
1990
2000
2010
2020

Supplemental Figure S15: DENV4 Whole Genome Genotypes MCMC Trees. (a) Genotype 4-II, (b) Genotypes 4-I, 4-III, and sylvatic. Posterior node support indicated for the major branches. Support values >0.8 have been eliminated in most instances for clarity. Red font indicates newly sequenced isolates from the QUT Arbovirus collection. Branches under positive selection (see section 3.2.2) are indicated by a red star. Scale axis is representative of calendar years.

Figure S15a

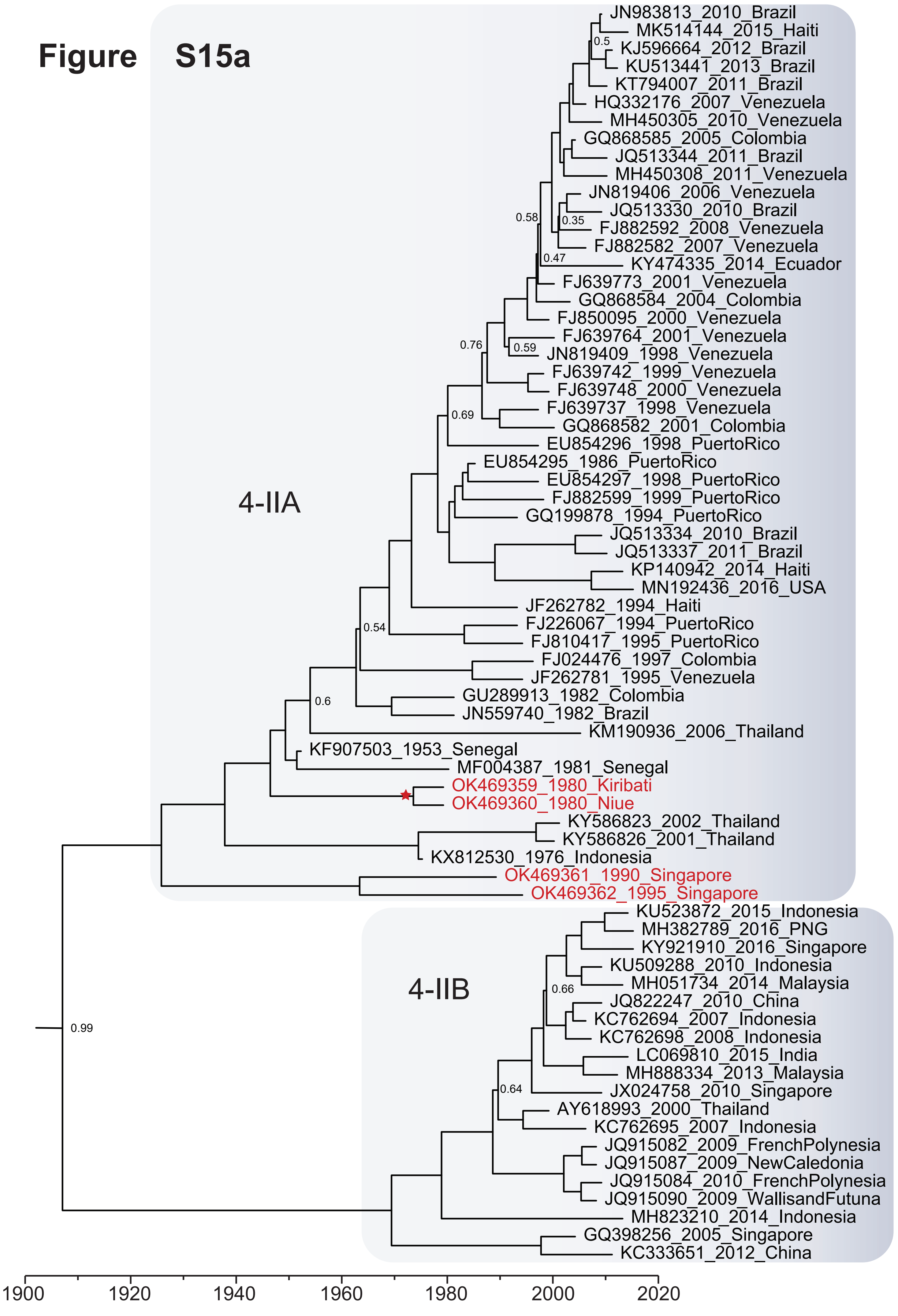

JN983813_2010_Brazil
MK514144_2015_Haiti
0.5
KJ596664_2012_Brazil
KU513441_2013_Brazil
KT794007_2011_Brazil
HQ332176_2007_Venezuela
MH450305_2010_Venezuela
GQ868585_2005_Colombia
JQ513344_2011_Brazil
MH450308_2011_Venezuela
JN819406_2006_Venezuela
JQ513330_2010_Brazil
0.58
0.35
FJ882592_2008_Venezuela
FJ882582_2007_Venezuela
0.47
KY474335_2014_Ecuador
FJ639773_2001_Venezuela
GQ868584_2004_Colombia
FJ850095_2000_Venezuela
FJ639764_2001_Venezuela
0.76
0.59
JN819409_1998_Venezuela
FJ639742_1999_Venezuela
FJ639748_2000_Venezuela
FJ639737_1998_Venezuela
0.69
GQ868582_2001_Colombia
EU854296_1998_PuertoRico
EU854295_1986_PuertoRico
EU854297_1998_PuertoRico
FJ882599_1999_PuertoRico
4-IIA
GQ199878_1994_PuertoRico
JQ513334_2010_Brazil
JQ513337_2011_Brazil
KP140942_2014_Haiti
MN192436_2016_USA
JF262782_1994_Haiti
FJ226067_1994_PuertoRico
0.54
FJ810417_1995_PuertoRico
FJ024476_1997_Colombia
JF262781_1995_Venezuela
GU289913_1982_Colombia
0.6
JN559740_1982_Brazil
KM190936_2006_Thailand
KF907503_1953_Senegal
MF004387_1981_Senegal
OK469359_1980_Kiribati
OK469360_1980_Niue
KY586823_2002_Thailand
KY586826_2001_Thailand
KX812530_1976_Indonesia
OK469361_1990_Singapore
OK469362_1995_Singapore
KU523872_2015_Indonesia
MH382789_2016_PNG
KY921910_2016_Singapore
KU509288_2010_Indonesia
MH051734_2014_Malaysia
4-IIB
0.66
JQ822247_2010_China
0.99
KC762694_2007_Indonesia
KC762698_2008_Indonesia
LC069810_2015_India
MH888334_2013_Malaysia
0.64
JX024758_2010_Singapore
AY618993_2000_Thailand
KC762695_2007_Indonesia
JQ915082_2009_FrenchPolynesia
JQ915087_2009_NewCaledonia
JQ915084_2010_FrenchPolynesia
JQ915090_2009_WallisandFutuna
MH823210_2014_Indonesia
GQ398256_2005_Singapore
KC333651_2012_China
1900
1920
1940
1960
1980
2000
2020

Figure S15b

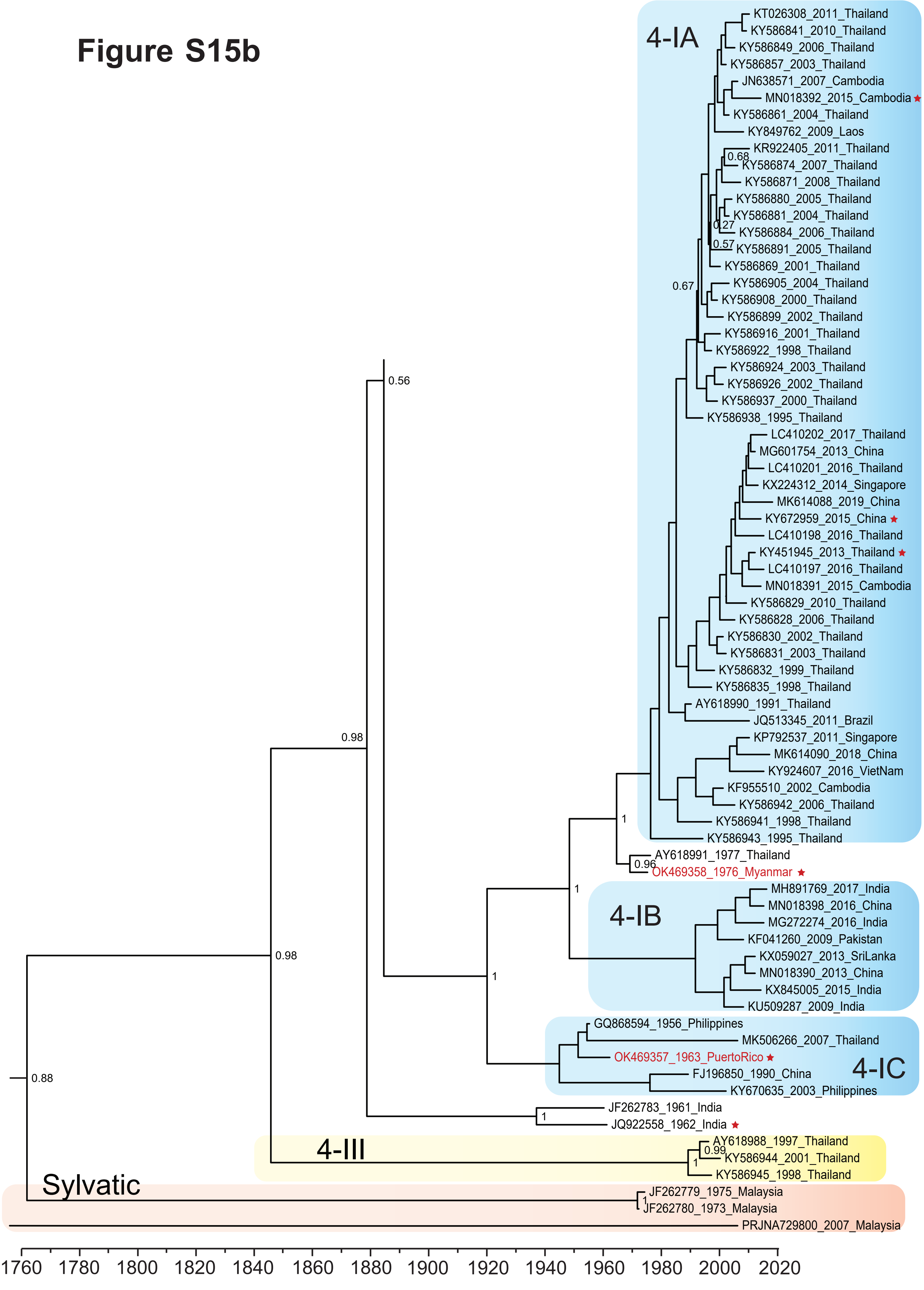
4-IA
KT026308_2011_Thailand
KY586841_2010_Thailand
KY586849_2006_Thailand
KY586857_2003_Thailand
JN638571_2007_Cambodia
MN018392_2015_Cambodia
KY586861_2004_Thailand
KY849762_2009_Laos
KR922405_2011_Thailand
0.68
KY586874_2007_Thailand
KY586871_2008_Thailand
KY586880_2005_Thailand
KY586881_2004_Thailand
0.27
KY586884_2006_Thailand
0.57
KY586891_2005_Thailand
KY586869_2001_Thailand
0.67
KY586905_2004_Thailand
KY586908_2000_Thailand
KY586899_2002_Thailand
KY586916_2001_Thailand
KY586922_1998_Thailand
KY586924_2003_Thailand
KY586926_2002_Thailand
KY586937_2000_Thailand
KY586938_1995_Thailand
LC410202_2017_Thailand
MG601754_2013_China
LC410201_2016_Thailand
KX224312_2014_Singapore
MK614088_2019_China
KY672959_2015_China
LC410198_2016_Thailand
KY451945_2013_Thailand
LC410197_2016_Thailand
MN018391_2015_Cambodia
KY586829_2010_Thailand
KY586828_2006_Thailand
KY586830_2002_Thailand
KY586831_2003_Thailand
KY586832_1999_Thailand
KY586835_1998_Thailand
AY618990_1991_Thailand
JQ513345_2011_Brazil
KP792537_2011_Singapore
MK614090_2018_China
KY924607_2016_VietNam
KF955510_2002_Cambodia
KY586942_2006_Thailand
KY586941_1998_Thailand
KY586943_1995_Thailand
0.56
0.98
1
AY618991_1977_Thailand
0.96
OK469358_1976_Myanmar
1
4-IB
MH891769_2017_India
MN018398_2016_China
MG272274_2016_India
KF041260_2009_Pakistan
KX059027_2013_SriLanka
MN018390_2013_China
KX845005_2015_India
KU509287_2009_India
0.98
1
GQ868594_1956_Philippines
MK506266_2007_Thailand
OK469357_1963_PuertoRico
FJ196850_1990_China
4-IC
KY670635_2003_Philippines
0.88
1
JF262783_1961_India
JQ922558_1962_India
4-III
AY618988_1997_Thailand
0.99
1
KY586944_2001_Thailand
KY586945_1998_Thailand
Sylvatic
JF262779_1975_Malaysia
1
JF262780_1973_Malaysia
PRJNA729800_2007_Malaysia
1760
1780
1800
1820
1840
1860
1880
1900
1920
1940
1960
1980
2000
2020

Supplemental Figure S16: DENV4 E Gene MCMC Trees. (a) DENV4 E gene MCMC tree, (b) Genotype 4-II, (c) Genotypes 4-I, 4-III, and sylvatic. Posterior node support indicated for the major branches. Support values >0.8 have been eliminated in most instances for clarity. Red circles/font indicates newly sequenced isolates from the QUT Arbovirus collection. Branches under positive selection (see section 3.2.2) are indicated by a red star. Scale axis is representative of calendar years.

Figure S16a

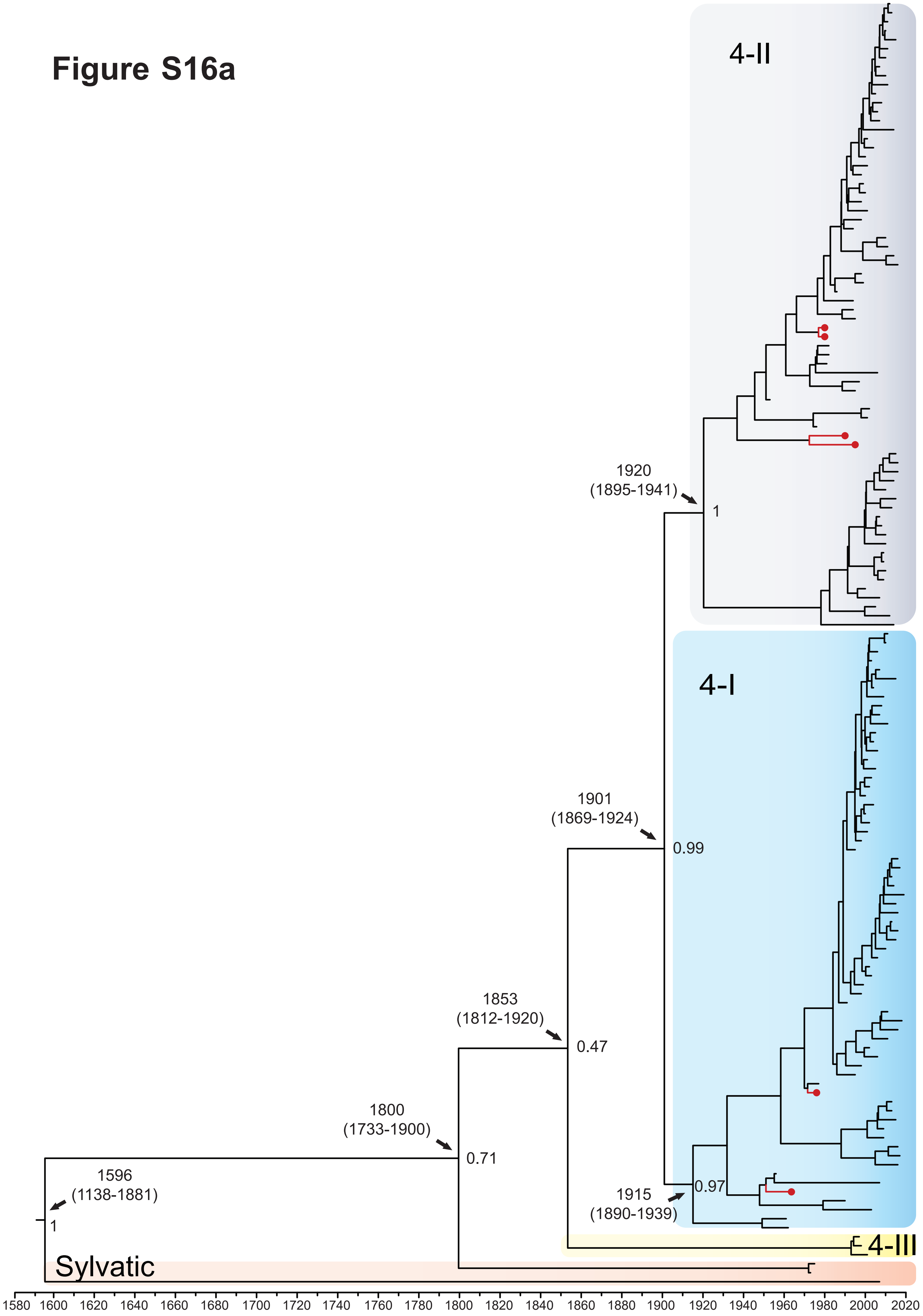

4-II
1920
(1895-1941)
1
4-I
1901
(1869-1924)
0.99
1853
(1812-1920)
0.47
1800
(1733-1900)
0.71
1915
(1890-1939)
0.97
1596
(1138-1881)
1
4-III
Sylvatic
1580 1600 1620 1640 1660 1680 1700 1720 1740 1760 1780 1800 1820 1840 1860 1880 1900 1920 1940 1960 1980 2000 2020

Figure S16b

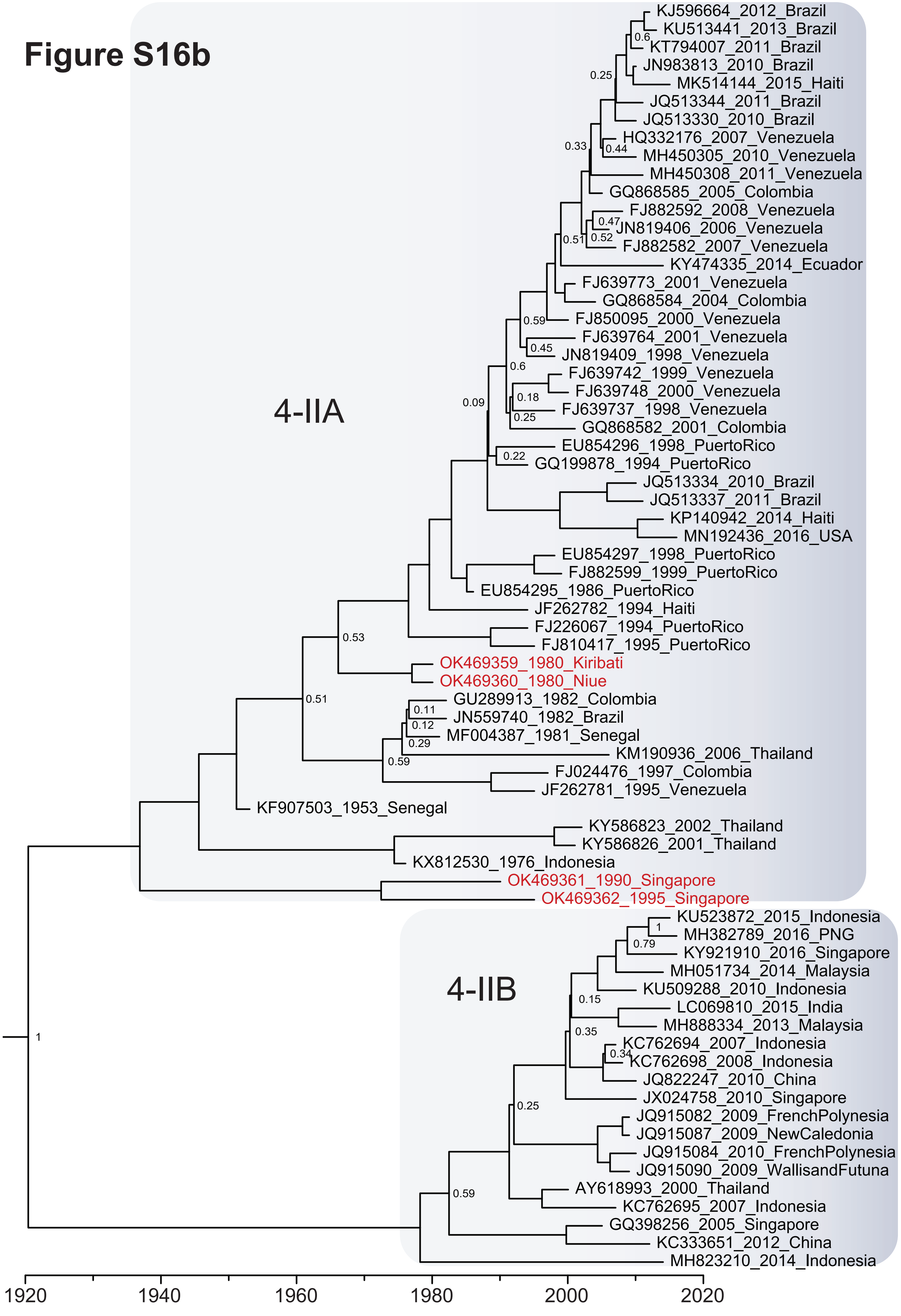
4-IIA
4-IIB
KJ596664_2012_Brazil
KU513441_2013_Brazil
KT794007_2011_Brazil
JN983813_2010_Brazil
MK514144_2015_Haiti
JQ513344_2011_Brazil
JQ513330_2010_Brazil
HQ332176_2007_Venezuela
MH450305_2010_Venezuela
MH450308_2011_Venezuela
GQ868585_2005_Colombia
FJ882592_2008_Venezuela
JN819406_2006_Venezuela
FJ882582_2007_Venezuela
KY474335_2014_Ecuador
FJ639773_2001_Venezuela
GQ868584_2004_Colombia
FJ850095_2000_Venezuela
FJ639764_2001_Venezuela
JN819409_1998_Venezuela
FJ639742_1999_Venezuela
FJ639748_2000_Venezuela
FJ639737_1998_Venezuela
GQ868582_2001_Colombia
EU854296_1998_PuertoRico
GQ199878_1994_PuertoRico
JQ513334_2010_Brazil
JQ513337_2011_Brazil
KP140942_2014_Haiti
MN192436_2016_USA
EU854297_1998_PuertoRico
FJ882599_1999_PuertoRico
EU854295_1986_PuertoRico
JF262782_1994_Haiti
FJ226067_1994_PuertoRico
FJ810417_1995_PuertoRico
OK469359_1980_Kiribati
OK469360_1980_Niue
GU289913_1982_Colombia
JN559740_1982_Brazil
MF004387_1981_Senegal
KM190936_2006_Thailand
FJ024476_1997_Colombia
JF262781_1995_Venezuela
KF907503_1953_Senegal
KY586823_2002_Thailand
KY586826_2001_Thailand
KX812530_1976_Indonesia
OK469361_1990_Singapore
OK469362_1995_Singapore
KU523872_2015_Indonesia
MH382789_2016_PNG
KY921910_2016_Singapore
MH051734_2014_Malaysia
KU509288_2010_Indonesia
LC069810_2015_India
MH888334_2013_Malaysia
KC762694_2007_Indonesia
KC762698_2008_Indonesia
JQ822247_2010_China
JX024758_2010_Singapore
JQ915082_2009_FrenchPolynesia
JQ915087_2009_NewCaledonia
JQ915084_2010_FrenchPolynesia
JQ915090_2009_WallisandFutuna
AY618993_2000_Thailand
KC762695_2007_Indonesia
GQ398256_2005_Singapore
KC333651_2012_China
MH823210_2014_Indonesia
0.6
0.25
0.33
0.44
0.47
0.51
0.52
0.59
0.45
0.6
0.09
0.18
0.25
0.22
0.53
0.51
0.11
0.12
0.29
0.59
1
1
0.79
0.15
0.35
0.34
0.25
0.59
1920
1940
1960
1980
2000
2020

Figure S16c

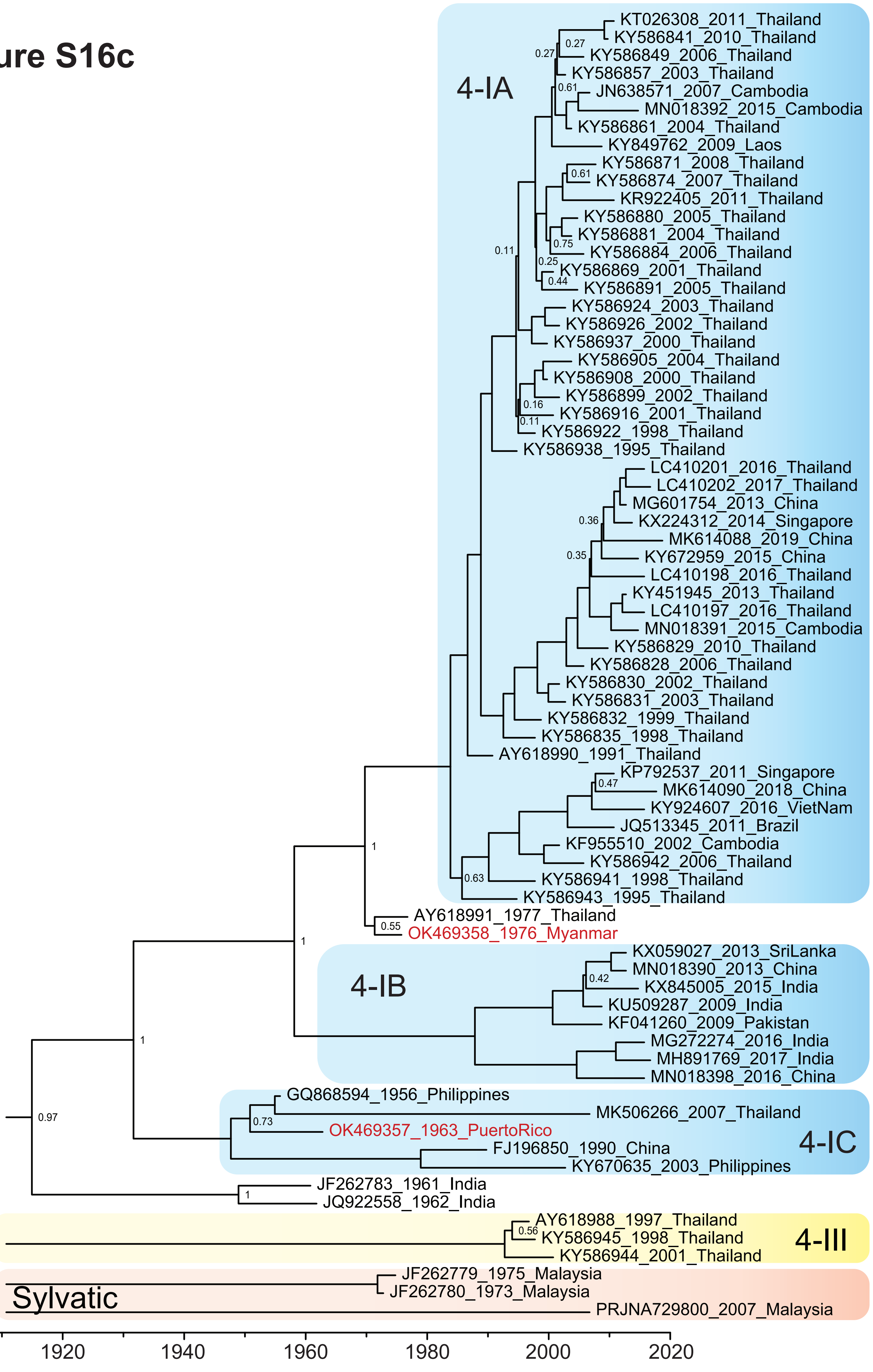

4-IA
KT026308_2011_Thailand
KY586841_2010_Thailand
KY586849_2006_Thailand
KY586857_2003_Thailand
JN638571_2007_Cambodia
MN018392_2015_Cambodia
KY586861_2004_Thailand
KY849762_2009_Laos
KY586871_2008_Thailand
KY586874_2007_Thailand
KR922405_2011_Thailand
KY586880_2005_Thailand
KY586881_2004_Thailand
KY586884_2006_Thailand
KY586869_2001_Thailand
KY586891_2005_Thailand
KY586924_2003_Thailand
KY586926_2002_Thailand
KY586937_2000_Thailand
KY586905_2004_Thailand
KY586908_2000_Thailand
KY586899_2002_Thailand
KY586916_2001_Thailand
KY586922_1998_Thailand
KY586938_1995_Thailand
LC410201_2016_Thailand
LC410202_2017_Thailand
MG601754_2013_China
KX224312_2014_Singapore
MK614088_2019_China
KY672959_2015_China
LC410198_2016_Thailand
KY451945_2013_Thailand
LC410197_2016_Thailand
MN018391_2015_Cambodia
KY586829_2010_Thailand
KY586828_2006_Thailand
KY586830_2002_Thailand
KY586831_2003_Thailand
KY586832_1999_Thailand
KY586835_1998_Thailand
AY618990_1991_Thailand
KP792537_2011_Singapore
MK614090_2018_China
KY924607_2016_VietNam
JQ513345_2011_Brazil
KF955510_2002_Cambodia
KY586942_2006_Thailand
KY586941_1998_Thailand
KY586943_1995_Thailand
AY618991_1977_Thailand
OK469358_1976_Myanmar
4-IB
KX059027_2013_SriLanka
MN018390_2013_China
KX845005_2015_India
KU509287_2009_India
KF041260_2009_Pakistan
MG272274_2016_India
MH891769_2017_India
MN018398_2016_China
GQ868594_1956_Philippines
MK506266_2007_Thailand
OK469357_1963_PuertoRico
FJ196850_1990_China
KY670635_2003_Philippines
4-IC
JF262783_1961_India
JQ922558_1962_India
AY618988_1997_Thailand
KY586945_1998_Thailand
KY586944_2001_Thailand
4-III
JF262779_1975_Malaysia
JF262780_1973_Malaysia
PRJNA729800_2007_Malaysia
Sylvatic
0.27
0.27
0.61
0.61
0.75
0.25
0.44
0.11
0.16
0.11
0.36
0.35
0.47
0.63
1
0.55
1
0.42
1
0.73
0.97
1
0.56
1900
1920
1940
1960
1980
2000
2020
